# One-Pot Synthesis of Difluorobicyclo[1.1.1]pentanes from α-Allyldiazoacetates

**DOI:** 10.1021/acs.orglett.3c01664

**Published:** 2023-07-10

**Authors:** Jack C. Sharland, Huw M. L. Davies

**Affiliations:** Department of Chemistry, Emory University, 1515 Dickey Drive, Atlanta Georgia 30322, United States

## Abstract

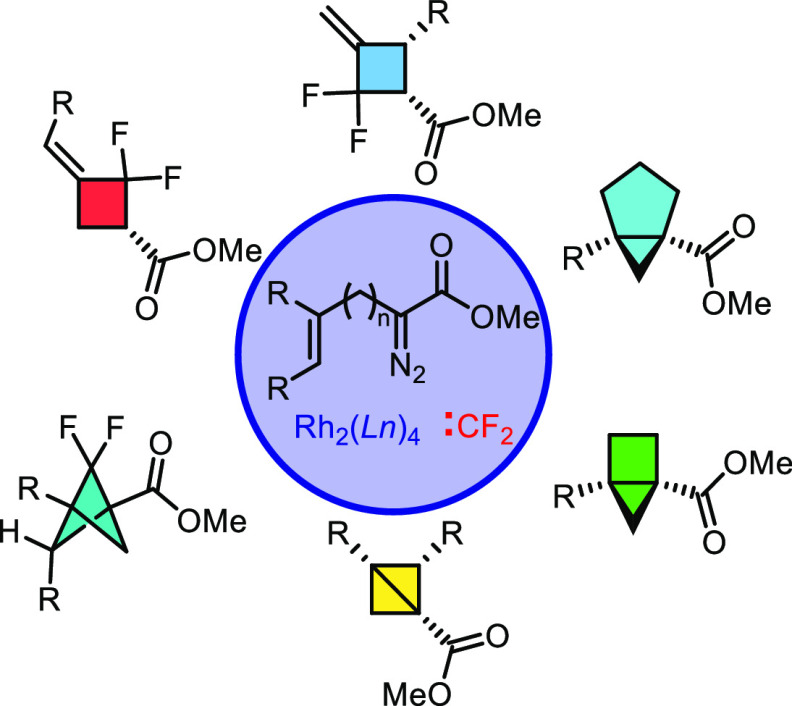

Rapid access to 2,2-difluorobicylco[1.1.1]pentanes is enabled from an α-allyldiazoacetate precursor in a one-pot process through cyclopropanation to afford a 3-aryl bicyclo[1.1.0]butane, followed by reaction with difluorocarbene in the same reaction flask. The modular synthesis of these diazo compounds affords novel 2,2-difluorobicyclo[1.1.1]pentanes that were inaccessible through previously reported methods. The reactions of chiral 2-arylbicyclo[1.1.0]butanes in the same manner generate altogether different products with high asymmetric induction, methylene-difluorocyclobutanes. Larger ring systems including bicyclo[3.1.0]hexanes are also rapidly furnished due to the modular nature of the diazo starting material.

2,2-Difluorobicylco[1.1.1]pentanes have generated considerable interest in recent years due to their role as phenyl bioisosteres.^1^ Inclusion of these groups in the place of phenyl rings can serve to eliminate metabolic liabilities in drug molecules and on occasion can even provide substances with greater potency than the corresponding aryl analogues.^1a,2^ Several effective methods have recently emerged for generating 2-substituted bicyclo[1.1.1]pentanes.^1d,3^ A particularly innovative method is the difluorocarbene-induced ring-expansion of 3-arylbicylco[1.1.0]butanes **2** to form difluorobicyclo[1.1.1]pentanes **3** (Scheme 1a).^1a,1b,3h^ While this route is practical for the exploration of several substrates, the scope of the transformation is limited due to the synthetic accessibility of bicyclo[1.1.0]butane precursors.^1a,1b^ Introduction of additional functionality at the other methylene positions of the difluorobicyclo[1.1.1]pentane using this ring expansion methodology has so far been limited to deuterium.^3h^ We envisioned that a wider array of derivatives would be accessible by combining intramolecular cyclopropanation of diazo compounds **4** with difluorocarbene ring expansion (Scheme 1b). These efforts resulted in the formation of the desired bicyclo[1.1.0]butanes **5** and difluorobicyclo[1.1.1]pentanes **6**. Additionally, an alternative difluorocarbene reaction was discovered to form difluorocyclobutanes **7** and **8**. The intramolecular cyclopropanation could also be applied to furnish extended strained ring systems, including bicyclo[1.1.0]pentanes **9** and bicyclo[1.1.0]hexanes **10**.

**Scheme 1:**
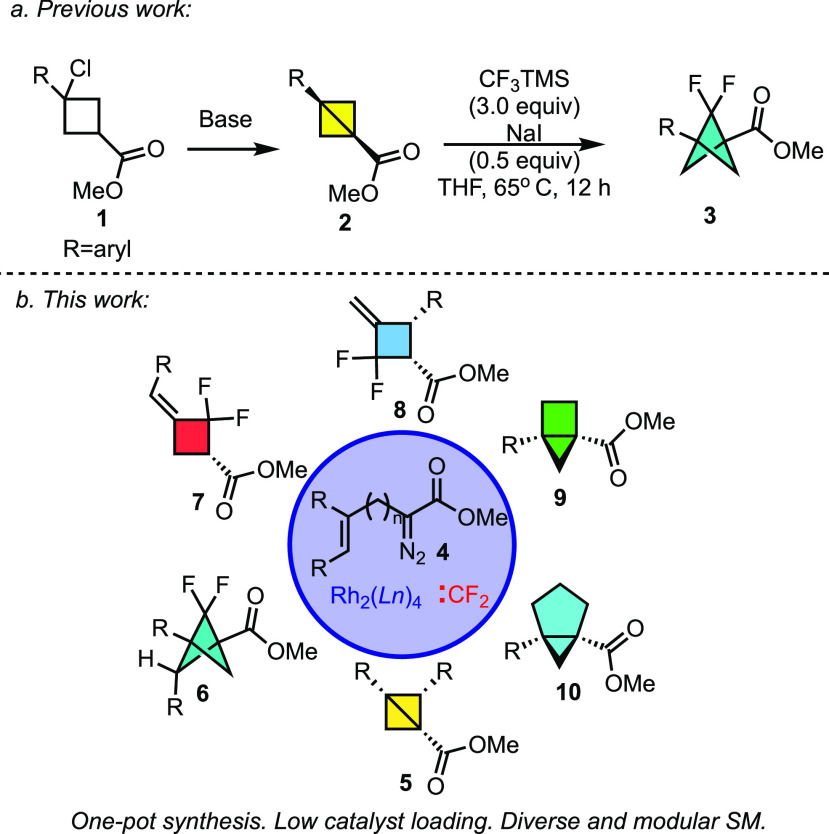
Modular Diazo Starting Material Provides Rapid Access to a Diverse Series of Molecules

The impetus for this study was a method we had previously reported for the asymmetric synthesis of 2-arylbicyclo[1.1.0]butanes **12** through the intramolecular cyclopropanation of α-allyldiazoacetates **11** in the presence of a chiral dirhodium tetracarboxylate catalyst (Scheme 2a).^4^ This method generated a series of 2-arylbicyclo[1.1.0]butanes with high yields and enantioselectivity. Soon thereafter Fox independently reported the synthesis of highly functionalized cyclobutanes **14** from α-allyldiazoacetates **13** in a one-pot process, highlighting the potential to telescope this reaction with further derivatization (Scheme 2b).^5^ The previous intramolecular cyclopropanation had been limited to the formation of 2-arylbicyclo[1.1.0]butanes,^4,5^ but we envisioned that this reaction could be extended to a wider range of bicyclo[1.1.0]butanes by using the appropriate α-allyldiazoacetates **15** (Scheme 2c). Given the mild conditions of the intramolecular cyclopropanation, we suspected that the intramolecular cyclopropanation could be telescoped to include a reaction with difluorocarbene, thereby generating a variety difluorobicyclo[1.1.1]pentanes **16** in a one-pot process without the need to isolate the intermediate bicyclo[1.1.0]butanes.^5^ The development of this approach and the discovery of some unexpected transformations are described herein.

**Scheme 2:**
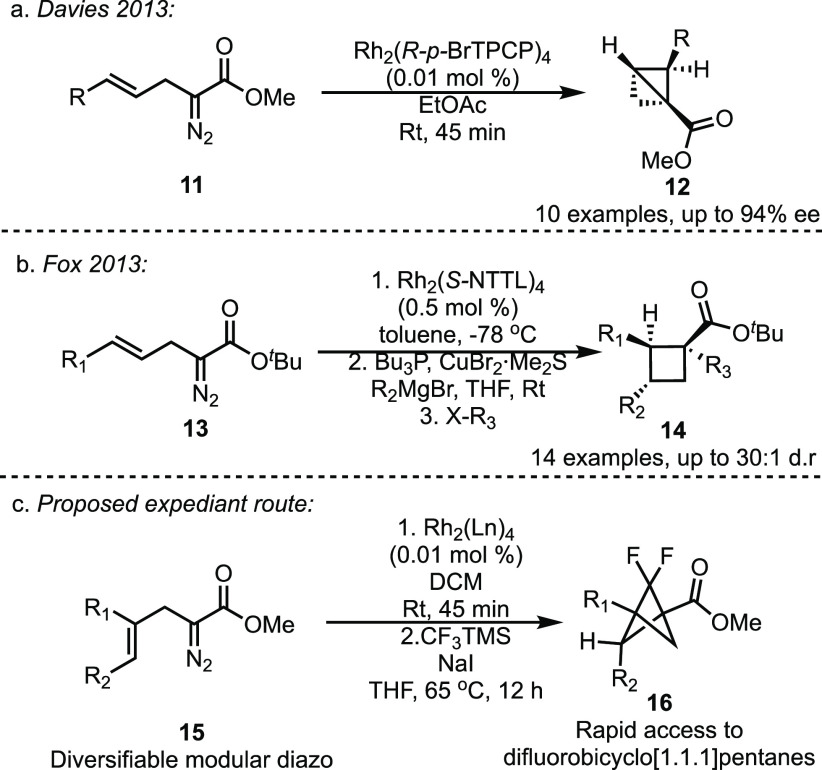
One-Pot Synthesis of Diverse Carbocycles

The first stage of the project examined whether the intramolecular cyclopropanation could offer a simple entry to 3-arylbicyclo[1.1.0]butanes, the substrates that had been previously used for the synthesis of difluorobicyclo[1.1.1]pentanes.^1a,1b^ A series of 3-substituted α-allyldiazoacetates **17** were prepared via a two-step process from readily accessible allyl-bromo-α-methylstyrene derivatives (see Supporting Information for details). The bromide was first displaced with methyl acetoacetate, followed by a deacylative diazo transfer to afford a diverse series of diazo compounds, **17a**–**h**. These precursors were then reacted in the presence of a dirhodium catalyst to afford 3-arylbicyclo[1.1.0]butanes **18** in high yield (Table 1). It is noteworthy that compound **18d** includes an *ortho*-substituted arene and compounds **18g** and **18h** feature thiophene heterocycles. None of these compounds have been prepared using the previously reported routes to access 3-arylbicyclo[1.1.0]butanes.^1a^ Electron-rich arenes were not compatible with this method, as can be seen with compound **17f**. The problem here appears to be the instability of the product which degrades rapidly upon forming. These substrates were shown to be incompatible in previous reports.^1a,1b^ Weakly electron-donating substituents are tolerated, however, and compounds **18b**, **18d**, and **18e**, are readily prepared.

**Table 1:**
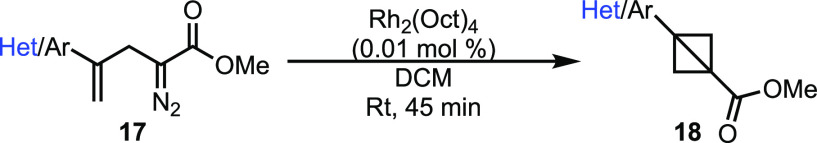

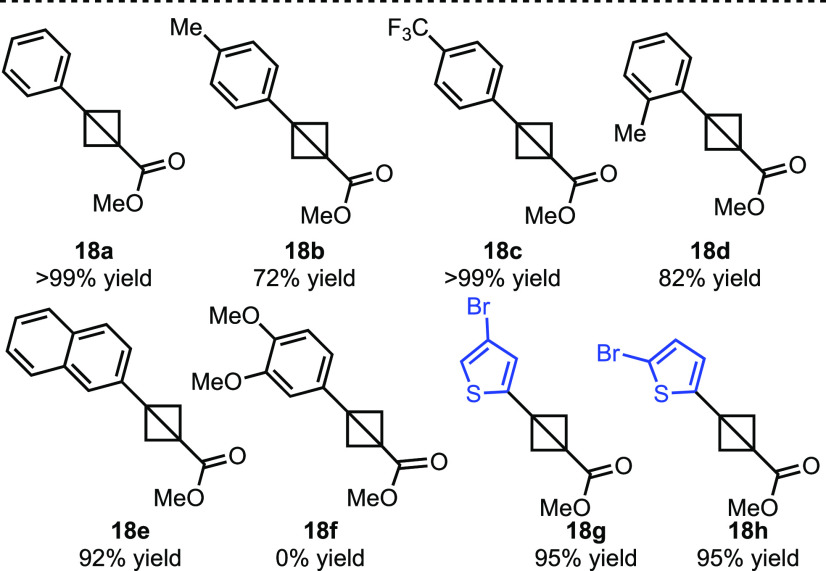
Synthesis of Bicyclo[1.1.0]butanes **18**

These 3-aryl substituted compounds were achiral, but if a group could be installed at the 2-position, a chiral trisubstituted difluorobicyclo[1.1.1]pentane could be generated.^1c,2b,4^ By using the more highly substituted diazoacetates **19**, 2-substituted 3-phenylbicyclo[1.1.0]butanes **20** were also prepared via this method (Table 2). Compounds **20a** and **20b** were generated in excellent yield; however, the formation of compound **20c** was less effective. To see if any chiral information could be generated in the synthesis of products **20a**–**c**, an exhaustive screen of chiral catalysts was performed (see Supporting Information for details). Unfortunately, the most effective catalyst, Rh_2_(*R-*NTTL)_4_, was able to afford the product with only moderate levels of enantioselectivity (up to 50% ee) regardless of the conditions used.^5^ As a result, the further reactions of **20a**–**c** were examined with racemic substrates generated by the intramolecular cyclopropanation with Rh_2_(Oct)_4_ as catalyst.

**Table 2:**
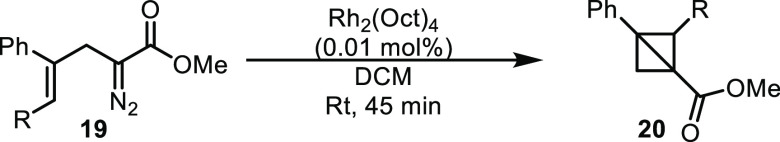

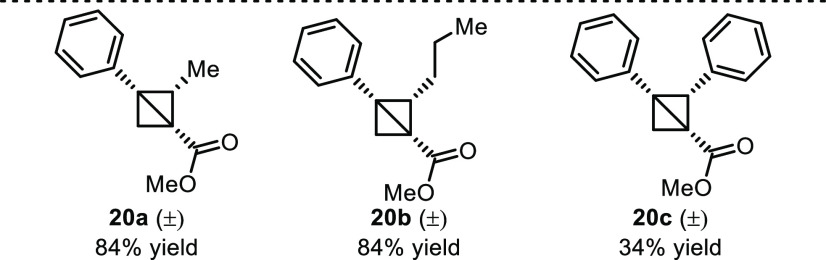
Synthesis of Trisubstituted Bicyclo[1.1.0]butanes **20**

With this method in hand, we wanted to evaluate the efficacy of telescoping the transformation to include difluorocarbene insertion to generate 2,2-difluorobicyclo[1.1.1]pentanes. Previous methods have required isolation of the bicyclo[1.1.0]butane by column chromatography before conducting difluorocarbene insertion, but we reasoned that the small amount of dirhodium catalyst (0.01 mol %) and the lack of unreacted starting material obviated the need for isolation of the product. A solvent exchange was needed, however, as the intramolecular cyclopropanation is less competent in THF as a solvent, and difluorocarbene insertion requires THF to achieve optimal results. The results of the reaction of the diazo compounds **17** and **19**, first with Rh_2_(Oct)_4_ followed by a solvent switch and treatment with NaI and CF_3_TMS, are reported in Table 3.^1a,1b^ 3-Aryl-bicyclo[1.1.0]butanes were generally effective in the ring-expansion with difluorocarbene, and several 3-aryl-2,2-difluorobicyclo[1.1.1]pentanes **21** could be synthesized by this method, including the novel derivatives, **21d**, **21e**, and **21f**.^1a,1b^ Unfortunately, the 2,3-disubstituted derivatives **19a**–**c** were less effective in these sequential reactions. **20a** and **20b** afforded the desired 2,2-difluorobicyclo[1.1.1]pentane products **22a** and **22b** but in low yield, whereas **20c** gave none of the desired product. Interestingly, **22a** and **22b** were generated as single diastereomers which suggests that the addition of difluorocarbene to the bicyclo[1.1.0]butane is controlled by the orientation of the substituents set during the intramolecular cyclopropanation.^1d,3h,6−8^

**Table 3:**
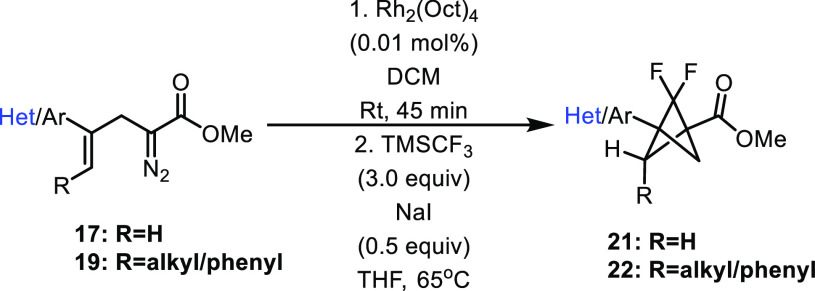

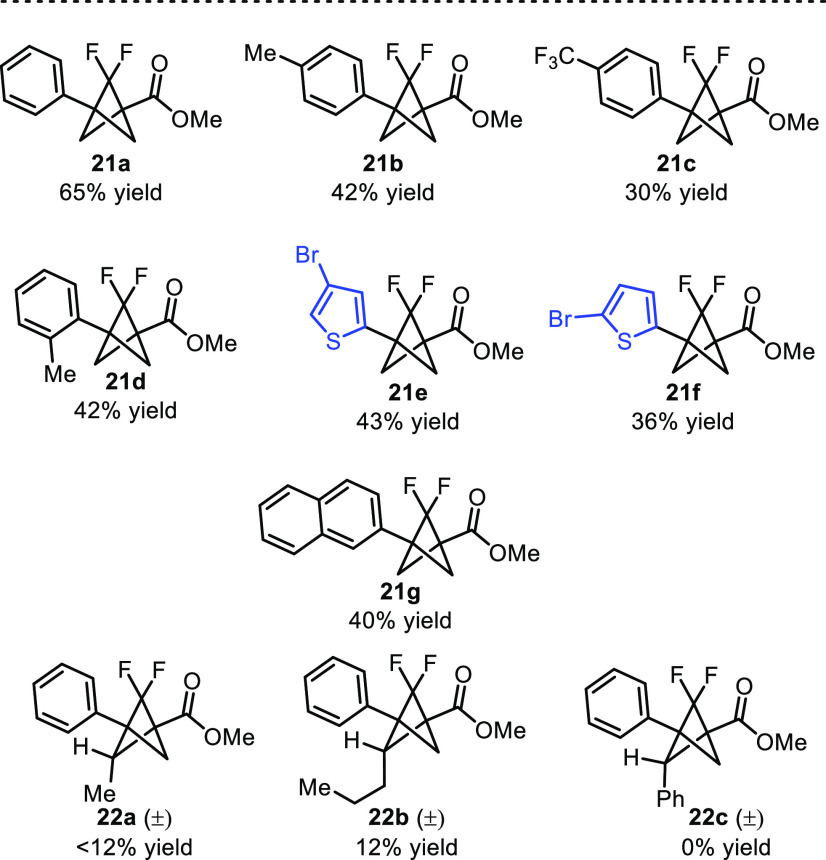
One-Pot Synthesis of 2,2-Difluorobicyclo[1.1.1]pentanes

We were curious to see if the chiral 2-arylbicyclo[1.1.0]butanes we had previously reported^5^ would similarly generate difluorobicyclo[1.1.1]pentanes, analogous to the 3-aryl-substituted system. To evaluate this possibility, α-allyl diazoacetates were prepared by the literature method^4^ and subjected to the one-pot difluorocarbene reaction optimized for difluorobicyclo[1.1.1]pentane generation (Scheme 3). In the first step the diazo compound **23** was reacted in the presence of Rh_2_(*S-p-*BrTPCP)_4_ (0.01 mol %) as catalyst^9^ and ethyl acetate (EtOAc) as solvent, the combination which was shown to afford the highest asymmetric induction in our previous study (Scheme 4).^4^ Again conversion to the desired bicyclo[1.1.0]butane product **24** was monitored by FTIR, and the reaction was complete after 45 min.^4^ The crude reaction mixture was concentrated *in vacuo* to remove the solvent, then dissolved in THF, and reacted with CF_3_TMS (3 equiv) at 65 °C in the presence of NaI (0.5 equiv) overnight. Upon completion of the reaction, the products were isolated and analyzed by ^1^H and ^19^F NMR. We observed that no difluorobicyclo[1.1.1]pentane **25** was generated. Instead, the reaction yielded a 2:1 mixture of methylene difluorocyclobutenes **26** and **27** (2:1 ratio).^10^ Furthermore, **26** and **27** were obtained with high enantioselectivity: compound **26** was isolated in 75% ee, and **27** was isolated in 91% ee. This type of reaction occurs with all 2-arylbicyclo[1.1.0]butanes we examined, but the products are generally quite unstable and could not be isolated in pure form.

**Scheme 3:**
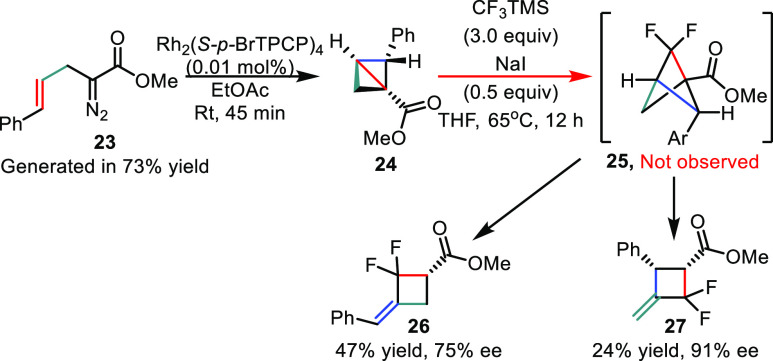
Formation of Methylene-Difluorocyclobutenes **26** and **27**

**Scheme 4:**
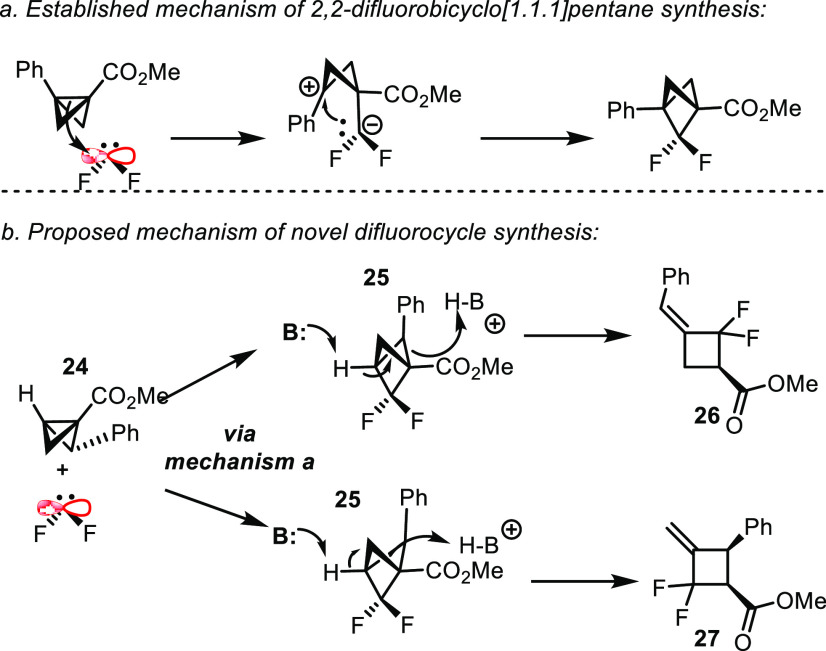
Proposed Mechanism of Difluorocarbene Reaction with Bicyclo[1.1.0]butanes

A reasonable mechanism to explain the role of substituents on the product outcome from the reaction of bicyclo[1.1.0]butane **24** with difluorocarbene is shown in Scheme 4.^11^ Previously, it has been proposed that the insertion of difluorocarbene is a stepwise process beginning with attack of the difluorocarbene on the central C1–C3 bond of the bicyclo[1.1.0]butane, followed by recombination of the resultant zwitterion to form a 2,2-difluorobicyclo[1.1.1]pentane.^1a,1b,3h,12^ In the case of 3-substituted bicyclo[1.1.1]pentanes **21a**–**g** and **22a**–**b**, the products are stable and isolable,^1a,1b^ and this was confirmed in our studies (Table 3). In contrast, for the 3-unsubstituted bicyclo[1.1.1]pentanes, we propose that the difluorobicyclo[1.1.1]pentane **25** is formed as expected, but they are unstable and rearrange under the reaction conditions. The methine C3 proton of **25** is acidic due to its proximity to the difluoromethylene group and the geometry of the bicyclo[1.1.1]pentane moiety. It is well-known that deprotonation and radical reactivity of the bicyclo[1.1.1]pentane methine proceeds easily under mild conditions, and it appears that the catalytic iodide present in the reaction mixture is sufficiently basic to catalyze this process.^3f,13^ The resultant anion then forms an alkene with either of the adjacent carbons, and the C1–C2/C4 bond is protonated by the resultant conjugate acid. This opens the caged compound resulting in the formation of isomeric difluorocyclobutenes **26** and **27** (Scheme 4). Attempts to trap out difluorobicyclo[1.1.1]pentane intermediate **25** by skipping workup or running the reaction for less time invariably failed, suggesting that the decomposition of this intermediate is fast. The weakest C–C bond (C1–C2) is the most easily cleaved during alkene formation, which explains the 2:1 product distribution in favor of **26** (Scheme 3). Interestingly only one alkene isomer of **26** was obtained in the reaction which indicates that the addition of difluorocarbene to the bicyclo[1.1.0]butane intermediate was diastereoselective as was observed in the trisubstituted bicyclo[1.1.0]butanes **22a** and **22b**.^3h^

The modularity of the diazo compound starting material has broader potential. Extending the alkene-diazo linker should allow facile access to expanded ring systems including bicyclo[2.1.0]pentanes and bicyclo[3.1.0]hexanes which have thus far been unexplored as substrates for the difluorocarbene reaction but could generate novel interesting caged architectures, difluorobicyclo[2.1.1]hexane and difluorobicyclo[3.1.1]heptane.^11,14^ Similar caged compounds have recently garnered significant interest as *meta*- and *ortho-*substituted phenyl-bioisosteres for medicinal applications.^14f,14g,15^ To this effect, diazo compounds were synthesized, and the intramolecular cyclopropanation was performed to generate the novel bicyclo[2.1.0]pentane **28** and bicyclo[3.1.0]hexane **29**. Both products were afforded in excellent yield, highlighting the robustness of this chemistry despite the modular design of the diazo compound precursors (Scheme 5). Interestingly bicylco[3.1.0]hexane **29** was afforded in high enantioselectivity (65% ee) through the use of the chiral catalyst Rh_2_(*S-p*-BrTPCP)_4_ and ethyl acetate as solvent (Scheme 3). Surprisingly, neither of these expanded ring systems were able to successfully react with difluorocarbene. Bicyclo[2.1.0]pentane **28** did not generate the desired 2,2-difluorobicyclo[2.1.1]hexane and instead formed a complex mixture of products. 3-Phenyl bicyclo[3.1.0]hexane **29** was also unreactive under the standard conditions. It is likely that **29** does not contain sufficient ring strain across the C1–C3 bond to engage with difluorocarbene. More forcing conditions (higher temperature, longer reaction times, alternative difluorocarbene sources) also did not afford difluorocarbene insertion product in either case.^3h^

**Scheme 5:**
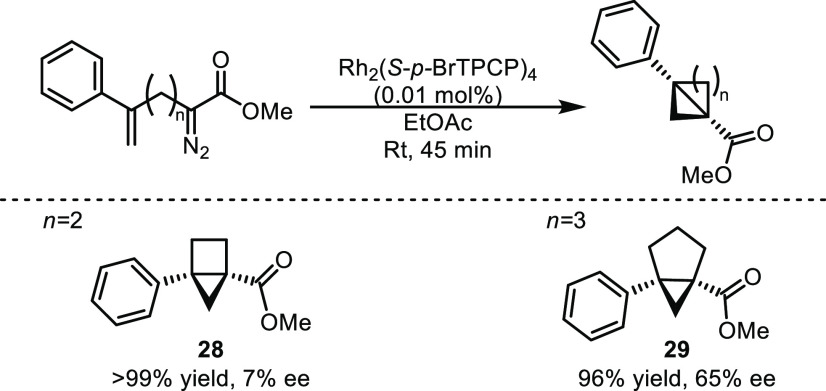
Expanded Ring Synthesis

In this work, we have developed a novel approach to synthesizing 3-arylbicyclo[1.1.0]butanes and more substituted analogues via a modular α-allyldiazoacetate precursor. The novel method for synthesizing 3-arylbicyclo[1.1.0]butanes is more tolerant of various functionality and generally proceeds with very high yield at low catalyst loading (0.01 mol %), enabling the synthesis of bicyclo[1.1.0]butanes that were unsuccessful by previously reported methods. It was also possible to generate a series of difluorobicyclo[1.1.1]pentanes from the α-allyl diazoacetate in a one-pot process with yields comparable to those of previous reports. We were also able to explore difluorocarbene insertion of 2-arylbicyclo[1.1.0]butanes and determine the structure of the unusual products generated as well as propose a mechanism for their formation, although the compounds proved unstable. Future work will be conducted to determine the scope of transformations accessible to these highly substituted bicyclo[1.1.0]butanes and improve the asymmetric induction. Additionally, diversification of the methylenedifluorocyclobutene products will be explored to afford stable and isolable highly substituted difluorocyclobutane products with high asymmetric induction. Finally, the novel bicyclo[3.1.0]hexane and bicyclo[2.1.0]pentane products will be diversified. Our hope is that new methods for the generation of difluorocarbene may allow these products to be transformed into the complex caged compounds initially targeted in this work.

## Data Availability

The data underlying this study are available in the published article and its online Supporting Information.

## Supporting Information Available

Complete experimental procedures and compound characterization (PDF)
