# Supplementary material for: One-Pot Synthesis of Difluorobicyclo[1.1.1]pentanes from α-Allyldiazoacetates
Source: Org Lett. 2023 Jul 10;25(28):5214–9. doi: 10.1021/acs.orglett.3c01664 (PMC10367075; doi:10.1021/acs.orglett.3c01664)
Supplement: Supplementary file 1 — ol3c01664_si_001.pdf [file ol3c01664_si_001.pdf]

One-Pot Synthesis of Difluorobicyclo[1.1.1]pentanes From α-Allyldiazoacetates.

Jack C. Sharland and Huw M. L. Davies*
Department of Chemistry, Emory University, 1515 Dickey Drive, Atlanta GA, 30322, United States

---

Supporting Information:

Table of Contents

1. *General Considerations……………………………………………………………………………………………S1*
2. *Preparation of Starting Materials…………………………………………………………………S2-S3*
3. *One-pot synthesis of difluorinated carbocycles ……………………………………………………..S3-S4*
4. *Known compounds used as precursors for scope elaboration…………………………………S4*
5. *Characterization of novel precursor compounds………………………………………………S5-S8*
6. *Characterization of known reaction intermediates and scope products……………..S8-S10*
7. *Characterization of Novel Compounds…………………………………………………………S10-S16*
8. *NMR Spectra………………………………………………………………………………………………S17-S59*
9. *HPLC/SFC data…………………………………………………………………………………………..S60-S65*
10. *References…………………………………………………………………………………………………... S66*

CAUTION: Diazo compounds are high energy compounds and need to be treated with respect. Even though we experienced no energetic decomposition in this work, care should be taken in handling large quantities of diazo compounds. Large scale reactions should be conducted behind a blast shield. For a more complete analysis of the risks associated with diazo compounds see the recent review by Bull *et. al.*[1]

1. General Considerations

All experiments were carried out in oven-dried glassware under argon atmosphere unless otherwise stated. Flash column chromatography was performed on silica gel. Unless otherwise noted, all other reagents were obtained from commercial sources (Sigma Aldrich, Fisher, TCI Chemicals, AK Scientific, Combi Blocks, Oakwood Chemicals, Ambeed) and used as received without purification. ^{1}H, ^{13C}, and ^{19}F NMR spectra were recorded at either 400 MHz (^{13C} at 100 MHz) on Bruker 400 spectrometer or 600 MHz (^{13C} at 151 MHz) on INOVA 600 or Bruker 600 spectrometer. NMR spectra were run in solutions of deuterated chloroform ($CDCl_3$) with residual chloroform taken as an internal standard (7.26 ppm for ^{1}H, and 77.16 ppm for ^{13C}), and were reported in parts per million (ppm). The abbreviations for multiplicity are as follows: s = singlet, d = doublet, t = triplet, q = quartet, p = pentet, m = multiplet, dd = doublet of doublet, etc. Coupling constants (J values) are obtained from the spectra. Thin layer chromatography was performed on aluminum-back silica gel plates with UV light and cerium aluminum molybdate (CAM) or permanganate ($KMnO_4$) stain to visualize. Mass spectra were taken on a Thermo Finnigan LTQ-FTMS spectrometer with APCI, ESI or NSI. IR spectra were collected on a Nicolet iS10 FT-IR spectrometer from Thermo Scientific and reported in unit of cm^{-1}. Enantiomeric excess (% ee) data were obtained on a Varian Prostar chiral HPLC instrument, an Agilent 1100 HPLC, or a Waters SFC, eluting the purified products using a mixed solution of HPLC-grade 2-propanol (*i*-PrOH) and *n*-hexane for HPLC, and a mixed solution of supercritical CO_2 and acetonitrile+0.2% formic acid (MeCN+0.2%FA).

2. Preparation of starting materials

General Method A: Synthesis of disubstituted α, β, and γ allyl diazoacetates:

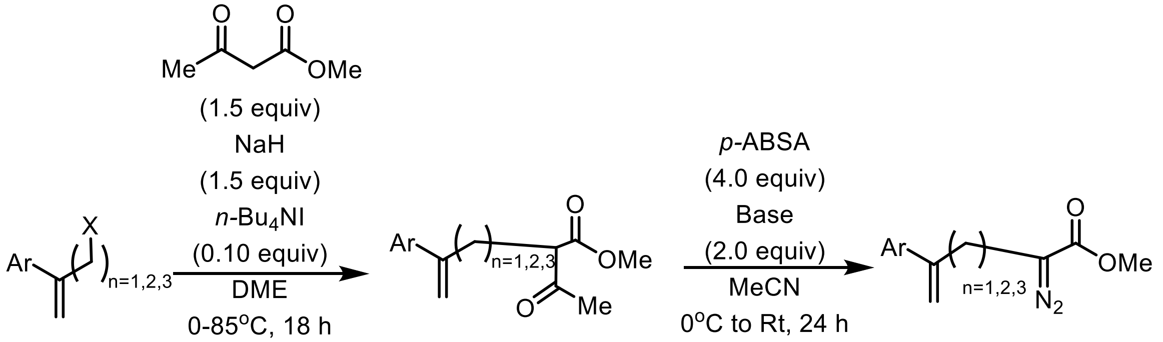

First, the acetoacetate precursors were synthesized. Under an argon atmosphere, a solution of methyl 3-oxobutanoate (1.5 equiv) in anhydrous dimethoxyethane (DME) was added dropwise to a stirred suspension of NaH (60% suspension on mineral oil, 1.5 equiv) in anhydrous DME at 0 °C in 1 h. Then, *n*-Bu_4NI (0.1 equiv) was added in one portion, followed by dropwise addition of (3-bromoprop-1-en-2-yl)arene, (3-bromobut-1-en-2-yl)arene, or (3-chloropent-1-en-2-yl)arene (1.0 equiv) in anhydrous DME at 0 °C over 1 h via addition funnel to generate a 1.5 M solution (relative to halide starting material). The resulting mixture was then heated to 85 °C in an aluminum block and stirred overnight. After reaction completion, the mixture was cooled to 0 °C, diluted slowly with 1 N HCl, and extracted with diethyl ether (Et_2O). The combined organic extracts were washed by brine, dried over anhydrous Na_2SO_4 and concentrated in vacuo. After a short flash chromatographic purification (0-10% Et_2O /hexanes), aggregation of product containing fractions and removal of solvent *in vacuo,* the resulting crude product was used directly without full characterization or further purification in the next step.

Under an argon atmosphere, in a flame-dried RBF, crude methyl 2-acetyl-4-arylpent-4-enoate, methyl 2-acetyl-5-arylhex-5-enoate, or methyl 2-acetyl-6-aryllhept-6-enoate (1.0 equiv) and *p*-acetamidobenzenesulfonyl azide (*p*-ABSA) (2.0 equiv) was dissolved in MeCN, to generate a 0.2 M solution of precursor, and the reaction mixture was cooled to 0 °C. DBU (4.0 equiv) was added dropwise at 0 °C. The reaction mixture was then warmed to room temperature and stirred overnight to afford a dark red solution. The crude reaction mixture was then extracted with Et_2O, washed by brine, dried over anhydrous Na_2SO_4, and concentrated in vacuo. Sample was chromatographed 0-3% Et_2O /hexanes and product containing fractions were aggregated. Solvent was removed *in vacuo* to afford the desired product as a highly colored oil. The products are stable if stored at -20 °C for at least 4 months.

General Method B: General method for the synthesis of trisubstituted allyl-diazoacetates:

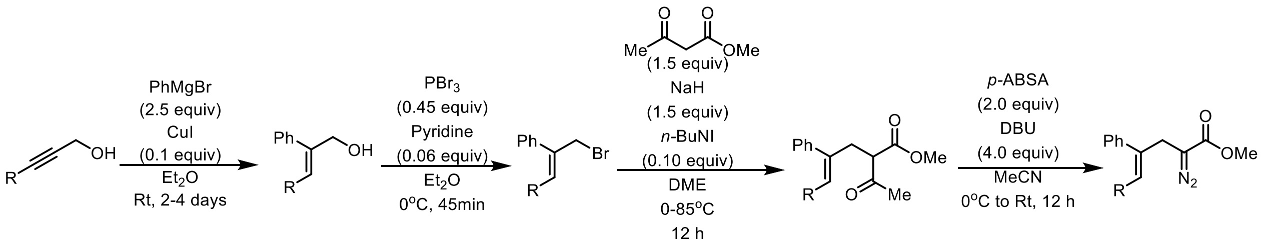

B-1: Copper-mediated addition of phenylmagnesium bromide to propargyl alcohols:

PhMgBr solution (3.0 M in Et_2O) was added dropwise to a suspension of Cu(I)I (0.10 equiv, 10 mol %) and propargyl alcohol in dry Et_2O (125 mL) at 0 °C in a flame dried RBF to afford a 1 M solution of propargyl alcohol. The reaction mixture was then warmed to room temperature and stirred for 2-4 days. The resultant mixture was then cooled to 0 °C, the reaction mixture was carefully quenched by dropwise addition of saturated aqueous NH_4Cl to yield a blueish-gray suspension. The reaction mixture was brought to room temperature and extracted with Et_2O, washed with brine, dried over Na_2SO_4 and

concentrated *in vacuo.* The obtained compound was purified by column chromatography (0-30% Et_2O/hexanes) to give the desired product. Spectra of the purified products obtained via this method, (*E*)-2-phenylbut-2-en-1-ol (3.41 g, 23 mmol, 65% yield), (*E*)-2-phenylhex-2-en-1-ol (2.46 g, 27 mmol, 28% yield), and (*E*)-2,3-diphenylprop-2-en-1-ol (7.53 g, 35.8 mmol, 95% yield), matched those reported in the literature.[2, 3]

B-2: Bromination of trisubstituted allyl-alcohols:

In a flame-dried RBF under an inert argon atmosphere, alkene (1 equiv) was dissolved in enough dry Et_2O to afford a 0.3 M solution. To this solution was added pyridine (0.06 equiv) and the reaction mixture was cooled to 0 °C. Then PBr_3 (0.45 equiv) was added dropwise. The reaction was warmed to room temperature with additional stirring for 45 min or until disappearance of starting material by TLC. After completion of reaction, the mixture was quenched by addition of ice cubes to yield a cloudy suspension. The aqueous and organic layers were separated, and the organic layer was dried over Na_2SO_4. Solvent was removed *in vacuo* and crude product was purified via silica plug (10% Et_2O /hexanes as eluent). After removal of solvent *in vacuo* the product was obtained, usually as a yellow oil. Spectra of the purified products obtained via this method, (*E*)-(1-bromobut-2-en-2-yl)benzene (4.1 g, 19 mmol, 84% yield), (*E*)-(1-bromohex-2-en-2-yl)benzene (920 mg, 3.85 mmol, 68% yield), and (*E*)-(3-bromoprop-1-ene-1,2-diyl)dibenzene (3.52 g, 12.9 mmol, 54% yield) matched those reported in the literature.[2, 3]

B-3: Synthesis of Synthesis of diazo compounds **19a-c**:

First, the acetoacetate precursors were synthesized. Under an argon atmosphere, a solution of methyl 3-oxobutanoate (1.5 equiv) in anhydrous dimethoxyethane (DME) was added dropwise to a stirred suspension of NaH (60% suspension on mineral oil, 1.5 equiv) in anhydrous DME at 0 °C in 1 h. Then, *n*-Bu_4NI (0.1 equiv) was added in one portion, followed by dropwise addition of trisubstituted allyl-bromide (1.0 equiv) in anhydrous DME at 0 °C over 1 h via addition funnel to generate a 1.5 M solution (relative to bromide starting material). The resulting mixture was then heated to 85 °C in an aluminum block and stirred overnight. After reaction completion, the mixture was cooled to 0 °C, diluted slowly with 1 N HCl, and extracted with ethyl acetate (EtOAc). The combined organic extracts were washed by brine, dried over anhydrous Na_2SO_4 and concentrated in vacuo. After a short flash chromatographic purification (0-10% EtOAc /hexanes), aggregation of product containing fractions and removal of solvent *in vacuo,* the resulting crude (*Z)*-trisubstituted α-allyl-acetoacetate was used directly in the next step. Nomenclature dictates the reassignment of the alkene geometry as "*Z*" after this synthetic step but the stereochemistry of the alkene is preserved throughout the synthesis.

Under an argon atmosphere, in a flame-dried RBF, crude *Z*-alkene (1.0 equiv) and *p*-acetamidobenzenesulfonyl azide (*p*-ABSA) (2.0 equiv) was dissolved in MeCN, to generate a 0.2 M solution of precursor, and the reaction mixture was cooled to 0 °C. DBU (4.0 equiv) was added dropwise at 0 °C. The reaction mixture was then warmed to room temperature and stirred overnight to afford a dark red solution. The crude reaction mixture was then extracted with EtOAc, washed by brine, dried over anhydrous Na_2SO_4, and concentrated in vacuo. Sample was chromatographed (0-3% or 10% diethyl ether/hexanes for alkyl or aryl products respectively) and product containing fractions were aggregated. Solvent was removed *in vacuo* to afford the diazo as a bright yellow-orange oil. The products are indefinitely stable if refrigerated except for methyl (Z)-2-diazo-4-phenyloct-4-enoate which decomposed after 4 months at 0 °C.

3. One-pot synthesis of difluorinated carbocycles and other products

General Method C: One-pot synthesis of difluorobicylco[1.1.1]pentanes **21a-g** and **22a-c**:

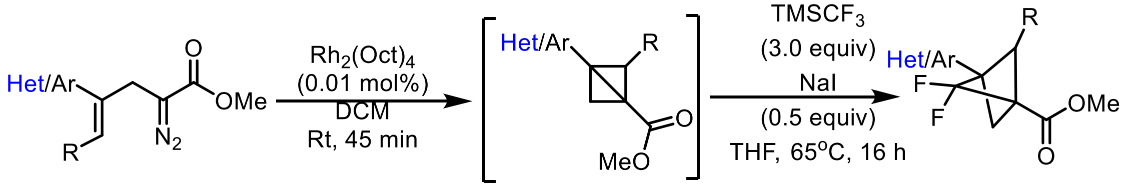

To a 16 mL flame-dried vial, kept under a dry atmosphere of argon, was added dry DCM (1.0 mL) and $Rh_2(Oct)_4$ (16 uL, c = 1.00 mg/ mL in DCM, 0.0001 equiv). Diazo compound (0.2 mmol, 1.0 equiv), dissolved in dry DCM (1 mL), was then added to the former solution drop-wise over 30 mins at room temperature via syringe pump. The 0.1 M reaction mixture was allowed to stir for another 15 min after the addition; when the diazo compound was fully consumed by IR analysis (disappearance of diazo ($C=N_2$) stretch at ~2100 cm^{-1}), the reaction mixture was concentrated in vacuo and analyzed by ^1H NMR in $CDCl_3$ over K_2CO_3 to confirm the presence of bicyclo[1.1.0]butane product. Once bicyclo[1.1.0]butane presence was confirmed the solution was evaporated to dryness *in vacuo*. The mixture was then dissolved in THF (2 mL, 0.1 M). $TMSCF_3$ (3 equiv) and NaI (0.5 equiv) were added to the solution. The resulting mixture was stirred at 65 °C overnight in an aluminum heating block. After completion, the reaction mixture was concentrated under reduced pressure, then the residue was dissolved in EtOAc, washed with DI water and brine, and dried over anhydrous Na_2SO_4. The solvent was removed *in vacuo* and the crude product was purified by column chromatography (gradient, 0-10% Et_2O /hexanes) and product containing fractions were aggregated. The solvent was removed *in vacuo* to afford the desired product in up to 65% yield.

General Method D: One-pot synthesis of chiral methylene difluorocyclobutenes **26** and **27**:

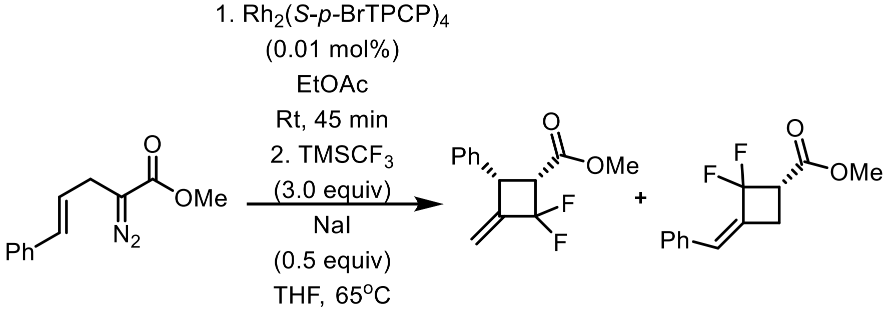

To a 16 mL flame-dried vial, kept under a dry atmosphere of argon, was added dry EtOAc (1.0 mL) and Rh_2(*S*-*p*-BrTPCP)$_4$ (16 µL, c = 1.00 mg/ mL in EtOAc, 0.0001 equiv). Methyl (E)-2-diazo-5-phenylpent-4-enoate (0.2 mmol, 1.0 equiv), dissolved in dry EtOAc (1 mL), was then added to the former solution drop-wise over 30 mins at room temperature via syringe pump. The 0.1 M reaction mixture was allowed to stir for another 15 min after the addition; when the diazo compound was fully consumed by IR analysis (disappearance of diazo ($C=N_2$) stretch at ~2100 cm^{-1}), the reaction mixture was concentrated in vacuo and analyzed by ^1H NMR in $CDCl_3$ over K_2CO_3 to confirm the presence of bicyclo[1.1.0]butane product. Once bicyclo[1.1.0]butane presence was confirmed as the spectra matched those reported in the literature, the solution was evaporated to dryness *in vacuo*. The mixture was then dissolved in THF (2 mL, 0.1 M). $TMSCF_3$ (3 equiv) and NaI (0.5 equiv) were added to the solution. The resulting mixture was stirred at 65 °C overnight in an aluminum heating block. After completion, the reaction mixture was concentrated under reduced pressure, then the residue was dissolved in EtOAc, washed with DI water and brine, and dried over anhydrous Na_2SO_4. The solvent was removed *in vacuo* and the crude product was purified by column chromatography (gradient, 0-2% Et_2O /hexanes) and product containing fractions were aggregated. The solvent was removed *in vacuo* to afford the desired products in up to 74% yield as a 2:1 mixture of methylene difluorocyclobutenes. The products appear as a single peak, to separate them one must collect low volume fractions (3-5 mL) and NMR each vial individually. Additionally, the products are unstable upon isolation and lose difluoromethane to generate a diene.

4. Known compounds used as precursors for scope elaboration:

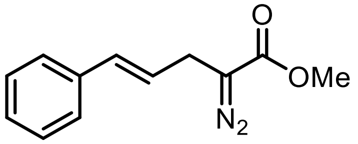

Methyl (E)-2-diazo-5-phenylpent-4-enoate **23** was synthesized according to known methods and spectra matched the literature reported spectra.[4]

5. Characterization of novel precursor compounds:

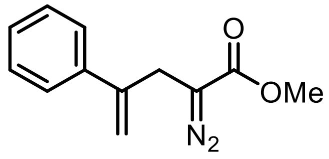

Methyl 2-diazo-4-phenylpent-4-enoate: Product **17a** is obtained from methyl 2-acetyl-4-phenylpent-4-enoate (5.23 g, 22.5 mmol) via method **A** and was purified by flash column chromatography (0-3% Et_2O /hexanes) to afford the product as a bright yellow liquid in 67% yield (3.28 g, 15.2 mmol).

^{1}H NMR: (600 MHz, $CDCl_3$) δ 7.46 (d, *J* = 6.8 Hz, 2H), 7.39 – 7.35 (m, 2H), 7.34 – 7.30 (m, 1H), 5.51 (s, 1H), 5.21 (d, *J* = 1.1 Hz, 1H), 3.78 (s, 3H), 3.55 (d, *J* = 1.3 Hz, 2H).

^{13}C NMR (151 MHz, $CDCl_3$) δ 142.9, 139.1, 128.6, 128.5, 128.1, 126.1, 126.0, 114.9, 52.0, 29.1.

FTIR(neat): 3015, 2970, 2950, 2079, 1738, 1683, 1435, 1365, 1348, 1228, 1216, 1203, 1114, 904, 779, 732, 699, 579, 528cm^{-1}

HRMS: (+pAPCI) m/z=M+1H calcd for: $C_{12}H_{13}N_2O_2$ 217.0972; Found: 217.0974

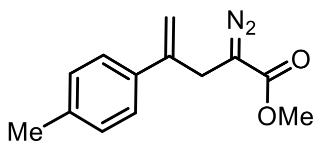

Methyl 2-diazo-4-(*p*-tolyl)pent-4-enoate: Product **17b** is obtained from methyl 2-acetyl-4-(*p*-tolyl)pent-4-enoate (5.00 g, 20.3 mmol) via method **A** and was purified by flash column chromatography (0-3% Et_2O /hexanes) to afford the product as a bright yellow liquid in 39% yield (1.83 g, 7.95 mmol).

^{1}H NMR (600 MHz, $CDCl_3$) δ 7.36 (d, J = 7.9 Hz, 2H), 7.17 (d, J = 7.9 Hz, 2H), 5.47 (s, 1H), 5.16 (d, J = 1.0 Hz, 1H), 3.78 (s, 3H), 3.53 (s, 2H), 2.37 (s, 3H).

^{13}C NMR (151 MHz, $CDCl_3$) δ 167.5, 142.6, 137.9, 136.1, 129.2, 125.9, 114.1, 52.0, 29.1, 21.1.

FTIR(neat): 2951, 2078, 1685, 1626, 1566, 1514, 1435, 1341, 1315, 1302, 1181, 1112, 1018, 975, 902, 825, 751, 734, 677, 641, 577, 535, 482 cm^{-1}

HRMS: (+pESI) m/z=M+1H calcd for $C_{13}H_{15}N_2O_2$ 231.1128; Found: 231.1127

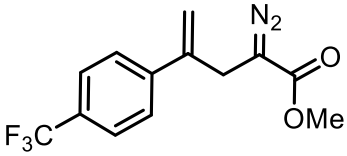

Methyl 2-diazo-4-(4-(trifluoromethyl)phenyl)pent-4-enoate: Product **17c** is obtained from methyl 2-acetyl-4-(4-trifluoromethylphenyl)pent-4-enoate (215 mg, 716 µmol) via method **A** and was purified by flash column chromatography (0-3% Et_2O /hexanes) to afford the product as a bright yellow liquid in 39% yield (80 mg, 0.28 mmol).

^{1}H NMR (600 MHz, $CDCl_3$) δ 7.62 (d, J = 8.3 Hz, 2H), 7.56 (d, J = 8.1 Hz, 2H), 5.57 (s, 1H), 5.31 (s, 1H), 3.78 (s, 3H), 3.56 (s, 2H).

^{13}C NMR (151 MHz, $CDCl_3$) δ 167.2, 142.6, 142.0, 130.1 (q, J = 32.5 Hz), 126.4, 125.5 (q, J = 3.8 Hz), 116.9, 52.1, 36.0, 29.1.

^{19}F NMR (565 MHz, $CDCl_3$) δ -62.62.

FTIR(neat): 2955, 2082, 1666, 1616, 1574, 1437, 1405, 1344, 1322, 1164, 1113, 1164, 1113, 1065, 1014, 976, 915, 847, 813, 751, 733, 721, 604, 536 cm^{-1}

HRMS: (+pAPCI) m/z=M+1H calcd for $C_{13}H_{12}N_2O_2F_3$ 285.0845; Found: 285.0850

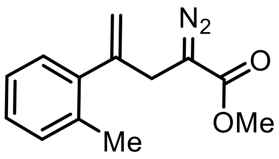

Methyl 2-diazo-4-(*o*-tolyl)pent-4-enoate: Product **17d** is obtained from methyl 2-acetyl-4-(*o*-tolyl)pent-4-enoate (303 mg, 1.231 mmol) via method **A** and was purified by flash column chromatography (0-3% Et_2O /hexanes) to afford the product as a bright yellow liquid in 69% yield (195 mg, 847 μmol).
^1H NMR (600 MHz, $CDCl_3$) δ 7.21 (dd, J = 4.0, 1.3 Hz, 2H), 7.19 – 7.14 (m, 1H), 7.12 (d, J = 7.6 Hz, 1H), 5.34 (d, J = 1.5 Hz, 1H), 5.07 (d, J = 1.2 Hz, 1H), 3.75 (s, 4H), 3.35 (s, 1H), 2.34 (s, 4H).
^{13}C NMR (151 MHz, $CDCl_3$) δ 167.4, 144.7, 141.0, 135.1, 130.3, 128.3, 127.5, 125.7, 116.0, 52.0, 31.6, 19.7.
FTIR(neat): 2952, 2078, 1688, 1487, 1435, 1339, 1304, 1188, 1107, 1043, 976, 910, 813, 769, 748, 730, 683, 593, 531, 500, 456 cm^{-1}
HRMS: (+pAPCI) m/z=M+1H calcd for $C_{13}H_{15}N_2O_2$ 231.1128; Found: 231.1131

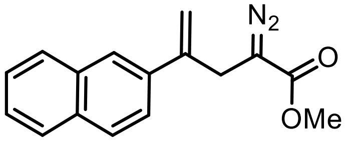

Methyl 2-diazo-4-(naphthalen-2-yl)pent-4-enoate: Product **17e** is obtained from methyl 2-acetyl-4-(naphthalen-2-yl)pent-4-enoate (631 mg, 2.23 mmol) via method **A** and was purified by flash column chromatography (0-3% Et_2O /hexanes) to afford the product as a bright yellow liquid in 62% yield (370 mg, 1.39 mmol).
^1H NMR (600 MHz, $CDCl_3$) δ 7.90 (d, J = 1.9 Hz, 1H), 7.87 – 7.74 (m, 3H), 7.63 (dd, J = 8.6, 1.9 Hz, 1H), 7.54 – 7.36 (m, 2H), 5.66 (s, 1H), 5.31 (s, 1H), 3.79 (s, 3H), 3.68 (s, 2H).
^{13}C NMR (151 MHz, $CDCl_3$) δ 167.4, 142.7, 136.2, 133.3, 133.1, 128.4, 128.1, 127.6, 126.3, 126.2, 125.02, 124.2, 115.4, 52.0, 29.2.
FTIR(neat): 3056, 2950, 2077, 1682, 1624, 1595, 1505, 1434, 1137, 1034, 1177, 1132, 1108, 1017, 975, 951, 893, 858, 818, 770, 747, 731, 669, 636, 572, 530, 473 cm^{-1}
HRMS: (+pAPCI) m/z=M+1H calcd for $C_{16}H_{15}N_2O_2$ 267.1128; Found: 267.1116

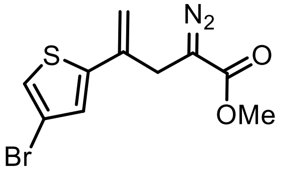

Methyl 4-(4-bromothiophen-2-yl)-2-diazopent-4-enoate: Product **17g** is obtained from methyl 2-acetyl-4-(4-bromothiophen-2-yl)pent-4-enoate (232 mg, 731 μmol) via method **A** and was purified by flash column chromatography (0-3% Et_2O /hexanes) to afford the product as a bright yellow oil in 24% yield (53 mg, 180 μmol).
^1H NMR (600 MHz, $CDCl_3$) δ 7.13 (d, *J* = 1.4 Hz, 1H), 7.02 (d, *J* = 1.4 Hz, 1H), 5.54 (s, 1H), 5.12 (s, 1H), 3.82 (s, 3H), 3.46 (s, 2H).
^{13}C NMR (151 MHz, $CDCl_3$) δ 167.2, 144.0, 135.8, 126.8, 122.2, 114.2, 110.1, 52.2, 29.2.
FTIR(neat): 3108, 2951, 2082, 1686, 1620, 1513, 1436, 1341, 1304, 1190, 1112, 1040, 975, 897, 864, 816, 734, 592, 535 cm^{-1}
HRMS: (+pESI) m/z=M+Na calcd for $C_{10}H_9N_2O_2SBrNa$ 322.9460; Found: 322.9460

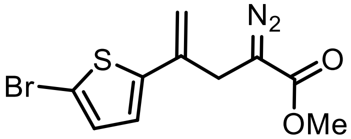

Methyl 4-(5-bromothiophen-2-yl)-2-diazopent-4-enoate: Product **17h** is obtained from methyl 2-acetyl-4-(5-bromothiophen-2-yl)pent-4-enoate (1.380 g, 4.352 mmol) via method **A** and was purified by flash column chromatography (0-3% Et_2O /hexanes) to afford the product as a bright yellow oil in 8% yield (106 mg, 353 μmol). The compound degrades rapidly and should therefore be used immediately after preparation.
^1H NMR (400 MHz, $CDCl_3$) δ 7.02 – 6.92 (m, 1H), 6.85 (d, *J* = 3.9 Hz, 1H), 5.43 (s, 1H), 5.06 (d, *J* = 1.3 Hz, 1H), 3.80 (s, 3H), 3.44 (d, *J* = 1.3 Hz, 2H).
^{13}C NMR: (151 MHz, $CDCl_3$) δ 167.2, 144.5, 136.0, 130.5, 124.7, 113.7, 111.9, 52.1, 28.9.

FTIR(neat): 2951, 2923, 2851, 2081, 1684, 1617, 1525, 1435, 1341, 1303, 1178, 1109, 1036, 964, 891, 794, 735, 703, 672, 590, 533, 458 cm^{-1}
HRMS: (+pESI) m/z=M+1H calcd for $C_{10}H_{10}N_2O_2SBr$ 300.9641; Found: 300.9641

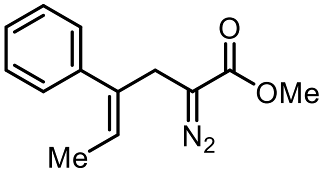

Methyl (Z)-2-diazo-4-phenylhex-4-enoate: Product **19a** is obtained from the precursor crude methyl (*Z*)-2-acetyl-4-phenylhex-4-enoate (Viscous yellow oil, 86% yield from first step, 4.1041 g, 16.66 mmol) ^{1}H NMR: δ 7.41 – 7.31 (m, 2H), 7.29 – 7.19 (m, 2H), 7.15 – 7.06 (m, 2H), 5.62 (qt, *J* = 6.8, 1.2 Hz, 1H), 3.65 (d, *J* = 1.4 Hz, 3H), 3.42 (t, *J* = 7.5 Hz, 1H), 2.91 (d, *J* = 7.4 Hz, 2H), 2.12 (s, 2H), 1.53 (dd, *J* = 6.9, 1.2 Hz, 3H). via method **B-3** and was purified by flash column chromatography (0-3% Et_2O /hexanes) to afford the product as a bright yellow liquid in 70% yield (2.62 g, 11.4 mmol).
^{1}H NMR: (600 MHz, $CDCl_3$) δ 7.45 – 7.32 (m, 2H), 7.30 – 7.23 (m, 2H), 7.21 – 7.08 (m, 1H), 5.70 (qt, J = 6.9, 1.2 Hz, 1H), 3.69 (s, 3H), 3.35 (d, J = 1.4 Hz, 2H), 1.62 (dd, J = 6.9, 1.2 Hz, 3H).
^{13}C NMR (151 MHz, $CDCl_3$) δ 171.1, 139.2, 136.3, 128.4, 128.3, 127.1, 124.5, 51.8, 32.6, 14.7.
FTIR(neat): 2952, 2079, 1737, 1687, 1493, 1436, 1338, 1295, 1240, 1187, 1116, 1075, 1046, 911, 804, 781, 763, 732, 730, 699, 647, 611, 569, 531 cm^{-1}
HRMS: (+pAPCI) m/z=M+1H calcd for $C_{13}H_{15}N_2O_2$ 231.1128; Found: 231.1131

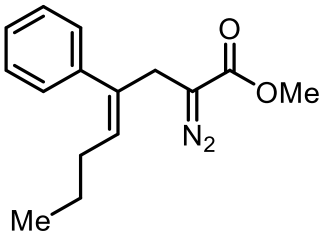

Methyl (Z)-2-diazo-4-phenyloct-4-enoate: Product **19b** is obtained from the crude precursor methyl (*Z*)-2-acetyl-4-phenyloct-4-enoate (Viscous yellow oil, up to 61% yield from the first step) [^{1}H NMR: δ 7.32 (t, *J* = 7.5 Hz, 2H), 7.24 (s, 1H), 7.11 (d, *J* = 7.5 Hz, 2H), 5.51 (t, *J* = 7.4 Hz, 1H), 3.82 – 3.57 (m, 3H), 3.43 (t, *J* = 7.5 Hz, 1H), 2.89 (dd, *J* = 7.7, 2.9 Hz, 3H), 2.12 (s, 3H), 1.99 – 1.77 (m, 3H), 1.30 (p, *J* = 7.3 Hz, 3H), 0.80 (t, *J* = 7.5 Hz, 3H)] (2.432 g, 8.862 mmol) via method **B-3** and was purified by flash column chromatography (0-3% Et_2O /hexanes) to afford the product as a bright yellow liquid (1.709 g, 75% yield, 6.614 mmol) which slowly decomposed in the refrigerator (4 months).
^{1}H NMR (600 MHz, $CDCl_3$) δ 7.39 – 7.32 (m, 2H), 7.31 – 7.25 (m, 1H), 7.21 – 7.16 (m, 2H), 5.63 – 5.57 (m, 1H), 3.72 (s, 3H), 3.36 (s, 2H), 1.99 (q, J = 7.6 Hz, 2H), 1.38 (h, J = 7.4 Hz, 2H), 0.86 (t, J = 7.4 Hz, 3H).
^{13}C NMR (151 MHz, $CDCl_3$) δ 167.4, 139.5, 135.4, 130.7, 128.4, 128.3, 127.1, 51.9, 32.7, 30.9, 23.0, 13.7.
FTIR(neat): 2956, 2871, 2078, 1689, 1493, 1435, 1378, 1294, 1187, 1107, 1022, 909, 805, 781, 741, 698, 623, 610, 533 cm^{-1}
HRMS: (+pAPCI) m/z=M+1H calcd for $C_{15}H_{19}N_2O_2$ 259.1441; Found: 259.1443

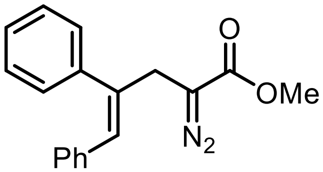

Methyl (Z)-2-diazo-4,5-diphenylpent-4-enoate: Product **19c** is obtained from crude methyl (*Z*)-2-acetyl-4,5-diphenylpent-4-enoate (Gummy off-white solid, up to 93% yield from the first step) [^{1}H NMR: δ 7.59 – 7.37 (m, 3H), 7.38 – 7.22 (m, 4H), 7.21 – 7.14 (m, 2H), 7.10 (tt, *J* = 3.3, 1.3 Hz, 3H), 6.97 – 6.87 (m, 2H), 6.54 (d, *J* = 1.1 Hz, 1H), 3.73 (s, 3H), 3.37 (ddd, *J* = 5.5, 4.2, 2.2 Hz, 1H), 3.11 (dd, *J* = 7.5, 1.2 Hz, 2H), 2.20 (s, 3H)] (3.683 g, 11.94 mmol) via method **B-3** and was purified by flash

column chromatography (0-10% Et_2O /hexanes) to afford the product as a bright orange liquid in 72% yield (2.51 g, 8.59 mmol).

^1H NMR (600 MHz, $CDCl_3$) δ 7.57 – 7.50 (m, 1H), 7.46 – 7.38 (m, 2H), 7.34 (d, *J* = 7.4 Hz, 2H), 7.26 – 7.21 (m, 1H), 7.16 – 7.11 (m, 2H), 7.01 (dd, *J* = 7.5, 2.2 Hz, 2H), 6.59 (s, 1H), 3.76 (s, 3H), 3.54 (d, *J* = 1.3 Hz, 2H).

^{13}C NMR (151 MHz, $CDCl_3$) δ 167.5, 139.6, 136.5, 131.7, 129.2, 128.8, 128.6, 128.0, 127.7, 126.9, 126.4, 52.0, 34.20, 23.7.

FTIR(neat): 3015, 2970, 2950, 2077, 1738, 1688, 1493, 1435, 1338, 1295, 1188, 1106, 918, 806, 758, 741, 693, 574, 537, 508 cm^{-1}

HRMS: (+pAPCI) m/z=M+1H calcd for $C_{18}H_{17}N_2O_2$ 293.1285; Found: 293.1286

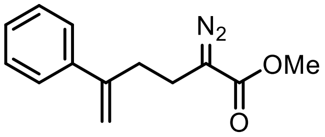

Methyl 5-phenyl-2-diazohex-5-enoate: Product **S1** is obtained from methyl 2-acetyl-5-phenylhex-5-enoate (1.69 g, 6.86 mmol) via method **A** and was purified by flash column chromatography (0-3% Et_2O /hexanes) to afford the product as a bright yellow liquid in 50% yield (786 mg, 3.41 mmol).

^1H NMR: (600 MHz, $CDCl_3$) δ 7.42 (d, J = 7.4 Hz, 2H), 7.37 (t, J = 7.3 Hz, 2H), 7.30 (t, J = 7.3 Hz, 1H), 5.37 (s, 1H), 5.16 (d, J = 1.3 Hz, 1H), 3.77 (s, 3H), 2.77 (t, J = 7.3 Hz, 2H), 2.48 (t, J = 7.3 Hz, 2H).

^{13}C NMR (151 MHz, $CDCl_3$) δ 167.8, 146.7, 140.3, 128.5, 127.7, 126.1, 114.1, 51.9, 33.6, 22.6.

FTIR(neat): 2951, 2075, 1684, 1626, 1599, 1574, 1496, 1435, 1349, 1310, 1169, 1118, 1028, 965, 898, 844, 814, 777, 739, 703, 616, 544 cm^{-1}

HRMS: (+pAPCI) m/z=M+1H calcd for $C_{13}H_{15}N_2O_2$ 231.1128; Found: 231.1122

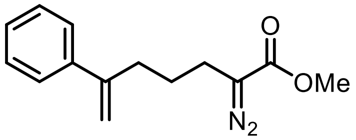

Methyl 2-diazo-6-phenylhept-6-enoate: Product **S2** is obtained from methyl 2-acetyl-6-phenylhept-6-enoate (1.168 g, 4.49 mmol) via method **A** and was purified by flash column chromatography (0-3% Et_2O /hexanes) to afford the product as a bright yellow liquid in 31% yield (343 mg, 1.40 mmol).

^1H NMR (400 MHz, $CDCl_3$) δ 7.44 – 7.39 (m, 2H), 7.36 (ddd, J = 8.1, 6.9, 1.0 Hz, 2H), 7.33 – 7.26 (m, 1H), 5.32 (d, J = 1.4 Hz, 1H), 5.11 (d, J = 1.4 Hz, 1H), 3.77 (s, 3H), 2.61 (td, J = 7.5, 1.3 Hz, 2H), 2.36 (t, J = 7.5 Hz, 2H), 1.71 (tt, J = 8.2, 6.9 Hz, 2H).

^{13}C NMR (101 MHz, $CDCl_3$) δ 169.8, 147.5, 140.9, 128.4, 127.5, 126.1, 113.0, 51.9, 34.3, 26.2, 22.8.

FTIR(neat): 2949, 2077, 1738, 1738, 1686, 1626, 1600, 1573, 1495, 1435, 1344, 1306, 1266, 1188, 1160, 1118, 1075, 1027, 896, 808, 778, 758, 738, 699, 539, 500 cm^{-1}

HRMS: (+pESI) m/z=M+1H calcd for $C_{14}H_{17}N_2O_2$ 245.1285; Found: 245.1284

6. Characterization of known reaction intermediates and scope products

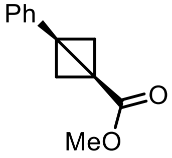

Methyl 3-phenylbicyclo[1.1.0]butane-1-carboxylate: Product **18a** is obtained as an intermediate during method **C** from the reaction between methyl 2-diazo-4-phenylpent-4-enoate (43 mg, 0.20 mmol) and $Rh_2(Oct)_4$ (15 µg, 0.020 µmol) as an off-white amorphous solid in >99% yield (38 mg, 0.20 mmol). Spectra matched the previously reported compound in literature.[5]

^1H NMR: (600 MHz, $CDCl_3$) δ 7.34 – 7.30 (m, 4H), 7.28 – 7.24 (m, 1H), 3.51 (s, 3H), 2.95 (s, 2H), 1.63 (s, 2H).

^{13}C NMR (151 MHz, $CDCl_3$) δ 170.1, 133.6, 128.5, 127.0, 125.9, 51.8, 35.8, 33.0, 23.3.

FTIR(neat): 3016, 2970, 2949, 1738, 1602, 1526, 1443, 1402, 1365, 1343, 1228, 1216, 1204, 1154, 1112, 1068, 1025, 998, 890, 784, 745, 694, 548, 527, 515 cm^{-1}

HRMS: (+pAPCI) m/z=M+1H calcd for $C_{12}H_{13}O_2$ 189.0910; Found: 189.0908

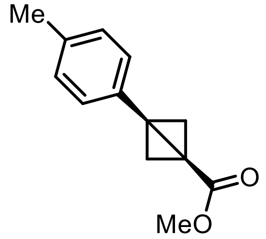

Methyl 3-(*p*-tolyl)bicyclo[1.1.0]butane-1-carboxylate: Product **18b** is obtained as an intermediate during method **C** from the reaction between methyl 2-diazo-4-(*p*-tolyl)pent-4-enoate (46m g, 0.20 mmol) and $Rh_2(Oct)_4$ (15 µg, 0.020 µmol) as a white crystalline solid in 72% yield (29 mg, 0.14 mmol)). Spectra matched the previously reported compound in literature.[5]

^{1}H NMR (600 MHz, $CDCl_3$) δ 7.21 (d, J = 8.1 Hz, 2H), 7.13 (d, J = 7.8 Hz, 2H), 3.51 (s, 3H), 2.92 (s, 2H), 2.34 (s, 3H), 1.60 (s, 2H).

^{13}C NMR: (151 MHz, $CDCl_3$) δ 170.2, 136.8, 130.4, 129.2, 125.9, 51.8, 35.8, 33.2, 22.9, 21.1.

FTIR(neat): 2951, 1707, 1607, 1532, 1499, 1440, 1404, 1344, 1194, 1157, 1112, 1097, 1067, 1018, 984, 891, 820, 770, 745, 539 cm^{-1}

HRMS: (+pAPCI) m/z=M+1H calcd for $C_{13}H_{15}O_2$ 203.1067; Found: 203.1065

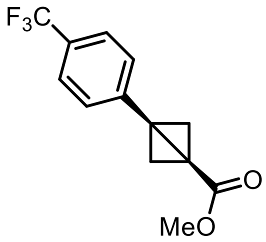

Methyl 3-(4-(trifluoromethyl)phenyl)bicyclo[1.1.0]butane-1-carboxylate: Product **18c** is obtained as an intermediate during method **C** from the reaction between methyl 2-diazo-4-(4-trifluoromethyl)phenylpent-4-enoate (20 mg, 70 µmol) and $Rh_2(Oct)_4$ (5.5 µg, 0.0070 µmol) as a white amorphous solid in >99% yield (18 mg, 0.20 mmol). Spectra matched the previously reported compound in literature.[5]

^{1}H NMR (600 MHz, $CDCl_3$) δ 7.57 (d, J = 8.1 Hz, 2H), 7.40 (d, J = 8.4 Hz, 2H), 3.52 (s, 3H), 2.98 (s, 2H), 1.69 (s, 2H).

^{13}C NMR (151 MHz, $CDCl_3$) δ 169.5, 138.3, 129.0 (q, J = 32.6 Hz), 126.1, 125.4 (q, J = 3.8 Hz), 52.0, 36.0, 31.7, 24.5.

^{19}F NMR (565 MHz, CDCl3) δ -62.49 (s, 3F).

FTIR(neat): 3015, 2970, 2950, 1738, 1617, 1440, 1365, 1324, 1228, 1216, 1204, 1161, 1116, 1063, 1014, 891, 843, 754, 693, 538, 527, 515 cm^{-1}

HRMS: (+pAPCI) m/z=M+1H calcd for $C_{13}H_{12}O_2F_3$ 257.0784; Found: 257.0777

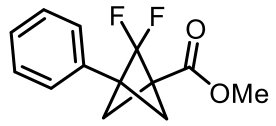

Methyl 2,2-difluoro-3-phenylbicyclo[1.1.1]pentane-1-carboxylate: Compound **21a** was prepared according to method **C** from methyl 2-diazo-4-phenylpent-4-enoate (43 mg, 0.20 mmol) and $Rh_2(Oct)_4$ (15 µg, 0.020 µmol) followed by addition of CF_3TMS (3.0 equiv, 0.60 mmol, 89µl) and NaI (0.5 equiv, 0.10 mmol, 15 mg). Reaction mixture was purified by flash column chromatography (0-10% Et_2O /hexanes) to afford the product as a white amorphous solid in 65% yield (31 mg, 0.13 mmol). Spectra matched the previously reported compound in literature. [5]

^{1}H NMR (400 MHz, $CDCl_3$) δ 7.38 (td, J = 5.6, 2.6 Hz, 3H), 7.30 (dd, J = 7.6, 2.0 Hz, 2H), 3.82 (s, 3H), 2.66 (t, J = 1.1 Hz, 2H), 2.14 (tt, J = 10.4, 1.2 Hz, 2H).

^{13}C NMR (101 MHz, $CDCl_3$) δ 165.8, 132.0, 128.6, 128.4, 127.1, 122.9, 54.8 (t, J = 19.4 Hz), 52.3, 50.2 (t, J = 19.5 Hz), 43.2 (t, J = 7.2 Hz).

^{19}F NMR (565 MHz, $CDCl_3$) δ -120.89 (t, J = 10.2 Hz).

FTIR(neat): 2956, 1793, 1514, 1496, 1439, 1392, 1323, 1238, 1202, 1142, 1110, 1090, 1022, 989, 954, 907, 857, 798, 765, 732, 697, 648, 601, 500 cm^{-1}
HRMS: (+pAPCI) m/z=M+1H calcd for $C_{13}H_{13}F_2O_2$ 239.0878; Found: 239.0882

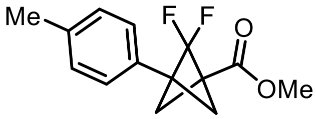

Methyl 2,2-difluoro-3-(*p*-tolyl)bicyclo[1.1.1]pentane-1-carboxylate: Compound **21b** was prepared according to method **C** from methyl 2-diazo-4-(*p*-tolyl)pent-4-enoate (46 mg, 0.20 mmol) and $Rh_2(Oct)_4$ (15 µg, 0.020 µmol) followed by addition of CF_3TMS (3.0 equiv, 0.60 mmol, 89 µl) and NaI (0.5 equiv, 0.10 mmol, 15 mg). Reaction mixture was purified by flash column chromatography (0-10% Et_2O /hexanes) to afford the product as a a white crystalline solid in 42% yield (21 mg, 83 µmol). The structure was confirmed by X-Ray crystallography and spectra matched the previously reported compound in literature.[5]
^1H NMR (600 MHz, $CDCl_3$) δ 7.19 (s, 4H), 3.81 (s, 3H), 2.63 (s, 2H), 2.37 (s, 3H), 2.11 (t, J = 10.4 Hz, 2H).
^{13}C NMR (151 MHz, $CDCl_3$) δ 165.8, 138.2, 129.3, 127.0, 122.9, 54.8, 54.7 (t, J = 19.3 Hz), 52.2, 50.2 (t, J = 19.5 Hz), 43.2 (t, J = 7.4 Hz), 21.2.
^{19}F NMR (565 MHz, $CDCl_3$) δ -121.01 (t, J = 10.4 Hz).
FTIR(neat): 2955, 1738, 1525, 1500, 1438, 1392, 1323, 1240, 1200, 1141, 1108, 1032, 989, 954, 906, 858, 822, 796, 769, 730, 602, 587, 505, 480 cm^{-1}
HRMS: (+pAPCI) m/z=M+1H calcd for $C_{14}H_{15}F_2O_2$ 253.1035; Found: 253.1035

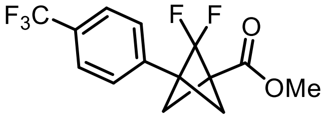

Methyl 2,2-difluoro-3-(4-(trifluoromethyl)phenyl)bicyclo[1.1.1]pentane-1-carboxylate: Compound **21c** was prepared according to method **C** from methyl 2-diazo-4-(4-trifluoromethyl)phenylpent-4-enoate (20 mg, 70 µmol) and $Rh_2(Oct)_4$ (5 µg, 0.007 µmol) followed by addition of CF_3TMS (3.0 equiv, 0.21 mmol, 31 µl) and NaI (0.5 equiv, 35 µmol, 5.3 mg). Reaction mixture was purified by flash column chromatography (0-10% Et_2O /hexanes) to afford the product as a clear colorless oil in 30% yield (6 mg, 20 µmol). Spectra matched the previously reported compound in literature. [5]
^1H NMR (600 MHz, $CDCl_3$) δ 7.65 (d, J = 7.9 Hz, 2H), 7.42 (d, J = 7.9 Hz, 2H), 3.83 (s, 3H), 2.69 (s, 2H), 2.18 (ddd, J = 10.8, 9.6, 1.3 Hz, 2H).
^{13}C NMR (151 MHz, $CDCl_3$) δ 165.8, 132.0, 129.6 (d, J = 2.2 Hz), 128.6, 128.4, 127.1, 54.8 (t, *J* = 19.4 Hz), 52.2, 50.2 (t, J = 19.5 Hz), 43.2 (t, *J* = 7.2 Hz), 29.7.
^{19}F NMR (471 MHz, $CDCl_3$) δ -62.75, -120.70 (t, J = 10.2 Hz).
FTIR(neat): 2958, 1738, 1622, 1503, 1440, 1411, 1395, 1321, 1243, 1108, 1066, 1028, 1017, 990, 954, 843, 790, 751, 711, 603, 507, 483 cm^{-1}
HRMS: (+pAPCI) m/z=M+1H calcd for $C_{14}H_{12}F_5O_2$ 307.0752; Found: 307.0754

7. Characterization of novel compounds:

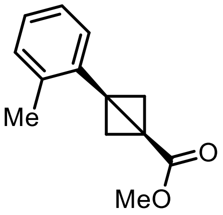

Methyl 3-(*o*-tolyl)bicyclo[1.1.0]butane-1-carboxylate: Product **18d** is obtained as an intermediate during method **C** from the reaction between methyl 2-diazo-4-(*o*-tolyl)pent-4-enoate (46 mg, 0.20 mmol) and $Rh_2(Oct)_4$ (15 µg, 0.020 µmol) as a clear colorless oil in 82% yield (33 mg, 0.16 mmol).
^{1}H NMR (600 MHz, $CDCl_3$) δ 7.25 – 7.15 (m, 3H), 7.12 (td, *J* = 7.5, 1.7 Hz, 1H), 7.06 (d, *J* = 7.5 Hz, 1H), 3.70 (s, 3H), 2.63 (s, 2H), 2.47 (s, 3H), 1.65 (s, 2H).
^{13}C NMR (151 MHz, $CDCl_3$) δ 171.3, 139.1, 132.5, 130.6, 127.3, 126.0, 125.1, 51.9, 38.9, 30.9, 20.7, 20.3.
FTIR(neat): 3016, 2970, 2948, 1738, 1484, 1439, 1365. 1228, 1216, 1204, 1156, 1131, 1091, 1070, 892, 753, 720, 538, 527, 515 cm^{-1}
HRMS: (+pAPCI) m/z=M+1H calcd for $C_{13}H_{15}O_2$ 203.1067; Found: 203.1063

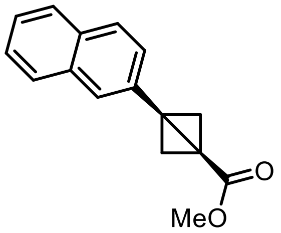

Methyl 3-(naphthalen-2-yl)bicyclo[1.1.0]butane-1-carboxylate: Product **18e** is obtained as an intermediate during method **C** from the reaction between methyl 2-diazo-naphthalen-2-ylpent-4-enoate (53 mg, 0.20 mmol) and $Rh_2(Oct)_4$ (15 µg, 0.020 µmol) as a yellow crystalline solid in 92% yield (44 mg, 0.18 mmol).
^{1}H NMR (600 MHz, $CDCl_3$) δ 7.85 – 7.81 (m, 3H), 7.80 (d, J = 8.6 Hz, 1H), 7.48 (dddd, J = 20.4, 8.0, 6.8, 1.4 Hz, 2H), 7.41 (dd, J = 8.5, 1.7 Hz, 1H), 3.48 (s, 3H), 3.08 (s, 2H), 1.71 (s, 2H).
^{13}C NMR (151 MHz, $CDCl_3$) δ 170.1, 133.4, 132.5, 131.3, 128.2, 127.7, 127.6, 126.4, 125.8, 125.7, 123.2, 51.9, 36.0, 33.4, 23.5.
FTIR(neat): 3016, 2970, 2949, 1738, 1628, 1600, 1501, 1439, 1365, 1334, 1228, 1216, 1203, 1155, 1134, 1090, 1065, 958, 950, 901, 856, 817, 772, 748, 653, 527, 516, 477 cm^{-1}
HRMS: (+pAPCI) m/z=M+1H calcd for $C_{16}H_{15}O_2$ 239.1067; Found: 239.1066

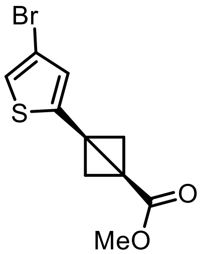

Methyl 3-(4-bromothiophen-2-yl)bicyclo[1.1.0]butane-1-carboxylate: Product **18g** is obtained as an intermediate during method **C** from the reaction between methyl 2-diazo-4-(4-bromothiophen-2-yl)pent-4-enoate (30 mg, 0.10 mmol) and $Rh_2(Oct)_4$ (7.8 µg, 0.010 µmol) as an off-white solid in 95% yield (26 mg, 95 µmol).
^{1}H NMR (600 MHz, $CDCl_3$) δ 7.05 (d, J = 1.5 Hz, 1H), 6.89 (d, J = 1.1 Hz, 1H), 3.60 (s, 4H), 2.88 (s, 2H), 1.76 (s, 2H).
^{13}C NMR (151 MHz, $CDCl_3$) δ 169.1, 139.0, 127.4, 121.4, 109.8, 52.2, 37.9, 29.4, 24.2.
FTIR(neat): 3016, 2970, 2949, 1738, 1440, 1365, 1306, 1228, 1216, 1157, 1091, 1032, 885, 818, 769, 740, 593, 538, 527, 515 cm^{-1}
HRMS: (+pAPCI) m/z=M+1H calcd for $C_{10}H_{10}O_2SBr$ 272.9579; Found: 272.9574

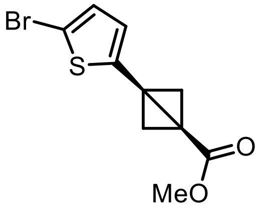

Methyl 3-(5-bromothiophen-2-yl)bicyclo[1.1.0]butane-1-carboxylate: Product **18h** is obtained as an intermediate during method **C** from the reaction between methyl 2-diazo-4-(5-bromothiophen-2-yl)pent-4-enoate (60 mg, 0.20 mmol) and $Rh_2(Oct)_4$ (15 µg, 0.020 µmol) as a waxy solid in 95% yield (52 mg, 0.19 mmol) at >94% purity.
^{1}H NMR (600 MHz, $CDCl_3$) δ 6.92 (d, J = 3.8 Hz, 1H), 6.75 (d, J = 3.8 Hz, 1H), 3.62 (s, 3H), 2.85 (s, 2H), 1.75 (s, 2H).
^{13}C NMR (151 MHz, $CDCl_3$) δ 169.2, 139.0, 130.4, 125.6, 109.9, 52.2, 38.1, 29.9, 23.8.
FTIR(neat): 3001, 2969, 2949, 1738, 1623, 1551, 1498, 1498, 1436, 1365, 1299, 1229, 1216, 1203, 1154, 1112, 1062, 1028, 997, 961, 880, 860, 794, 768, 735, 654, 577, 539, 527, 515 cm^{-1}
HRMS: (+pAPCI) m/z=M+1H calcd for $C_{10}H_{10}O_2SBr$ 272.9579; Found: 272.9581

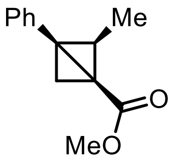

Methyl 2-methyl-3-phenylbicyclo[1.1.0]butane-1-carboxylate: Product **20a** is obtained as an intermediate in 50% ee during method **C** from the reaction between methyl (Z)-2-diazo-4-phenylhex-4-enoate (46 mg, 0.20 mmol) and $Rh_2(Oct)_4$ (15 µg, 0.020 µmol) as a yellow oil in 84% yield (39 mg, 0.17 mmol) in >90% purity and is observed via ^{1}H NMR.
^{1}H NMR (600 MHz, $CDCl_3$) δ 7.34 – 7.31 (m, 4H), 7.29 – 7.23 (m, 1H), 3.69 (s, 3H), 2.67 (d, J = 1.5 Hz, 1H), 1.81 (q, J = 6.1 Hz, 1H), 1.46 (d, J = 6.1 Hz, 3H), 1.40 (d, J = 1.5 Hz, 1H).
^{13}C NMR (151 MHz, $CDCl_3$) δ 171.0, 133.3, 128.5, 127.8, 127.0, 51.5, 44.8, 36.6, 34.8, 24.0, 12.1.
FTIR(neat): 2948, 1708, 1602, 1522, 1483, 1439, 1371, 1331, 1192, 1155, 1120, 1097, 1042, 1025, 910, 843, 755, 701, 696, 596 cm^{-1}
HRMS: (+pAPCI) m/z=M+1H calcd for $C_{13}H_{15}O_2$ 203.1067; Found: 203.1065
Chiral HPLC: (OD-H, 0/100 IPA/hexanes, flow rate= 1.0 mL/min, l=230 nm) RT: 20.3 min (minor), 25.6 min (major).

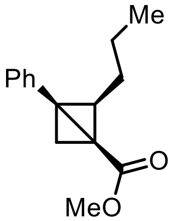

Methyl 3-phenyl-2-propylbicyclo[1.1.0]butane-1-carboxylate: Product **20b** is obtained as an intermediate during method **C** from the reaction between methyl (Z)-2-diazo-4-phenyloct-4-enoate (52 mg, 0.20 mmol) and $Rh_2(Oct)_4$ (15 µg, 0.020 µmol) as a yellow oil in 84% yield (39 mg, 0.17 mmol)and is observed via ^{1}H NMR.
^{1}H NMR (600 MHz, $CDCl_3$) δ 7.30 (d, J = 4.5 Hz, 4H), 7.26 – 7.21 (m, 1H), 3.66 (s, 3H), 2.58 (d, J = 1.4 Hz, 1H), 1.82 (ddd, J = 10.1, 8.2, 5.5 Hz, 1H), 1.78 – 1.71 (m, 1H), 1.68 (dd, J = 8.1, 5.2 Hz, 1H), 1.63 (dtt, J = 9.9, 7.5, 5.5 Hz, 1H), 1.54 – 1.43 (m, 1H), 1.33 (d, J = 1.4 Hz, 1H), 0.95 (t, J = 7.3 Hz, 3H).
^{13}C NMR (151 MHz, $CDCl_3$) δ 170.9, 133.5, 128.5, 127.9, 127.0, 51.6, 50.9, 36.3, 34.2, 28.7, 22.9, 22.6, 14.2.
FTIR(neat): 2957, 2872, 1736, 1713, 1603, 1439, 1365, 1332, 1228, 1194, 1155, 1099, 1027, 912, 782, 753, 696, 527 cm^{-1}
HRMS: (+pAPCI) m/z=M+1H calcd for $C_{15}H_{19}O_2$ 231.1380; Found: 231.1381

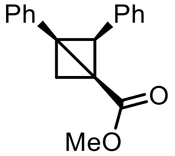

Methyl 2,3-diphenylbicyclo[1.1.0]butane-1-carboxylate: Product **20c** is obtained as an intermediate during method **C** from the reaction between methyl (Z)-2-diazo-4,5-diphenylpent-4-enoate (58 mg, 0.20 mmol) and $Rh_2(Oct)_4$ (15 µg, 0.020 µmol) as an off-white solid in 34% yield (18 mg, 68 µmol) and is observed via ^{1}H NMR.
^{1}H NMR (600 MHz, $CDCl_3$) δ 7.28 – 7.23 (m, 3H), 7.22 – 7.14 (m, 5H), 6.98 (dd, J = 8.1, 1.6 Hz, 2H), 3.68 (s, 3H), 2.68 (d, J = 0.8 Hz, 1H), 2.66 (s, 1H), 1.52 (s, 1H).
^{13}C NMR (151 MHz, $CDCl_3$) δ 169.8, 133.1, 132.0, 129.2, 128.5, 128.4, 127.3, 127.1, 126.9, 51.7, 51.1, 36.4, 34.0, 25.3.

FTIR(neat): 3027, 2946, 1705, 1605, 1521, 1498, 1580, 1439, 1413, 1267, 1198, 1155, 1087, 1026, 970, 936, 910, 878, 781, 760, 698 cm^{-1}
HRMS: (+pAPCI) m/z=M+1H calcd for $C_{18}H_{17}O_2$ 265.1223; Found: 265.1222

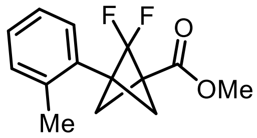

Methyl 2,2-difluoro-3-(*o*-tolyl)bicyclo[1.1.1]pentane-1-carboxylate: Compound **21d** was prepared according to method **C** from methyl 2-diazo-4-(*o*-tolyl)pent-4-enoate (46 mg, 0.20 mmol) and $Rh_2(Oct)_4$ (15 µg, 0.020 µmol) followed by addition of CF_3TMS (3.0 equiv, 0.60 mmol, 89 µl) and NaI (0.5 equiv, 0.10 mmol, 15 mg). Reaction mixture was purified by flash column chromatography (0-10% Et_2O /hexanes) to afford the product as a clear colorless oil in 42% yield (21 mg, 83 µmol).
^1H NMR (600 MHz, $CDCl_3$) δ 7.26 – 6.98 (m, 4H), 3.82 (s, 3H), 2.75 (s, 2H), 2.43 (s, 3H), 2.24 (ddd, J = 10.8, 9.5, 1.3 Hz, 2H).
^{13}C NMR (151 MHz, $CDCl_3$) δ 165.7, 137.3, 131.0, 130.4, 128.7, 128.5, 126.0, 55.7 (t, J = 19.3 Hz), 52.2, 50.5 (t, J = 19.8 Hz), 43.6 (t, *J* = 7.1 Hz), 20.4.
^{19}F NMR (376 MHz, $CDCl_3$) δ -117.61 (t, J = 10.2 Hz).
FTIR(neat): 2956, 1736, 1509, 1490, 1438, 1383, 1318, 1240, 1198, 1140, 1106, 988, 954, 862, 800, 762, 730, 603, 515, 478 cm^{-1}
HRMS: (+pAPCI) m/z=M+1H calcd for $C_{14}H_{15}F_2O_2$ 253.1035; Found: 253.1035

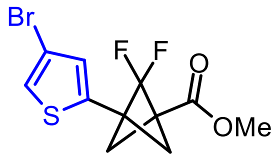

Methyl 3-(4-bromothiophen-2-yl)-2,2-difluorobicyclo[1.1.1]pentane-1-carboxylate: Compound **21e** was prepared according to method **C** from methyl 4-(4-bromothiophen-2-yl)-2-diazopent-4-enoate (26 mg, 86 µmol) and $Rh_2(Oct)_4$ (7 µg, 0.0086 µmol) followed by addition of CF_3TMS (3.0 equiv, 0.26 mmol, 38 µl) and NaI (0.5 equiv, 43 µmol, 6.7 mg). Reaction mixture was purified by flash column chromatography (0-10% Et_2O /hexanes) to afford the product as a yellow oil with a peppery aroma in 43% yield (12 mg, 37 µmol).
^1H NMR (600 MHz, $CDCl_3$) δ 7.16 (t, J = 1.6 Hz, 1H), 6.90 (t, J = 1.5 Hz, 1H), 3.77 (s, 3H), 2.60 (s, 2H), 2.14 (t, J = 10.4 Hz, 2H).
^{13}C NMR (151 MHz, $CDCl_3$) δ 165.1, 135.0, 129.2, 123.2, 109.9, 65.9, 52.4, 50.9 (t, J = 19.6 Hz), 50.3 (t, J = 19.9 Hz), 44.5 (t, J = 6.8 Hz).
^{19}F NMR (565 MHz, $CDCl_3$) δ -120.98 (t, J = 10.1 Hz).
FTIR(neat): 3111, 2955, 1740, 1499, 1441, 1374, 1292, 1245, 1203, 1153, 1103, 990, 840, 811, 742, 585 cm^{-1}
HRMS: (+pAPCI) m/z=M+1H calcd for $C_{11}H_{10}O_2F_2SBr$ 322.9548; Found: 322.9548

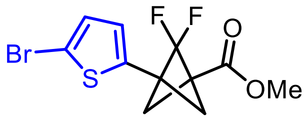

Methyl 3-(5-bromothiophen-2-yl)-2,2-difluorobicyclo[1.1.1]pentane-1-carboxylate: Compound **21f** was prepared according to method **C** from methyl 4-(5-bromothiophen-2-yl)-2-diazopent-4-enoate (60 mg, 0.20 mmol) and $Rh_2(Oct)_4$ (15 µg, 0.020 µmol) followed by addition of CF_3TMS (3.0 equiv, 0.60 mmol, 89 µl) and NaI (0.5 equiv, 0.10 mmol, 15 mg). Reaction mixture was purified by flash column chromatography (0-10% Et_2O /hexanes) to afford the product as a white amorphous solid in 36% yield (23 mg, 71 µmol) and 90% purity.
^1H NMR (600 MHz, $CDCl_3$) δ 6.97 (d, J = 3.7 Hz, 1H), 6.76 (d, J = 3.9 Hz, 1H), 3.81 (s, 3H), 2.62 (s, 2H), 2.16 (t, J = 9.5, 2H).

^{13}C NMR (151 MHz, $CDCl_3$) δ 165.2, 135.2, 130.1, 127.0, 112.5, 52.3, 51.0 (t, J = 19.5 Hz), 50.6 (t, J = 20.0 Hz), 44.4 (t, J = 6.8 Hz).
^{19}F NMR (471 MHz, $CDCl_3$) δ -121.04 (t, J = 10.0 Hz).
FTIR(neat): 2954, 1739, 1501, 1449, 1438, 1374, 1327, 1289, 1242, 1201, 1181, 1150, 1103, 1054, 990, 959, 838, 795, 723, 600, 498 cm^{-1}
HRMS: (+pAPCI) m/z=M+1H calcd for $C_{11}H_{10}O_2F_2SBr$ 322.9548; Found: 322.9550

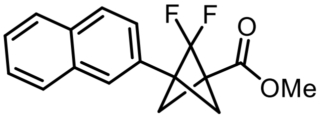

Methyl 2,2-difluoro-3-(naphthalen-2-yl)bicyclo[1.1.1]pentane-1-carboxylate: Compound **21g** was prepared according to method **C** from methyl 2-diazo-naphthalen-2-ylpent-4-enoate (53 mg, 0.20 mmol) and $Rh_2(Oct)_4$ (15 µg, 0.020 µmol) followed by addition of CF_3TMS (3.0 equiv, 0.60 mmol, 89 µl) and NaI (0.5 equiv, 0.10 mmol, 15 mg). Reaction mixture was purified by flash column chromatography (0-10% Et_2O /hexanes) to afford the product as a white amorphous solid in 40% yield (23 mg, 80 µmol).
^1H NMR (600 MHz, $CDCl_3$) δ 7.86 (dd, J = 8.9, 3.6 Hz, 3H), 7.75 (s, 1H), 7.52 (tq, J = 7.3, 3.6 Hz, 2H), 7.42 (dd, J = 8.4, 1.7 Hz, 1H), 3.84 (s, 3H), 2.75 (s, 2H), 2.23 (t, J = 10.2 Hz, 2H).
^{13}C NMR (151 MHz, $CDCl_3$) δ 165.8, 133.1, 133.1, 129.4, 128.5, 127.82, 127.80, 126.6, 126.4, 126.4, 124.5, 55.1 (t, J = 19.4 Hz), 52.3, 50.3 (t, J = 19.6 Hz), 43.3 (t, J = 7.1 Hz).
^{19}F NMR (376 MHz, $CDCl_3$) δ -120.70 (t, J = 10.3 Hz).
FTIR(neat): 3024, 2954, 1736, 1602, 1504, 1438, 1397, 1350, 1317, 1249, 1236, 1205, 1141, 1105, 1016, 989, 958, 897, 860, 818, 749, 720, 602, 514, 478 cm^{-1}
HRMS: (+pAPCI) m/z=M+1H calcd for $C_{17}H_{15}F_2O_2$ 289.1035; Found: 289.1036

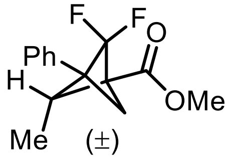

Methyl 2,2-difluoro-4-methyl-3-phenylbicyclo[1.1.1]pentane-1-carboxylate: Product **22a** is obtained as a minor product from general method **C** from the reaction between methyl (Z)-2-diazo-4-phenylhex-4-enoate (46 mg, 0.20 mmol) and Rh_2(*S*-NTTL)$_4$ (28 µg, 0.020 µmol) or $Rh_2(Oct)_4$ (15 µg, 0.020 µmol) followed by addition of CF_3TMS (3.0 equiv, 0.60 mmol, 89 µl) and NaI (0.5 equiv, 0.10 mmol, 15 mg). Reaction mixture was purified by flash column chromatography (0-10% Et_2O /hexanes) to afford the product as a clear colorless oil, obtained in <12% yield as a mixture of enantiomers.
^1H NMR: (600 MHz, $CDCl_3$) δ 7.43 – 7.31 (m, 3H), 7.24 (dd, J = 8.0, 1.6 Hz, 2H), 3.80 (s, 3H), 3.28 (ttd, J = 6.4, 5.4, 3.0 Hz, 1H), 2.79 (ddd, J = 19.3, 3.9, 1.1 Hz, 1H), 2.66 (dtd, J = 7.2, 3.4, 1.6 Hz, 1H), 1.31 (dd, J = 6.4, 1.1 Hz, 3H).
^{13}C NMR: (151 MHz, $CDCl_3$) δ 165.6, 131.3, 128.6, 128.2, 127.2, 52.1, 51.3, 48.0 (d, J = 6.5 Hz), 47.97 (d, J = 6.5 Hz), 36.95(d, J = 7.1), 36.90 (d, J = 7.1), 6.04 (d, J = 5.7 Hz).
^{19}F NMR: (565 MHz, $CDCl_3$) δ -125.39 (d, J = 136.5 Hz), -126.35 (dd, J = 137.1, 19.3 Hz).
FTIR(neat): 2995, 2916, 2848, 1736, 1605, 1494, 1438, 1406, 1385, 1318, 1239, 1181, 1201, 1123, 1097, 1043, 959, 846, 765, 718, 697, 577 cm^{-1}
HRMS: (+pAPCI) m/z=M+1H calcd for $C_{14}H_{15}F_2O_2$ 253.1035; Found: 253.1033

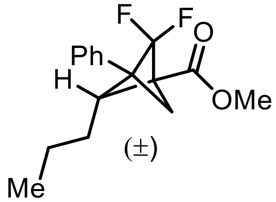

Methyl 2,2-difluoro-4-propyl-3-phenylbicyclo[1.1.1]pentane-1-carboxylate: Product **22b** is obtained as a minor product from general method **C** from the reaction between methyl (Z)-2-diazo-4-phenyloct-4-enoate (52 mg, 0.20 mmol) and Rh_2(*S*-NTTL)$_4$ (28 µg, 0.020 µmol) or $Rh_2(Oct)_4$ (15 µg, 0.020 µmol) followed by addition of CF_3TMS (3.0 equiv, 0.60 mmol, 89 µl) and NaI (0.5 equiv, 0.10 mmol, 15 mg). Reaction mixture was purified by flash column chromatography (0-10% Et_2O /hexanes) to afford the product as a clear colorless oil in 12% yield as a mixture of enantiomers.

^{1}H NMR: (600 MHz, $CDCl_3$) δ 7.40 – 7.32 (m, 3H), 7.26 – 7.23 (m, 2H), 3.80 (s, 3H), 3.18 (qd, J = 6.9, 3.0 Hz, 1H), 2.80 (dq, J = 18.4, 2.2 Hz, 1H), 2.62 (p, J = 3.9 Hz, 1H), 1.77 (td, J = 14.2, 6.5 Hz, 1H), 1.68 – 1.59 (m, 1H), 1.43 – 1.23 (m, 2H), 0.89 (t, J = 7.4 Hz, 3H).

^{13}CNMR: (151 MHz, $CDCl_3$) δ 165.9, 128.6, 128.2, 127.2, 56.6, 54.3, 52.1, 36.63 (d, J = 7.8 Hz), 36.57 (d, J = 7.8 Hz), 30.1, 23.8, 21.8, 14.2.

^{19}F NMR: (376 MHz, $CDCl_3$) δ -125.93 (dt, J = 135.8, 3.3 Hz), -126.48 (ddd, J = 136.7, 17.3, 3.0 Hz).

FTIR(neat): 2969, 1738, 1437, 1365, 1318, 1229, 1216, 1204, 1109, 901, 697, 527 cm^{-1}

HRMS: (+pAPCI) m/z=M+1H calcd for $C_{16}H_{19}F_2O_2$ 281.1348; Found: 281.1351

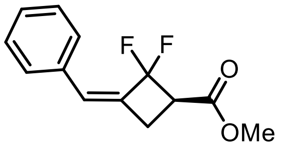

Methyl (R,Z)-3-benzylidene-2,2-difluorocyclobutane-1-carboxylate: Product **26** is obtained as the major product from general method **D** from the reaction between methyl (E)-2-diazo-5-phenylpent-4-enoate (43 mg, 0.20 mmol) and Rh_2(*S*-BTPCP)$_4$ (35 µg, 0.020 µmol) followed by addition of CF_3TMS (3.0 equiv, 0.60 mmol, 89 µl) and NaI (0.5 equiv, 0.10 mmol, 15 mg). Reaction mixture was purified by flash column chromatography (0-2% Et_2O /hexanes) to afford the product as a clear colorless oil in 47% yield in 75% ee.

^{1}H NMR (500 MHz, $CDCl_3$) δ 7.37 – 7.18 (m, 5H), 6.74 (s, 1H), 4.34 (dtd, J = 24.6, 8.0, 2.1 Hz, 1H), 3.66 (s, 3H), 3.11 (dq, J = 8.0, 1.6 Hz, 2H).

^{13}C NMR (151 MHz, $CDCl_3$) δ 169.2, 137.0, 135.7, 134.9, 128.4, 128.3, 128.24, 128.18, 128.1, 75.3 (dd, J = 22.1, 19.8 Hz), 51.7, 31.6, 28.1 (d, J = 5.2 Hz), 22.7.

^{19}F NMR (565 MHz, $CDCl_3$) δ -86.76 (d, J = 41.8 Hz, 1F), -89.86 (dd, J = 41.7, 24.6 (H-F coupling) Hz, 1F).

FTIR(neat): 3027, 2952, 2849, 1745, 1719, 1507, 1495, 1436, 13798, 1290, 1232, 1180, 1116, 1072, 959, 923, 820, 750, 696, 450 cm^{-1}

HRMS: (+pAPCI) m/z=M+1H calcd for $C_{13}H_{13}F_2O_2$ 239.0878; Found: 239.0876

Chiral SFC: (CEL-1, flow rate = 2.5 mL/min, 5/95 MeCN+0.2%FA/CO_2, l=280) tR = 0.60 min (minor), 2.42 min (major).

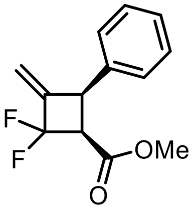

Methyl (1S,4R)-2,2-difluoro-3-methylene-4-phenylcyclobutane-1-carboxylate: Product **27** is obtained as the minor product from general method **D** from the reaction between methyl (E)-2-diazo-5-phenylpent-4-enoate (43 mg, 0.20 mmol) and Rh_2(*S*-BTPCP)$_4$ (35 µg, 0.020 µmol) followed by addition of CF_3TMS (3.0 equiv, 0.60 mmol, 89 µl) and NaI (0.5 equiv, 0.10 mmol, 15 mg). Reaction mixture was purified by flash column chromatography (0-2% Et_2O /hexanes) to afford the product as a clear colorless oil in 24% yield in 91% ee.

^{1}H NMR (600 MHz, $CDCl_3$) δ 7.37 – 7.30 (m, 2H), 7.27 – 7.18 (m, 3H), 6.36 (s, 1H), 5.68 (dd, J = 1.4, 0.8 Hz, 1H), 4.76 (d, J = 10.4 Hz, 1H), 4.63 (ddd, J = 24.0, 10.4, 2.4 Hz, 1H), 3.71 (s, 3H).

^{13}C NMR (151 MHz, $CDCl_3$) δ 166.5, 128.8, 128.7, 128.4, 127.5, 127.0, 126.9, 125.9, 80.3 (dd, J = 23.6, 18.6 Hz), 52.0, 41.2 (d, J = 5.2 Hz).

^{19}F NMR (565 MHz, $CDCl_3$) δ -87.47 (d, J = 40.7 Hz, 1F), -88.44 (dd, J = 40.6, 23.8 (H-F coupling) Hz, 1F).

FTIR(neat): 3030, 2954, 1741, 1723, 1628, 1495, 1438, 1315, 1267, 1181, 1150, 1097, 1072, 1031, 990, 946, 926, 861, 847, 814, 758, 698, 561 cm^{-1}

HRMS: (+pAPCI) m/z=M+1H calcd for $C_{13}H_{13}F_2O_2$ 239.0878; Found: 239.0876

Chiral HPLC: (AD-H, flow rate = 1 mL/min, 0/100 IPA/hexanes, l = 280 nm) tR = 4.85 min (minor), 5.20 min (major).

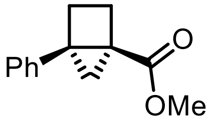

methyl (1S,4S)-4-phenylbicyclo[2.1.0]pentane-1-carboxylate: Product **28** is obtained as a clear colorless oil in 7% ee from method **C** from the reaction between methyl 2-diazo-5-phenylhex-5-enoate (**S1**, 46 mg, 0.20 mmol) and Rh_2(*S-p*-BrTPCP)$_4$ (35 µg, 0.020 µmol) in >99% yield (40 mg, 0.20 mmol).

^{1}H NMR (600 MHz, $CDCl_3$)) δ 7.44 – 7.30 (m, 4H), 7.24 (t, J = 6.9 Hz, 1H), 3.61 (s, 2H), 2.63 (td, J = 10.9, 8.2 Hz, 2H), 2.36 (dt, J = 4.7, 1.7 Hz, 1H), 1.86 (t, J = 7.4 Hz, 1H), 1.74 (t, J = 7.2 Hz, 1H), 1.72 (d, J = 4.7 Hz, 1H).

^{13}C NMR (151 MHz, $CDCl_3$) δ 171.1, 138.2, 128.2, 127.0, 126.5, 51.5, 42.9, 35.7, 29.2, 25.1, 21.3.

FTIR(neat): 2946, 2864, 1708, 1601, 1500, 1435, 1375, 1329, 1288, 1239, 1191, 1147, 1103, 1075, 1028, 1000, 962, 924, 898, 811, 783, 733, 694, 664, 547, 516 cm^{-1}

HRMS: (+pAPCI) m/z=M+1H calcd for $C_{13}H_{15}O_2$ 203.1067; Found: 203.1062

SFC: (OJ-3, 5/95 MeOH/IPA+2%FA/CO_2, flow rate = 2.5 mL/min, l = 210/230) tR = 0.70 min (major), 0.85 min (minor).

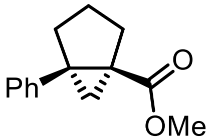

Methyl (1S,5S)-5-phenylbicyclo[3.1.0]hexane-1-carboxylate: Product **29** is obtained as a light pink oil in 65% ee from method **C** from the reaction between methyl 2-diazo-6-phenylhept-6-enoate (**S2**, 53 mg, 0.22 mmol) and Rh_2(*S-p*-BrTPCP)$_4$ (39 µg, 0.022 µmol) in 96% yield (45 mg, 0.21 mmol).

^{1}H NMR (600 MHz, $CDCl_3$) δ 7.31 – 7.25 (overlaps with $CDCl_3$, m, 4H), 7.24 – 7.18 (m, 1H), 3.37 (s, 3H), 2.59 (td, *J* = 12.4, 8.2 Hz, 1H), 2.11 (ddd, *J* = 24.3, 12.5, 7.9 Hz, 2H), 2.02 (dd, *J* = 12.9, 7.9 Hz, 1H), 1.94 – 1.91 (m, 1H), 1.84 (dt, *J* = 13.3, 8.1 Hz, 1H), 1.41 – 1.27 (m, 1H).

^{13}C NMR (151 MHz, $CDCl_3$) δ 172.8, 140.9, 128.8, 128.1, 126.6, 51.3, 44.6, 37.5, 36.6, 29.0, 21.2, 17.8.

FTIR(neat): 2948, 2871, 1715, 1602, 1497, 1435, 1435, 1365, 1272, 1229, 1216, 1200, 1151, 1110, 1077, 1032, 945, 889, 777, 753, 699, 537 cm^{-1}

HRMS: (+pAPCI) m/z=M+1H calcd for $C_{14}H_{17}O_2$ 217.1223; Found: 217.1223

SFC: (SSW, 3/97 MeOH:IPA+2%FA/CO_2, flow rate = 2.5 mL/min, l = 210 nm) tR = 1.11 min (major), 1.46 min (minor).

8. NMR spectra:

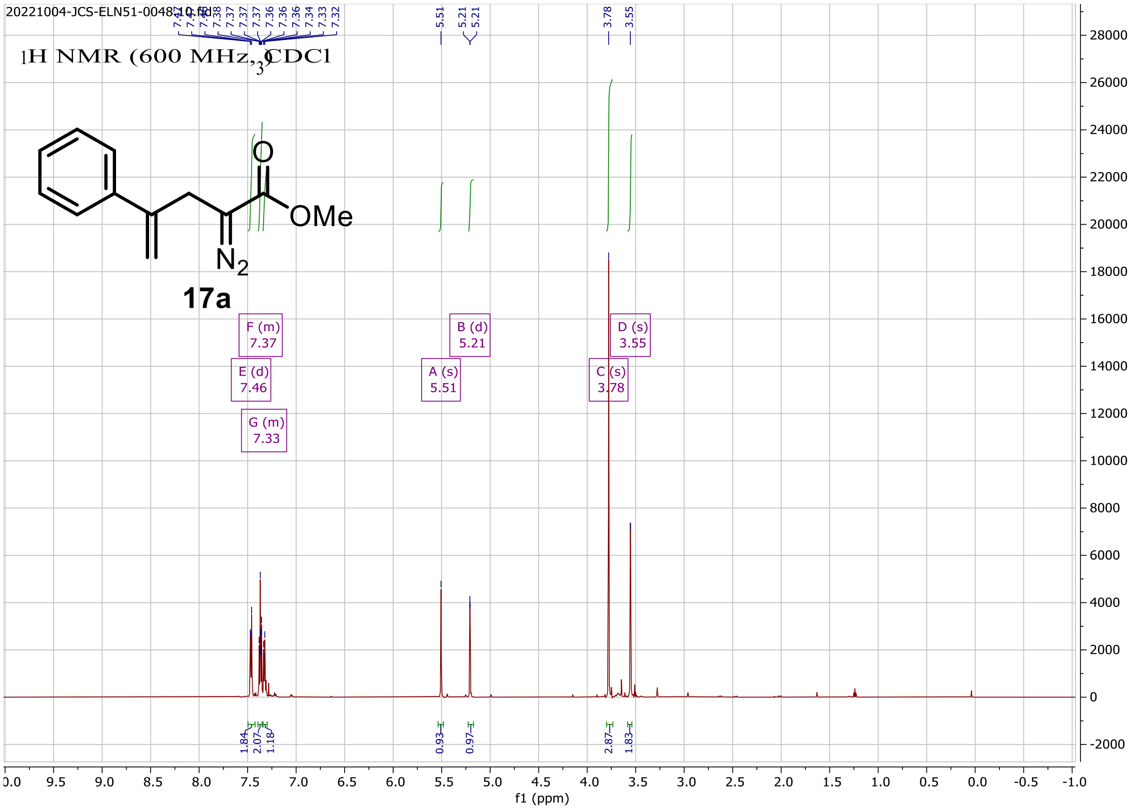

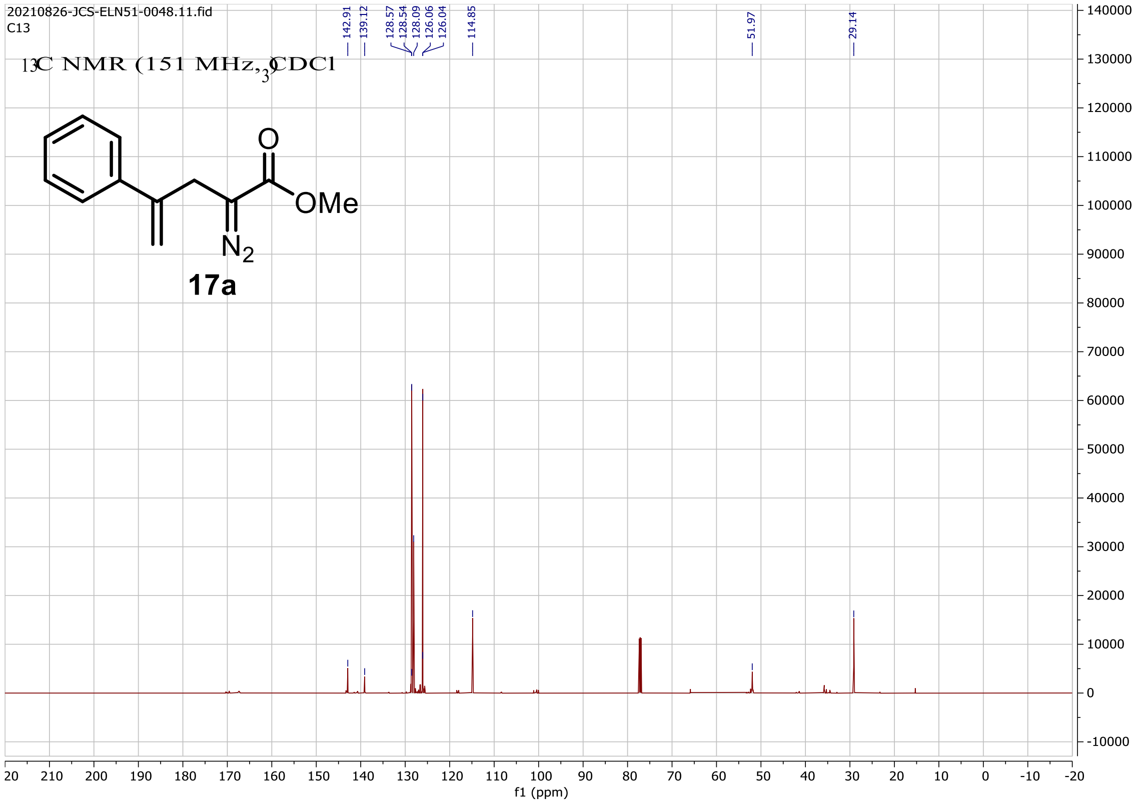

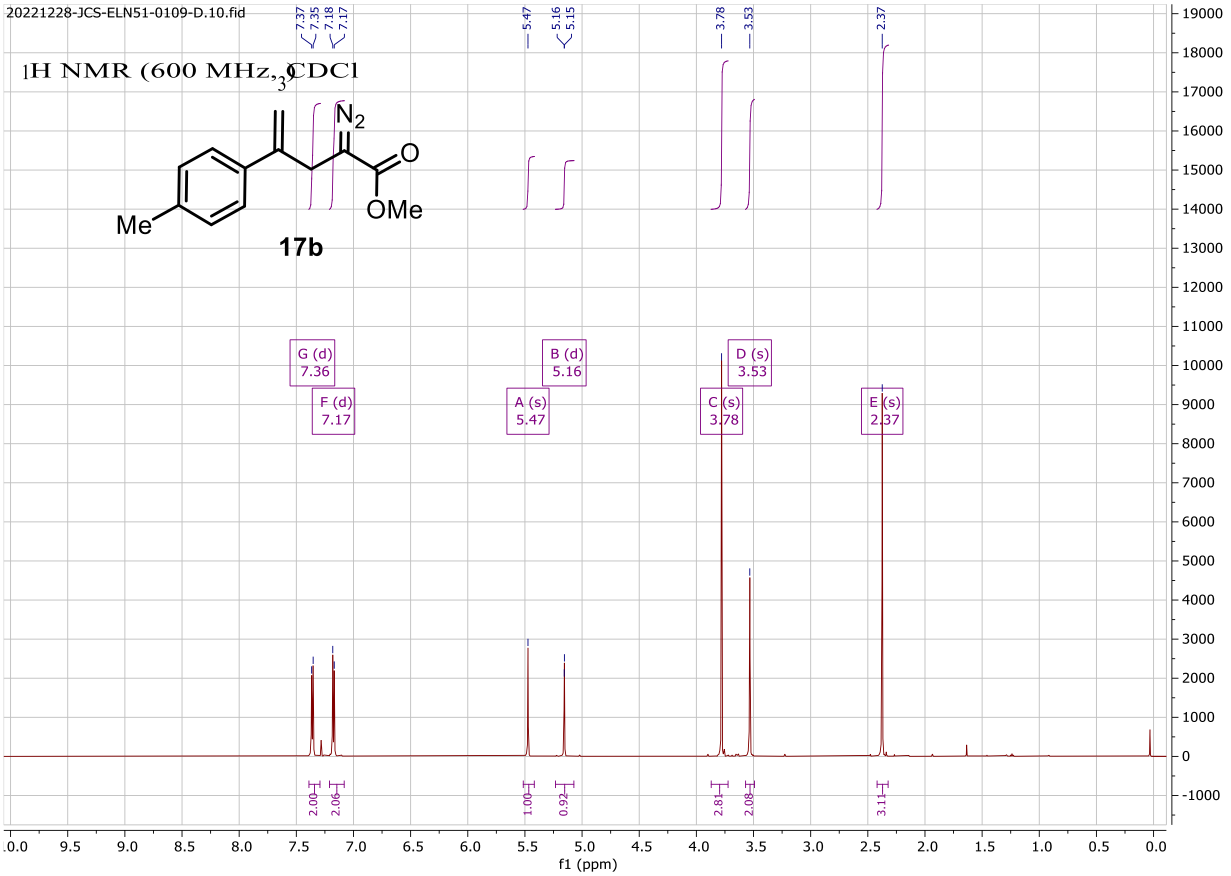
20221228-JCS-ELN51-0109-D.10.fid
1H NMR (600 MHz, CDCl3)
7.37
7.35
7.18
7.17
5.47
5.16
5.15
3.78
3.53
2.37
N2
O
OMe
Me
17b
G (d) 7.36
F (d) 7.17
A (s) 5.47
B (d) 5.16
C (s) 3.78
D (s) 3.53
E (s) 2.37
2.00
2.06
1.00
0.92
2.81
2.08
3.11
f1 (ppm)

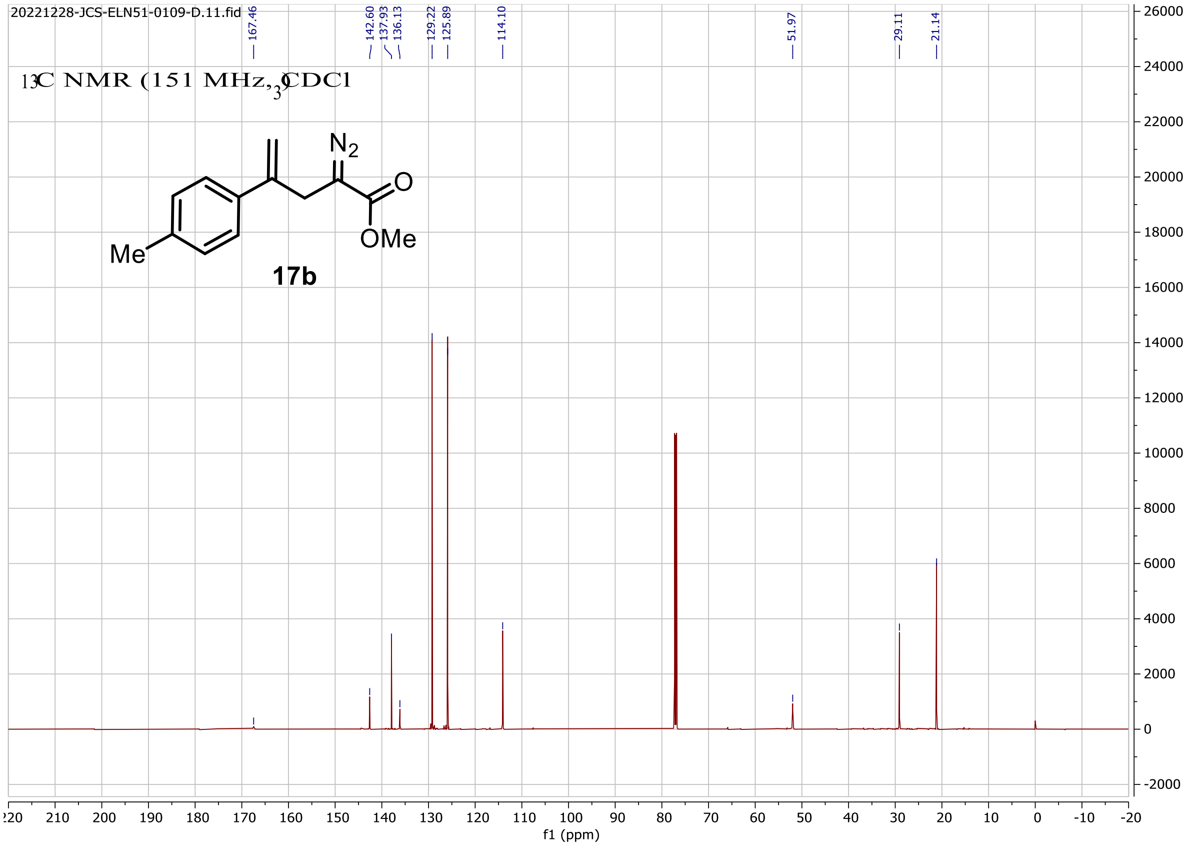
20221228-JCS-ELN51-0109-D.11.fid
13C NMR (151 MHz, CDCl3)
167.46
142.60
137.93
136.13
129.22
125.89
114.10
51.97
29.11
21.14
N2
O
OMe
Me
17b
f1 (ppm)

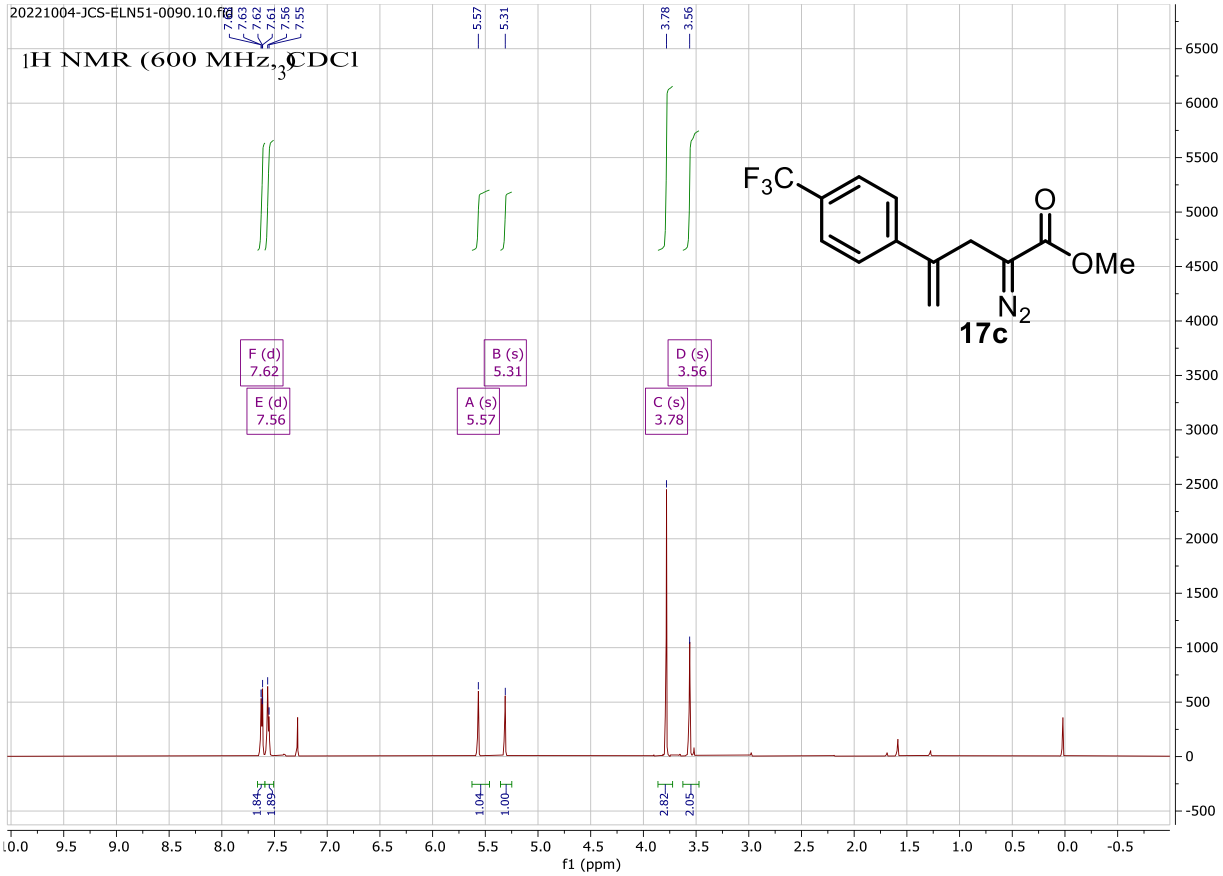
20221004-JCS-ELN51-0090.10.fid
1H NMR (600 MHz, CDCl3)
F3C
O
OMe
N2
17c
F (d)
7.62
E (d)
7.56
A (s)
5.57
B (s)
5.31
C (s)
3.78
D (s)
3.56
f1 (ppm)

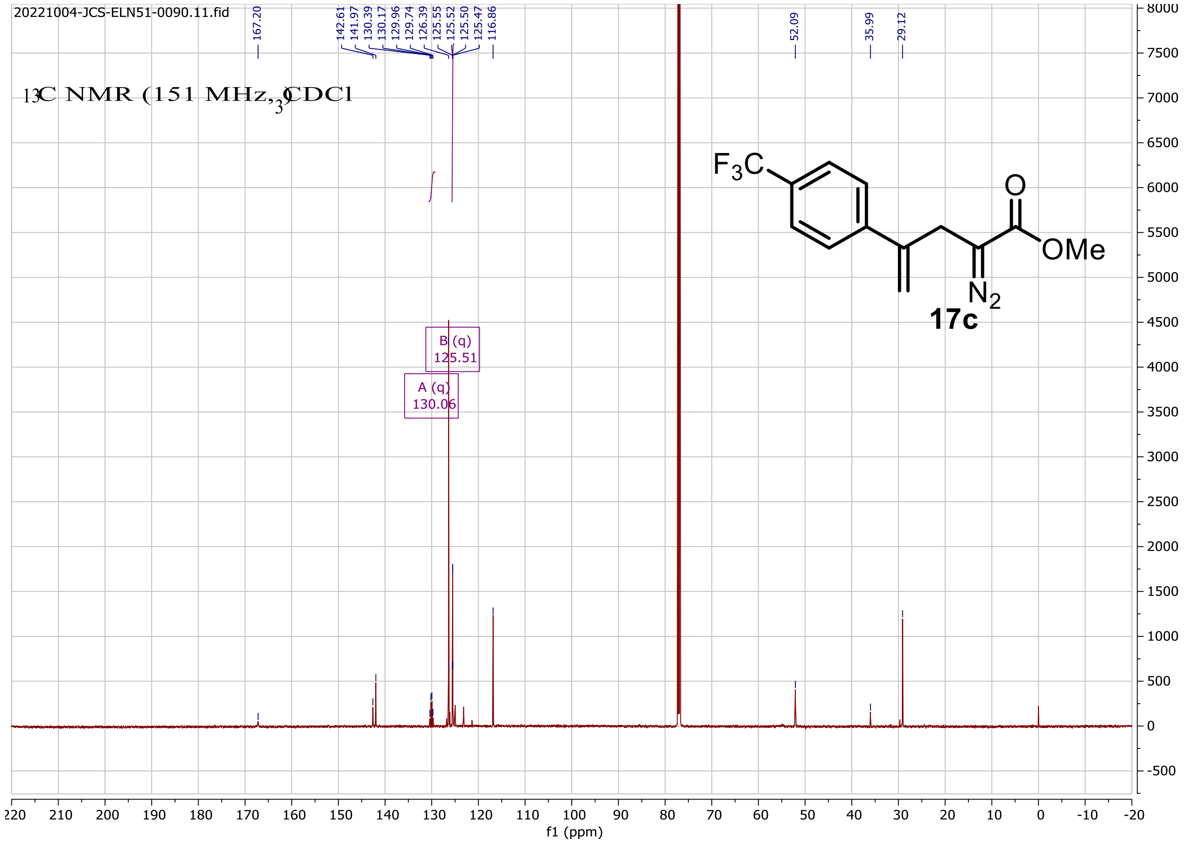
20221004-JCS-ELN51-0090.11.fid
13C NMR (151 MHz, CDCl3)
F3C
O
OMe
N2
17c
B (q)
125.51
A (q)
130.06
f1 (ppm)

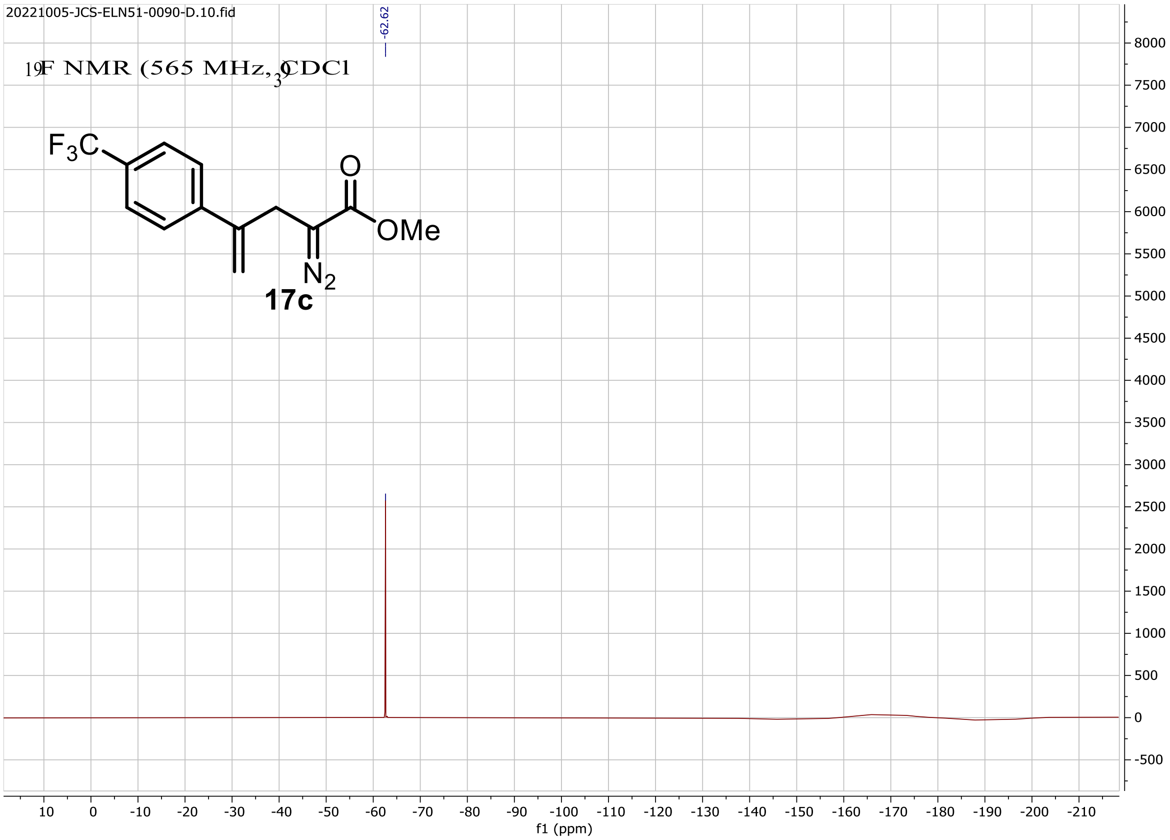
20221005-JCS-ELN51-0090-D.10.fid
-62.62
19F NMR (565 MHz, CDCl3)
F3C
O
OMe
N2
17c
f1 (ppm)

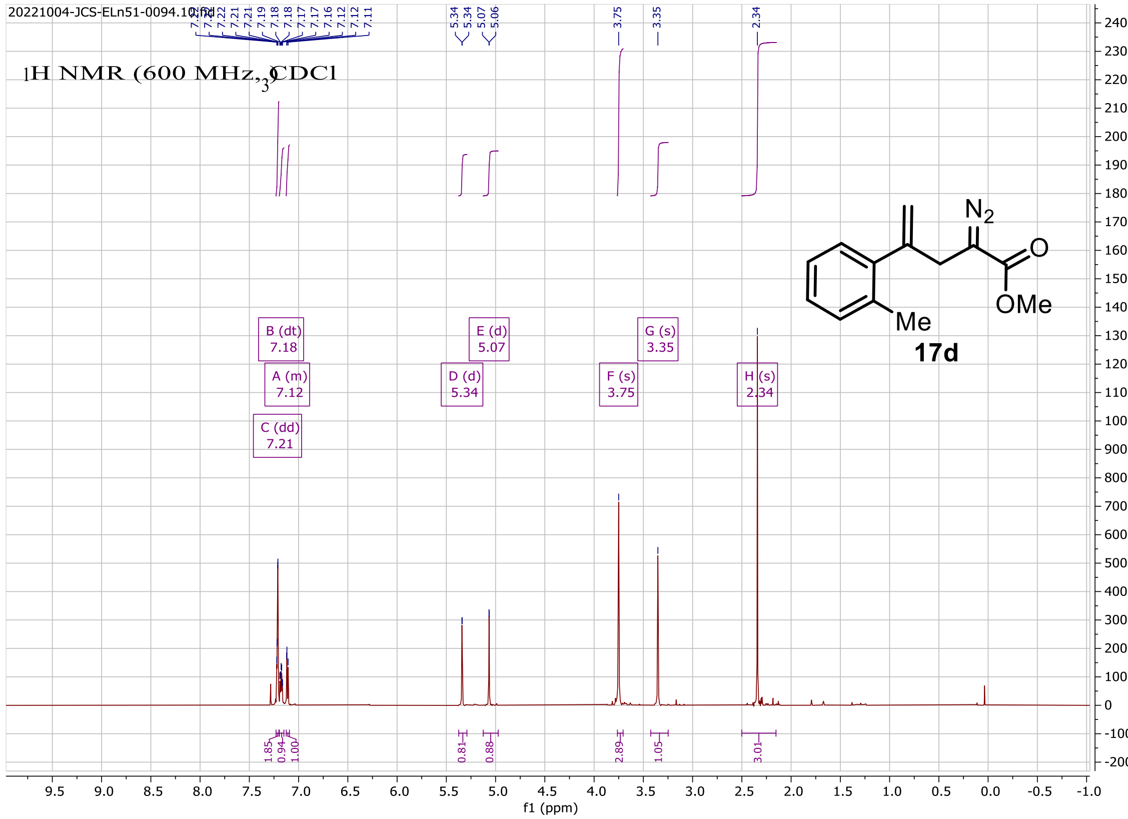
20221004-JCS-ELn51-0094.10.fid
1H NMR (600 MHz, CDCl3)
N2
O
OMe
Me
17d
B (dt) 7.18
A (m) 7.12
C (dd) 7.21
E (d) 5.07
D (d) 5.34
G (s) 3.35
F (s) 3.75
H (s) 2.34
1.85
0.94
1.00
0.81
0.88
2.89
1.05
3.01
f1 (ppm)

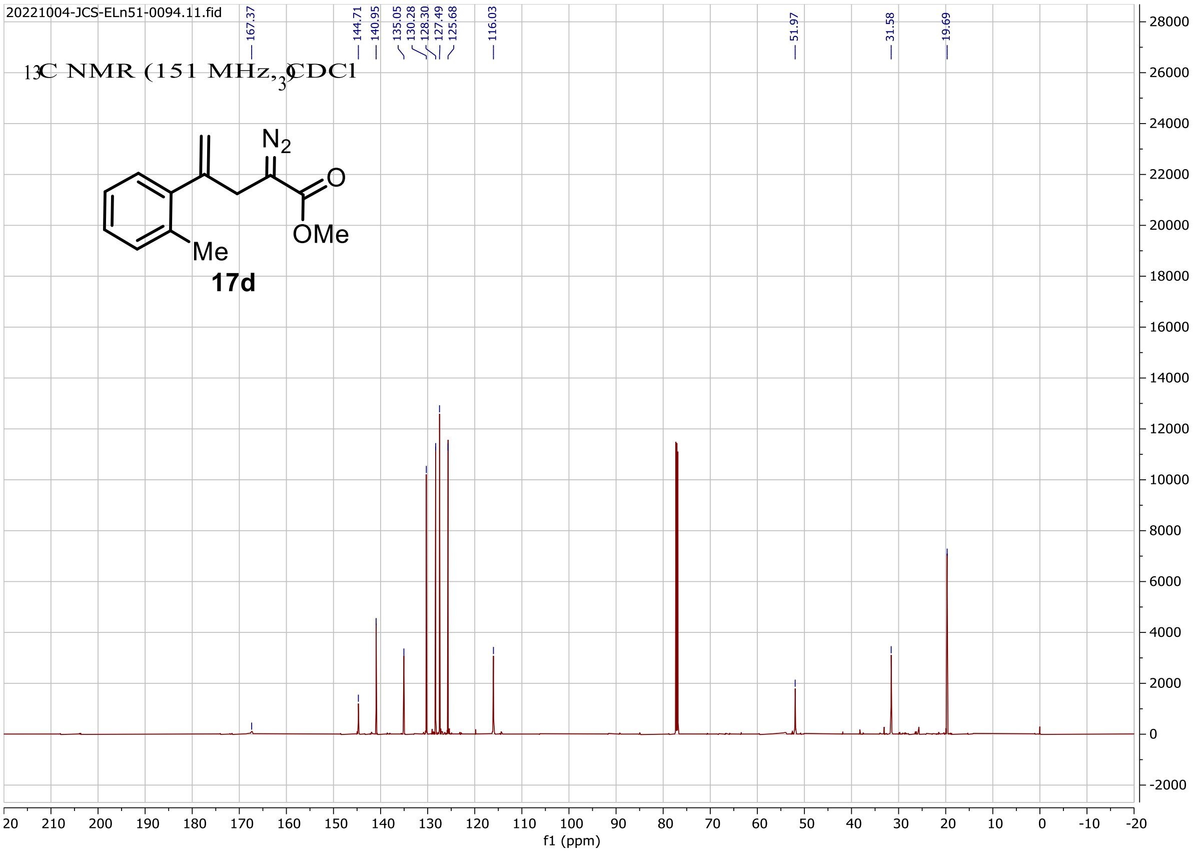
20221004-JCS-ELn51-0094.11.fid
13C NMR (151 MHz, CDCl3)
167.37
144.71
140.95
135.05
130.28
128.30
127.49
125.68
116.03
51.97
31.58
19.69
N2
O
OMe
Me
17d
f1 (ppm)

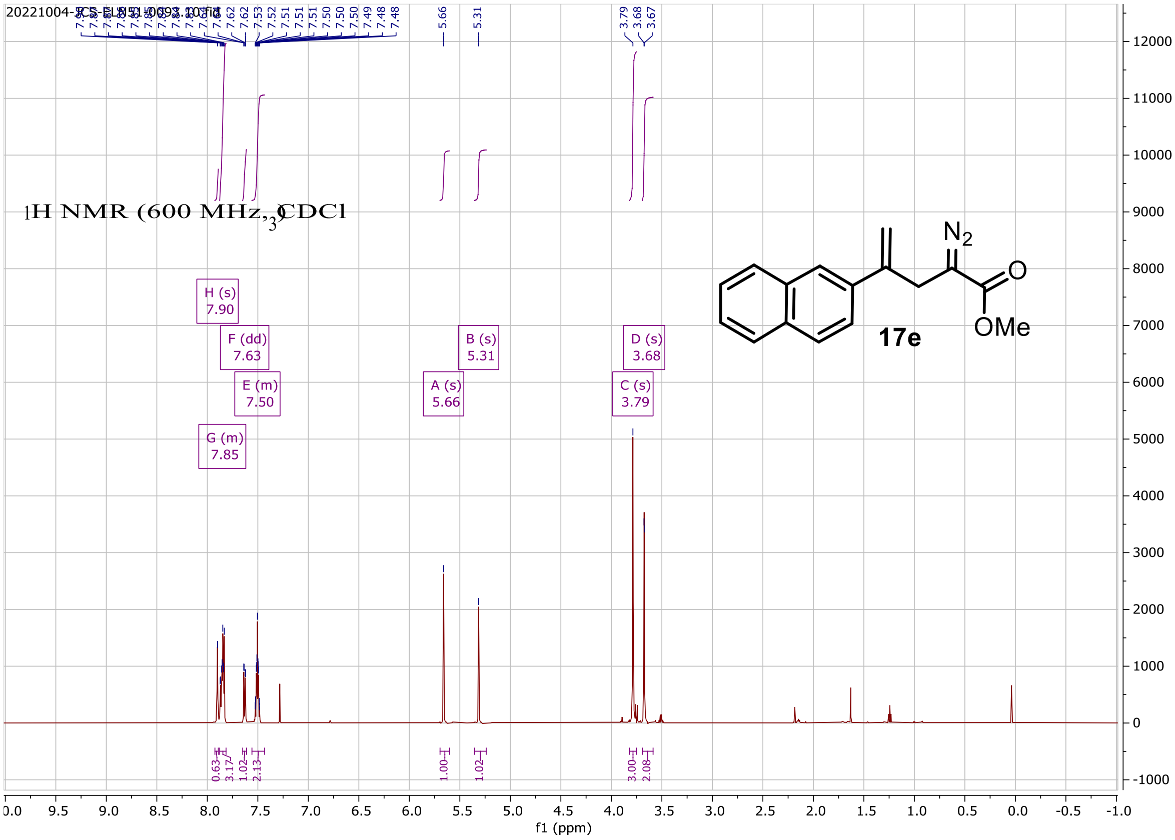
1H NMR (600 MHz, CDCl3)
5.66
5.31
3.79
3.68
3.67
H (s) 7.90
F (dd) 7.63
E (m) 7.50
G (m) 7.85
A (s) 5.66
B (s) 5.31
C (s) 3.79
D (s) 3.68
N2
O
OMe
17e
0.63
3.17
1.02
2.13
1.00
1.02
3.00
2.08
f1 (ppm)

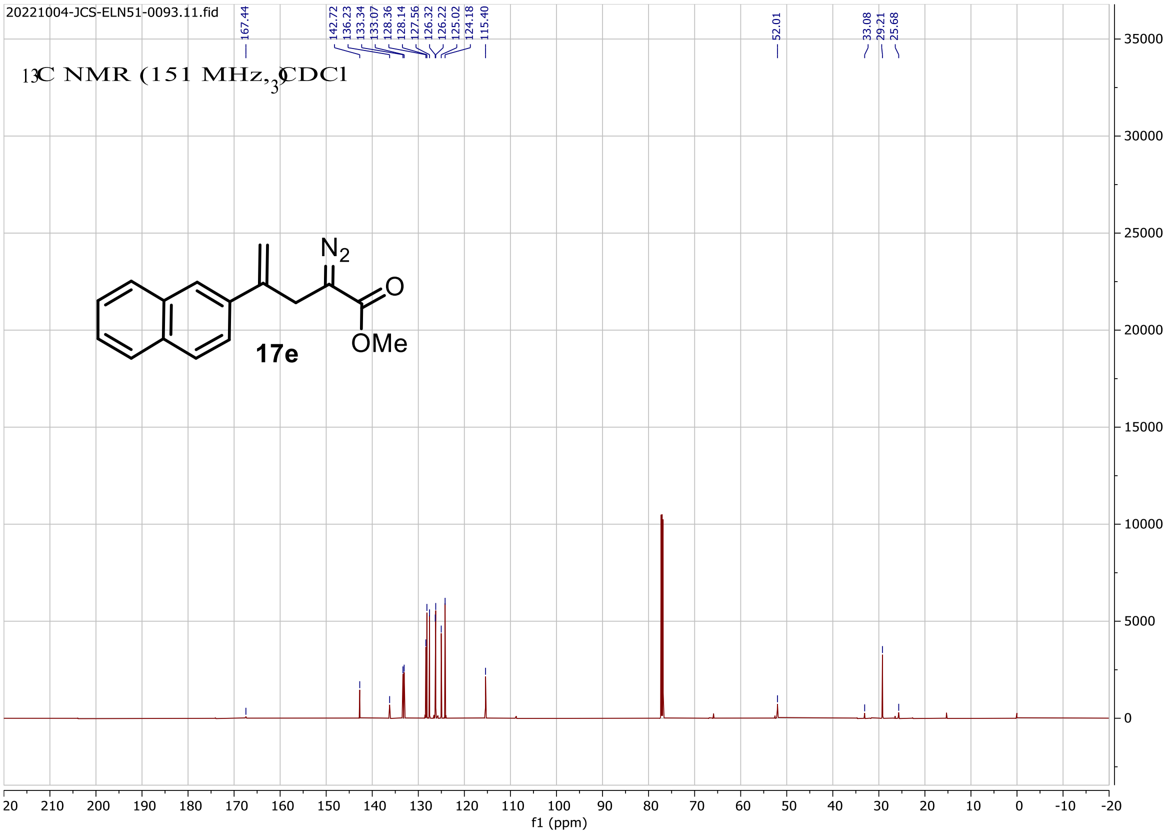

20221004-JCS-ELN51-0093.11.fid
13C NMR (151 MHz, CDCl3)
167.44
142.72
136.23
133.34
133.07
128.36
128.14
127.56
126.32
126.22
125.02
124.18
115.40
52.01
33.08
29.21
25.68
N2
O
OMe
17e
f1 (ppm)

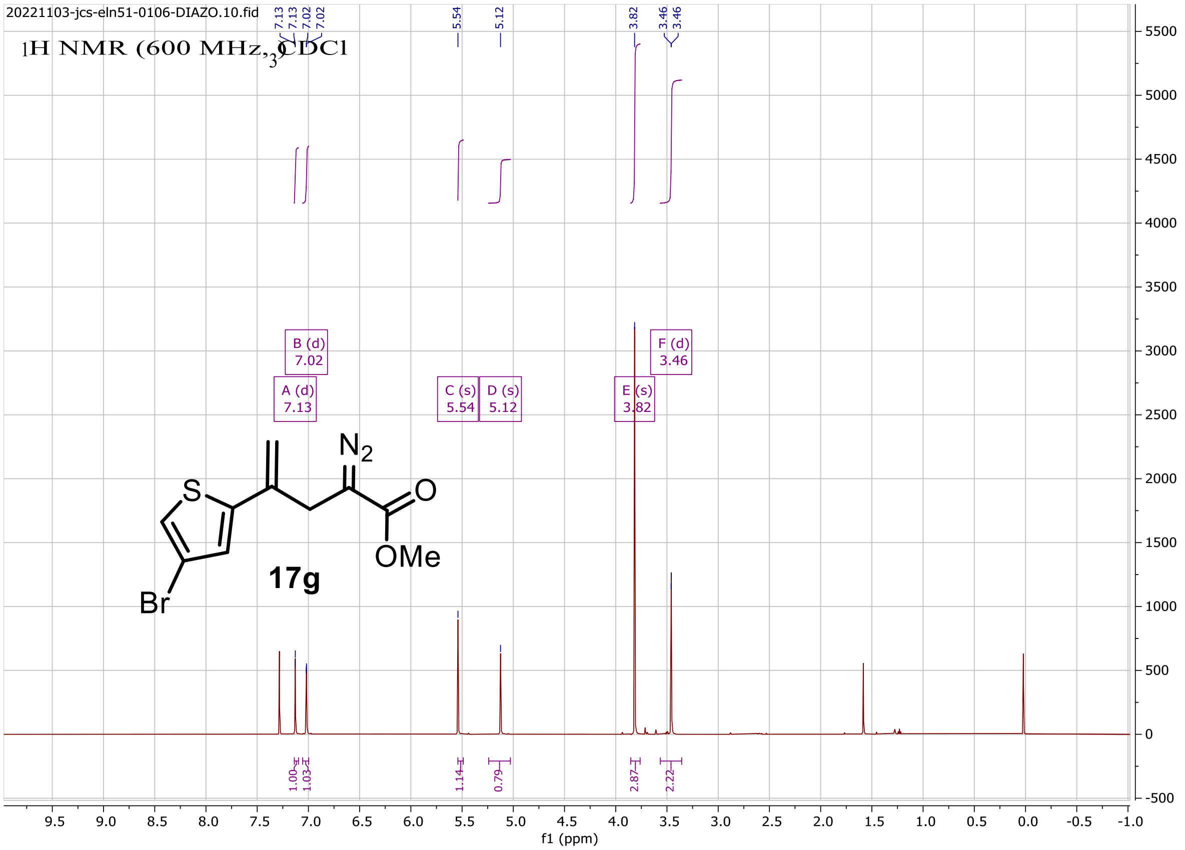

20221103-jcs-eln51-0106-DIAZO.10.fid
1H NMR (600 MHz, CDCl3)
7.13
7.13
7.02
7.02
5.54
5.12
3.82
3.46
3.46
A (d) 7.13
B (d) 7.02
C (s) 5.54
D (s) 5.12
E (s) 3.82
F (d) 3.46
S
Br
N2
O
OMe
17g
1.00
1.03
1.14
0.79
2.87
2.22
f1 (ppm)

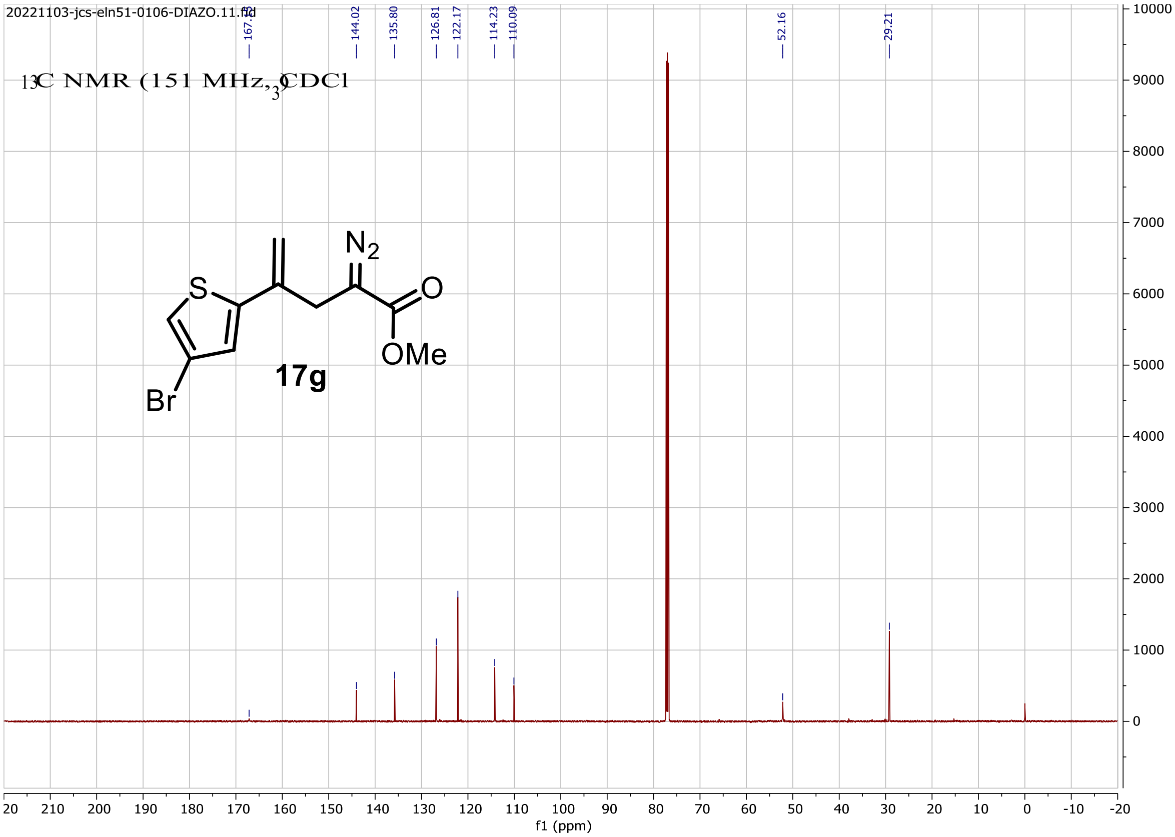
20221103-jcs-eln51-0106-DIAZO.11.fid
13C NMR (151 MHz, CDCl3)
167.42
144.02
135.80
126.81
122.17
114.23
110.09
52.16
29.21
S
N2
O
OMe
Br
17g
f1 (ppm)

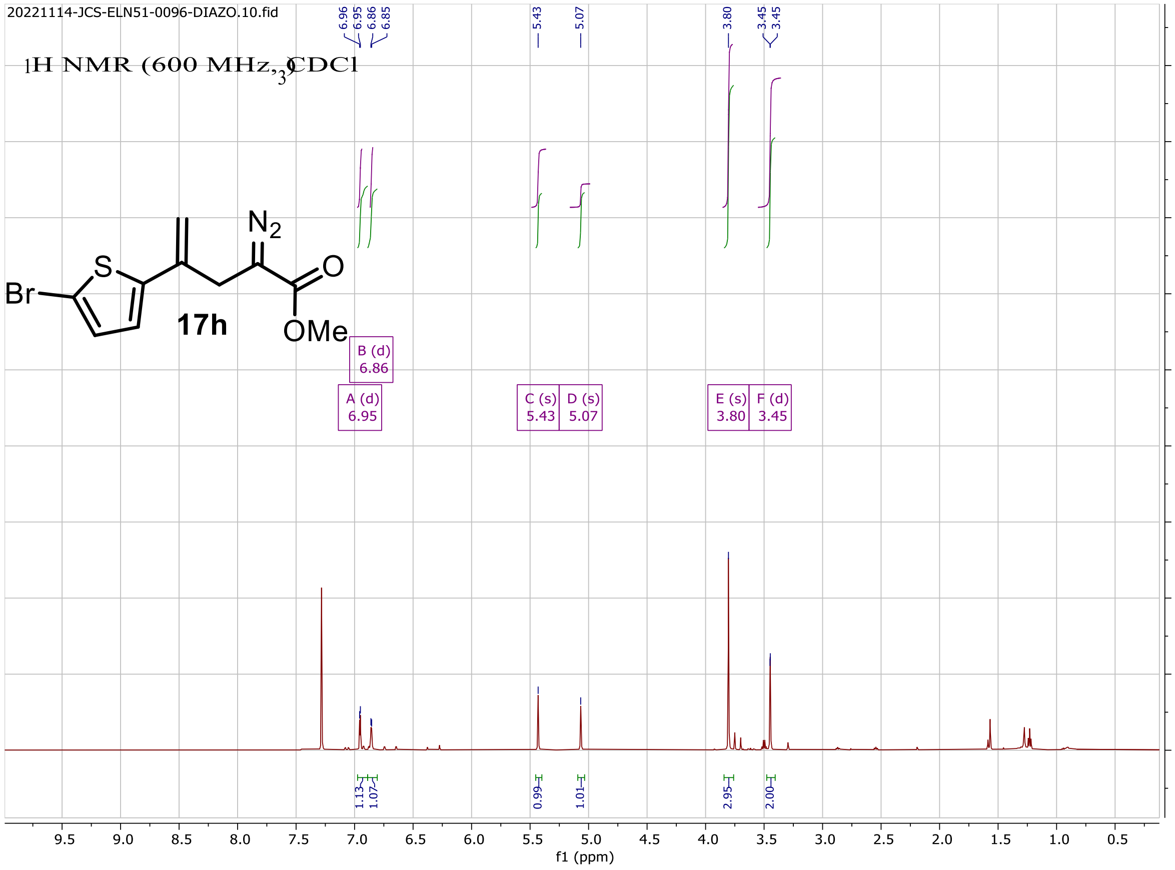
20221114-JCS-ELN51-0096-DIAZO.10.fid
1H NMR (600 MHz, CDCl3)
6.96
6.95
6.86
6.85
5.43
5.07
3.80
3.45
3.45
Br
S
N2
O
OMe
17h
B (d) 6.86
A (d) 6.95
C (s) 5.43
D (s) 5.07
E (s) 3.80
F (d) 3.45
1.13
1.07
0.99
1.01
2.95
2.00
f1 (ppm)

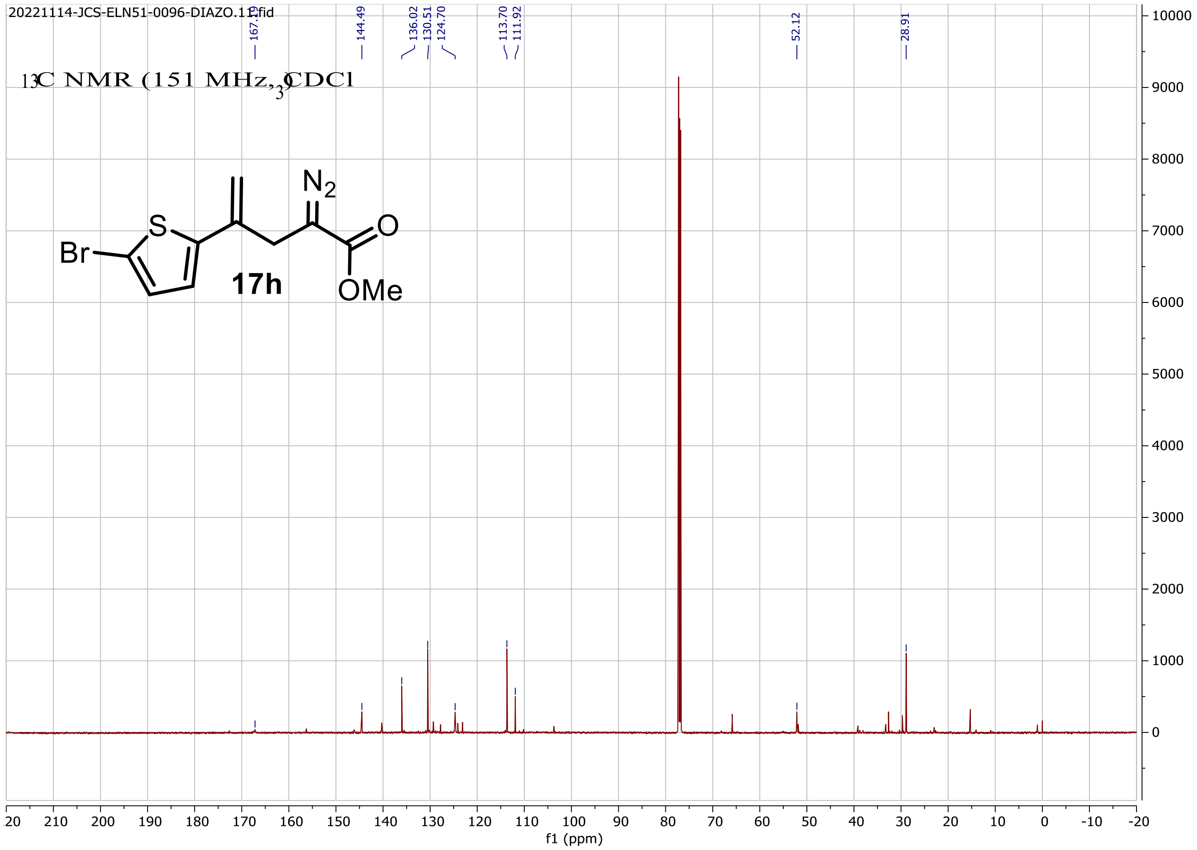
20221114-JCS-ELN51-0096-DIAZO.11.fid
144.49
136.02
130.51
124.70
113.70
111.92
52.12
28.91
13C NMR (151 MHz, CDCl3)
N2
S
O
Br
OMe
f1 (ppm)

17h

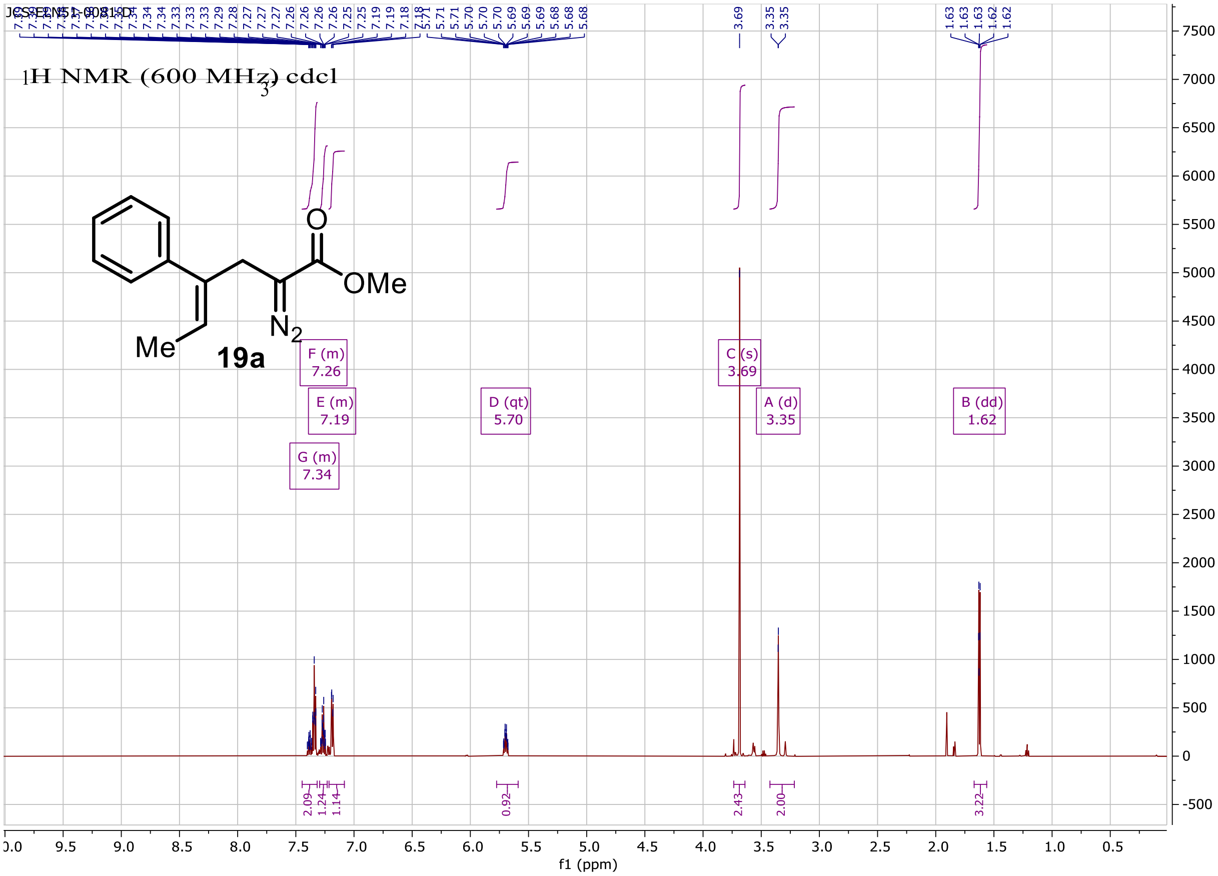
1H NMR (600 MHz) cdcl3
3.69
3.35
3.35
O
OMe
Me
N2
F (m) 7.26
E (m) 7.19
G (m) 7.34
D (qt) 5.70
C (s) 3.69
A (d) 3.35
B (dd) 1.62
2.09
1.24
1.14
0.92
2.43
2.00
3.22
f1 (ppm)

19a

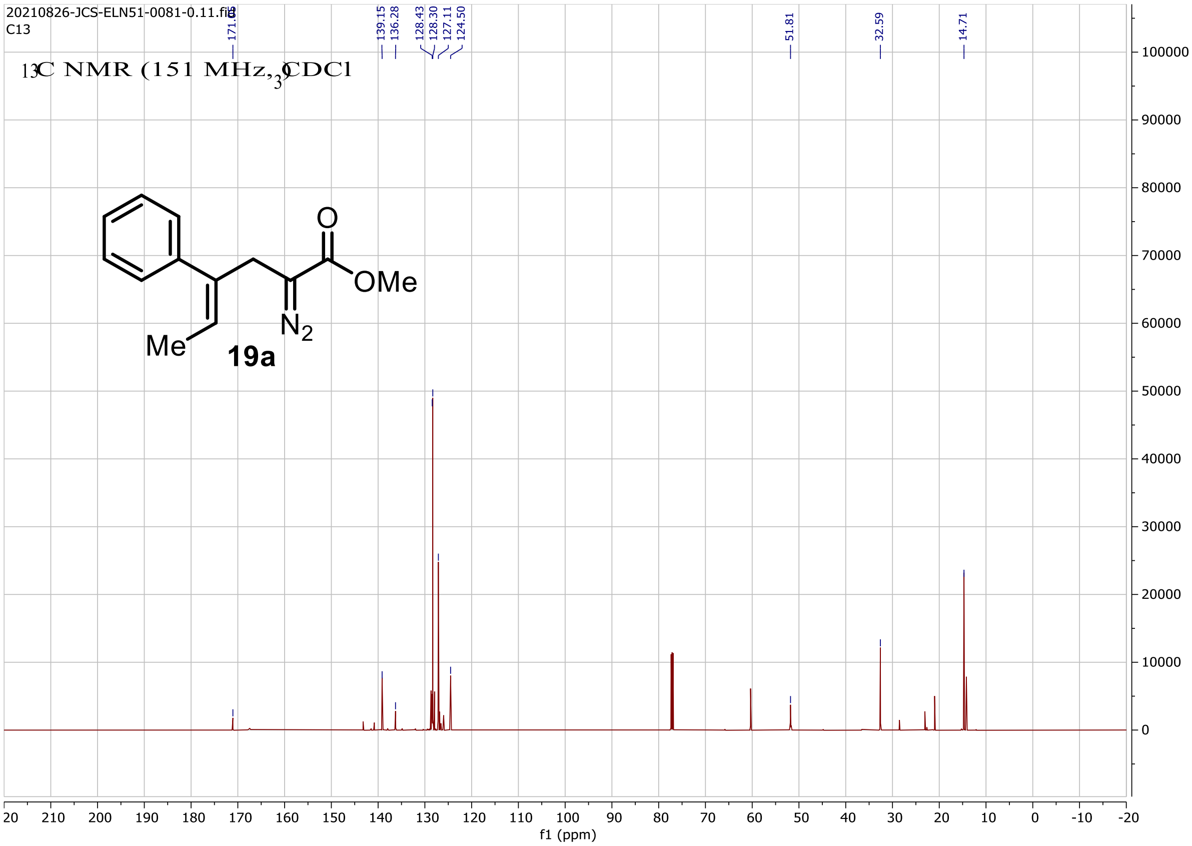
20210826-JCS-ELN51-0081-0.11.fid
C13
13C NMR (151 MHz, CDCl3)
171.62
139.15
136.28
128.43
128.30
127.11
124.50
51.81
32.59
14.71
O
OMe
N2
Me
19a
f1 (ppm)

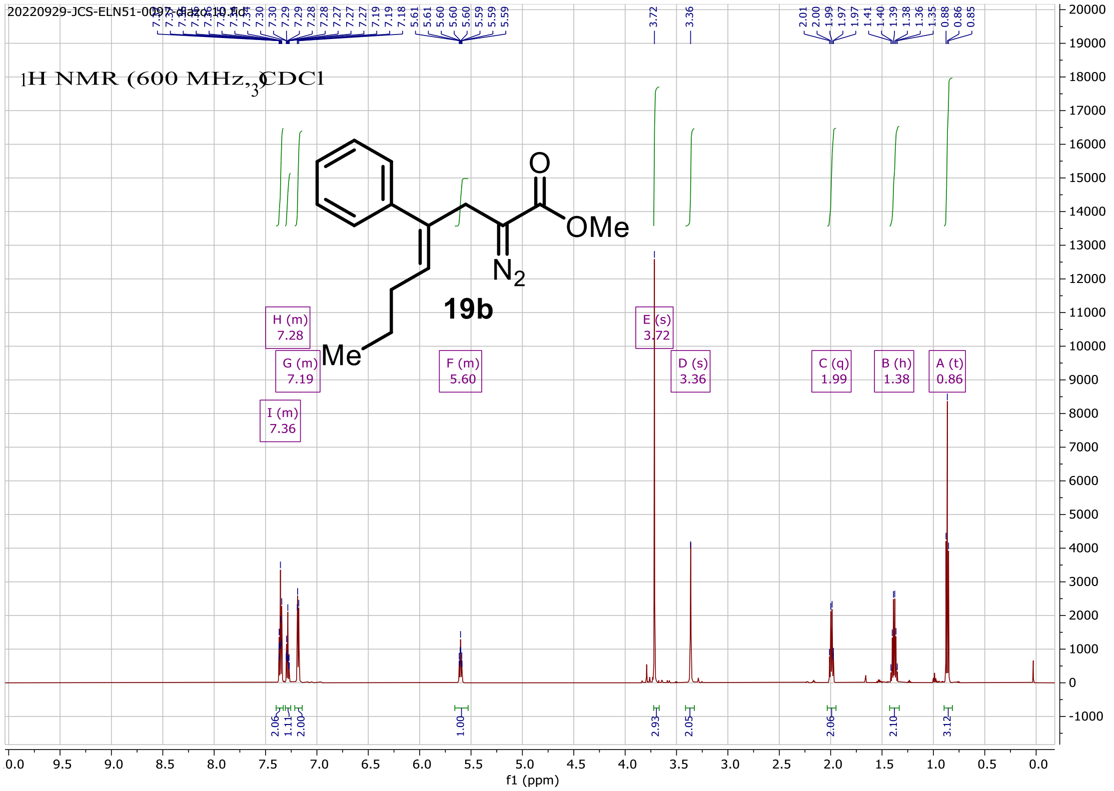
1H NMR (600 MHz, CDCl3)
O
OMe
N2
Me
19b
H (m) 7.28
G (m) 7.19
I (m) 7.36
F (m) 5.60
E (s) 3.72
D (s) 3.36
C (q) 1.99
B (h) 1.38
A (t) 0.86
f1 (ppm)

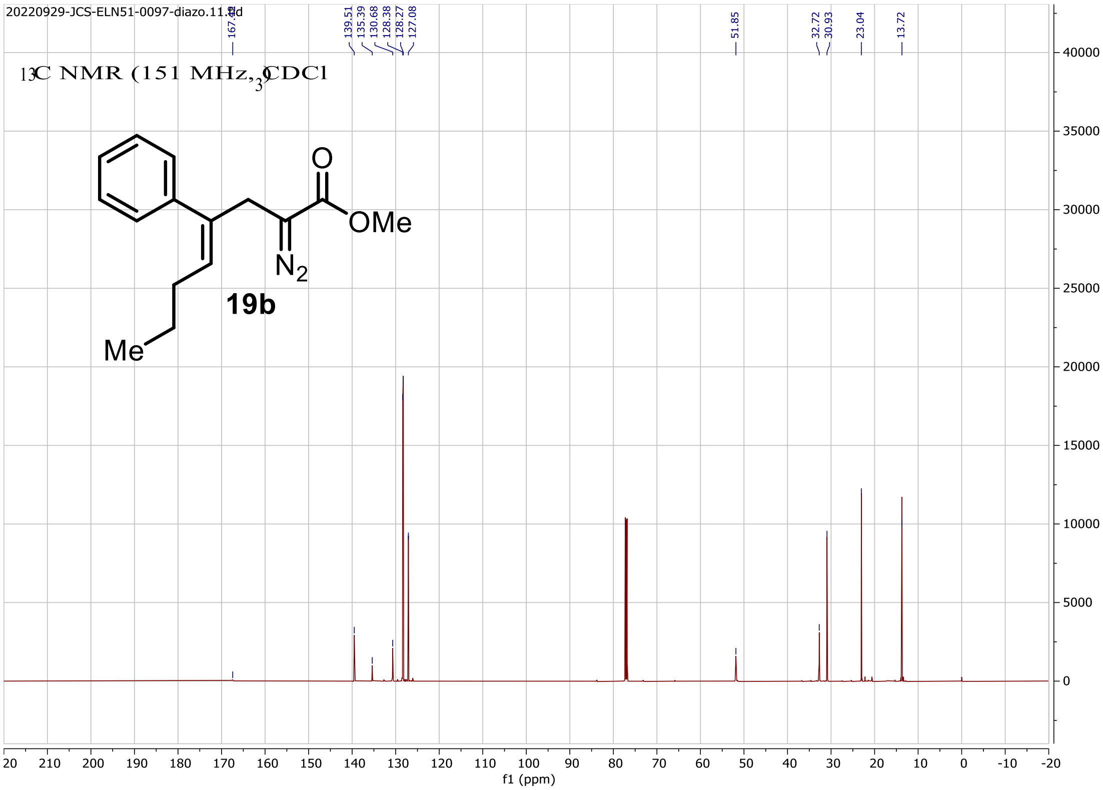
20220929-JCS-ELN51-0097-diazo.11.fid
13C NMR (151 MHz, CDCl3)
167.40
139.51
135.39
130.68
128.38
128.27
127.08
51.85
32.72
30.93
23.04
13.72
O
OMe
N2
Me
19b
f1 (ppm)

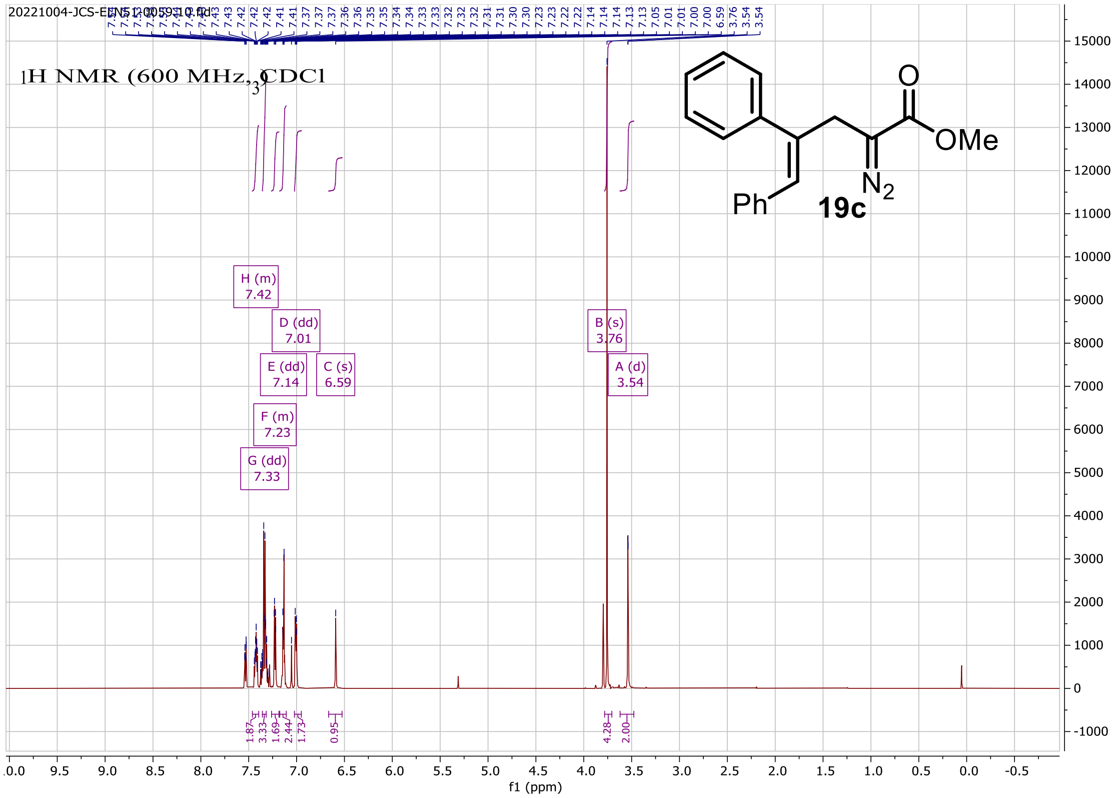
1H NMR (600 MHz, CDCl3)
H (m) 7.42
D (dd) 7.01
E (dd) 7.14
C (s) 6.59
F (m) 7.23
G (dd) 7.33
B (s) 3.76
A (d) 3.54
1.87
3.33
1.69
2.44
1.73
0.95
4.28
2.00
O
OMe
N2
Ph
19c
f1 (ppm)

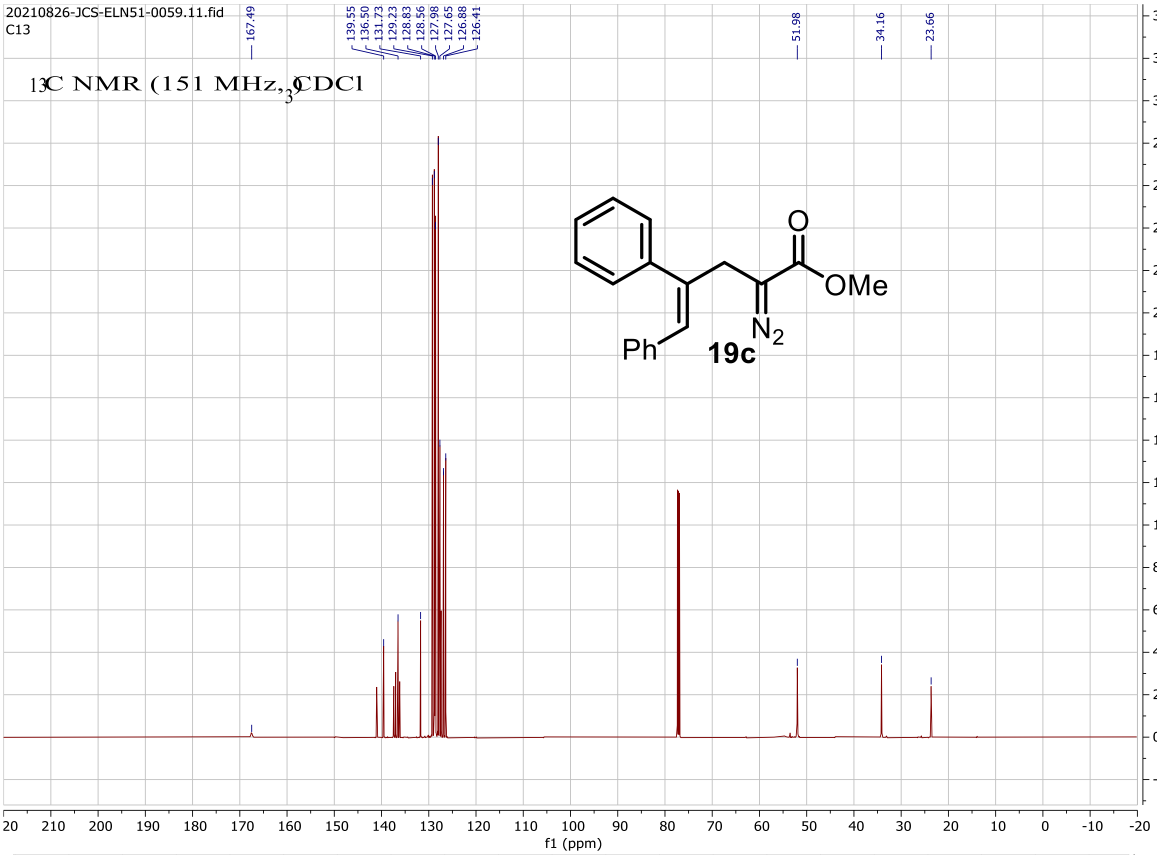
20210826-JCS-ELN51-0059.11.fid
C13
13C NMR (151 MHz, CDCl3)
167.49
139.55
136.50
131.73
129.23
128.83
128.56
127.98
127.65
126.88
126.41
51.98
34.16
23.66
OMe
O
N2
Ph
19c
f1 (ppm)

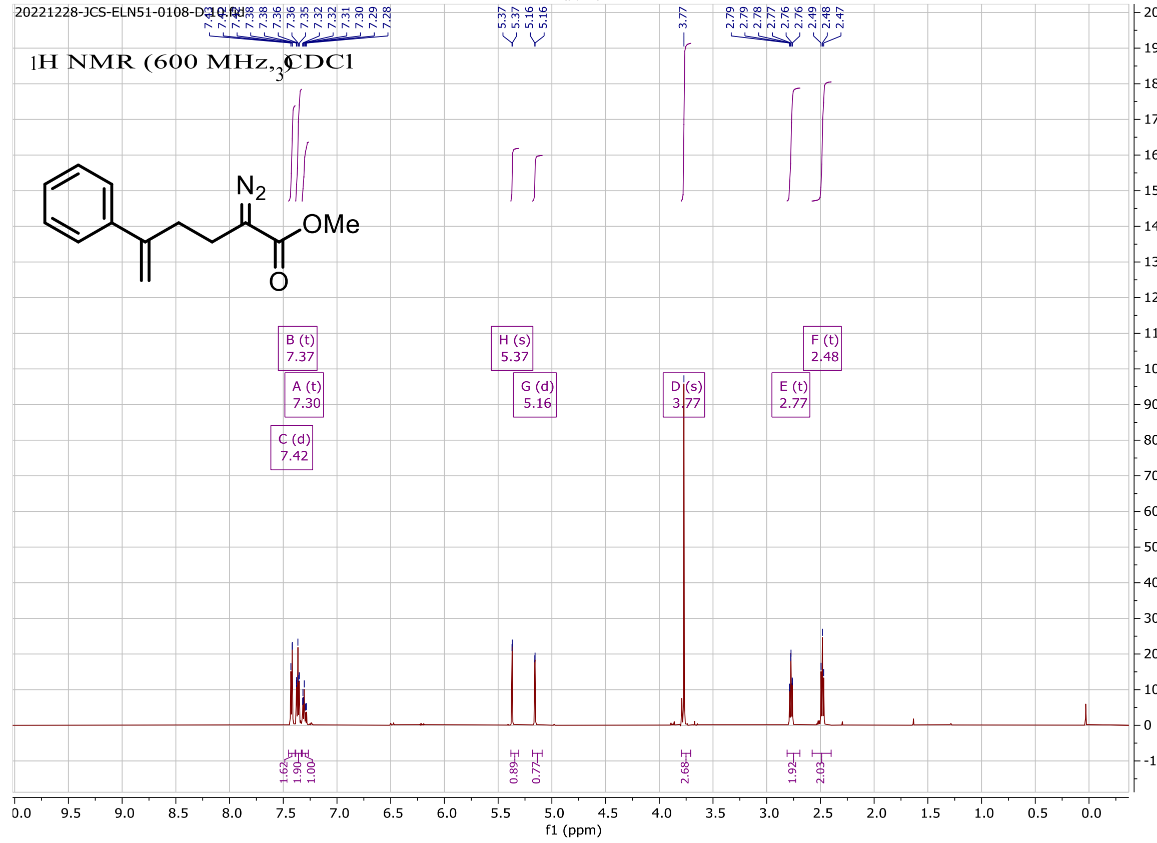
20221228-JCS-ELN51-0108-D10.fid
1H NMR (600 MHz, CDCl3)
N2
OMe
O
B (t)
7.37
A (t)
7.30
C (d)
7.42
H (s)
5.37
G (d)
5.16
D (s)
3.77
E (t)
2.77
F (t)
2.48
1.62
1.90
1.00
0.89
0.77
2.68
1.92
2.03
f1 (ppm)

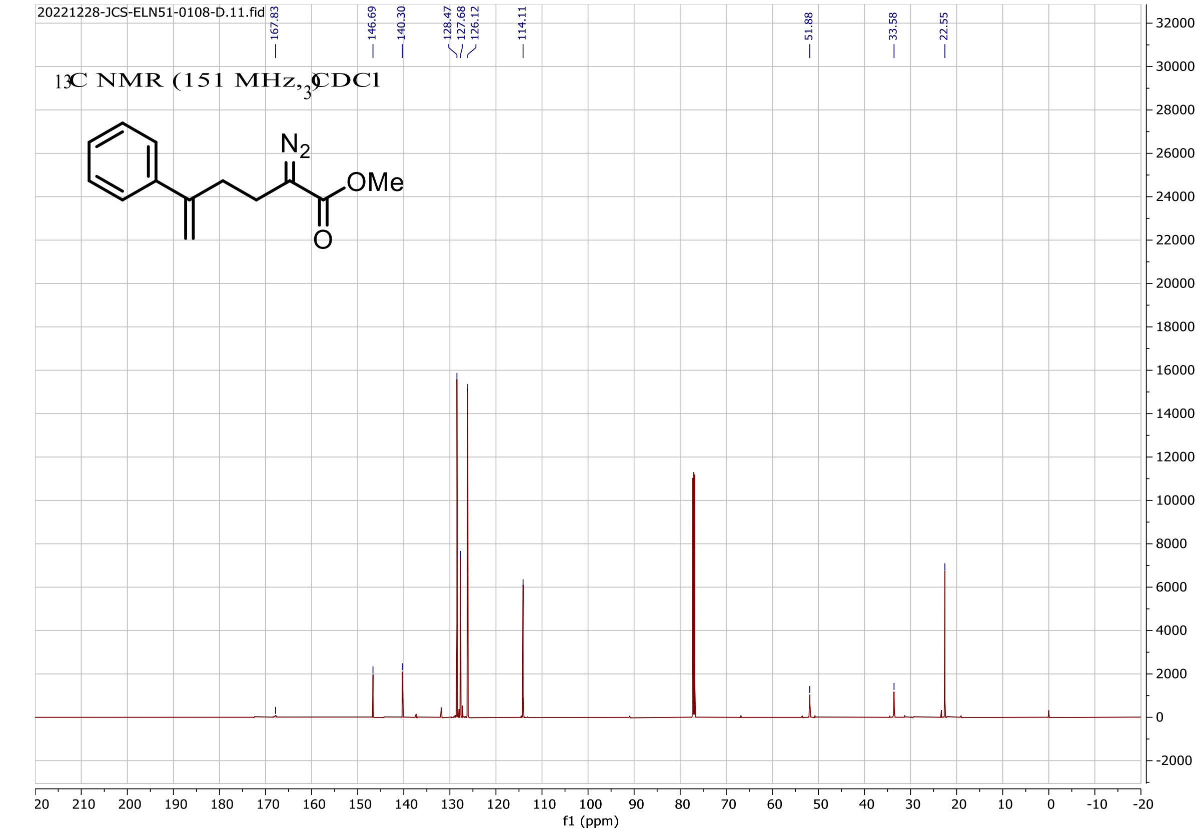
20221228-JCS-ELN51-0108-D.11.fid
167.83
146.69
140.30
128.47
127.68
126.12
114.11
51.88
33.58
22.55
13C NMR (151 MHz, CDCl3)
N2
OMe
O
f1 (ppm)

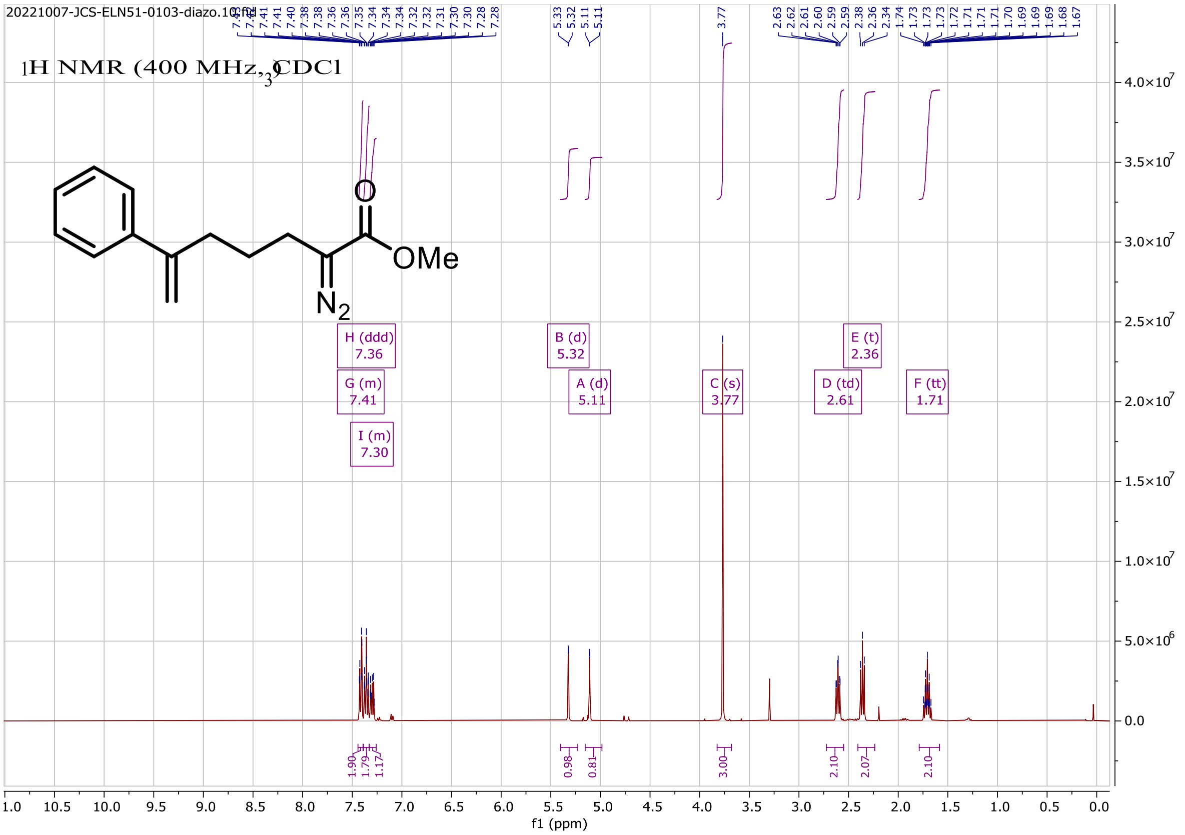
20221007-JCS-ELN51-0103-diazo.10.fid
1H NMR (400 MHz, CDCl3)
O
OMe
N2
H (ddd) 7.36
G (m) 7.41
I (m) 7.30
B (d) 5.32
A (d) 5.11
C (s) 3.77
D (td) 2.61
E (t) 2.36
F (tt) 1.71
1.90
1.79
1.17
0.98
0.81
3.00
2.10
2.07
2.10
f1 (ppm)

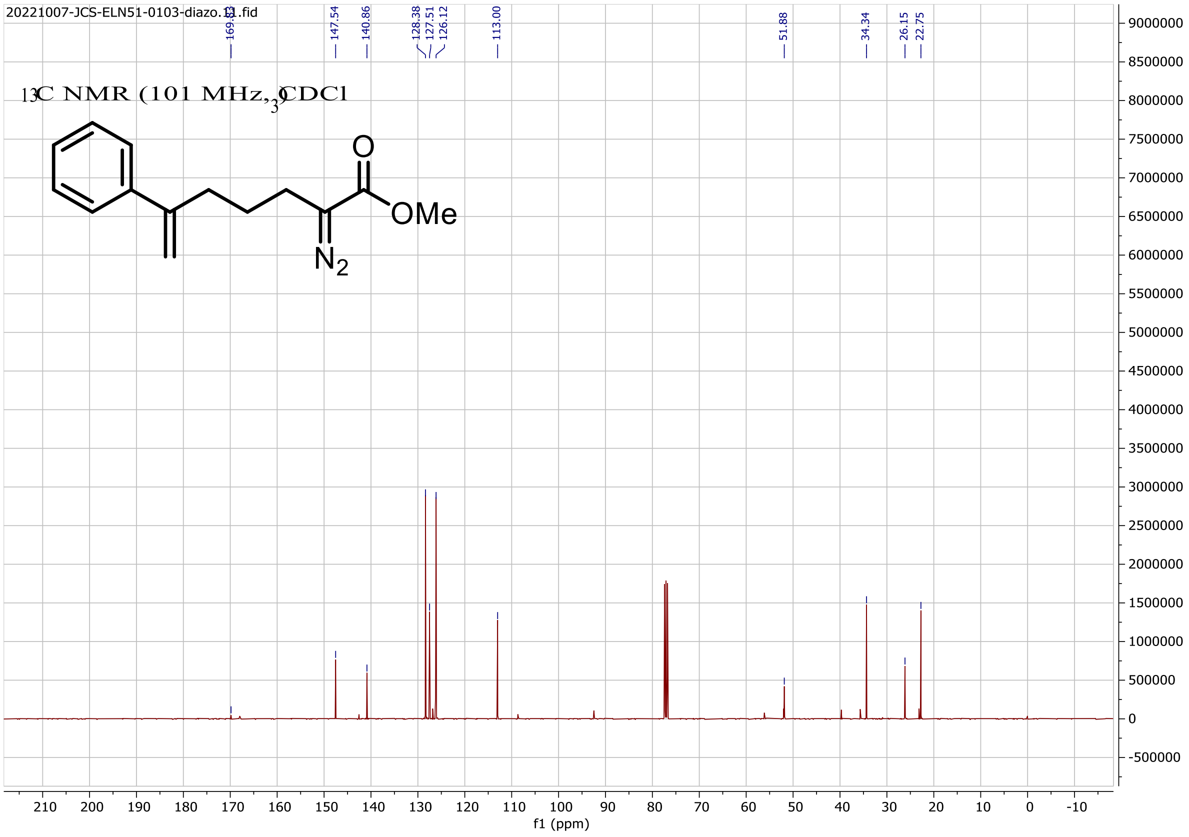
20221007-JCS-ELN51-0103-diazo.11.fid
13C NMR (101 MHz, CDCl3)
OMe
N2
O
169.1[illegible]
147.54
140.86
128.38
127.51
126.12
113.00
51.88
34.34
26.15
22.75
f1 (ppm)

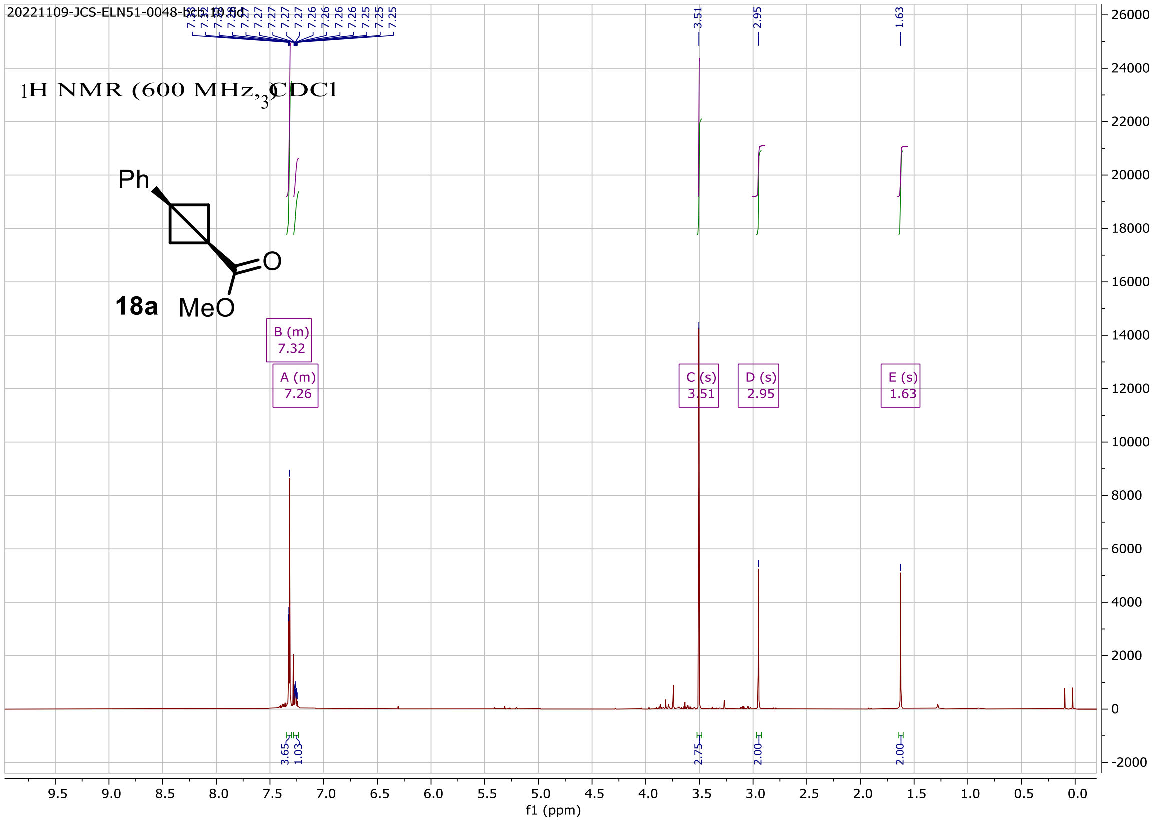
1H NMR (600 MHz, CDCl3)
Ph
O
MeO
18a
B (m) 7.32
A (m) 7.26
C (s) 3.51
D (s) 2.95
E (s) 1.63
3.51
2.95
1.63
3.65
1.03
2.75
2.00
2.00
f1 (ppm)

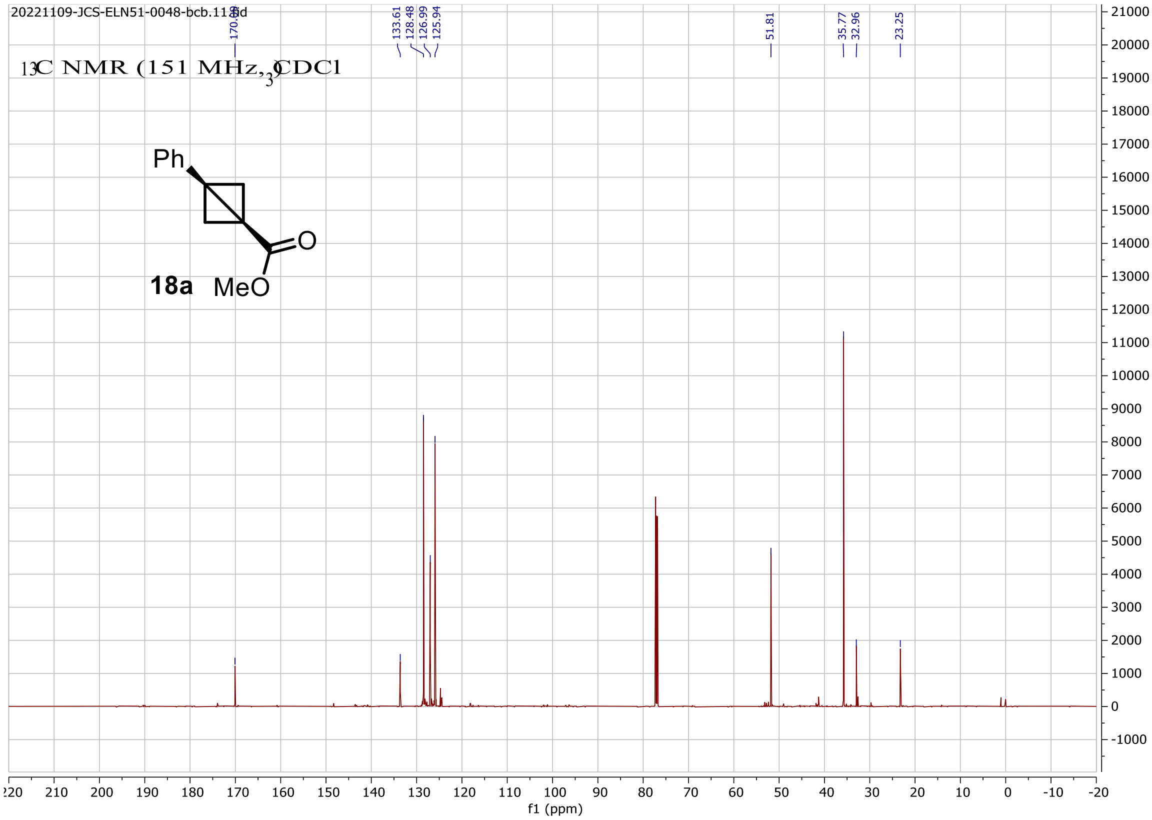
20221109-JCS-ELN51-0048-bcb.11.fid
13C NMR (151 MHz, CDCl3)
Ph
O
18a
MeO
170.04
133.61
128.48
126.99
125.94
51.81
35.77
32.96
23.25
f1 (ppm)

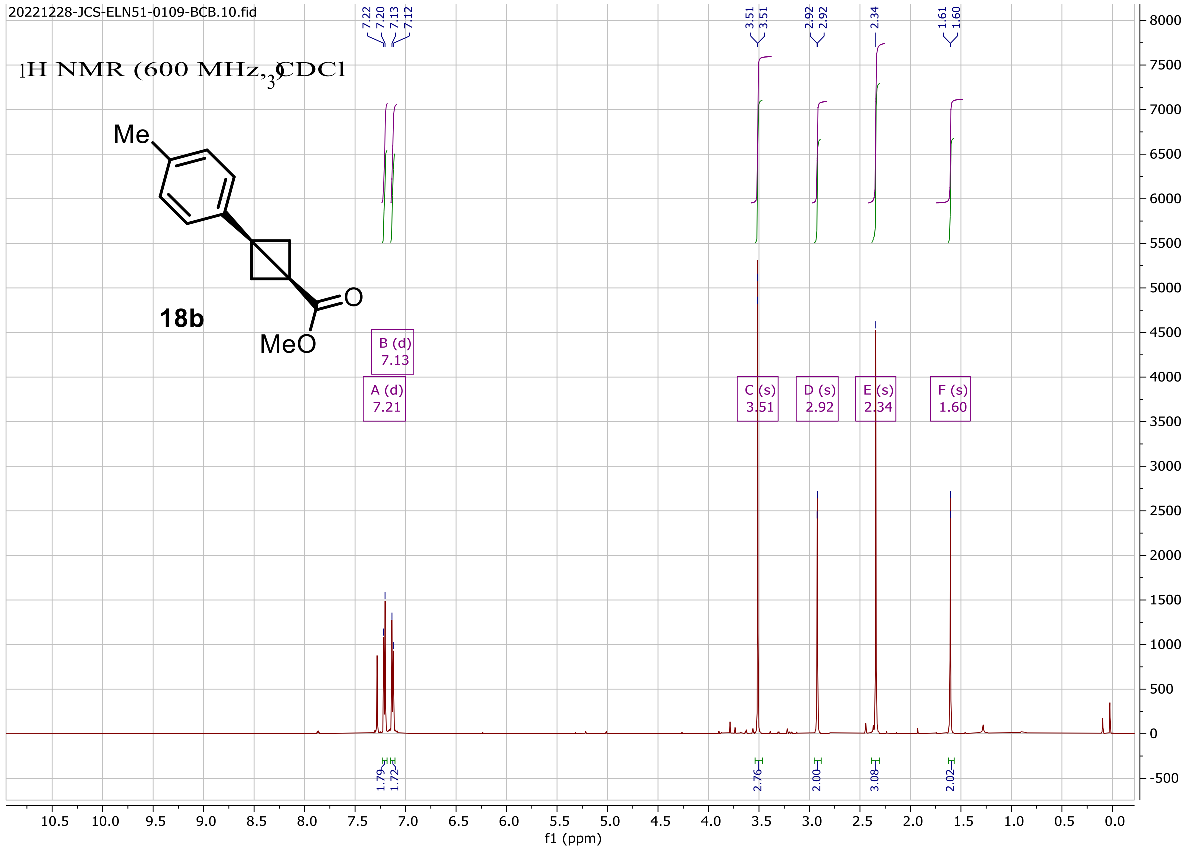
20221228-JCS-ELN51-0109-BCB.10.fid
1H NMR (600 MHz, CDCl3)
Me
O
18b
MeO
7.22
7.20
7.13
7.12
3.51
3.51
2.92
2.92
2.34
1.61
1.60
B (d) 7.13
A (d) 7.21
C (s) 3.51
D (s) 2.92
E (s) 2.34
F (s) 1.60
1.79
1.72
2.76
2.00
3.08
2.02
f1 (ppm)

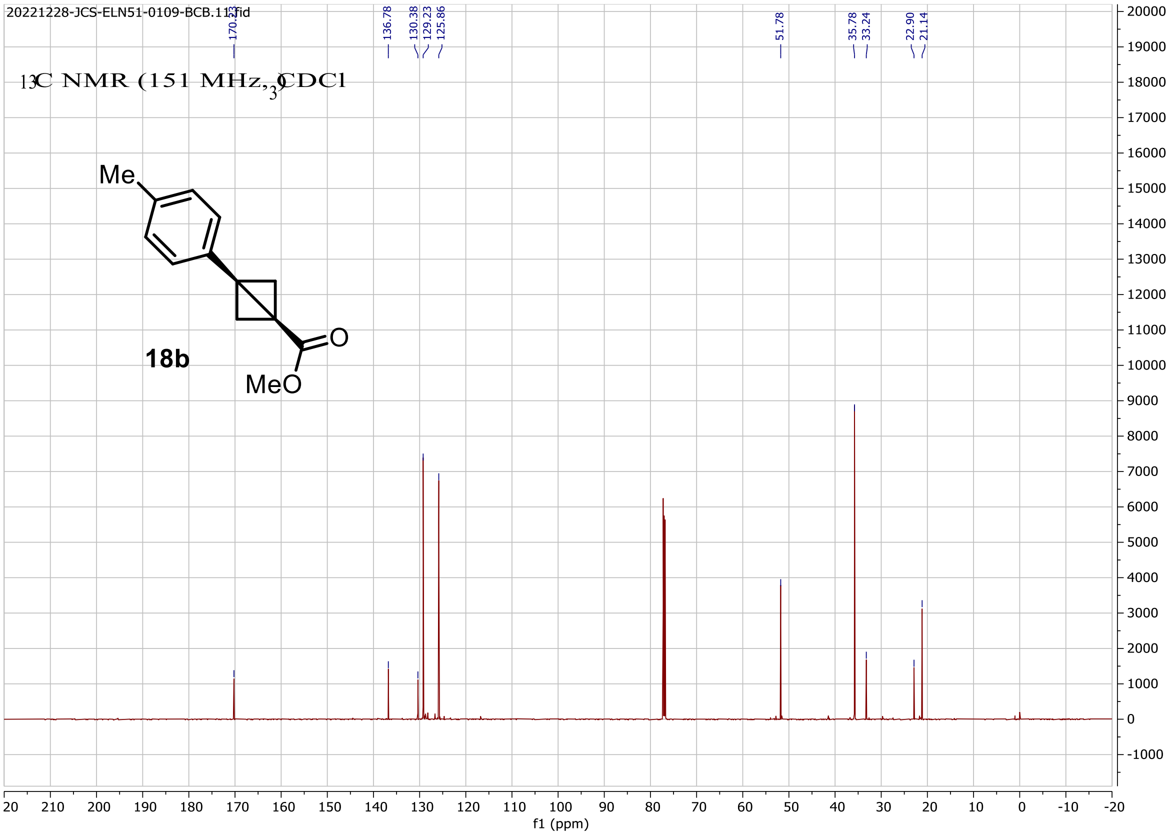
20221228-JCS-ELN51-0109-BCB.11.fid
13C NMR (151 MHz, CDCl3)
Me
18b
MeO
O
170.23
136.78
130.38
129.23
125.86
51.78
35.78
33.24
22.90
21.14
f1 (ppm)

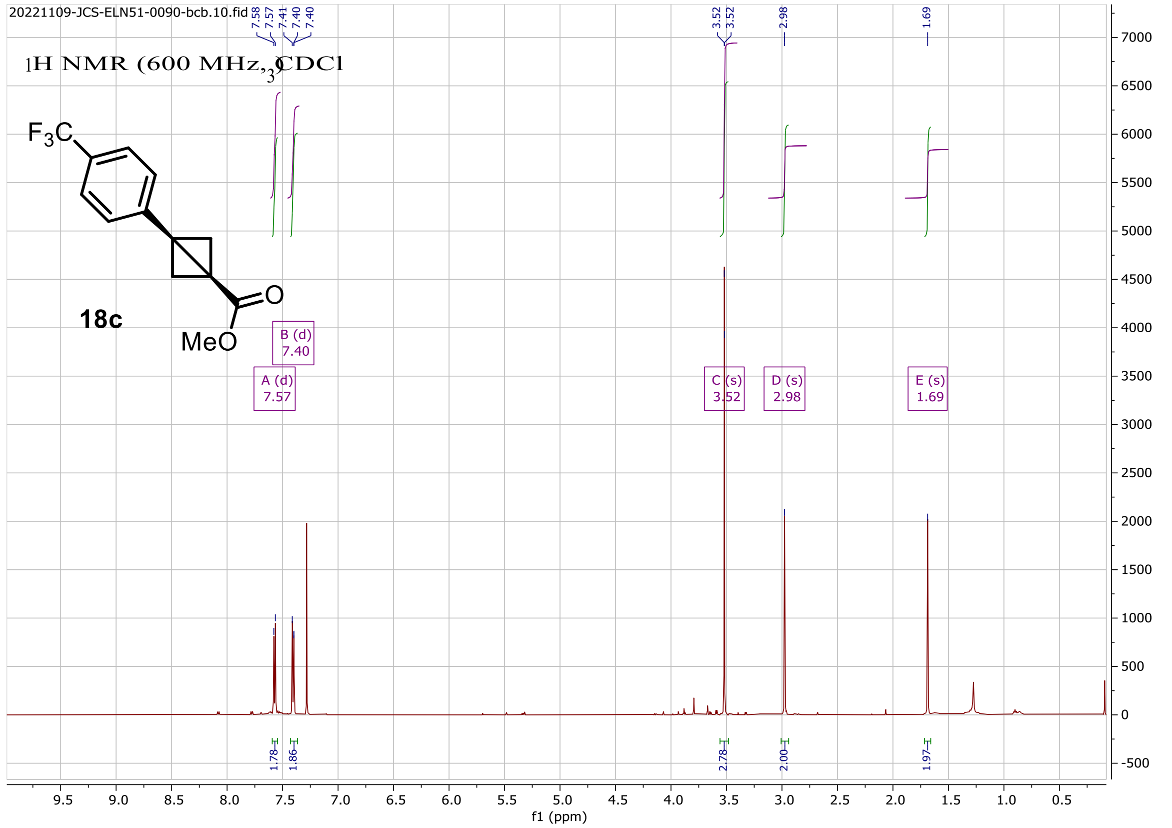
20221109-JCS-ELN51-0090-bcb.10.fid
1H NMR (600 MHz, CDCl3)
F3C
18c
MeO
O
7.58
7.57
7.41
7.40
7.40
3.52
3.52
2.98
1.69
B (d)
7.40
A (d)
7.57
C (s)
3.52
D (s)
2.98
E (s)
1.69
1.78
1.86
2.78
2.00
1.97
f1 (ppm)

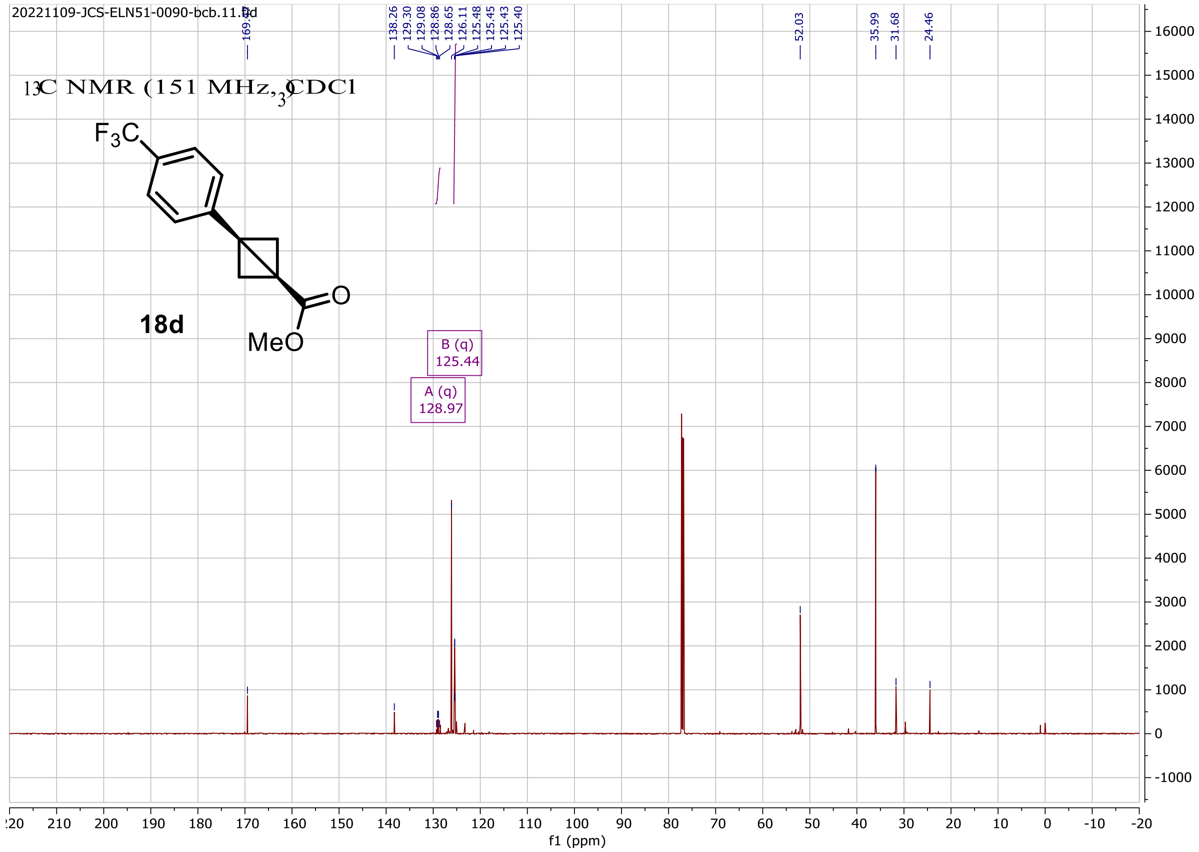

20221109-JCS-ELN51-0090-bcb.11.fid
13C NMR (151 MHz, CDCl3)
F3C
18d
MeO
O
169.9
138.26
129.30
129.08
128.86
128.65
126.11
125.48
125.45
125.43
125.40
52.03
35.99
31.68
24.46
B (q)
125.44
A (q)
128.97
f1 (ppm)

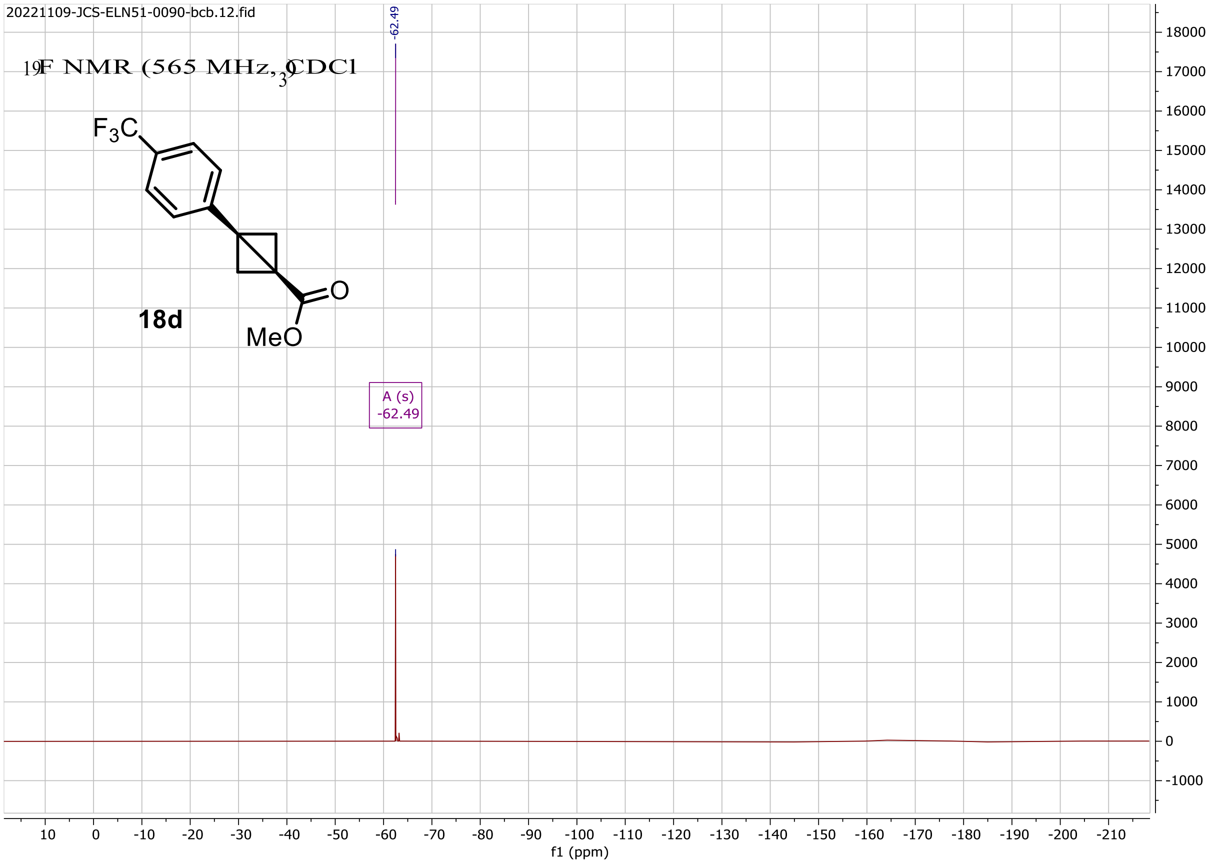

20221109-JCS-ELN51-0090-bcb.12.fid
19F NMR (565 MHz, CDCl3)
F3C
18d
MeO
O
-62.49
A (s)
-62.49
f1 (ppm)

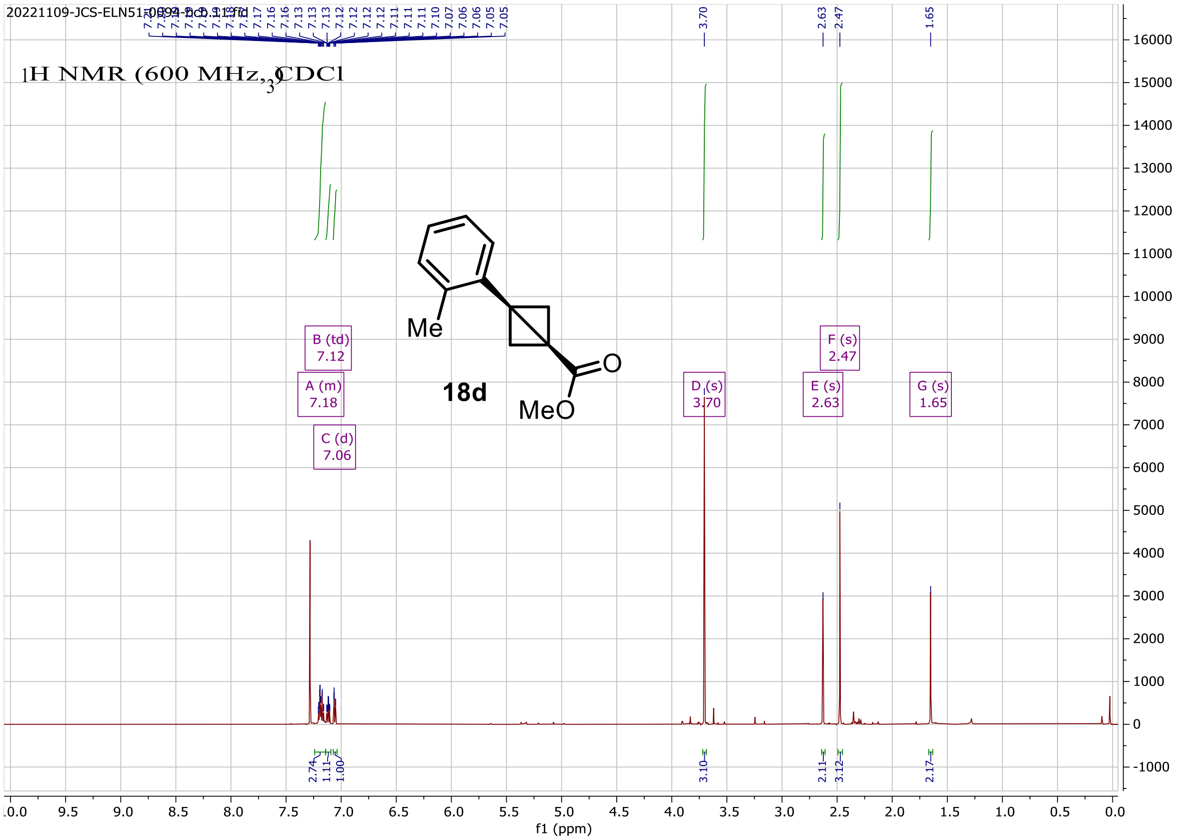
20221109-JCS-ELN51-0094-bcb.11.fid
1H NMR (600 MHz, CDCl3)
7.18
7.12
7.06
3.70
2.63
2.47
1.65
B (td)
7.12
A (m)
7.18
C (d)
7.06
D (s)
3.70
E (s)
2.63
F (s)
2.47
G (s)
1.65
Me
18d
MeO
O
2.74
1.11
1.00
3.10
2.11
3.12
2.17
f1 (ppm)

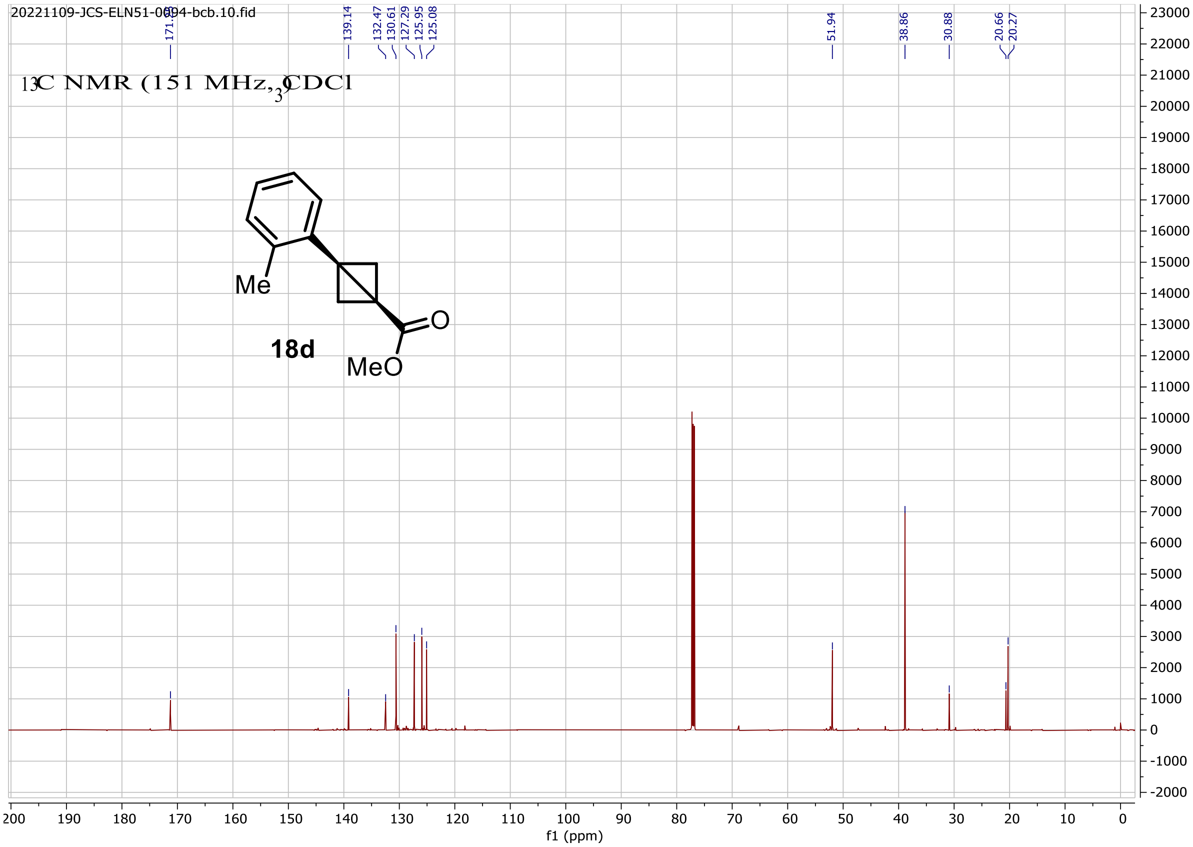
20221109-JCS-ELN51-0094-bcb.10.fid
13C NMR (151 MHz, CDCl3)
139.14
132.47
130.61
127.29
125.95
125.08
51.94
38.86
30.88
20.66
20.27
Me
18d
MeO
O
f1 (ppm)

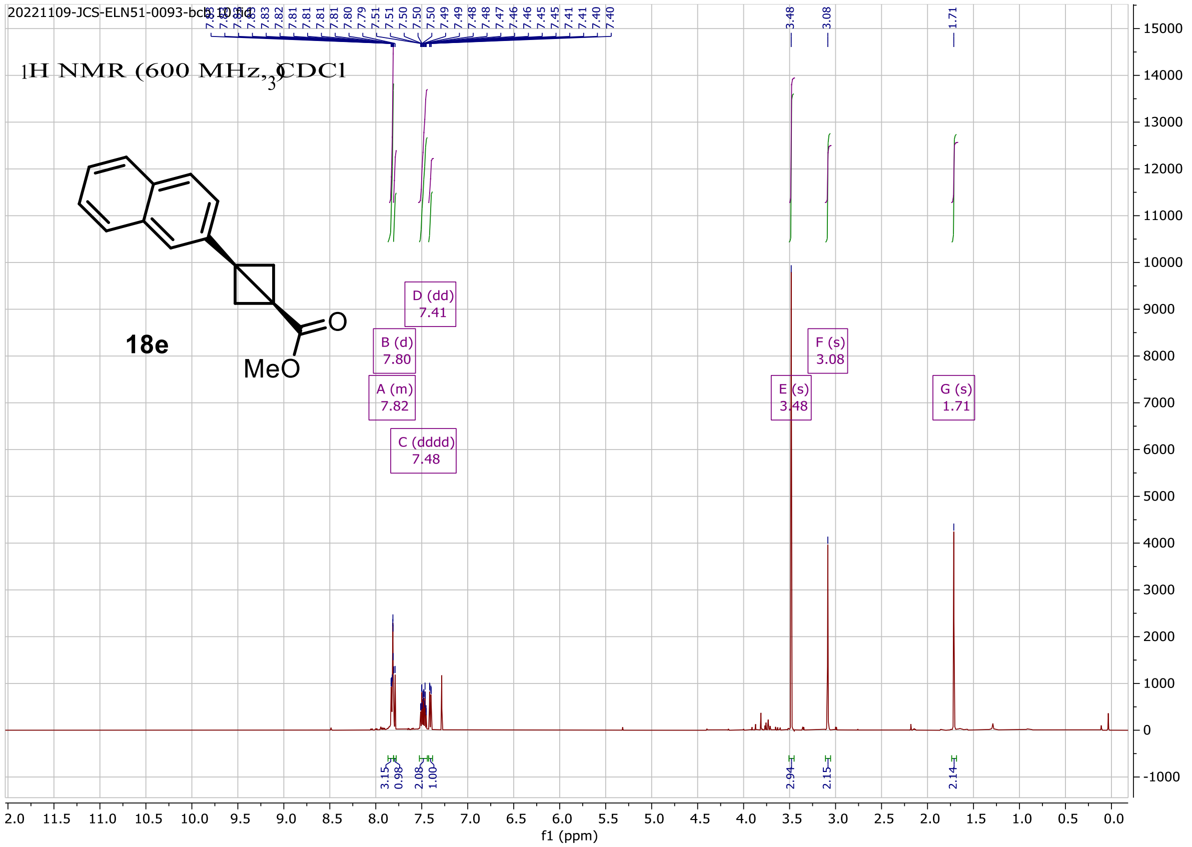
20221109-JCS-ELN51-0093-bcb.10.fid
1H NMR (600 MHz, CDCl3)
18e
MeO
O
A (m) 7.82
B (d) 7.80
C (dddd) 7.48
D (dd) 7.41
E (s) 3.48
F (s) 3.08
G (s) 1.71
3.48
3.08
1.71
3.15
0.98
2.08
1.00
2.94
2.15
2.14
f1 (ppm)

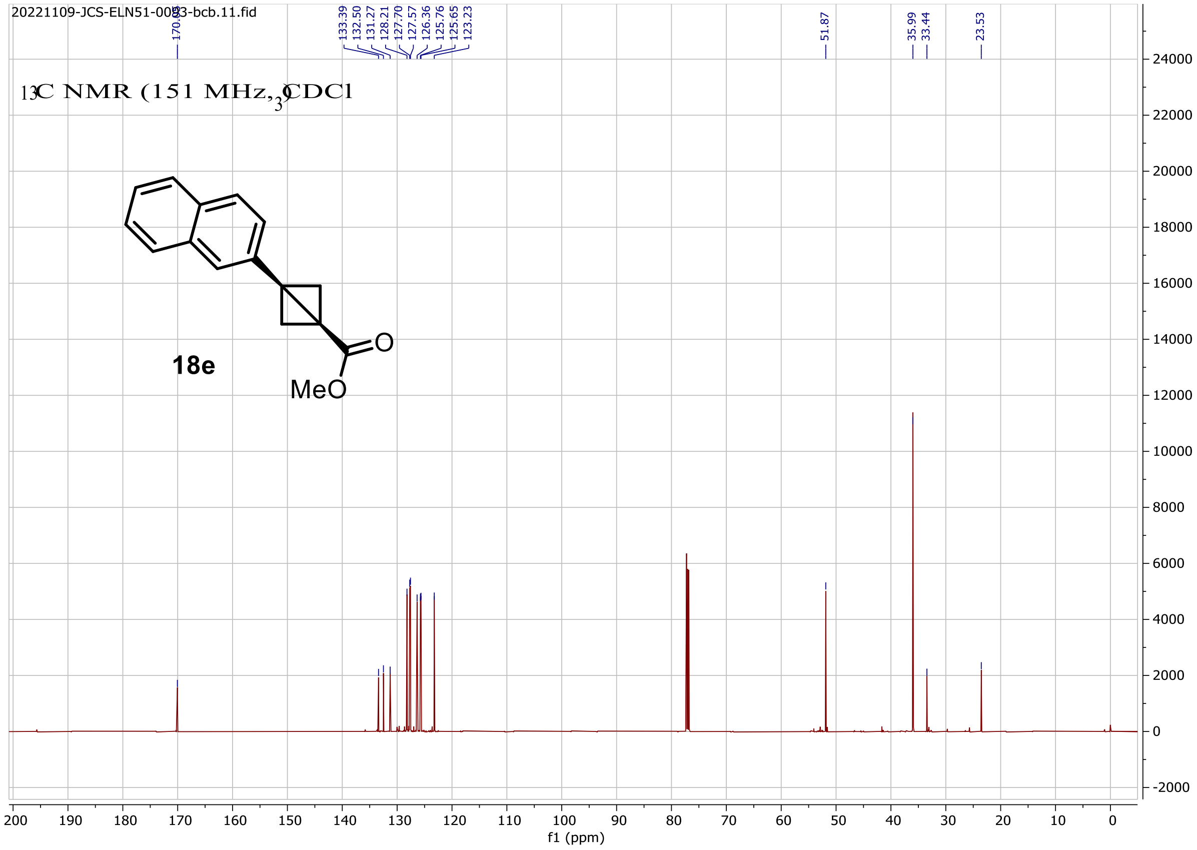
20221109-JCS-ELN51-0093-bcb.11.fid
13C NMR (151 MHz, CDCl3)
18e
MeO
O
133.39
132.50
131.27
128.21
127.70
127.57
126.36
125.76
125.65
123.23
51.87
35.99
33.44
23.53
f1 (ppm)

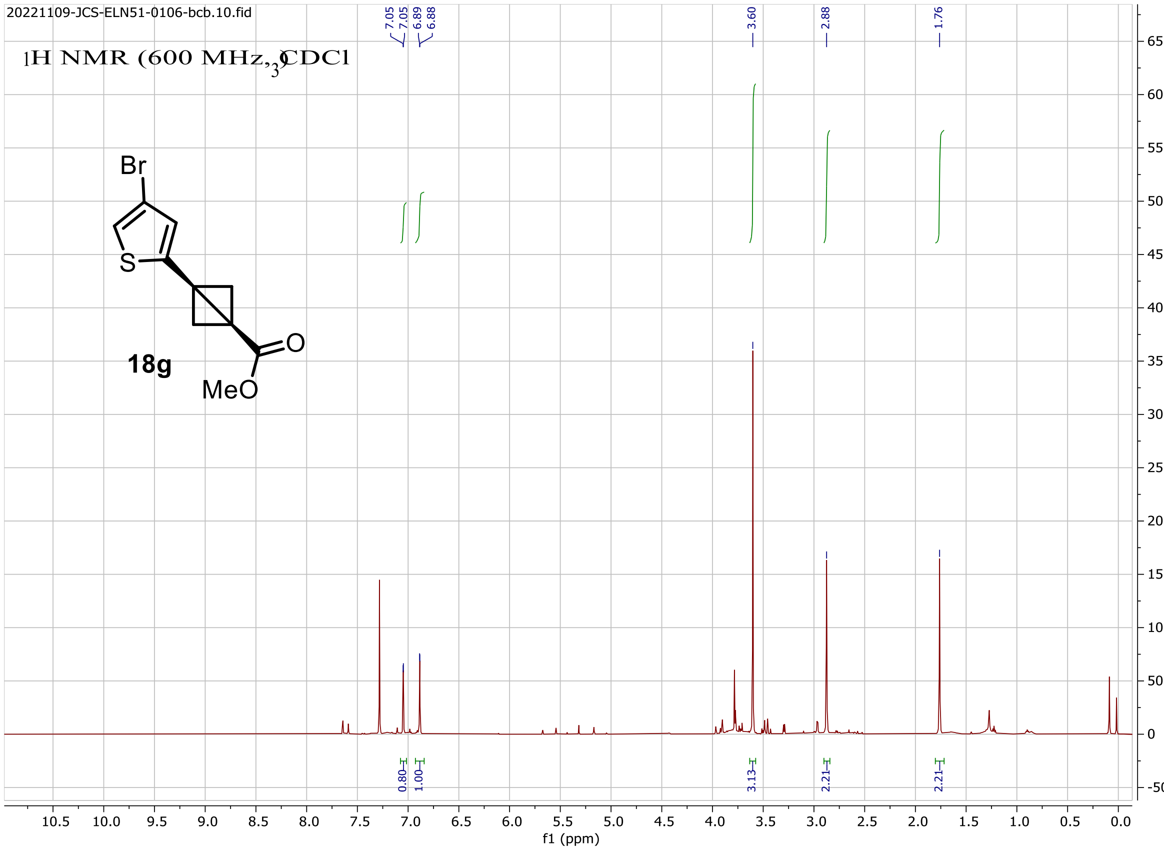
20221109-JCS-ELN51-0106-bcb.10.fid
1H NMR (600 MHz, CDCl3)
7.05
7.05
6.89
6.88
3.60
2.88
1.76
Br
S
18g
O
MeO
0.80
1.00
3.13
2.21
2.21
f1 (ppm)

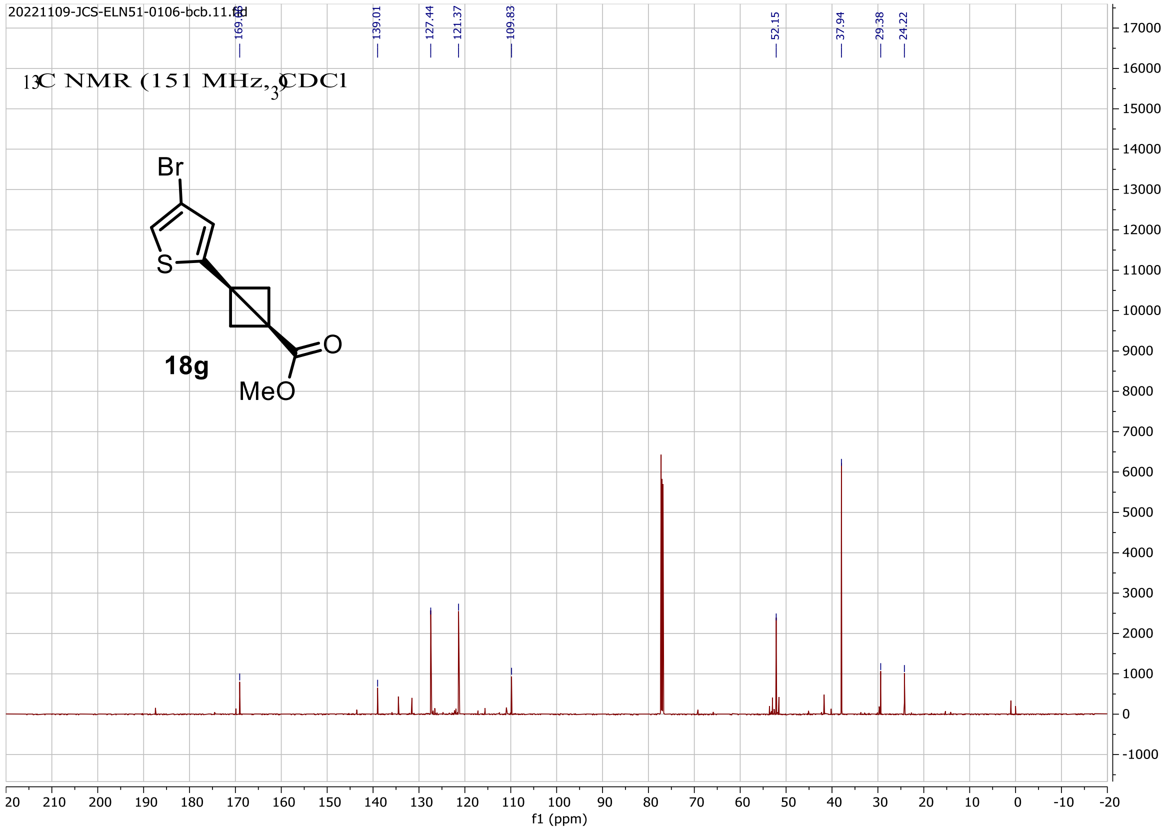
20221109-JCS-ELN51-0106-bcb.11.fid
13C NMR (151 MHz, CDCl3)
139.01
127.44
121.37
109.83
52.15
37.94
29.38
24.22
Br
S
18g
O
MeO
f1 (ppm)

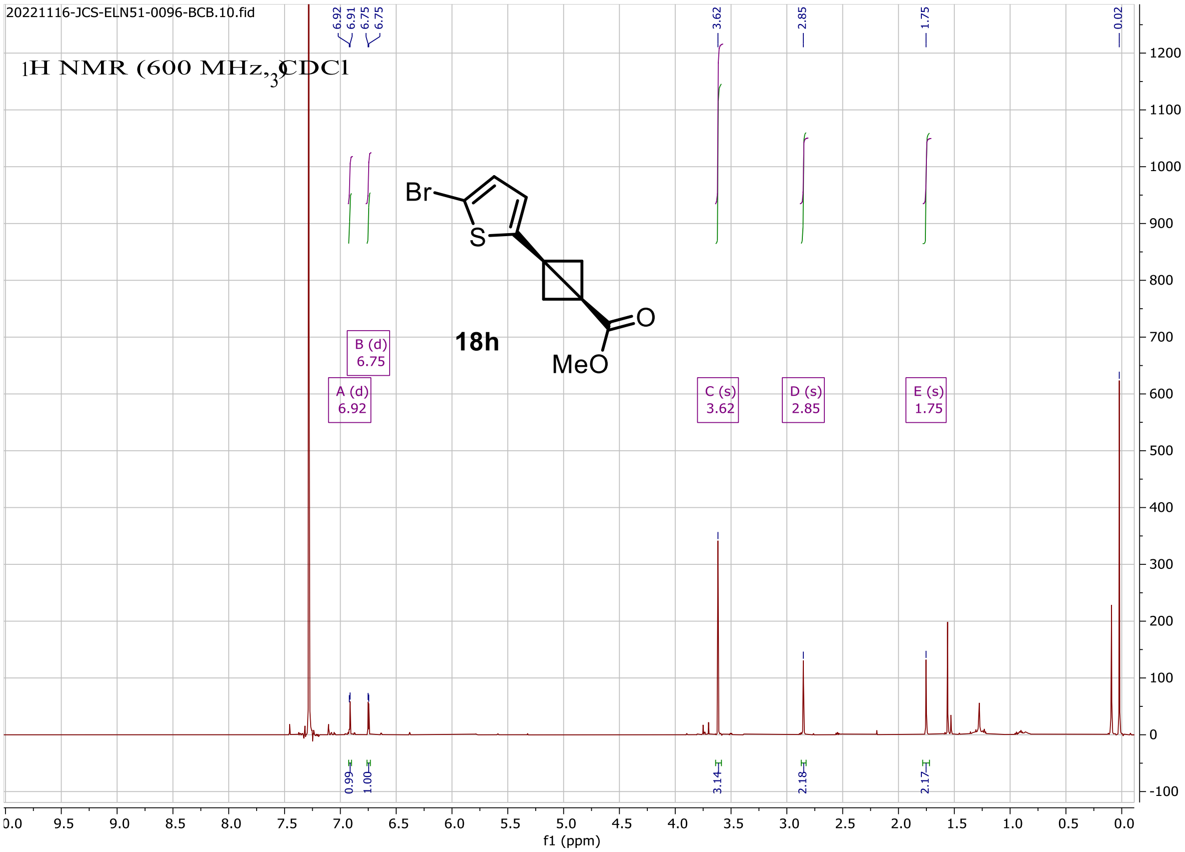
20221116-JCS-ELN51-0096-BCB.10.fid
1H NMR (600 MHz, CDCl3)
6.92
6.91
6.75
6.75
3.62
2.85
1.75
0.02
Br
S
MeO
O
18h
A (d)
6.92
B (d)
6.75
C (s)
3.62
D (s)
2.85
E (s)
1.75
0.99
1.00
3.14
2.18
2.17
f1 (ppm)

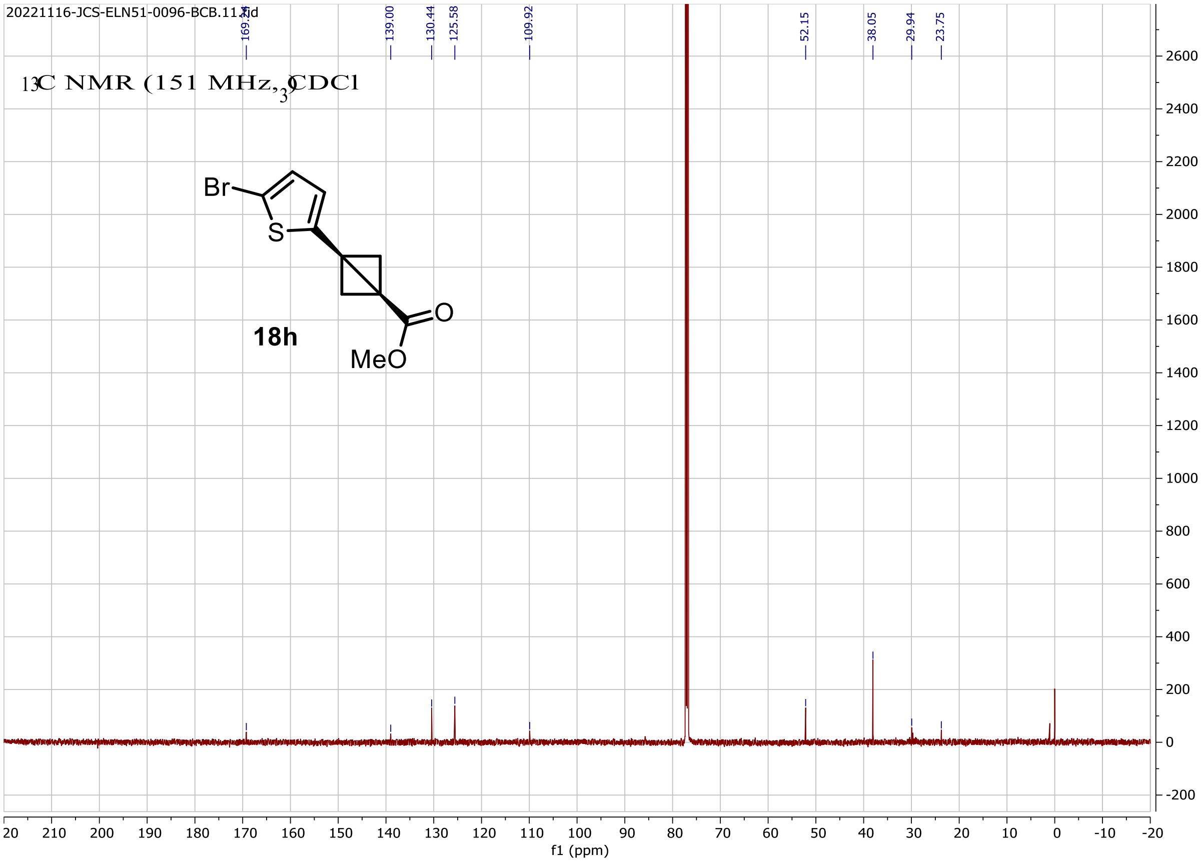
20221116-JCS-ELN51-0096-BCB.11.fid
13C NMR (151 MHz, CDCl3)
139.00
130.44
125.58
109.92
52.15
38.05
29.94
23.75
Br
S
MeO
O
18h
f1 (ppm)

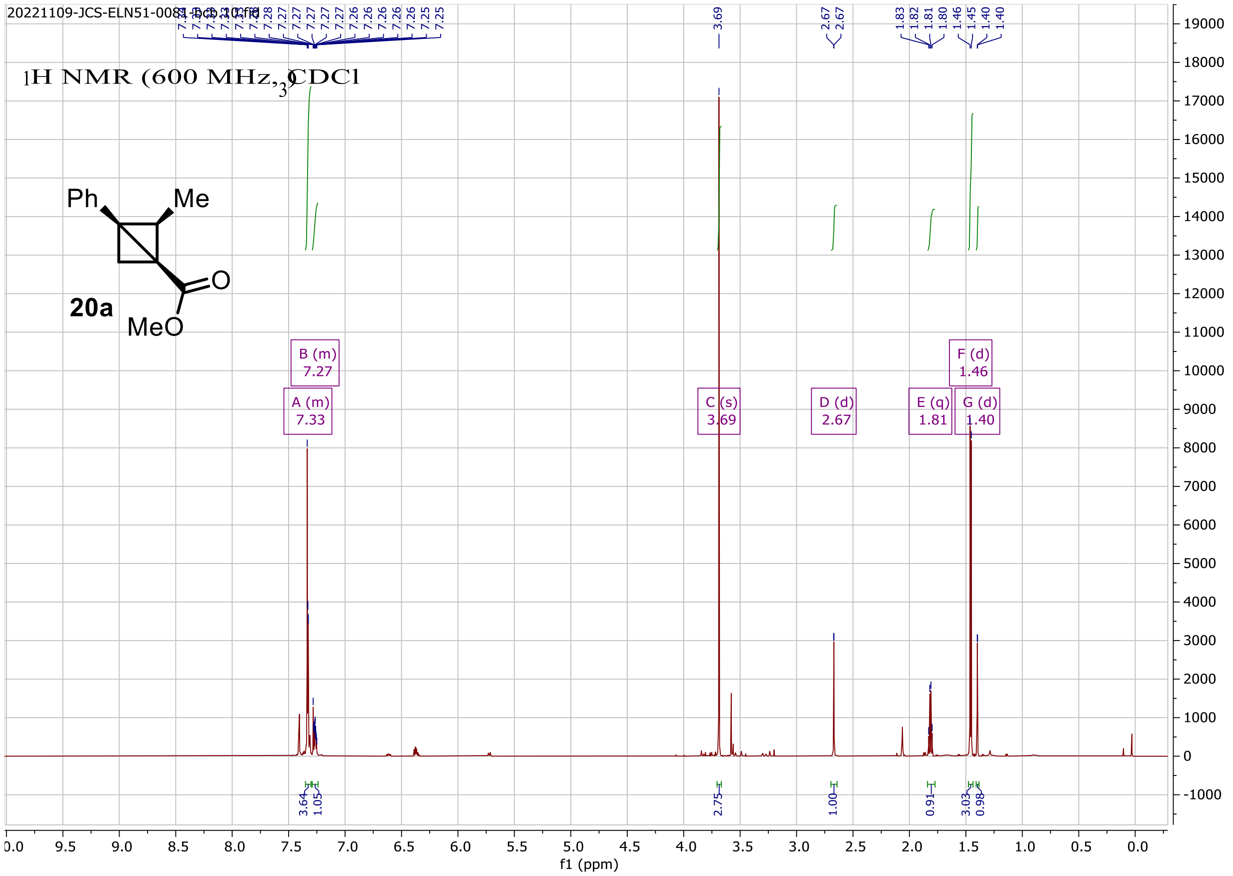
20221109-JCS-ELN51-0081-bcb.10.fid
1H NMR (600 MHz, CDCl3)
Ph
Me
O
20a
MeO
A (m) 7.33
B (m) 7.27
C (s) 3.69
D (d) 2.67
E (q) 1.81
F (d) 1.46
G (d) 1.40
3.64
1.05
2.75
1.00
0.91
3.03
0.98
f1 (ppm)

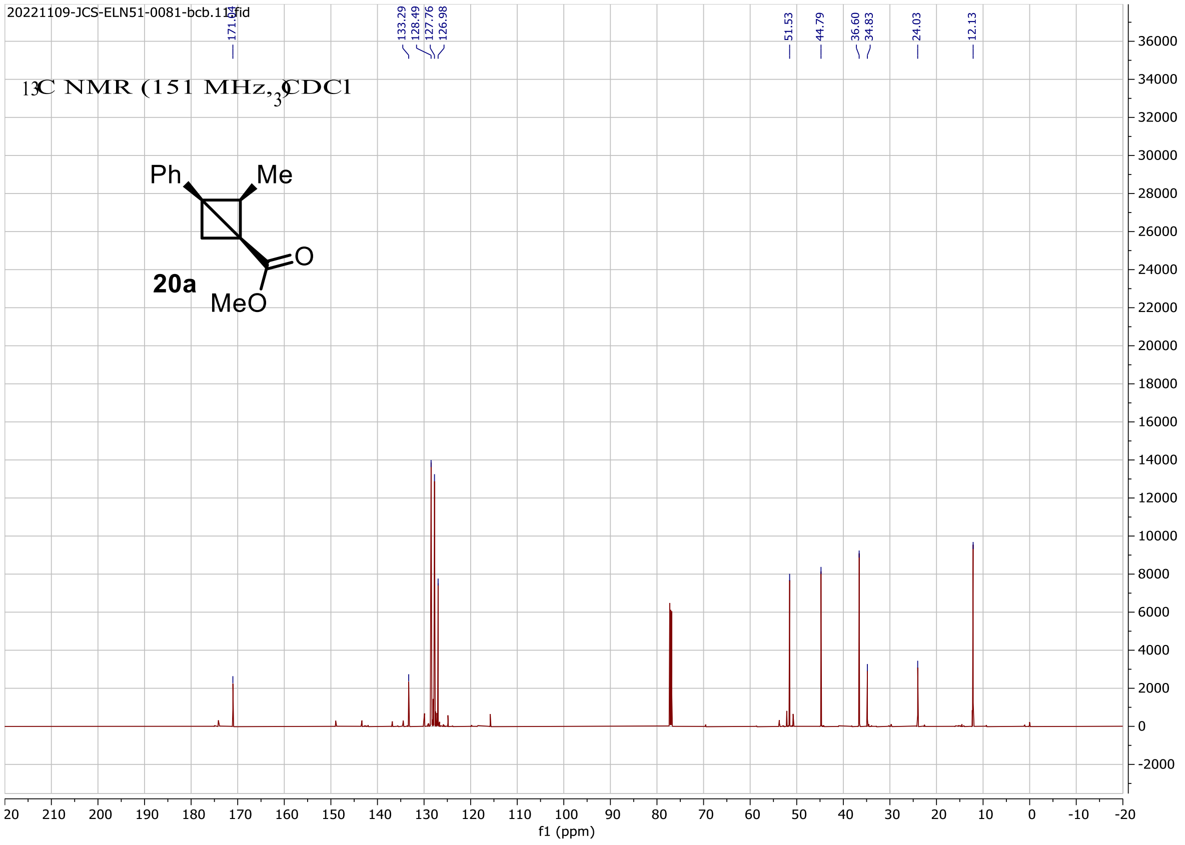
20221109-JCS-ELN51-0081-bcb.11.fid
13C NMR (151 MHz, CDCl3)
171.12
133.29
128.49
127.76
126.98
51.53
44.79
36.60
34.83
24.03
12.13
Ph
Me
O
20a
MeO
f1 (ppm)

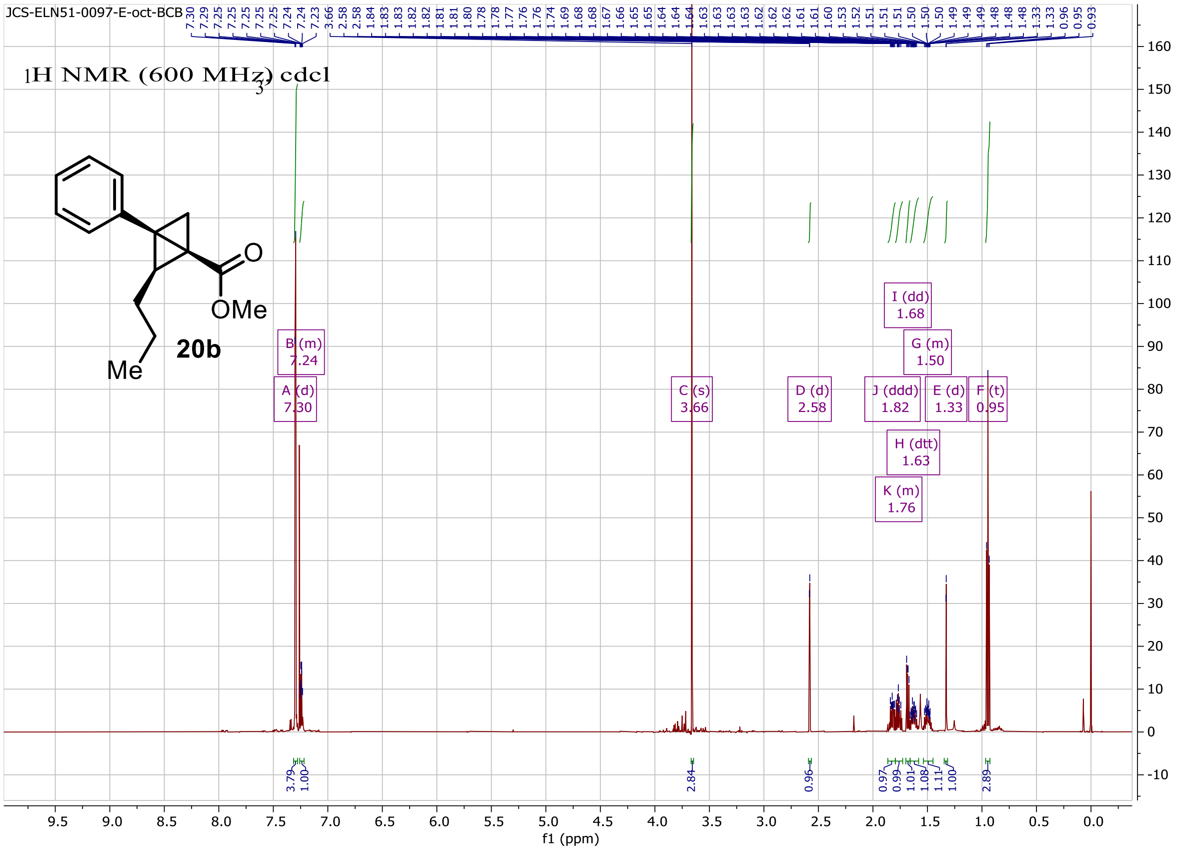

JCS-ELN51-0097-E-oct-BCB
7.30 7.29 7.25 7.25 7.25 7.25 7.25 7.24 7.24 7.23 3.66 2.58 2.58 1.84 1.83 1.83 1.82 1.82 1.81 1.81 1.80 1.78 1.78 1.77 1.76 1.76 1.74 1.69 1.68 1.68 1.67 1.66 1.65 1.65 1.64 1.64 1.64 1.63 1.63 1.63 1.63 1.62 1.62 1.62 1.61 1.61 1.60 1.53 1.52 1.51 1.51 1.51 1.50 1.50 1.50 1.49 1.49 1.49 1.48 1.48 1.33 1.33 0.96 0.95 0.93
1H NMR (600 MHz) cdcl3
O
OMe
Me
20b
I (dd) 1.68
B (m) 7.24
G (m) 1.50
A (d) 7.30
C (s) 3.66
D (d) 2.58
J (ddd) 1.82
E (d) 1.33
F (t) 0.95
H (dtt) 1.63
K (m) 1.76
3.79 1.00 2.84 0.96 0.97 0.99 1.01 1.08 1.11 1.00 2.89
f1 (ppm)

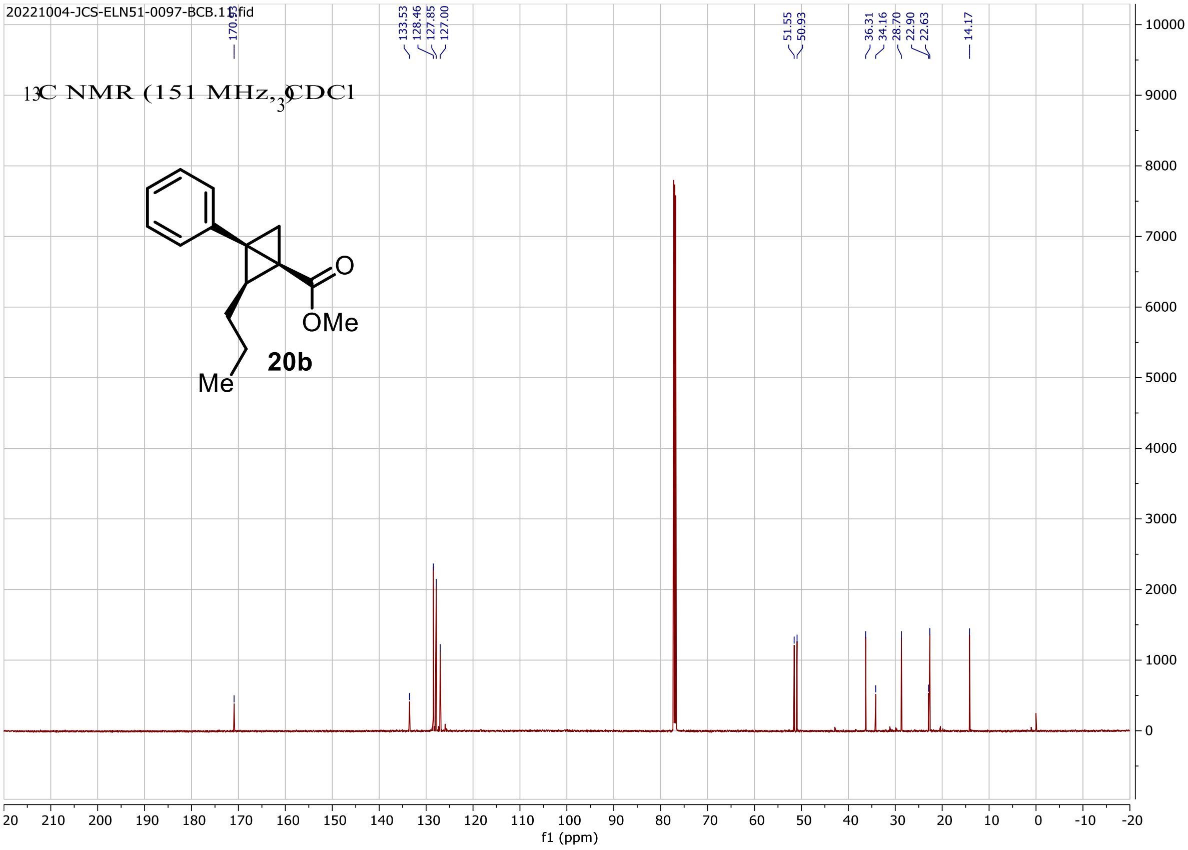

20221004-JCS-ELN51-0097-BCB.11.fid
170.95
133.53 128.46 127.85 127.00
51.55 50.93
36.31 34.16 28.70 22.90 22.63
14.17
13C NMR (151 MHz, CDCl3)
O
OMe
Me
20b
f1 (ppm)

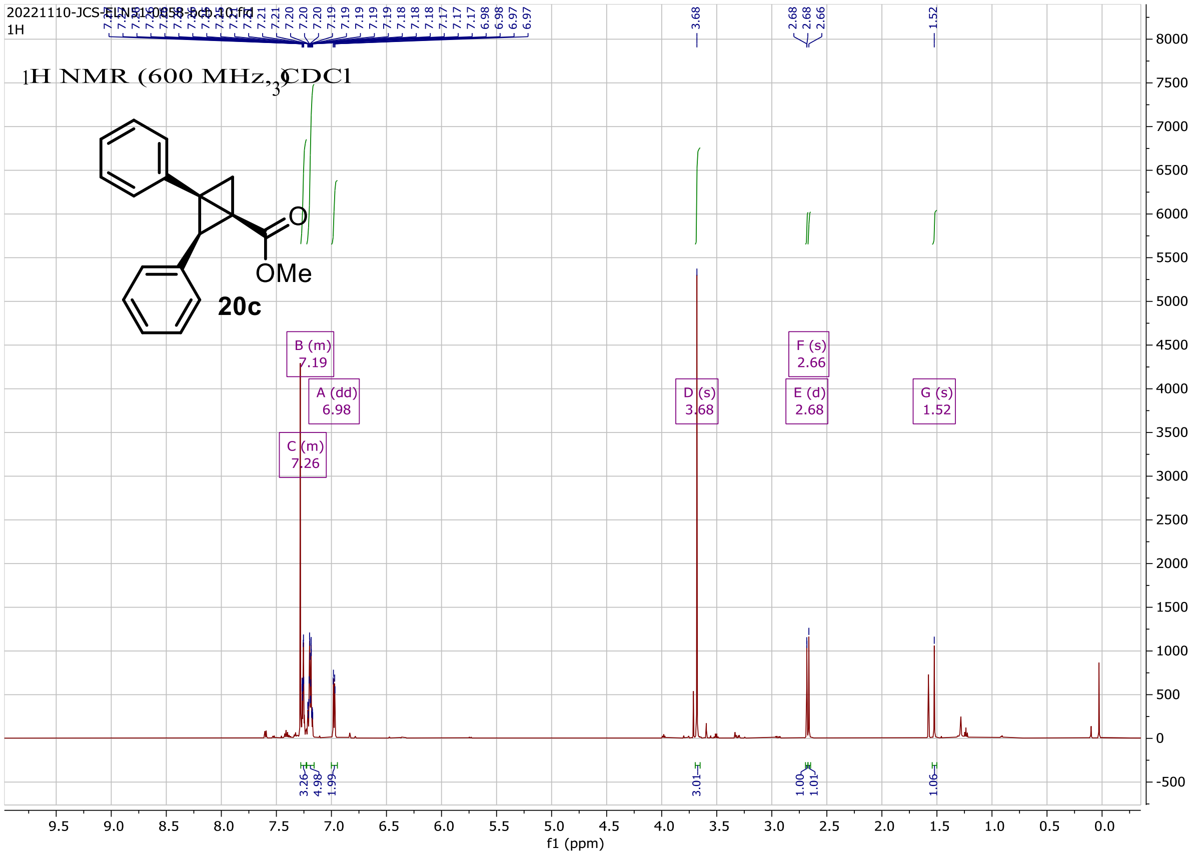
20221110-JCS-ELN51-0058-bcb.10.fid
1H
1H NMR (600 MHz, CDCl3)
O
OMe
20c
B (m) 7.19
A (dd) 6.98
C (m) 7.26
D (s) 3.68
F (s) 2.66
E (d) 2.68
G (s) 1.52
3.68
2.68
2.68
2.66
1.52
3.26
4.98
1.99
3.01
1.00
1.01
1.06
f1 (ppm)

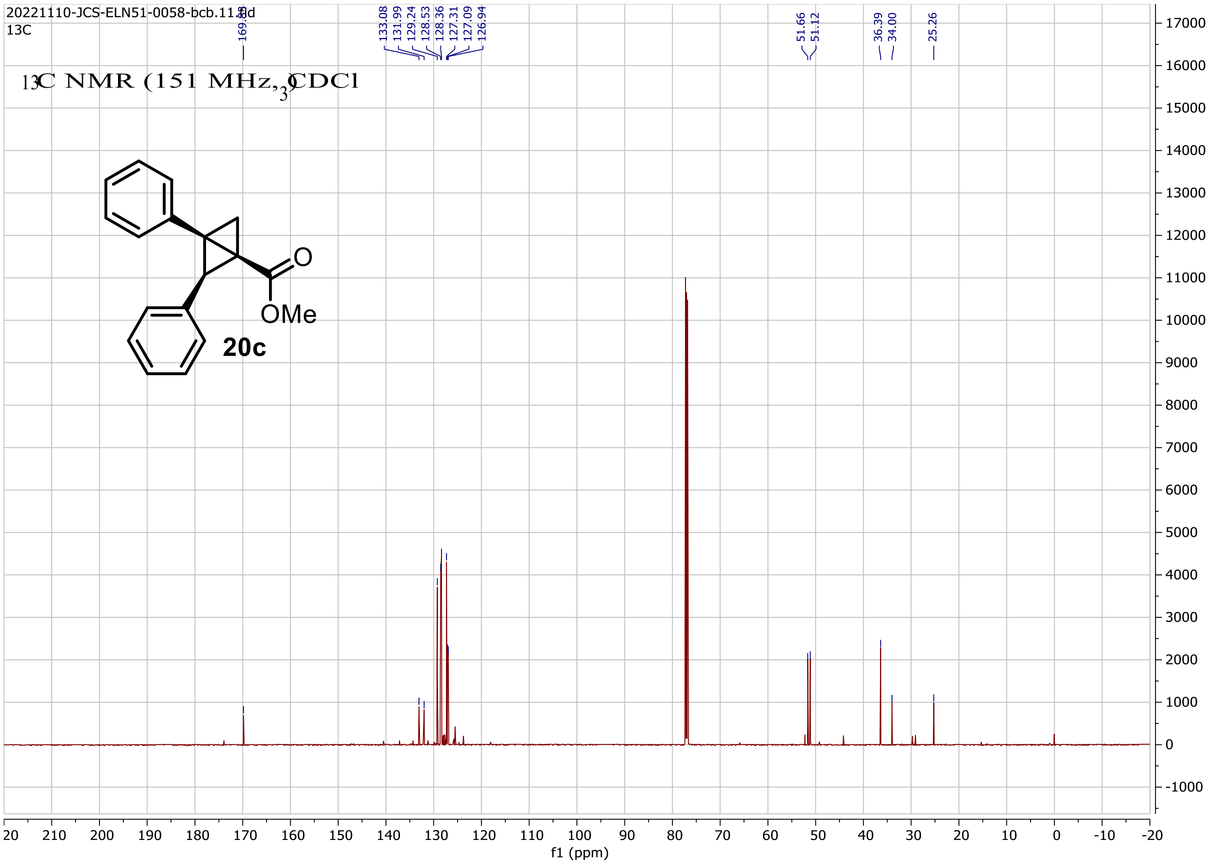
20221110-JCS-ELN51-0058-bcb.11.fid
13C
13C NMR (151 MHz, CDCl3)
O
OMe
20c
133.08
131.99
129.24
128.53
128.36
127.31
127.09
126.94
51.66
51.12
36.39
34.00
25.26
f1 (ppm)

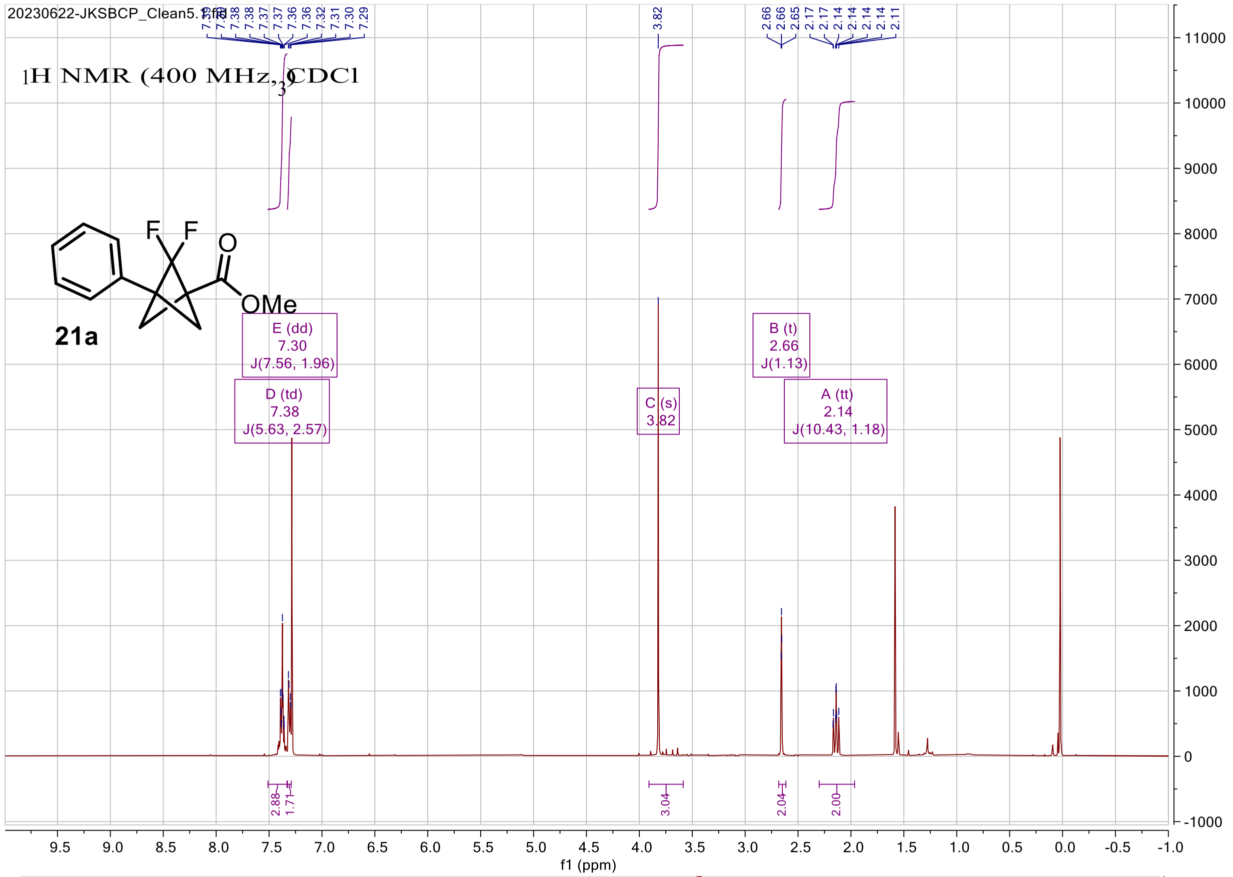
20230622-JKSBCP_Clean5.1.fid
1H NMR (400 MHz, CDCl3)
21a
E (dd) 7.30 J(7.56, 1.96)
D (td) 7.38 J(5.63, 2.57)
C (s) 3.82
B (t) 2.66 J(1.13)
A (tt) 2.14 J(10.43, 1.18)
f1 (ppm)

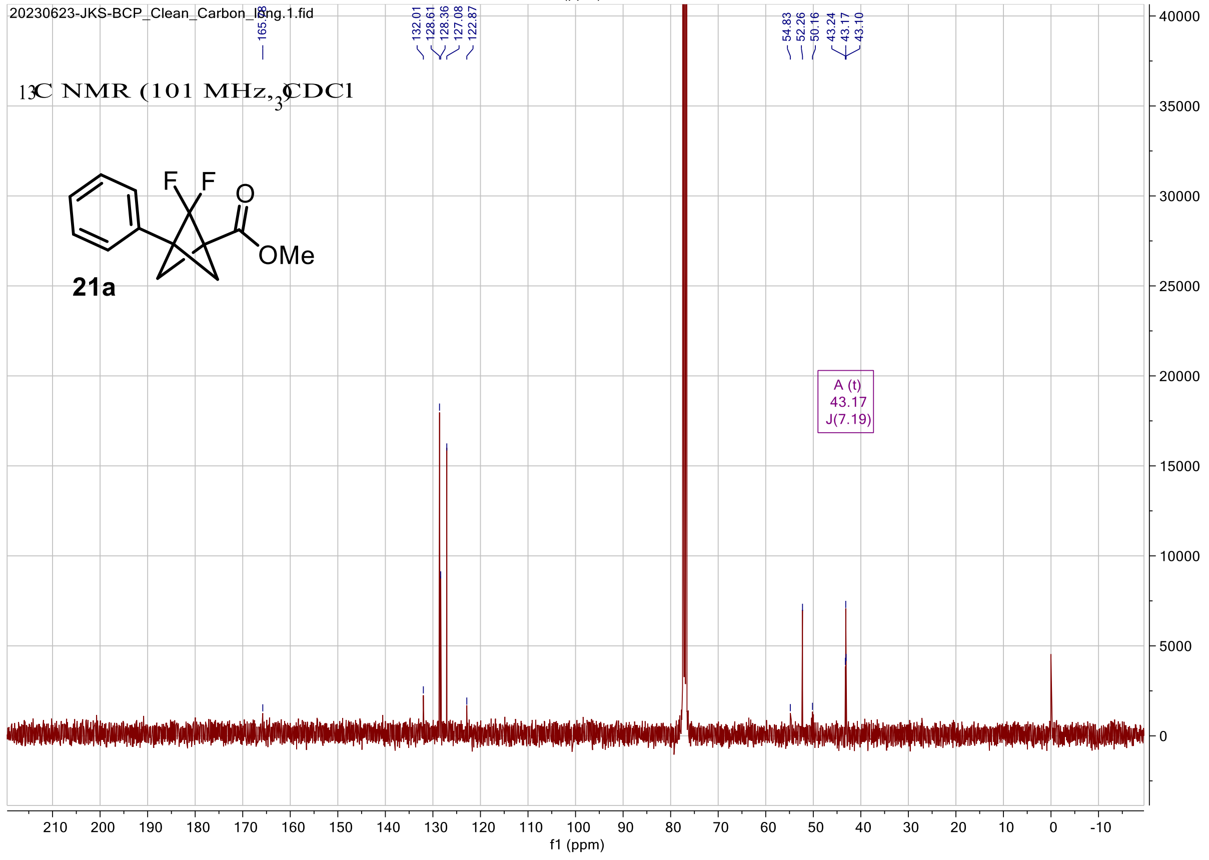
20230623-JKS-BCP_Clean_Carbon_long.1.fid
13C NMR (101 MHz, CDCl3)
21a
A (t) 43.17 J(7.19)
f1 (ppm)

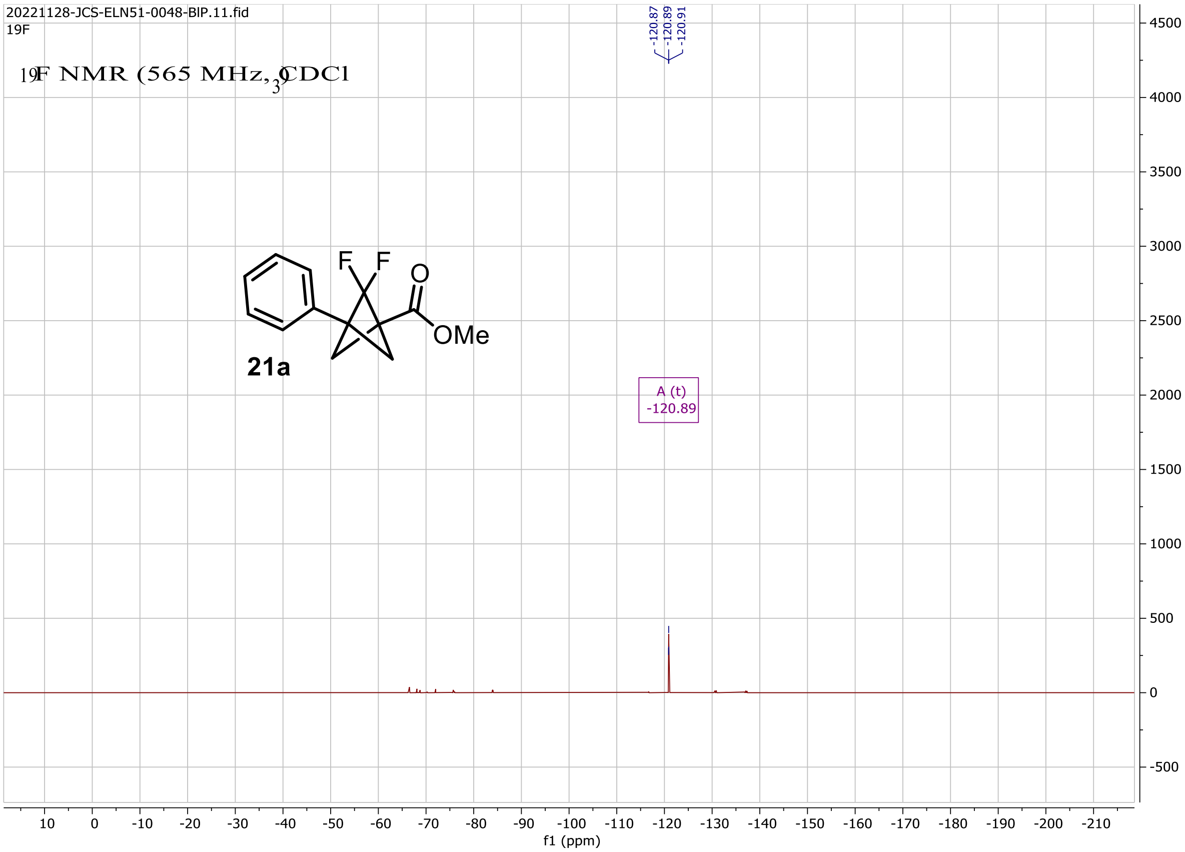
20221128-JCS-ELN51-0048-BIP.11.fid
19F
19F NMR (565 MHz, CDCl3)
-120.87
-120.89
-120.91
21a
OMe
A (t)
-120.89
f1 (ppm)

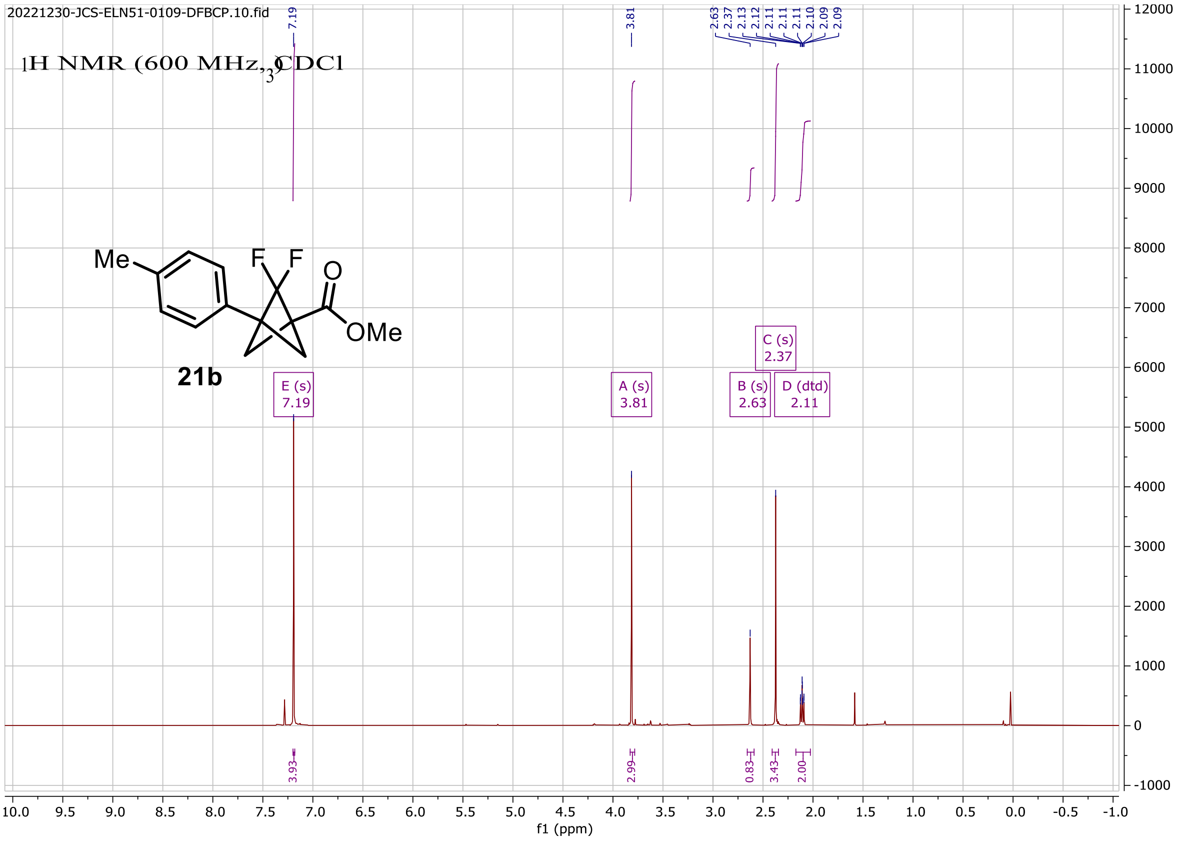
20221230-JCS-ELN51-0109-DFBCP.10.fid
1H NMR (600 MHz, CDCl3)
7.19
3.81
2.63
2.37
2.13
2.12
2.11
2.11
2.11
2.11
2.10
2.09
2.09
21b
Me
OMe
E (s)
7.19
A (s)
3.81
B (s)
2.63
C (s)
2.37
D (dtd)
2.11
3.93
2.99
0.83
3.43
2.00
f1 (ppm)

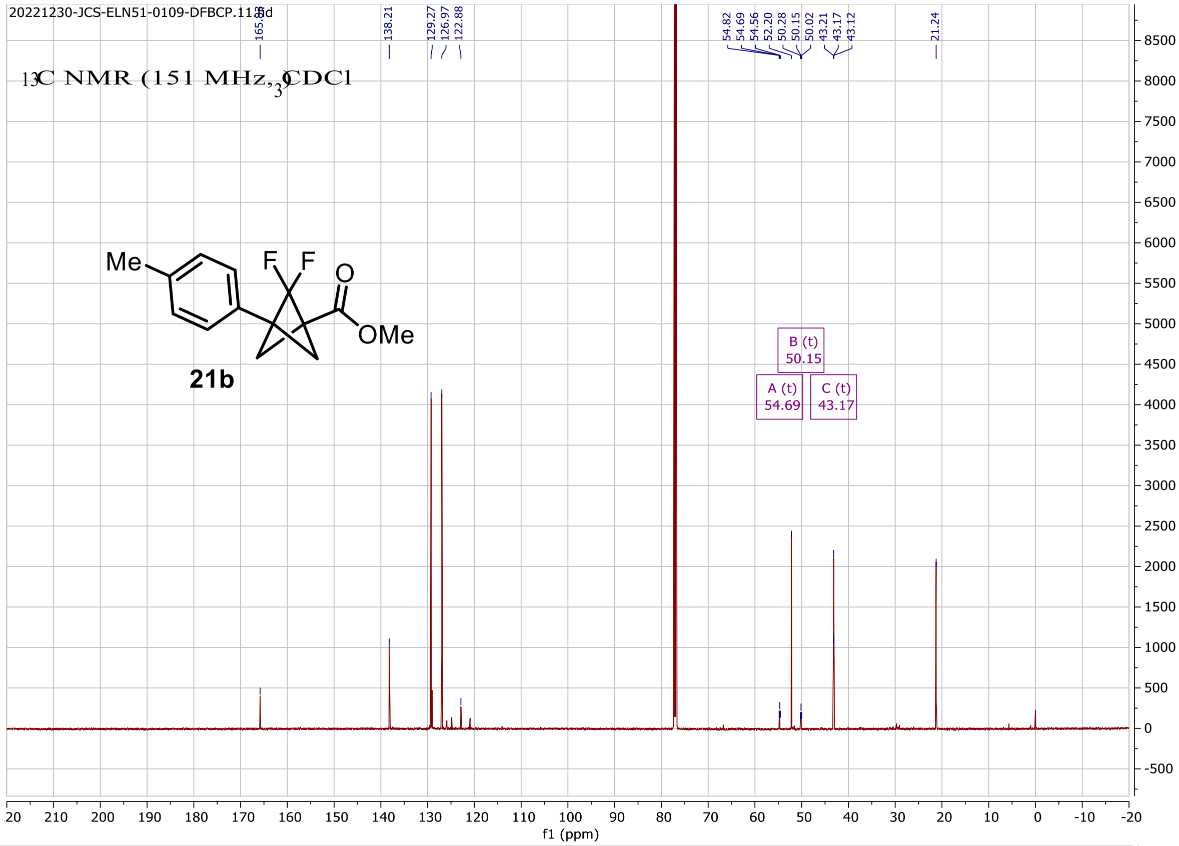

20221230-JCS-ELN51-0109-DFBCP.11.fid
13C NMR (151 MHz, CDCl3)
165.88
138.21
129.27
126.97
122.88
54.82
54.69
54.56
52.20
50.28
50.15
50.02
43.21
43.17
43.12
21.24
Me
F
F
O
OMe
21b
B (t)
50.15
A (t)
54.69
C (t)
43.17
f1 (ppm)

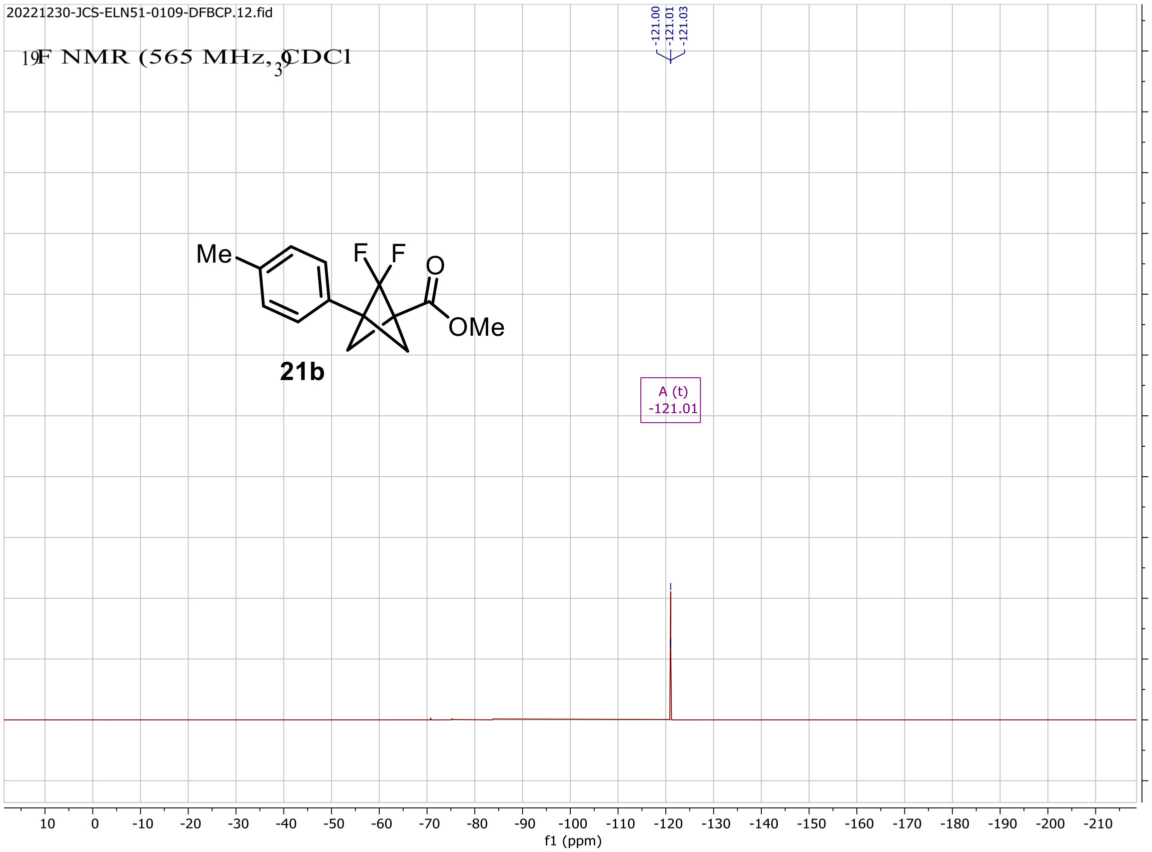

20221230-JCS-ELN51-0109-DFBCP.12.fid
19F NMR (565 MHz, CDCl3)
-121.00
-121.01
-121.03
Me
F
F
O
OMe
21b
A (t)
-121.01
f1 (ppm)

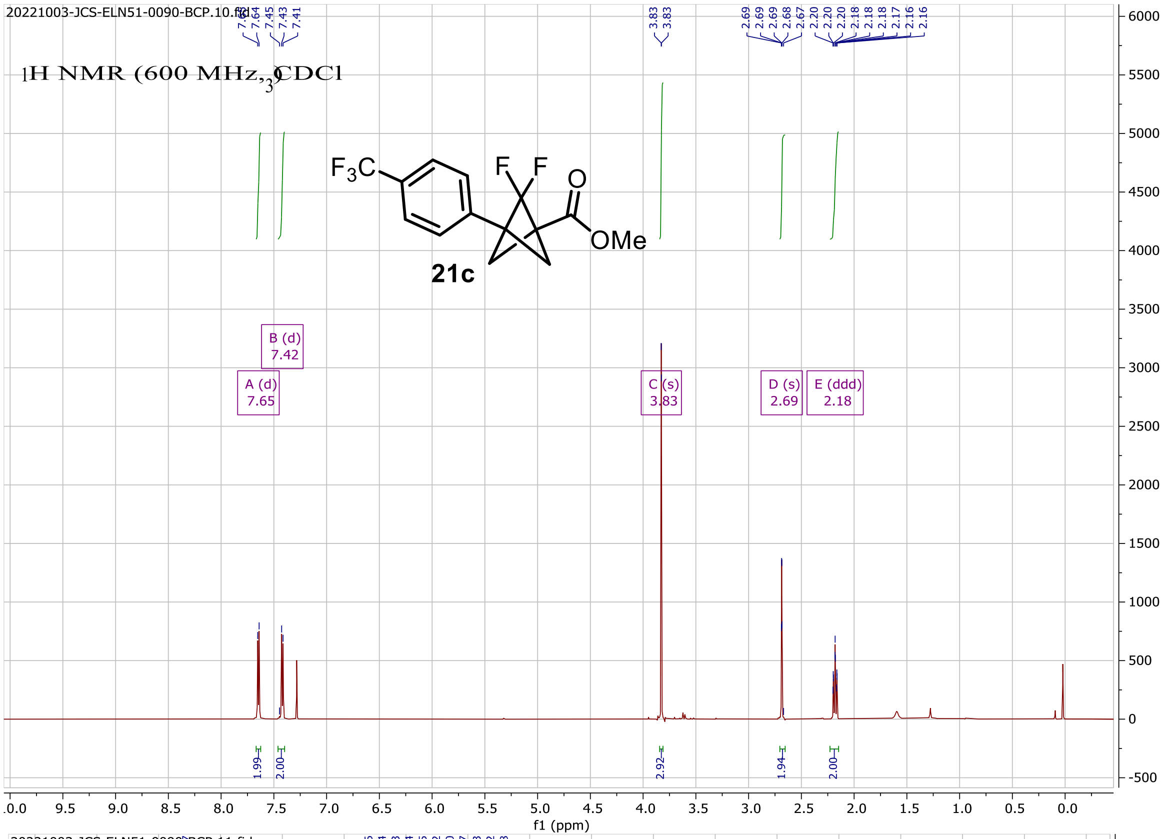
20221003-JCS-ELN51-0090-BCP.10.fid
1H NMR (600 MHz, CDCl3)
F3C
F F
O
OMe
21c
A (d) 7.65
B (d) 7.42
C (s) 3.83
D (s) 2.69
E (ddd) 2.18
1.99
2.00
2.92
1.94
2.00
f1 (ppm)

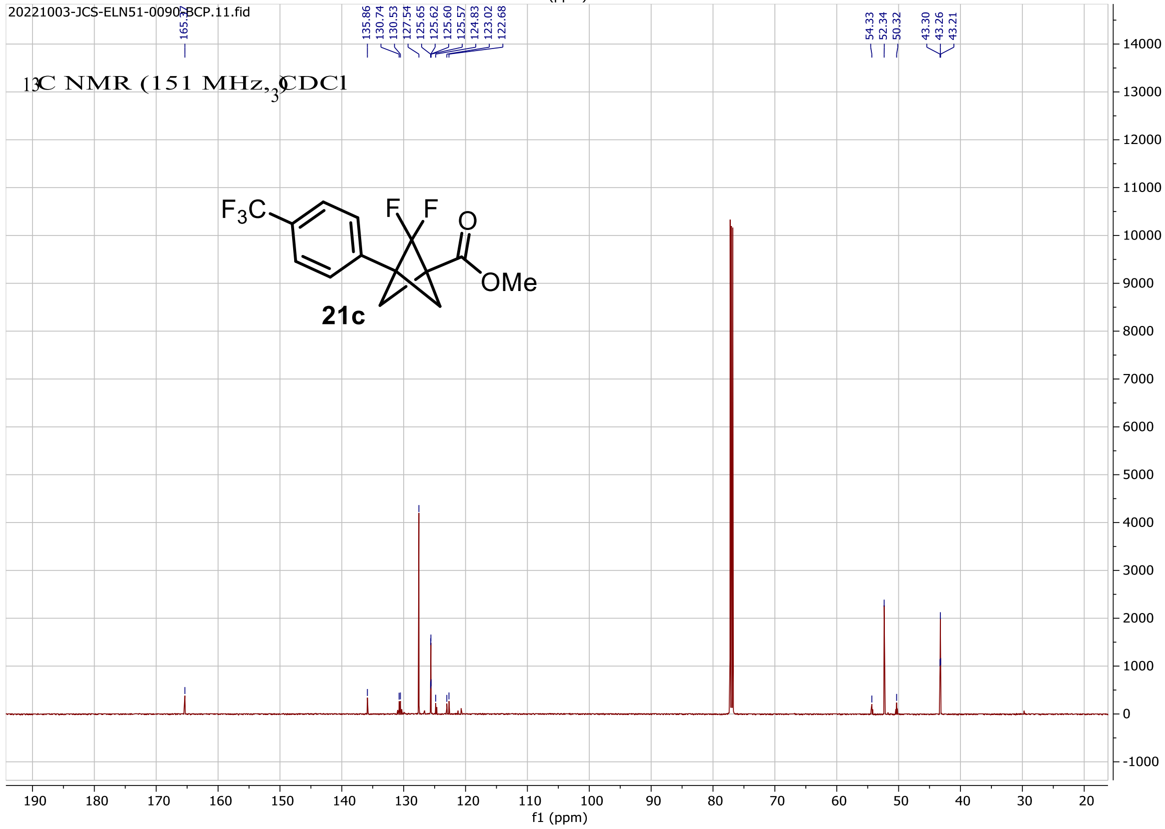
20221003-JCS-ELN51-0090-BCP.11.fid
13C NMR (151 MHz, CDCl3)
135.86
130.74
130.53
127.54
125.65
125.62
125.60
125.57
124.83
123.02
122.68
54.33
52.34
50.32
43.30
43.26
43.21
F3C
F F
O
OMe
21c
f1 (ppm)

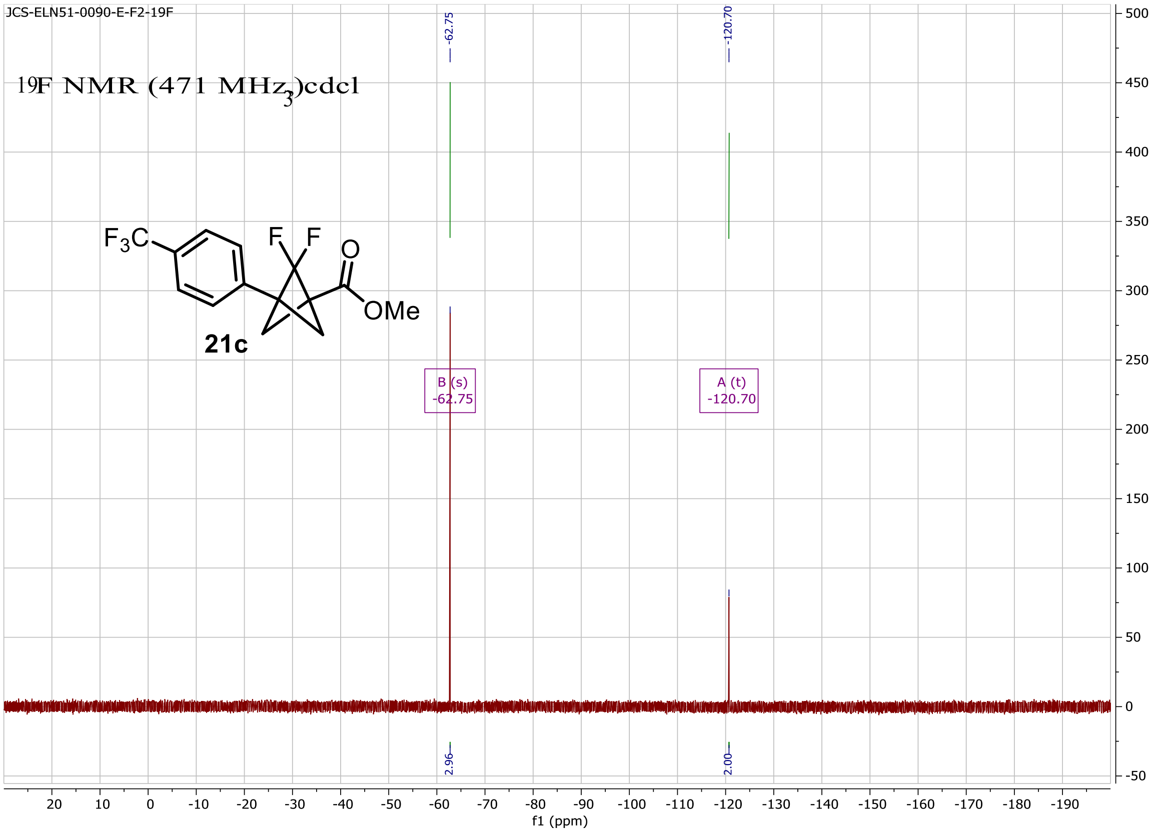

JCS-ELN51-0090-E-F2-19F
19F NMR (471 MHz) cdcl3
F3C
F
F
O
OMe
21c
B (s)
-62.75
A (t)
-120.70
f1 (ppm)

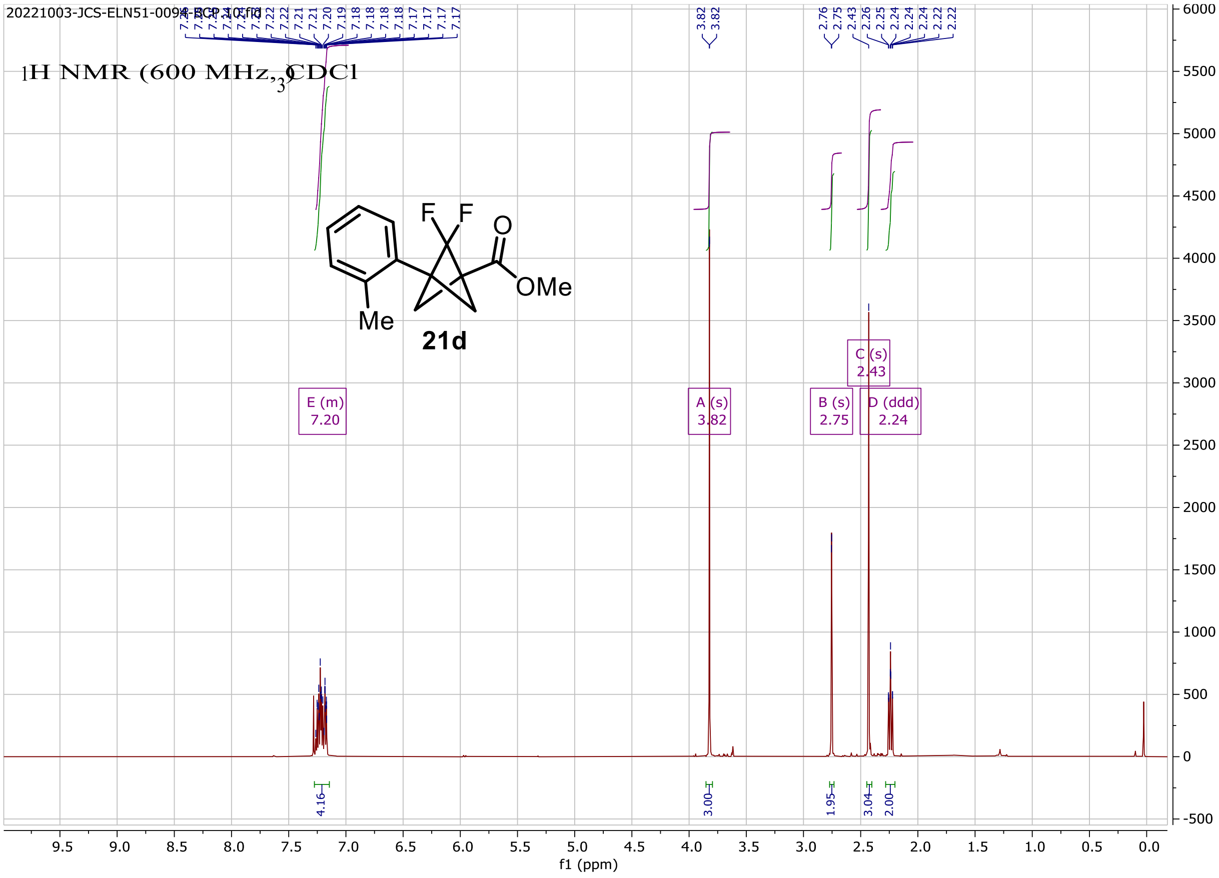

1H NMR (600 MHz, CDCl3)
F
F
O
OMe
Me
21d
E (m)
7.20
A (s)
3.82
B (s)
2.75
C (s)
2.43
D (ddd)
2.24
f1 (ppm)

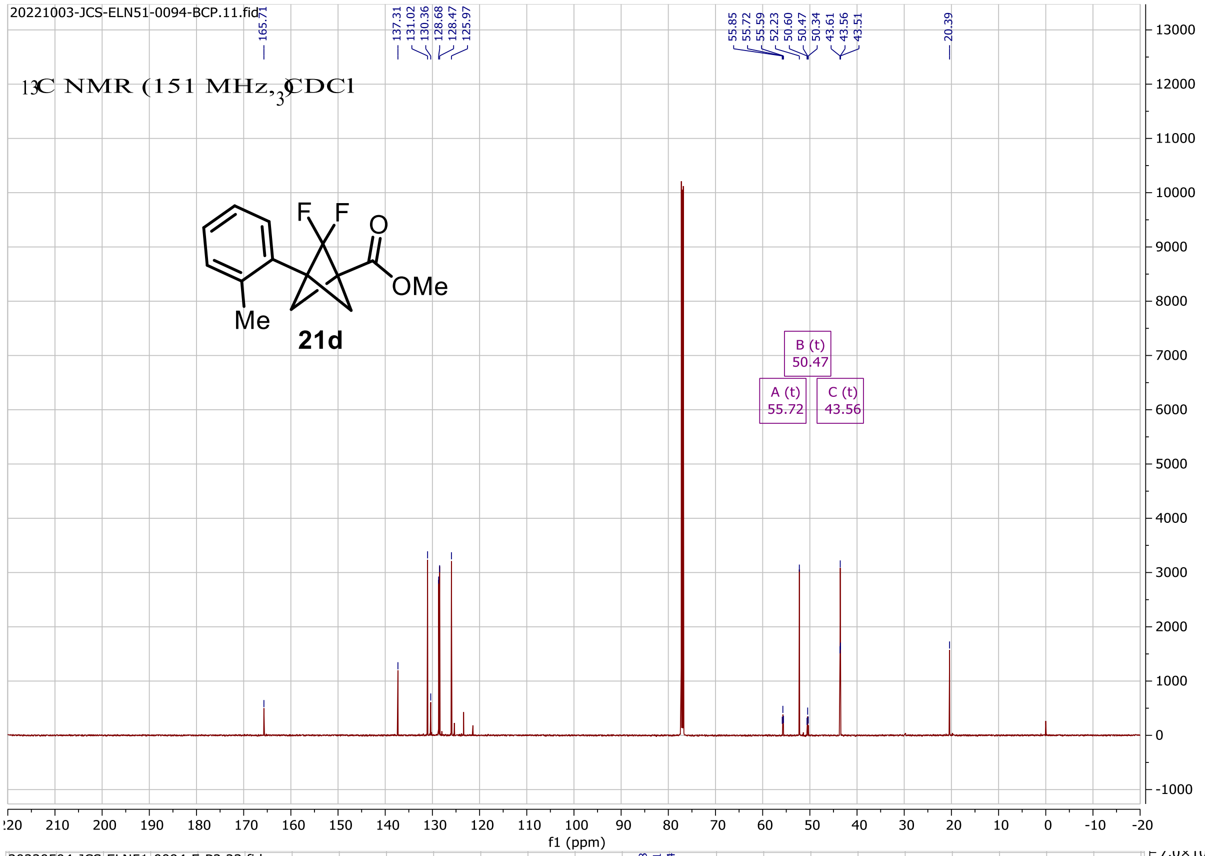
20221003-JCS-ELN51-0094-BCP.11.fid
13C NMR (151 MHz, CDCl3)
165.71
137.31
131.02
130.36
128.68
128.47
125.97
55.85
55.72
55.59
52.23
50.60
50.47
50.34
43.61
43.56
43.51
20.39
F
F
O
OMe
Me
21d
B (t)
50.47
A (t)
55.72
C (t)
43.56
f1 (ppm)

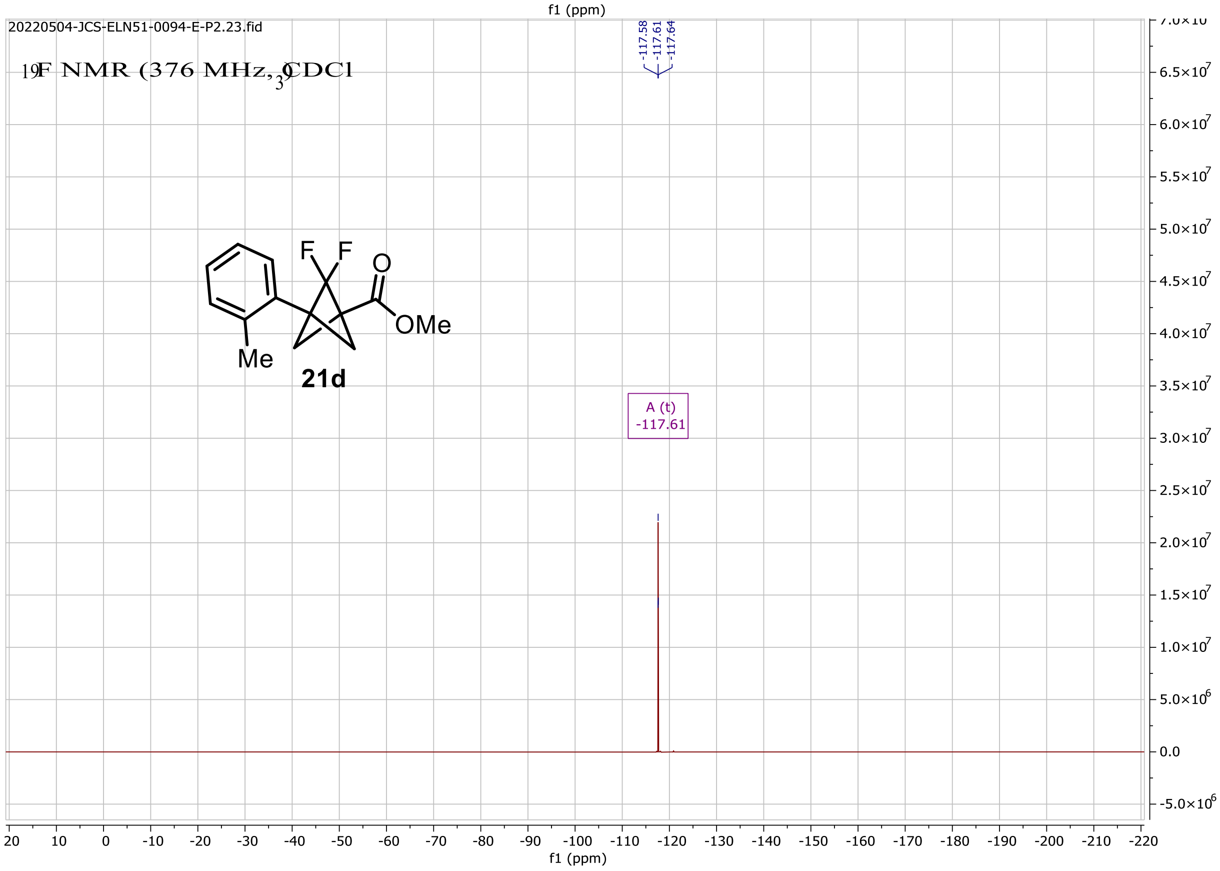
20220504-JCS-ELN51-0094-E-P2.23.fid
19F NMR (376 MHz, CDCl3)
-117.58
-117.61
-117.64
F
F
O
OMe
Me
21d
A (t)
-117.61
f1 (ppm)

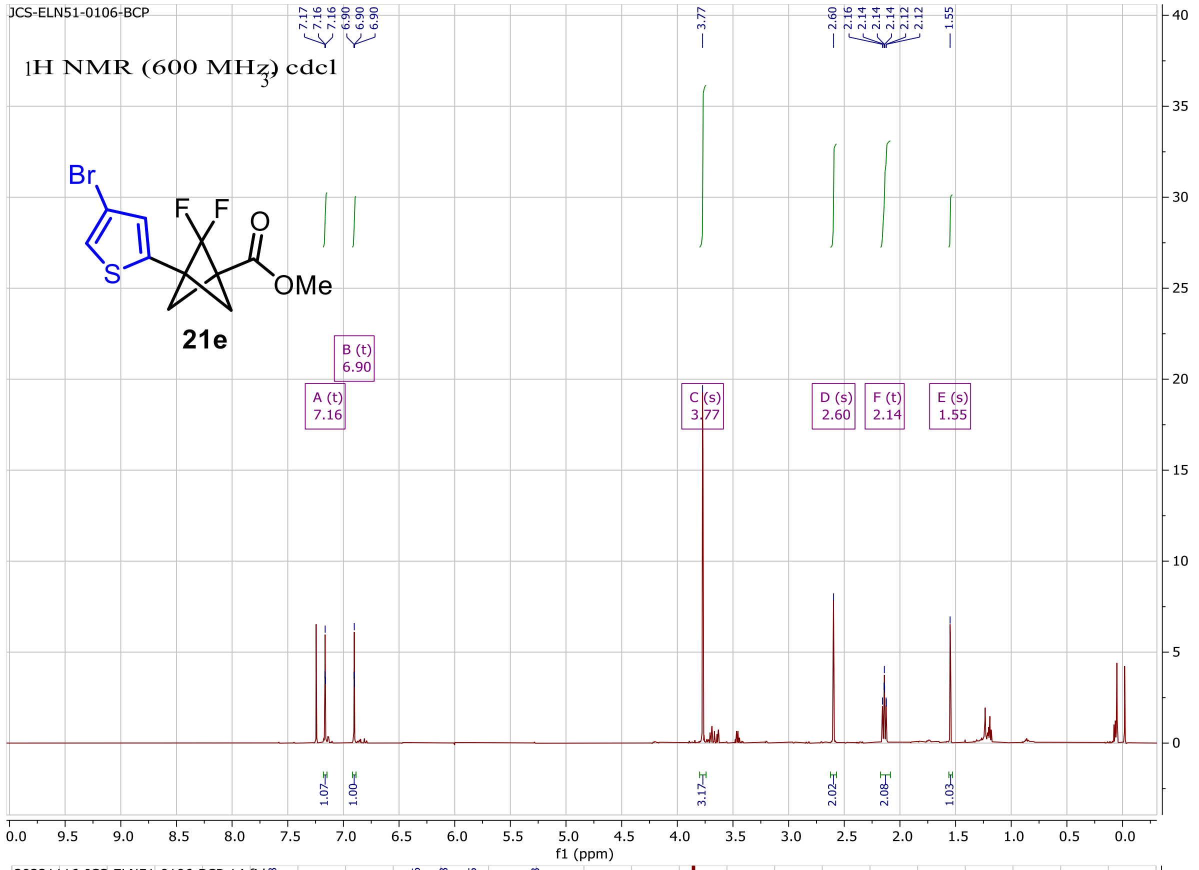
JCS-ELN51-0106-BCP
1H NMR (600 MHz, cdcl3)
Br
F F
O
S
OMe
21e
A (t) 7.16
B (t) 6.90
C (s) 3.77
D (s) 2.60
F (t) 2.14
E (s) 1.55
f1 (ppm)

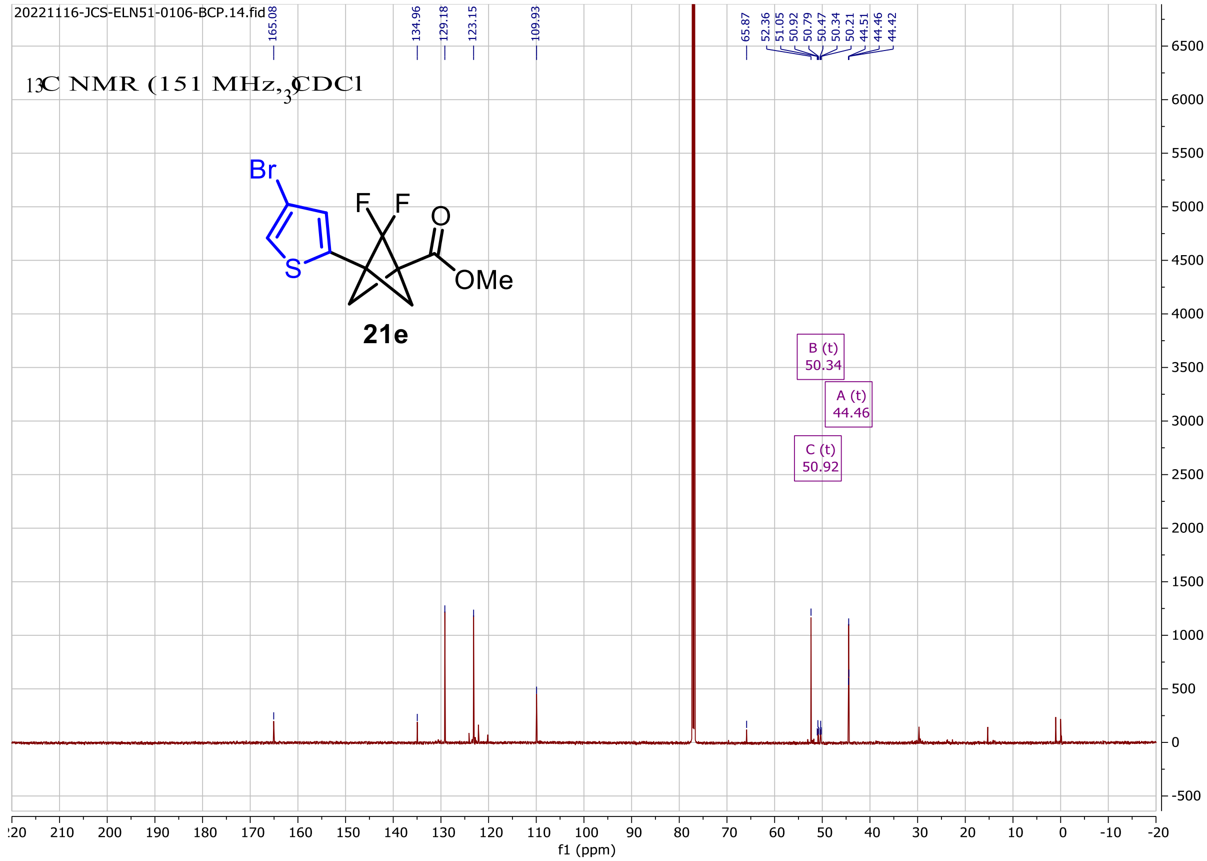
20221116-JCS-ELN51-0106-BCP.14.fid
13C NMR (151 MHz, CDCl3)
Br
F F
O
S
OMe
21e
B (t) 50.34
A (t) 44.46
C (t) 50.92
f1 (ppm)

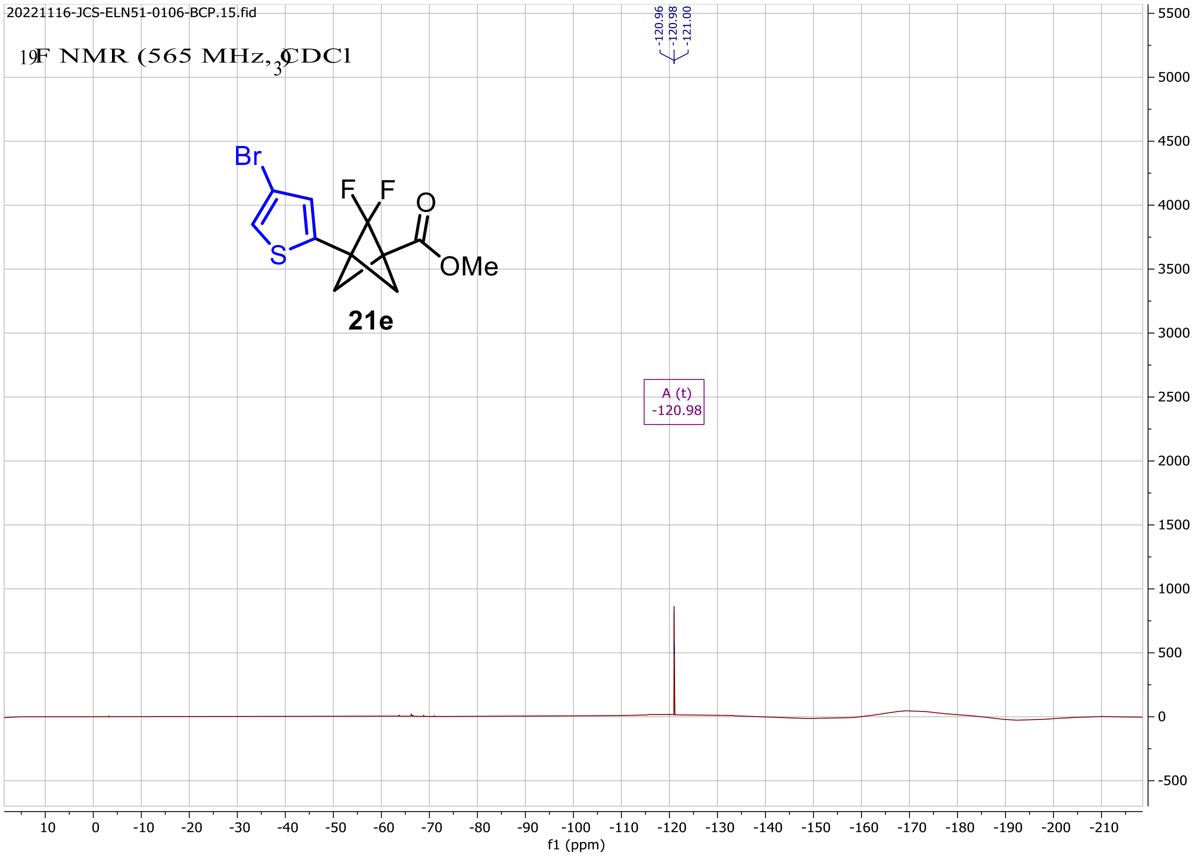
20221116-JCS-ELN51-0106-BCP.15.fid
19F NMR (565 MHz, CDCl3)
-120.96
-120.98
-121.00
Br
F
F
O
S
OMe
21e
A (t)
-120.98
f1 (ppm)

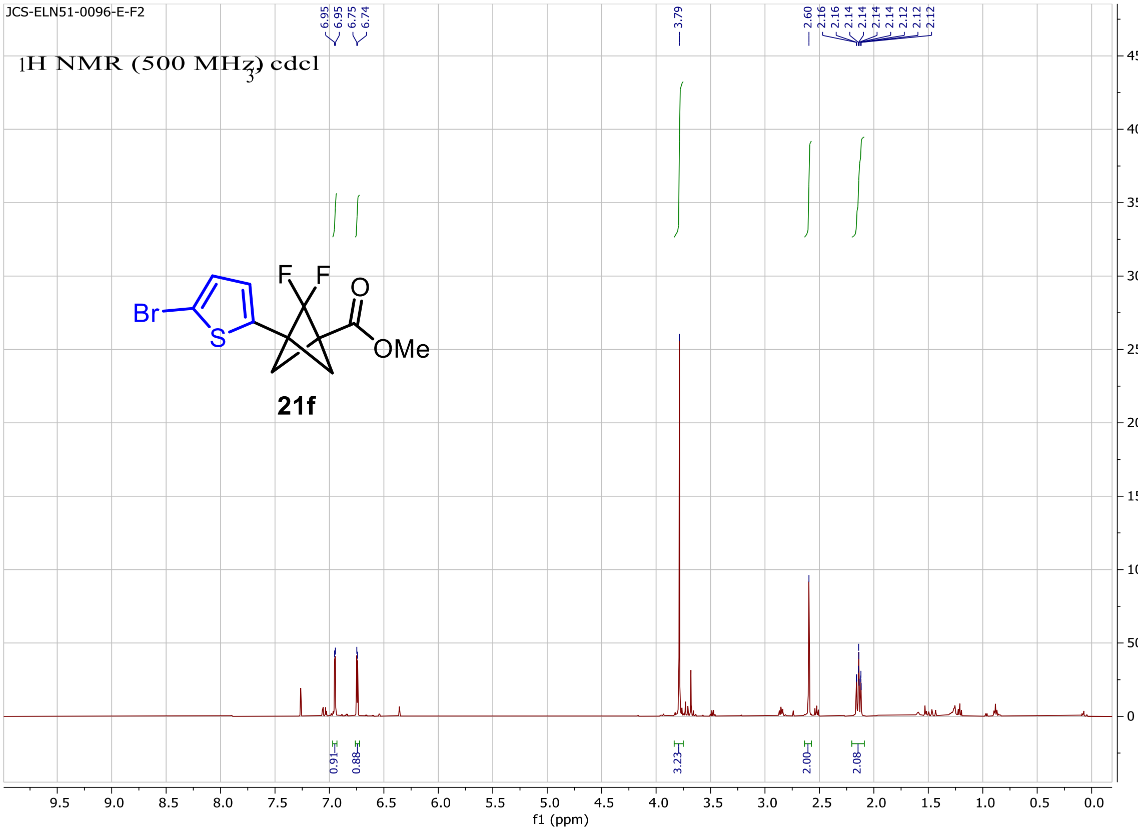
JCS-ELN51-0096-E-F2
1H NMR (500 MHz, cdcl3)
6.95
6.95
6.75
6.74
3.79
2.60
2.16
2.16
2.14
2.14
2.14
2.14
2.12
2.12
2.12
Br
F
F
O
S
OMe
21f
0.91
0.88
3.23
2.00
2.08
f1 (ppm)

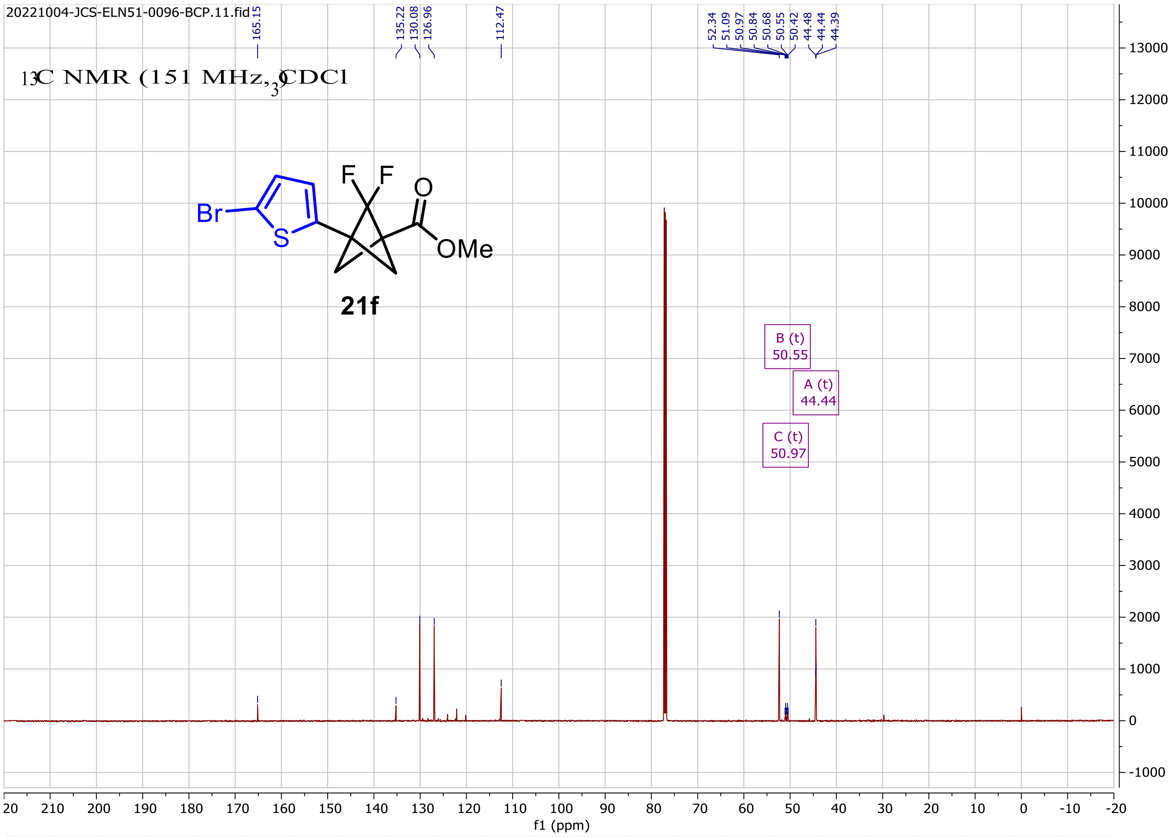
20221004-JCS-ELN51-0096-BCP.11.fid
13C NMR (151 MHz, CDCl3)
165.15
135.22
130.08
126.96
112.47
52.34
51.09
50.97
50.84
50.68
50.55
50.42
44.48
44.44
44.39
Br
S
F
F
O
OMe
21f
B (t)
50.55
A (t)
44.44
C (t)
50.97
f1 (ppm)

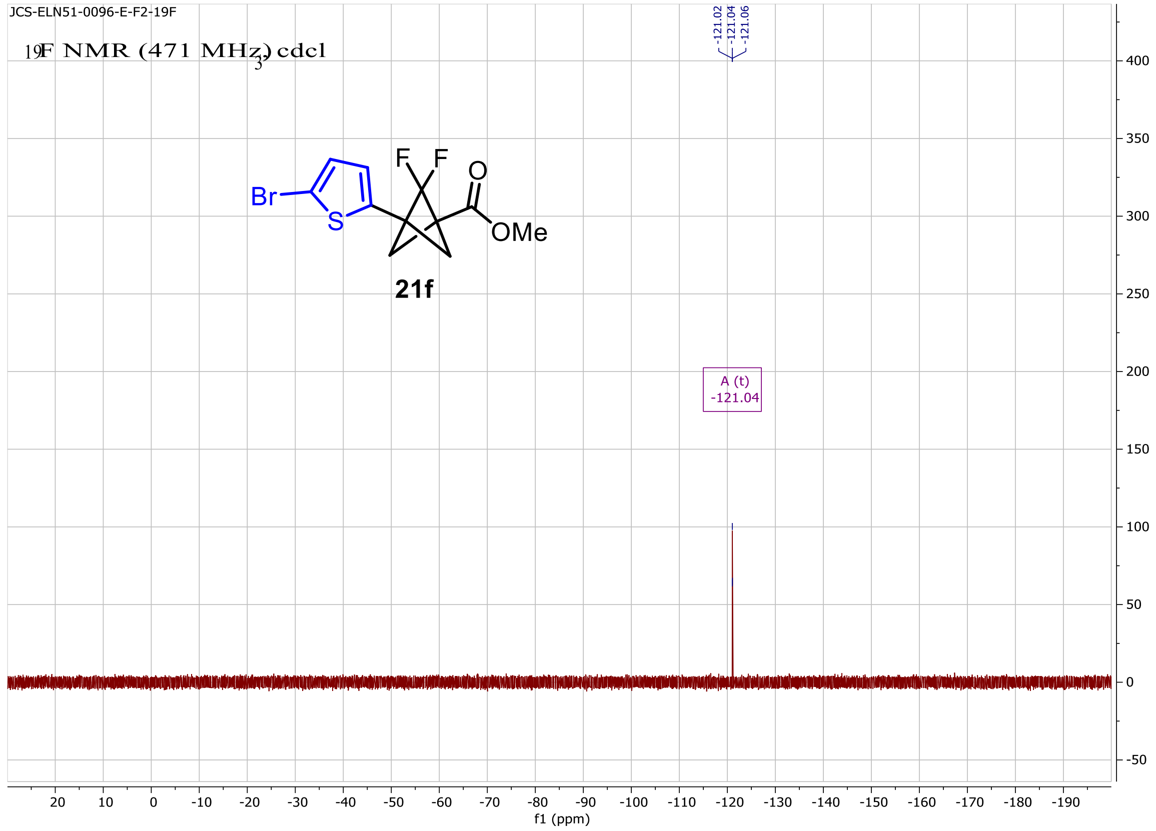
JCS-ELN51-0096-E-F2-19F
19F NMR (471 MHz) cdcl3
-121.02
-121.04
-121.06
Br
S
F
F
O
OMe
21f
A (t)
-121.04
f1 (ppm)

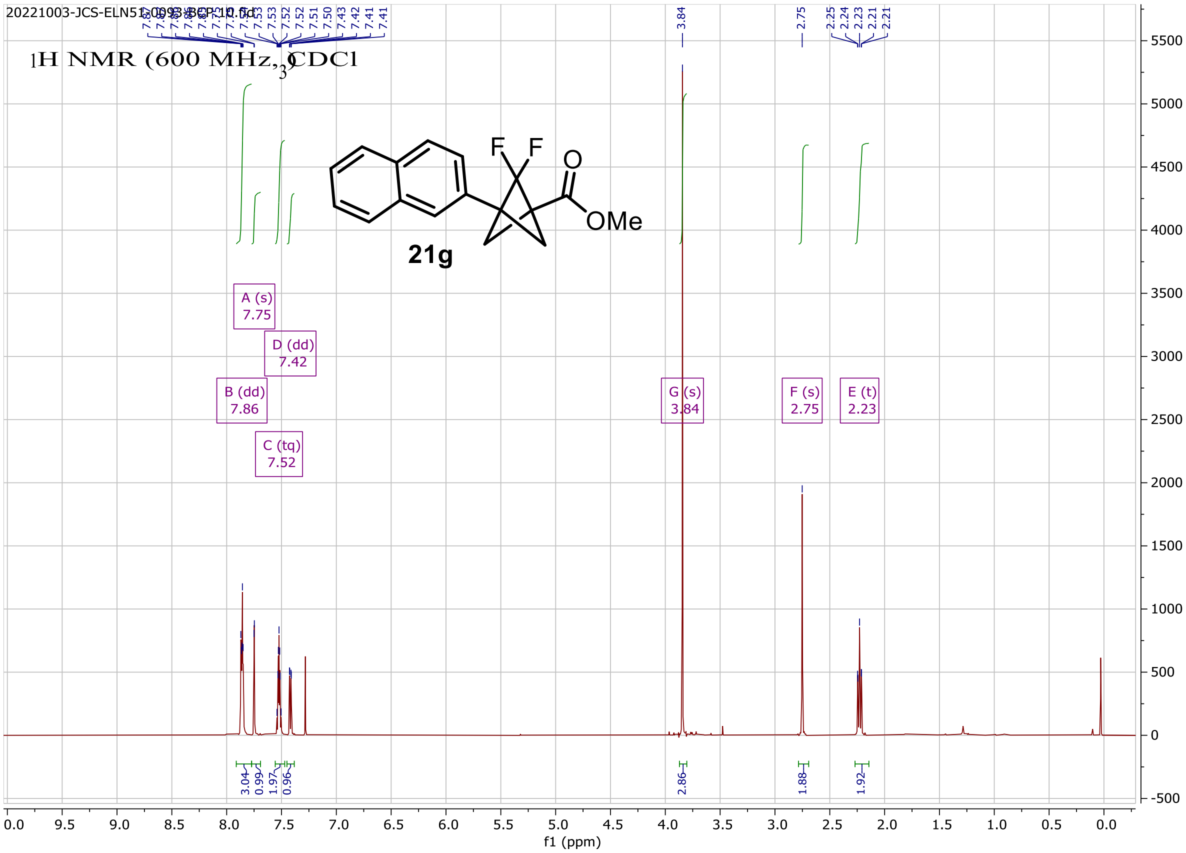
20221003-JCS-ELN51-0093-BCP.10.fid
1H NMR (600 MHz, CDCl3)
F
F
O
OMe
21g
A (s) 7.75
D (dd) 7.42
B (dd) 7.86
C (tq) 7.52
G (s) 3.84
F (s) 2.75
E (t) 2.23
3.84
2.75
2.25
2.24
2.23
2.21
2.21
3.04
0.99
1.97
0.96
2.86
1.88
1.92
f1 (ppm)

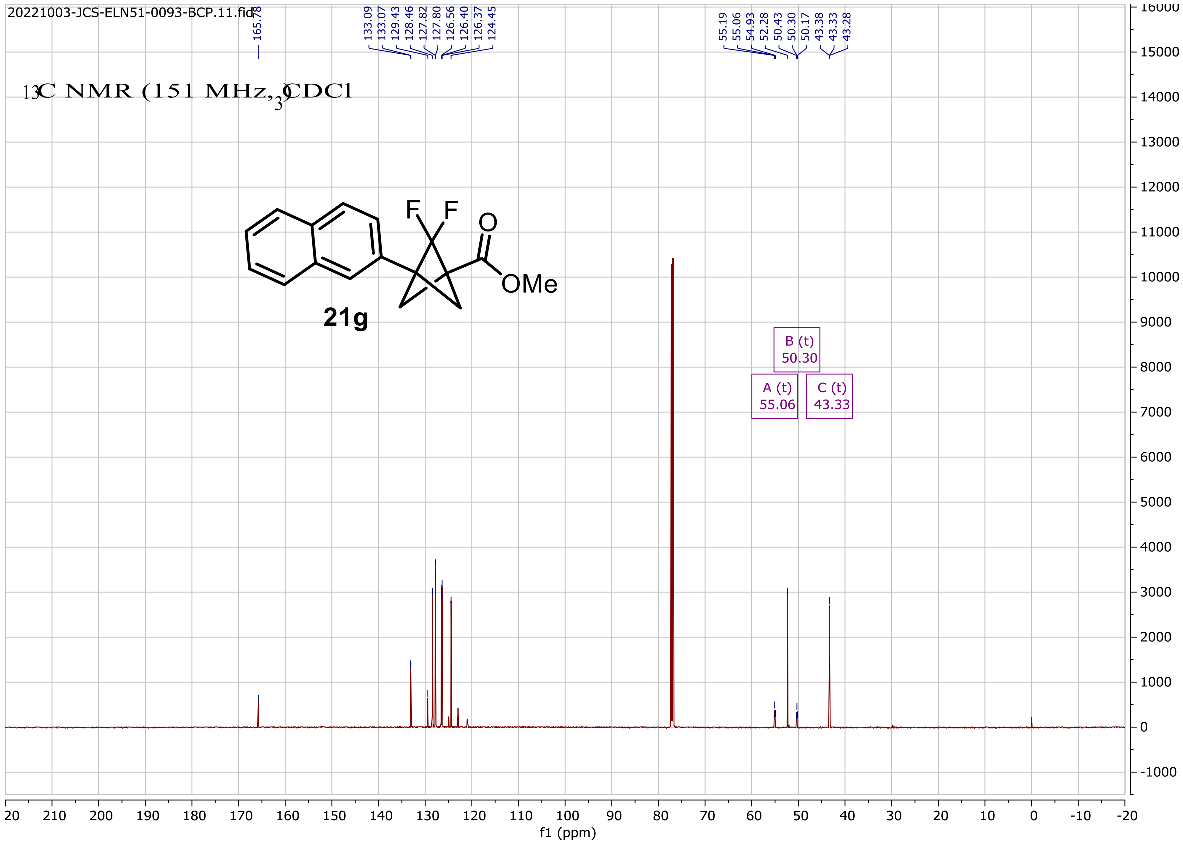
20221003-JCS-ELN51-0093-BCP.11.fid
13C NMR (151 MHz, CDCl3)
165.78
133.09
133.07
129.43
128.46
127.82
127.80
126.56
126.40
126.37
124.45
55.19
55.06
54.93
52.28
50.43
50.30
50.17
43.38
43.33
43.28
F
F
O
OMe
21g
B (t) 50.30
A (t) 55.06
C (t) 43.33
f1 (ppm)

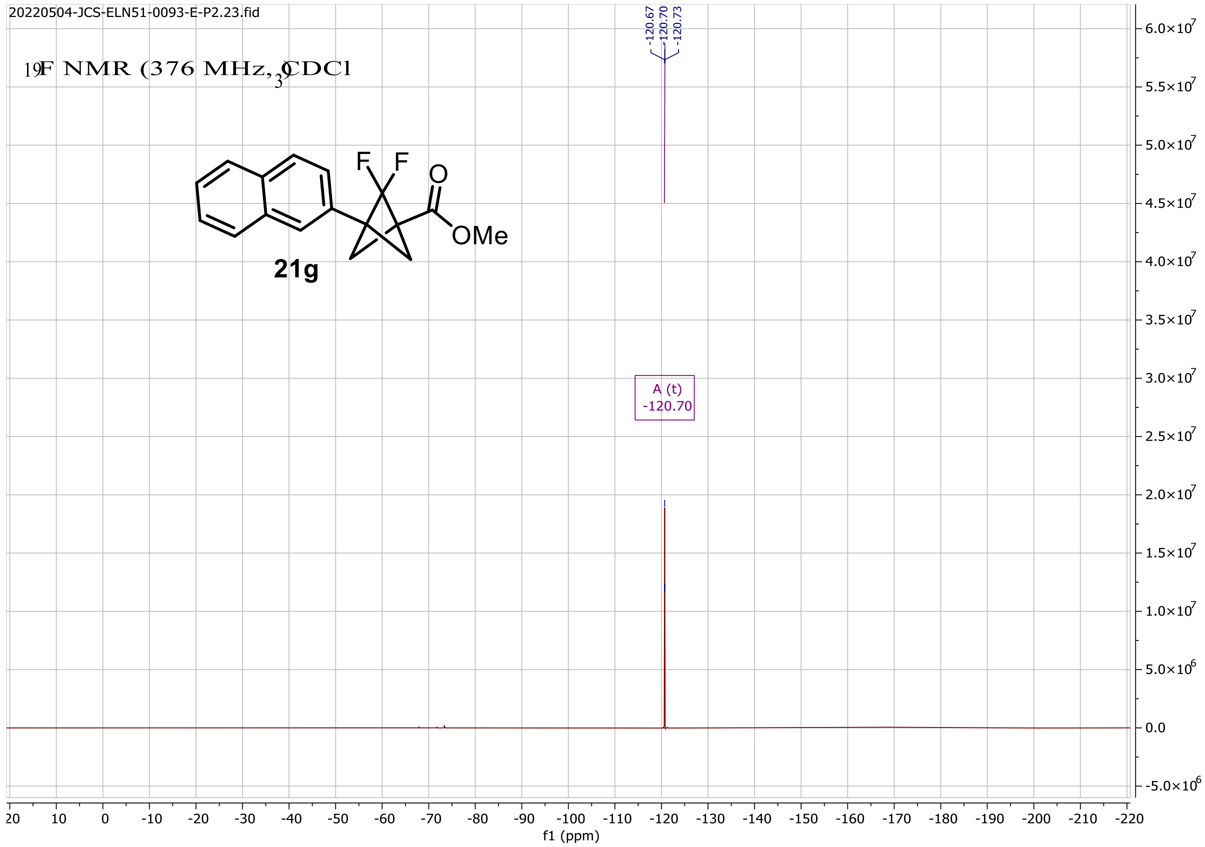
20220504-JCS-ELN51-0093-E-P2.23.fid
19F NMR (376 MHz, CDCl3)
-120.67
-120.70
-120.73
F
F
O
OMe
21g
A (t)
-120.70
f1 (ppm)

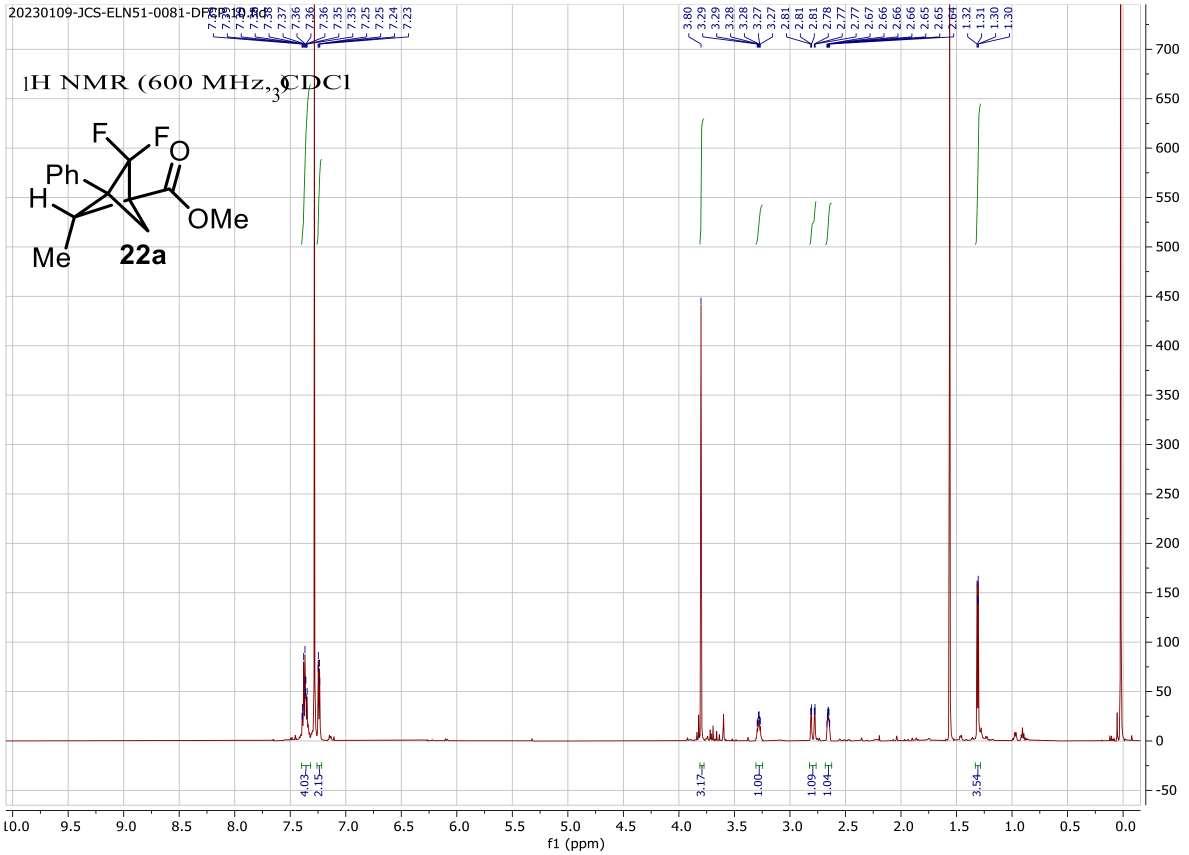
20230109-JCS-ELN51-0081-DFCP-10.fid
1H NMR (600 MHz, CDCl3)
F
F
O
Ph
H
OMe
Me
22a
4.03
2.15
3.17
1.00
1.09
1.04
3.54
f1 (ppm)

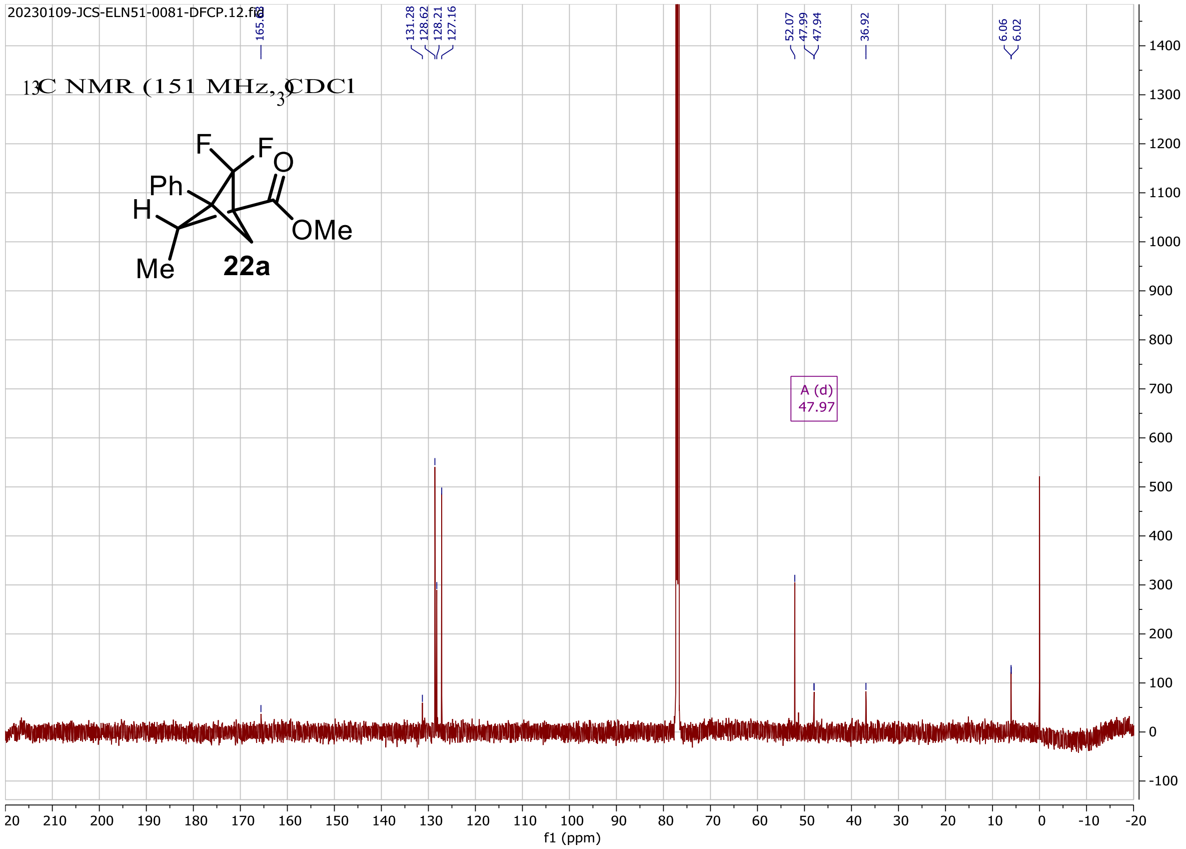
20230109-JCS-ELN51-0081-DFCP.12.fid
13C NMR (151 MHz, CDCl3)
22a
A (d)
47.97
f1 (ppm)

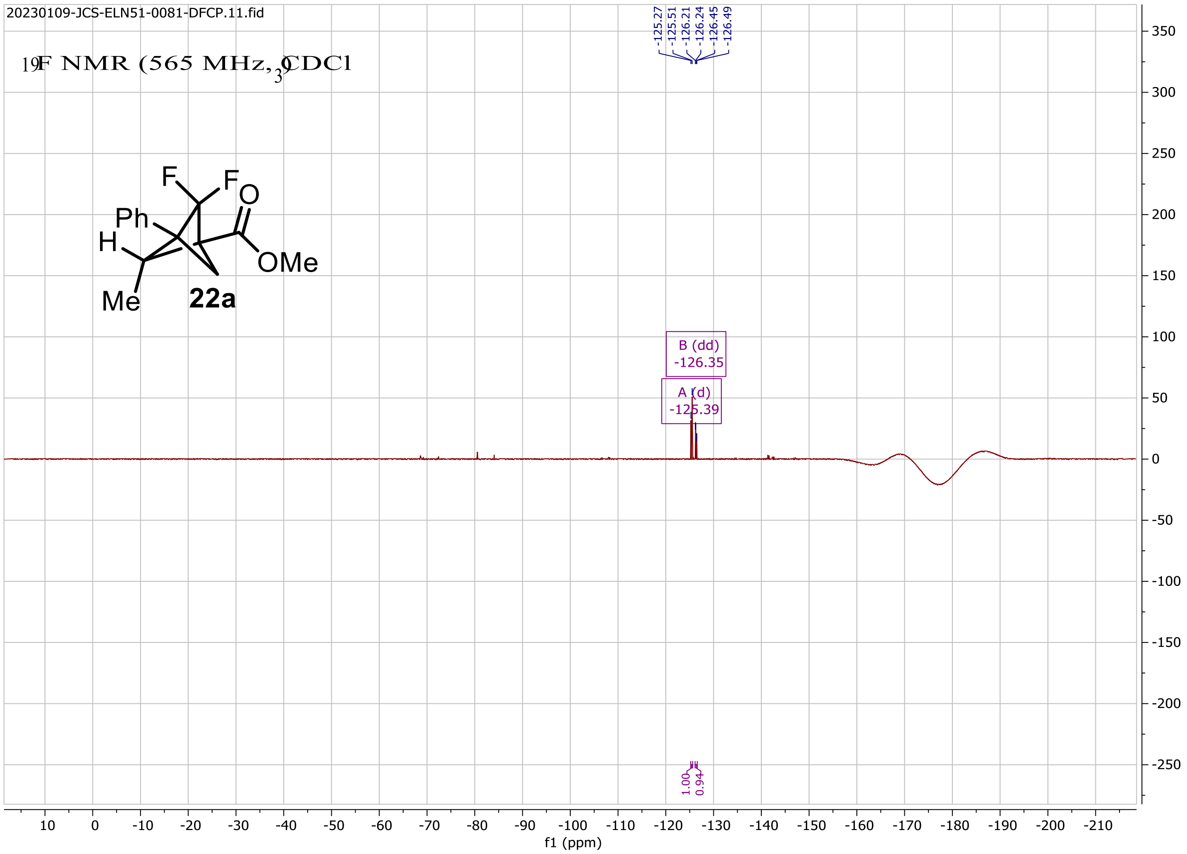
20230109-JCS-ELN51-0081-DFCP.11.fid
19F NMR (565 MHz, CDCl3)
22a
B (dd)
-126.35
A (d)
-125.39
f1 (ppm)

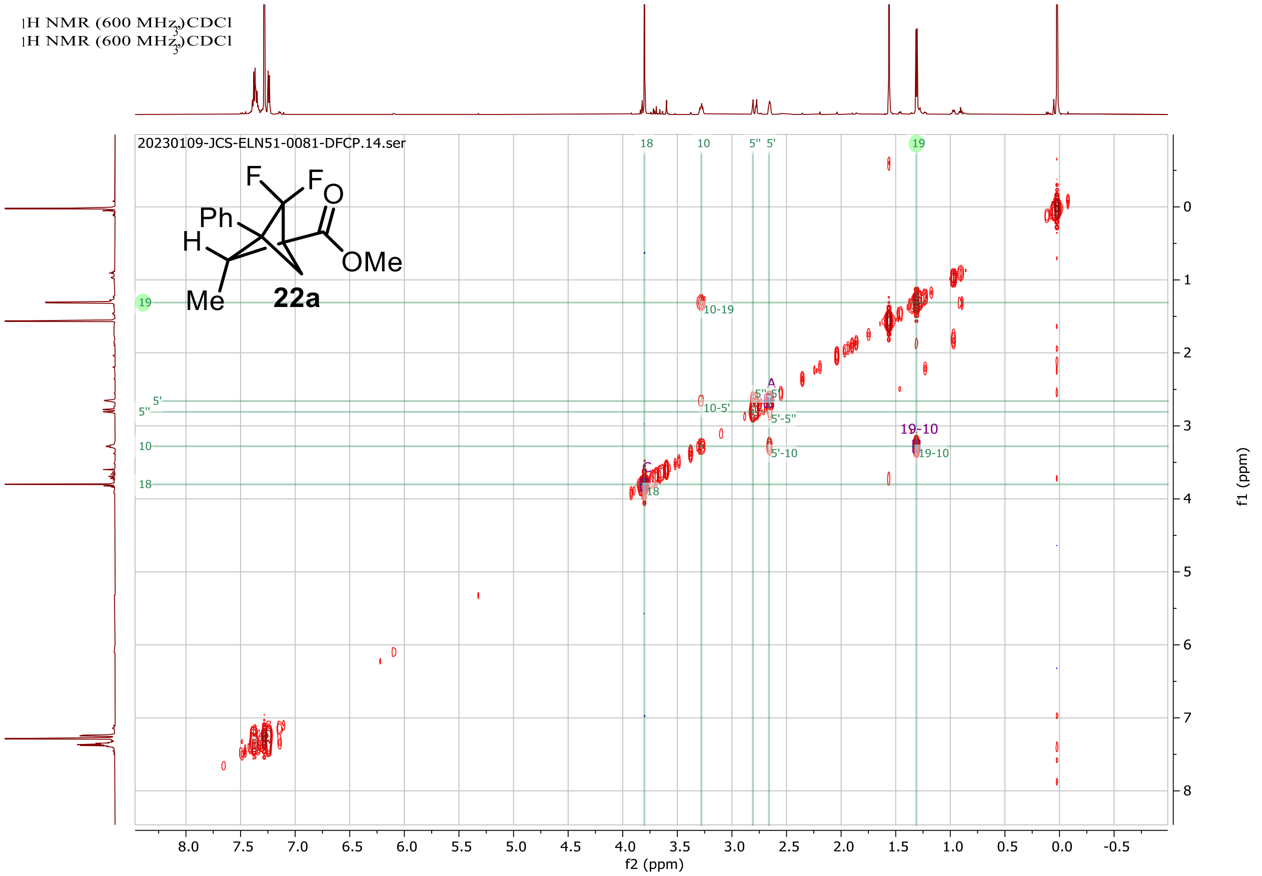
1H NMR (600 MHz)CDCl3
1H NMR (600 MHz)CDCl3
20230109-JCS-ELN51-0081-DFCP.14.ser
22a
f2 (ppm)
f1 (ppm)

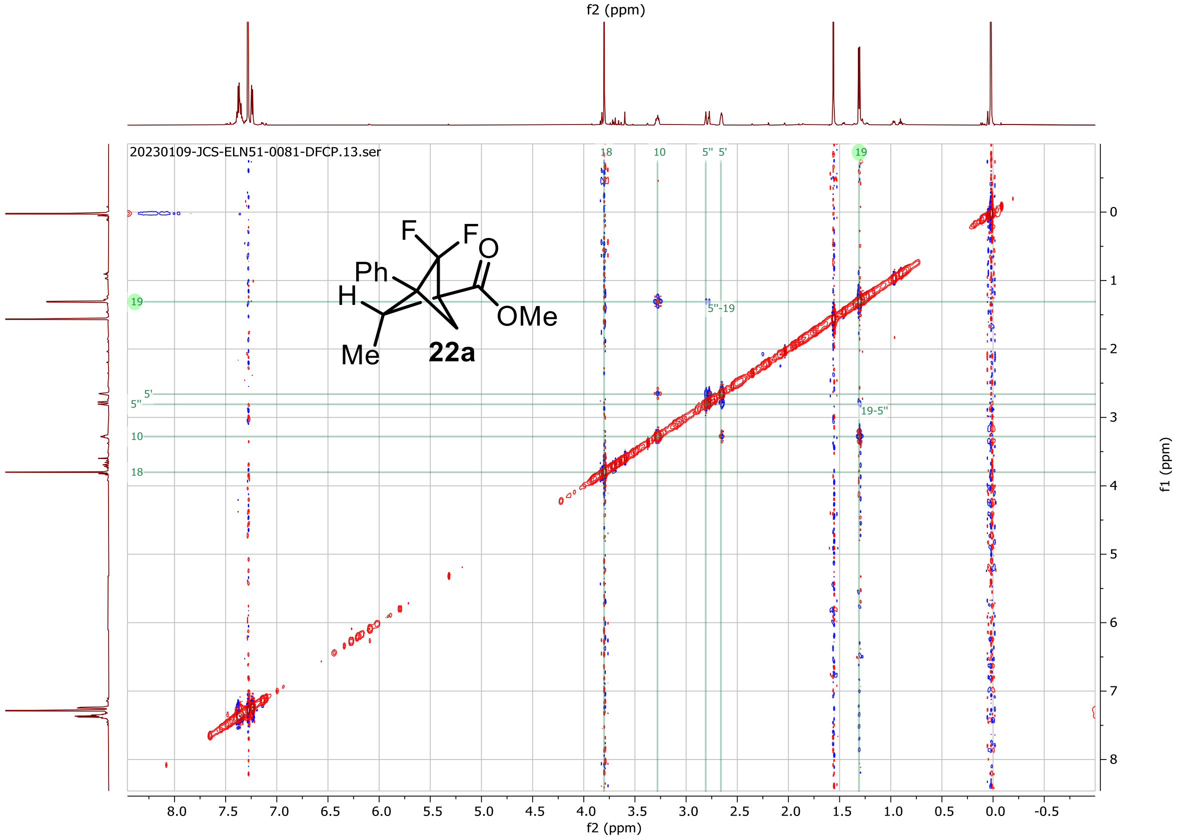
20230109-JCS-ELN51-0081-DFCP.13.ser
22a
f2 (ppm)
f1 (ppm)

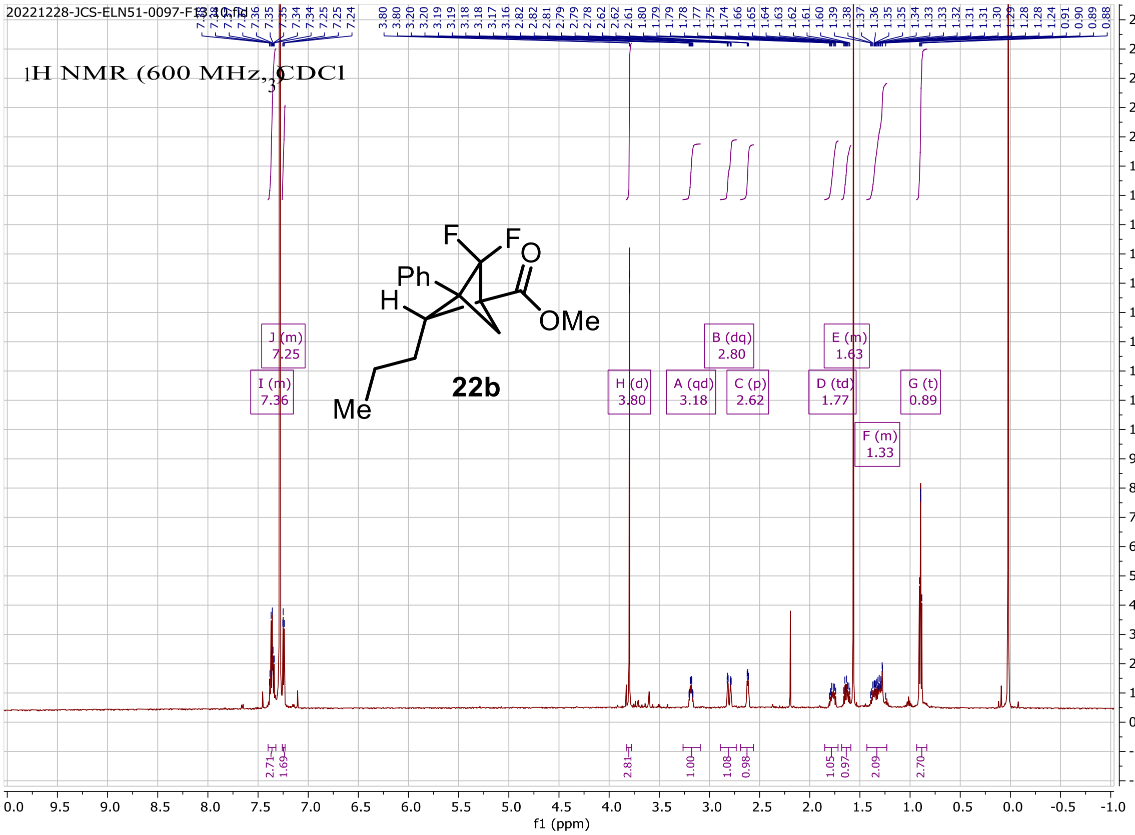
20221228-JCS-ELN51-0097-F13.10.fid
1H NMR (600 MHz, CDCl3)
22b
J (m) 7.25
I (m) 7.36
H (d) 3.80
A (qd) 3.18
B (dq) 2.80
C (p) 2.62
D (td) 1.77
E (m) 1.63
F (m) 1.33
G (t) 0.89
f1 (ppm)

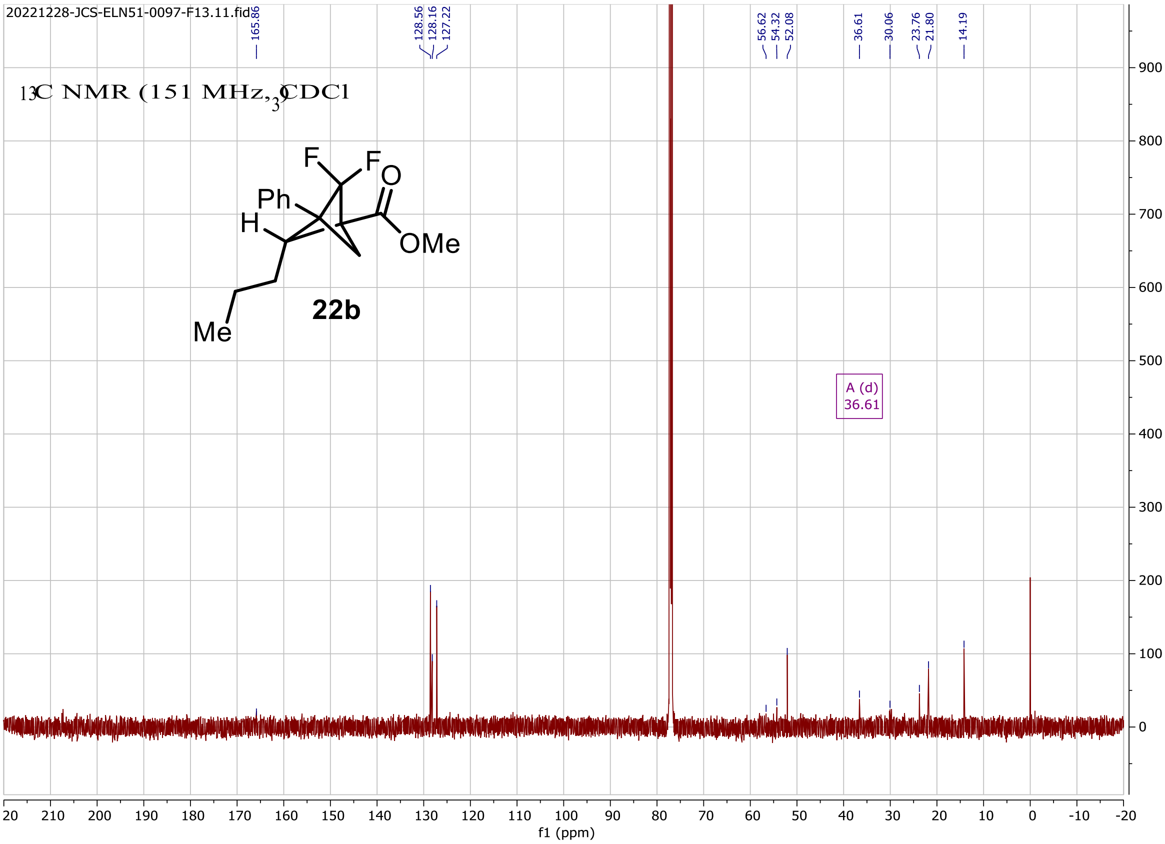
20221228-JCS-ELN51-0097-F13.11.fid
165.86
128.56
128.16
127.22
56.62
54.32
52.08
36.61
30.06
23.76
21.80
14.19
13C NMR (151 MHz, CDCl3)
22b
A (d) 36.61
f1 (ppm)

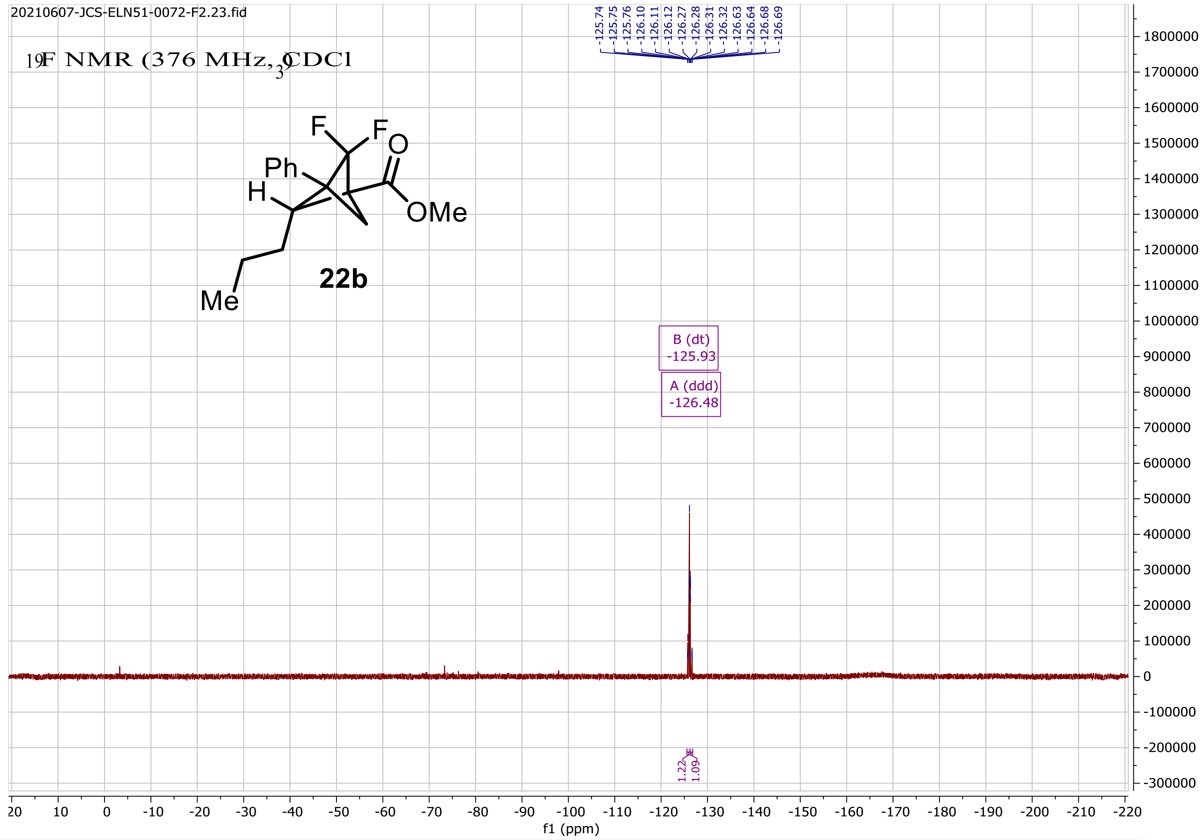

20210607-JCS-ELN51-0072-F2.23.fid
19F NMR (376 MHz, CDCl3)
F
F
O
Ph
H
OMe
Me
22b
-125.74
-125.75
-125.76
-126.10
-126.11
-126.12
-126.27
-126.28
-126.31
-126.32
-126.63
-126.64
-126.68
-126.69
B (dt)
-125.93
A (ddd)
-126.48
1.22
1.09
f1 (ppm)

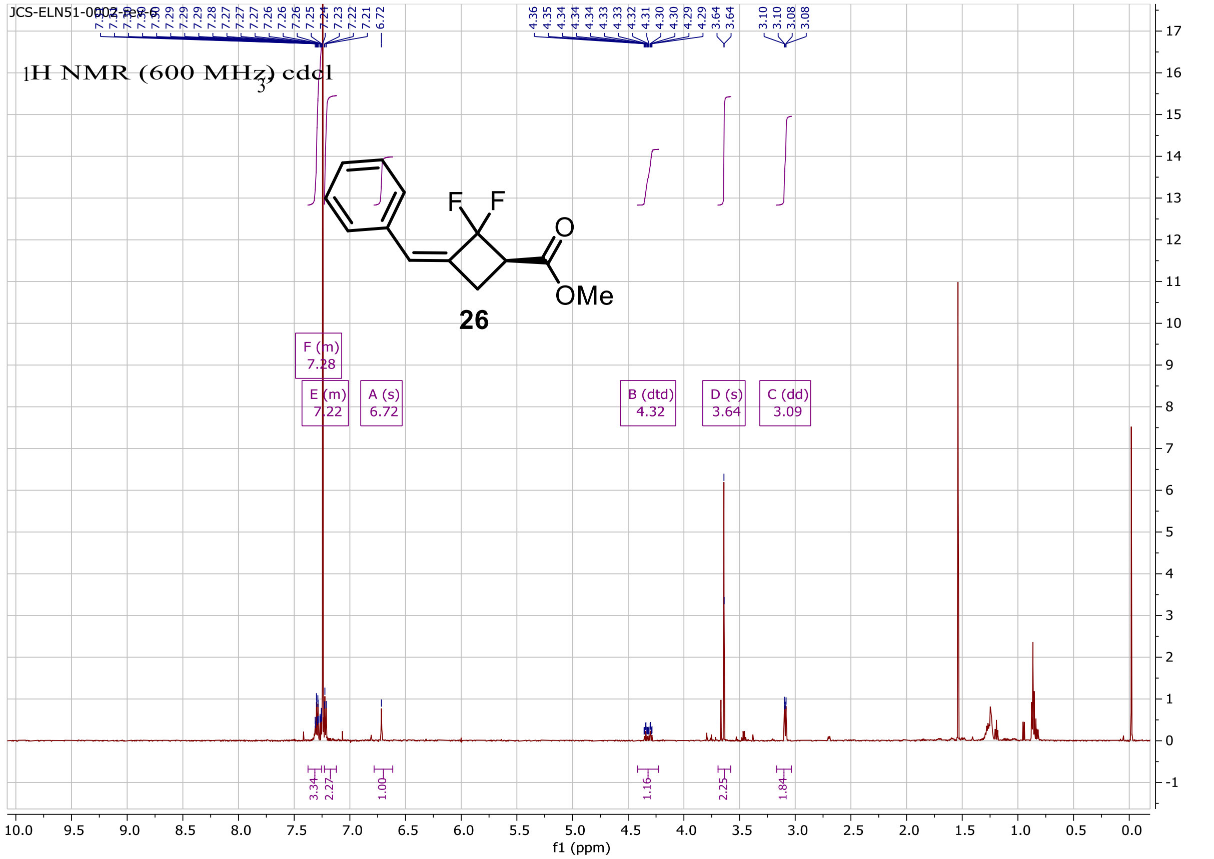

1H NMR (600 MHz) cdcl3
F
F
O
OMe
26
7.29
7.29
7.28
7.27
7.27
7.26
7.26
7.25
7.24
7.23
7.22
7.21
6.72
4.36
4.35
4.34
4.34
4.34
4.33
4.32
4.31
4.30
4.30
4.29
4.29
3.64
3.64
3.10
3.10
3.08
3.08
F (m)
7.28
E (m)
7.22
A (s)
6.72
B (dtd)
4.32
D (s)
3.64
C (dd)
3.09
3.34
2.27
1.00
1.16
2.25
1.84
f1 (ppm)

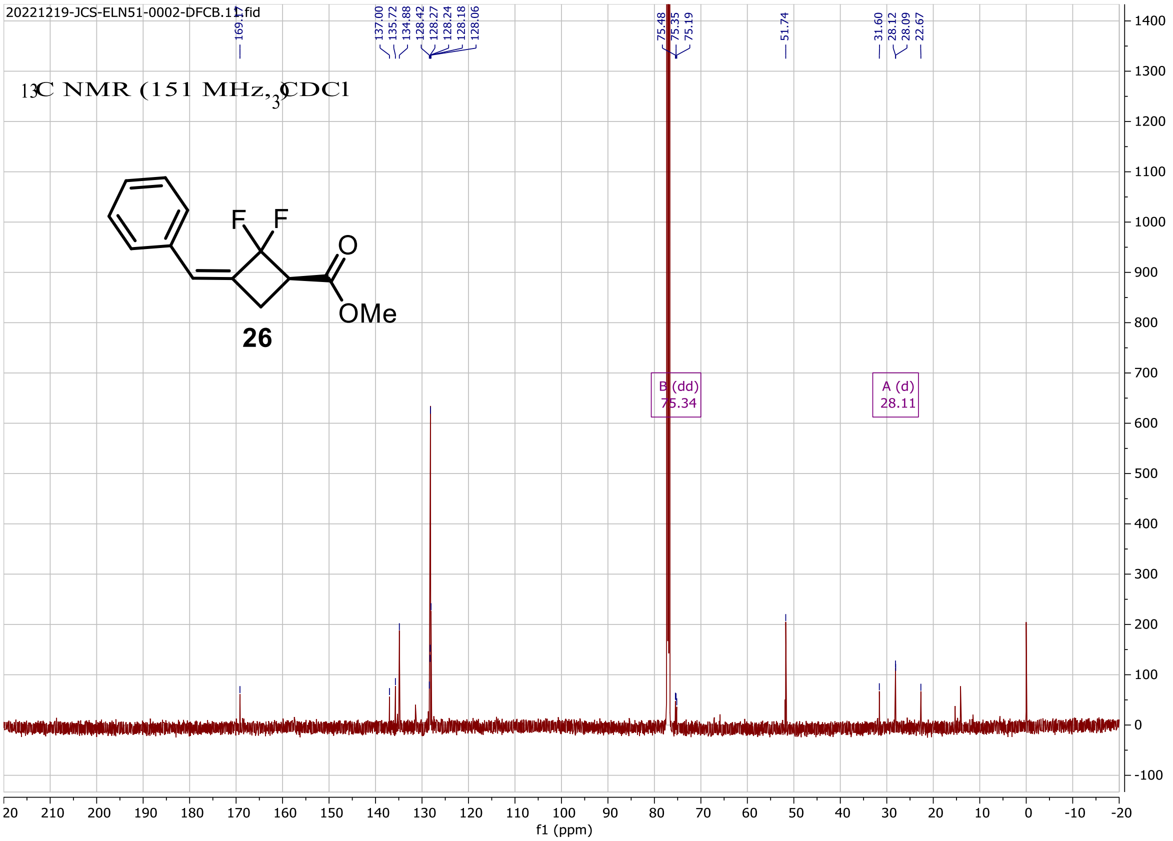

20221219-JCS-ELN51-0002-DFCB.11.fid
13C NMR (151 MHz, CDCl3)
169.17
137.00
135.72
134.88
128.42
128.27
128.24
128.18
128.06
75.48
75.35
75.19
51.74
31.60
28.12
28.09
22.67
B (dd)
75.34
A (d)
28.11
26
OMe
O
F
F
f1 (ppm)

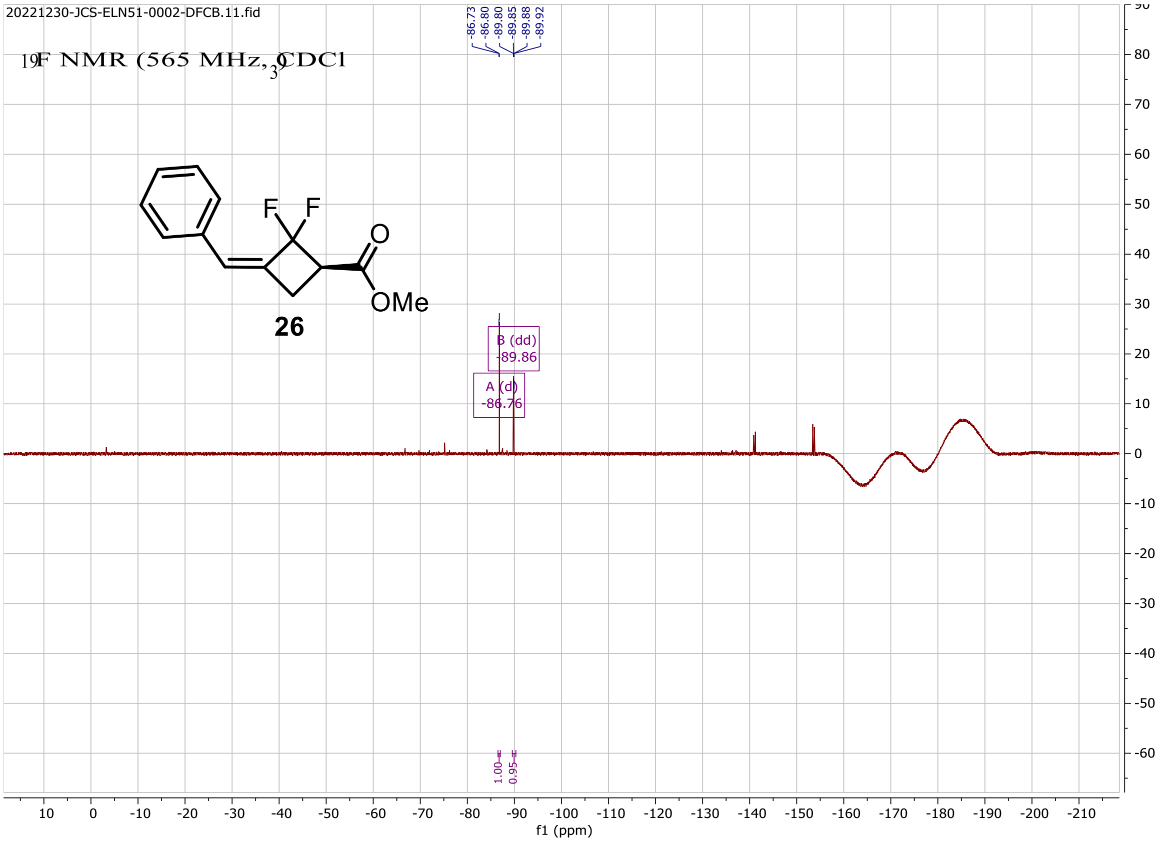

20221230-JCS-ELN51-0002-DFCB.11.fid
19F NMR (565 MHz, CDCl3)
-86.73
-86.80
-89.80
-89.85
-89.88
-89.92
B (dd)
-89.86
A (d)
-86.76
1.00
0.95
26
OMe
O
F
F
f1 (ppm)

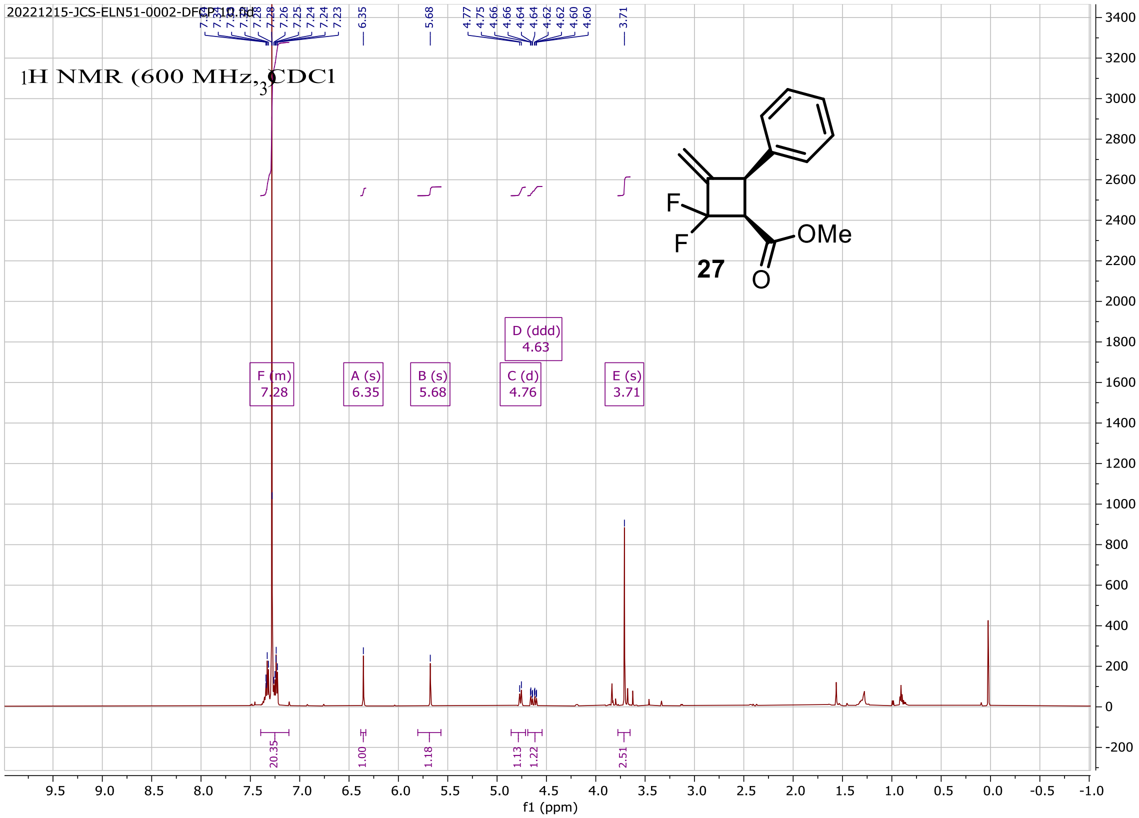
20221215-JCS-ELN51-0002-DFCP.10.fid
1H NMR (600 MHz, CDCl3)
27
F (m) 7.28
A (s) 6.35
B (s) 5.68
C (d) 4.76
D (ddd) 4.63
E (s) 3.71
f1 (ppm)

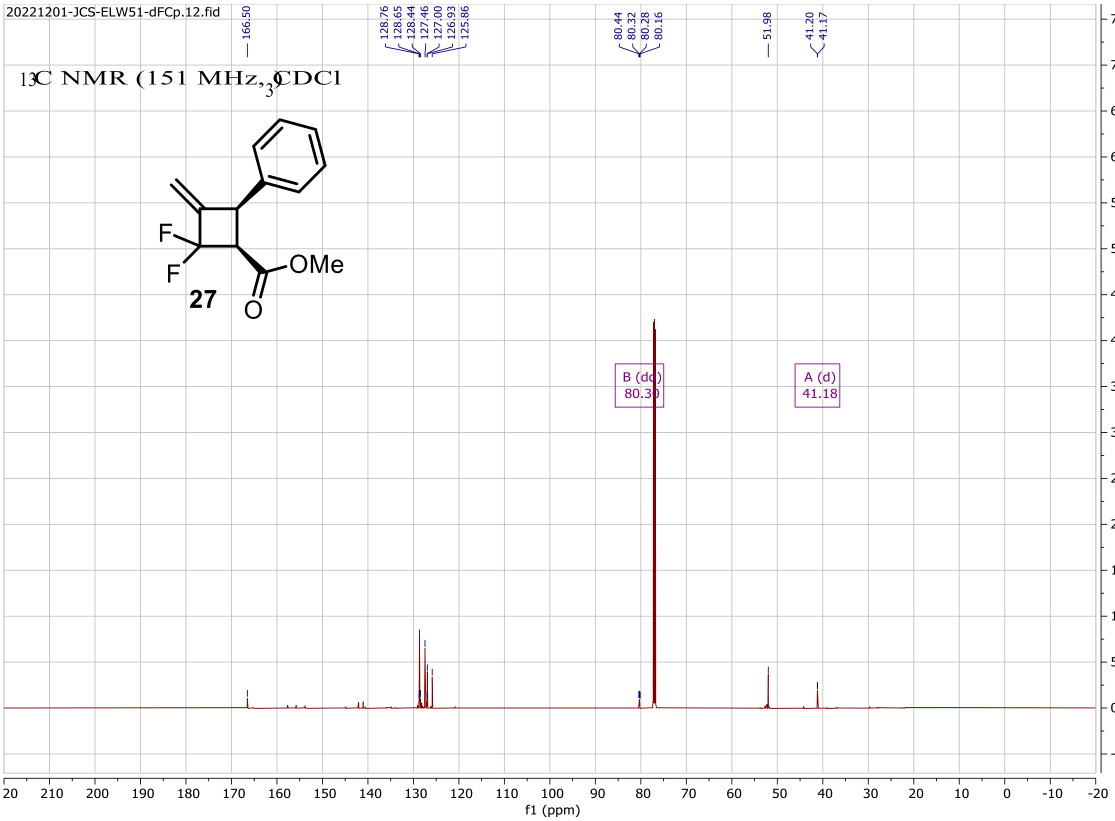
20221201-JCS-ELW51-dFCp.12.fid
13C NMR (151 MHz, CDCl3)
27
B (dd) 80.30
A (d) 41.18
f1 (ppm)

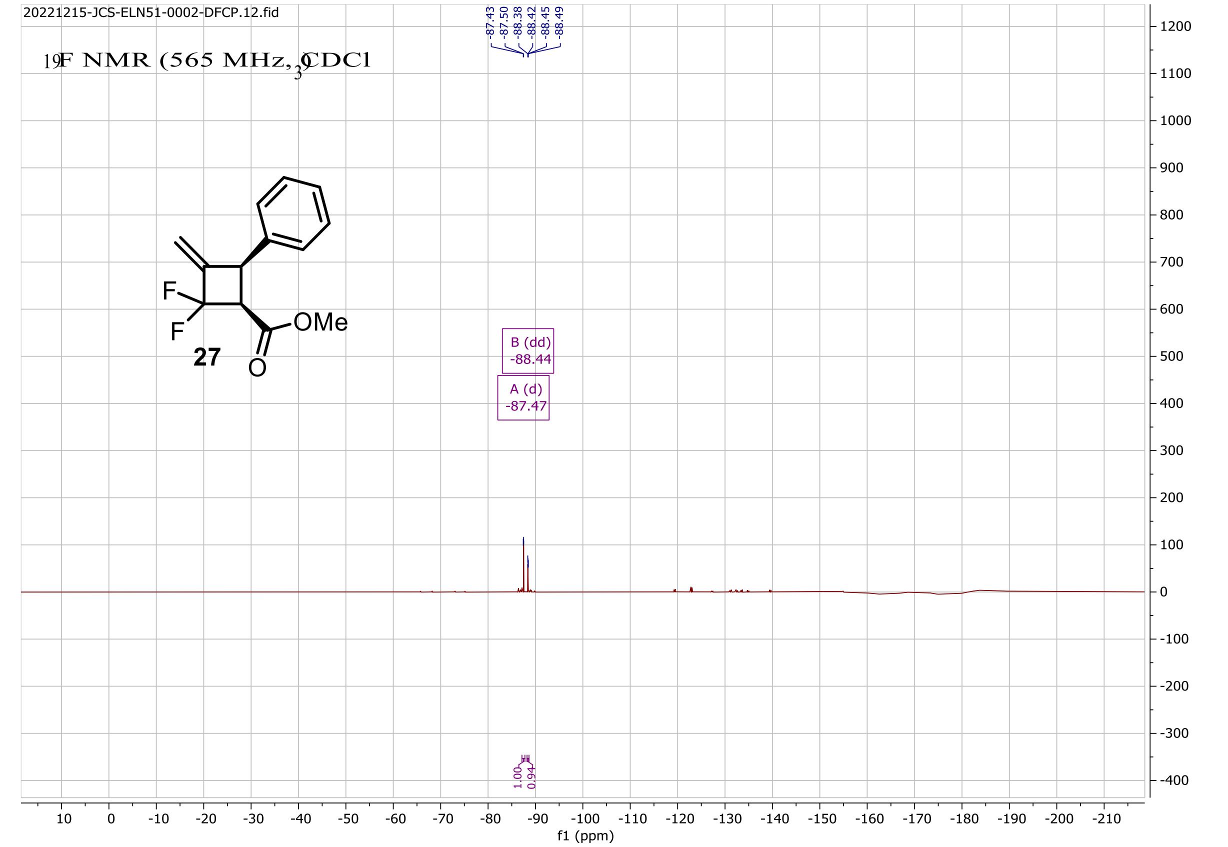
20221215-JCS-ELN51-0002-DFCP.12.fid
19F NMR (565 MHz, CDCl3)
-87.43
-87.50
-88.38
-88.42
-88.45
-88.49
F
F
OMe
O
27
B (dd)
-88.44
A (d)
-87.47
1.00
0.94
f1 (ppm)

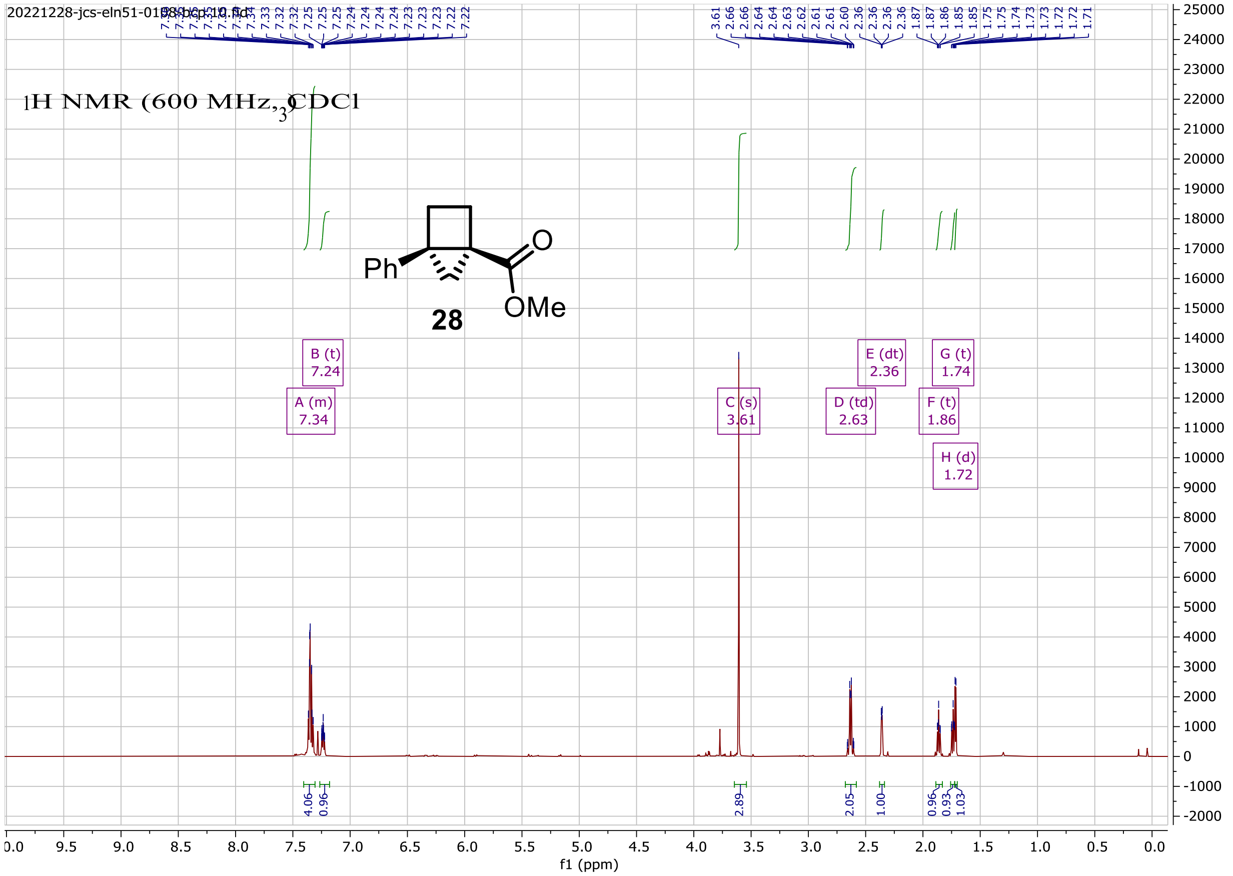
1H NMR (600 MHz, CDCl3)
Ph
O
OMe
28
B (t)
7.24
A (m)
7.34
C (s)
3.61
D (td)
2.63
E (dt)
2.36
F (t)
1.86
G (t)
1.74
H (d)
1.72
4.06
0.96
2.89
2.05
1.00
0.96
0.93
1.03
f1 (ppm)

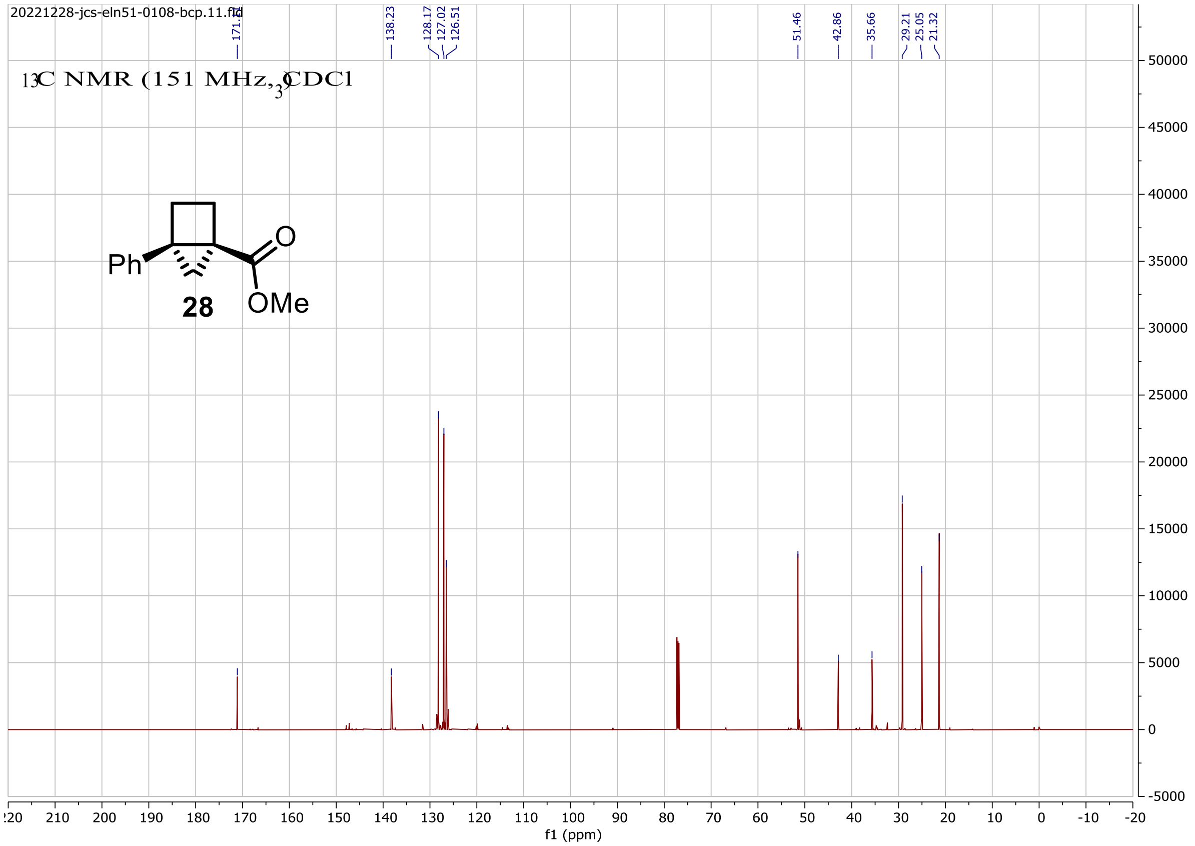
20221228-jcs-eln51-0108-bcp.11.fid
171.12
138.23
128.17
127.02
126.51
51.46
42.86
35.66
29.21
25.05
21.32
13C NMR (151 MHz, CDCl3)
Ph
O
OMe
28
f1 (ppm)

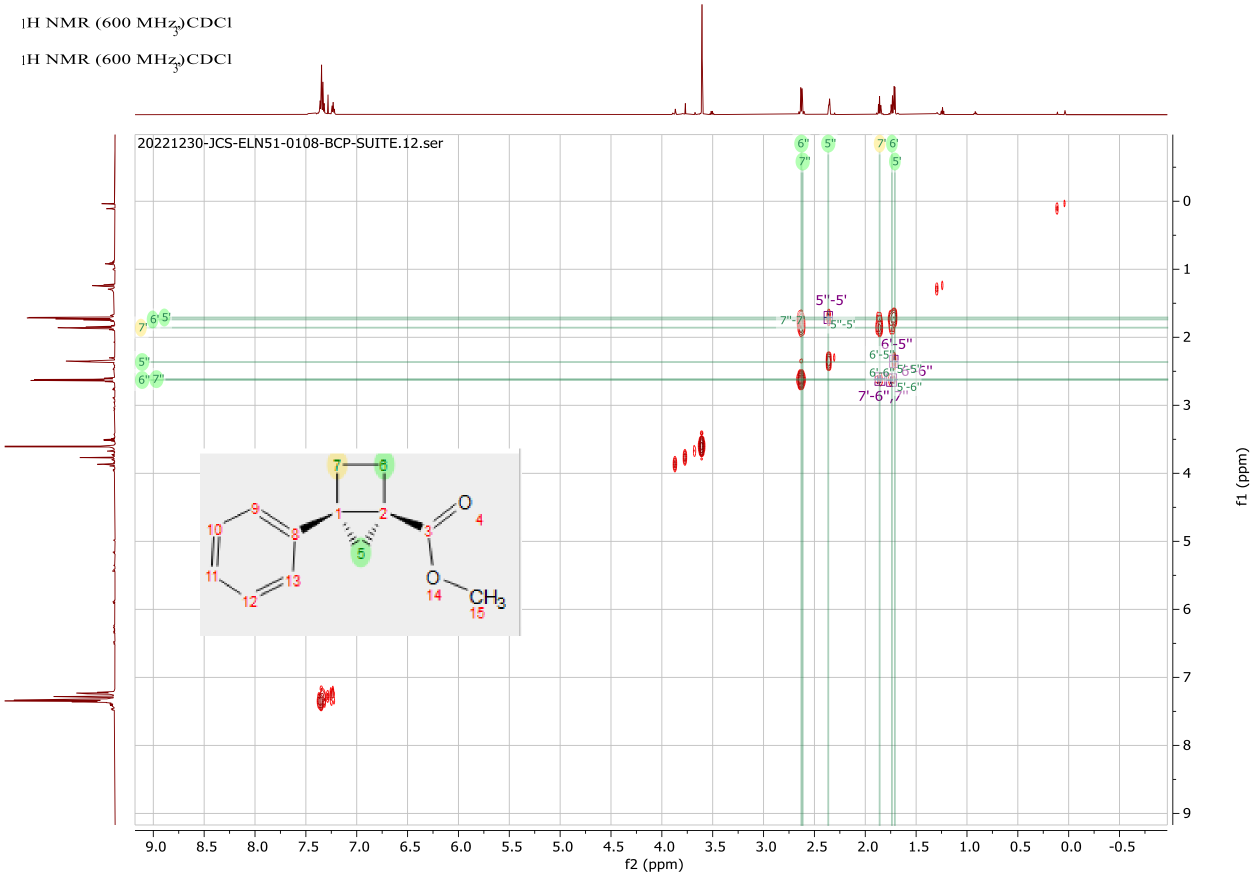
1H NMR (600 MHz, CDCl3)
1H NMR (600 MHz, CDCl3)
20221230-JCS-ELN51-0108-BCP-SUITE.12.ser
f2 (ppm)
f1 (ppm)
CH3

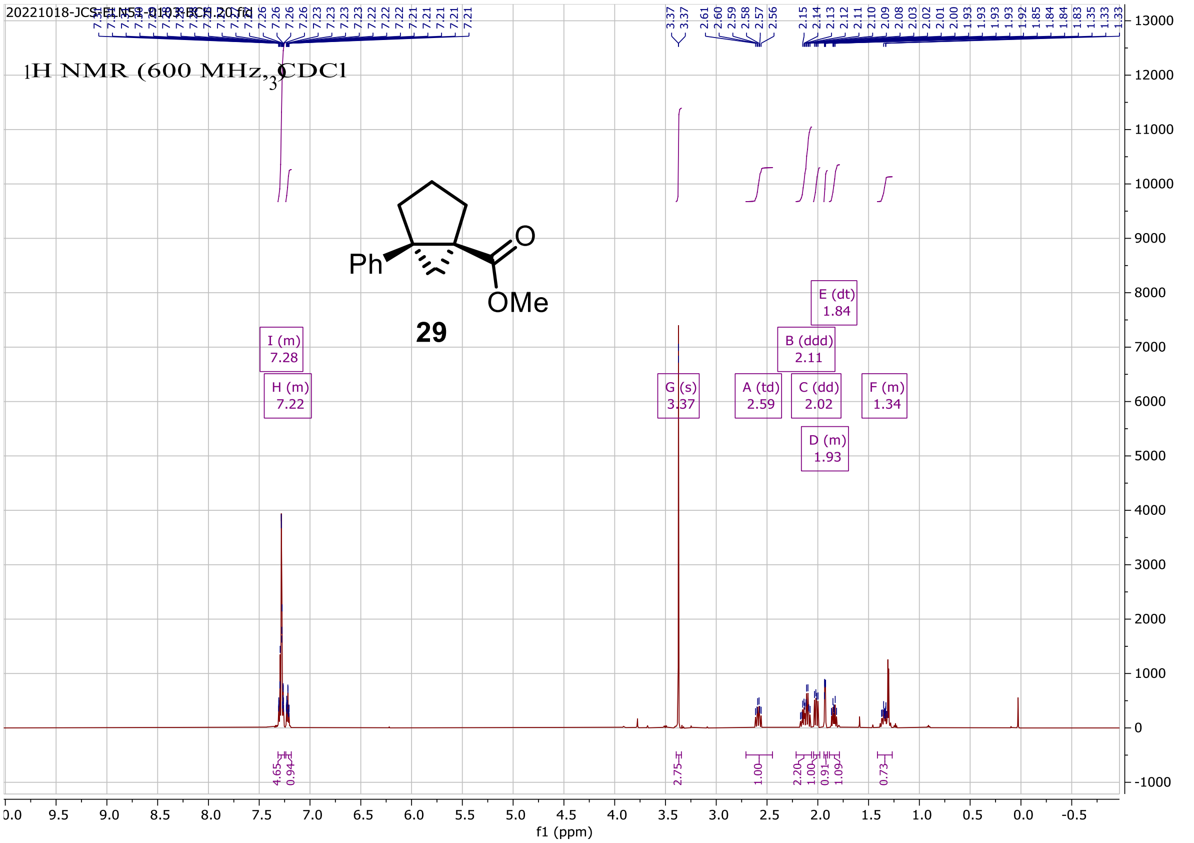
20221018-JCS-ELN51-0103-BCH.20.fid
1H NMR (600 MHz, CDCl3)
29
I (m) 7.28
H (m) 7.22
G (s) 3.37
A (td) 2.59
B (ddd) 2.11
C (dd) 2.02
D (m) 1.93
E (dt) 1.84
F (m) 1.34
f1 (ppm)

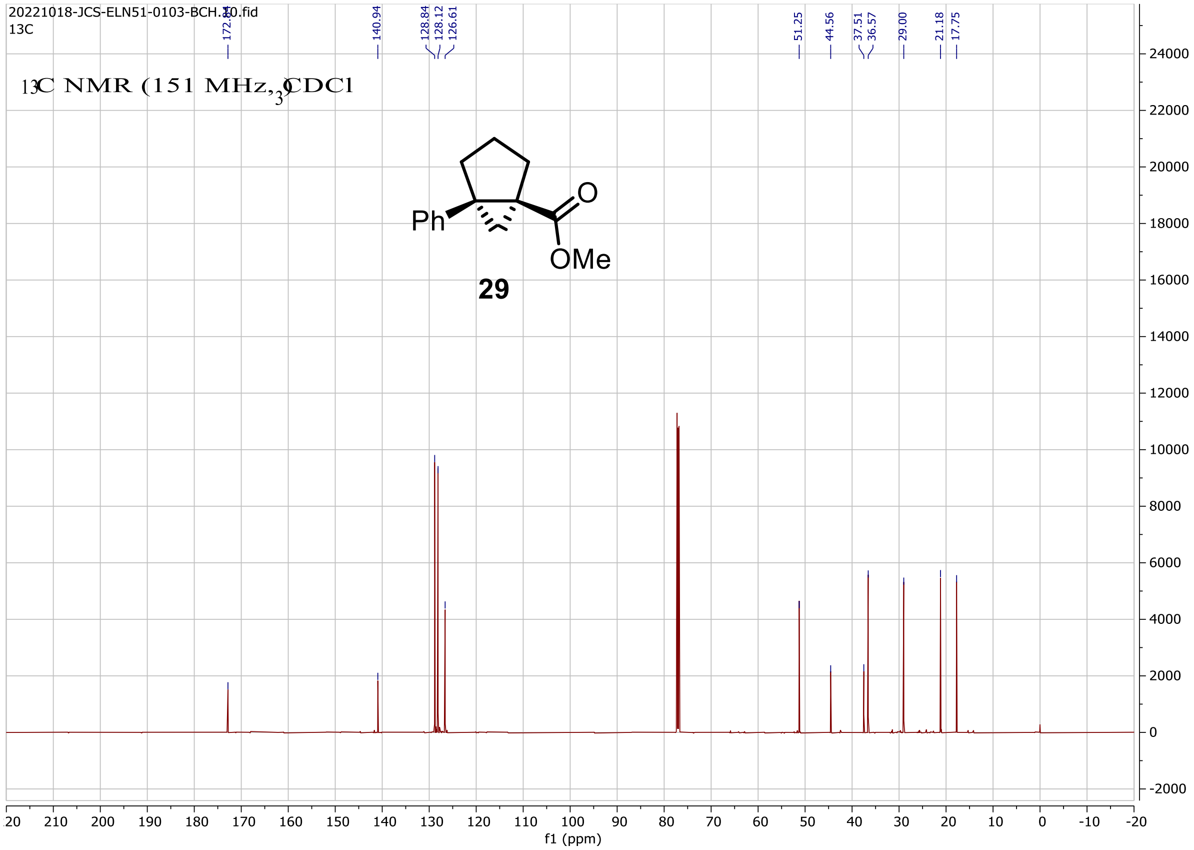
20221018-JCS-ELN51-0103-BCH.10.fid
13C
13C NMR (151 MHz, CDCl3)
140.94
128.84
128.12
126.61
51.25
44.56
37.51
36.57
29.00
21.18
17.75
29
f1 (ppm)

9. HPLC/SFC Data:

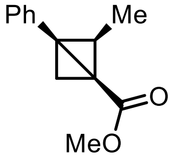

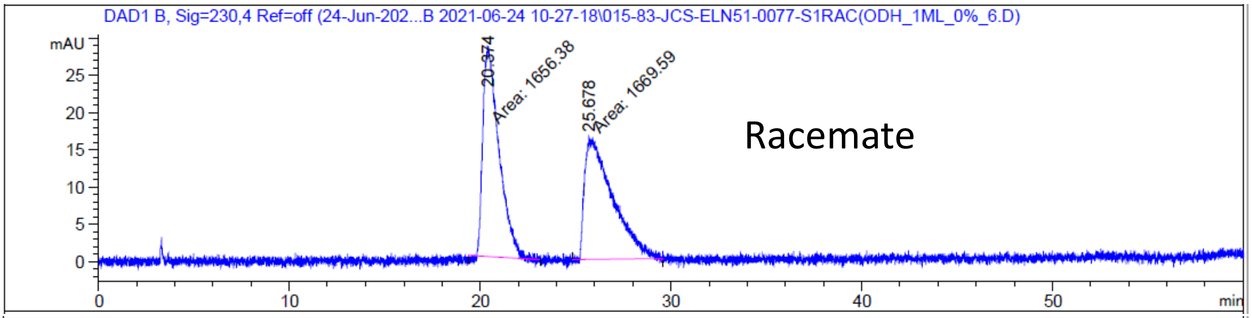

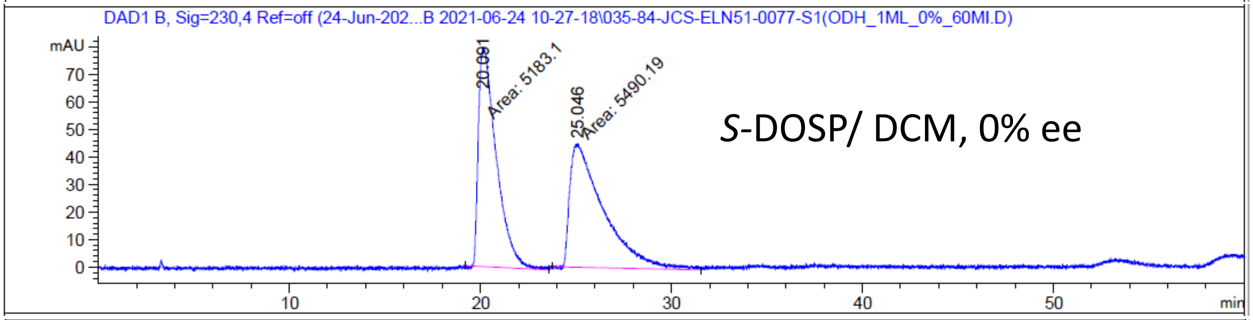

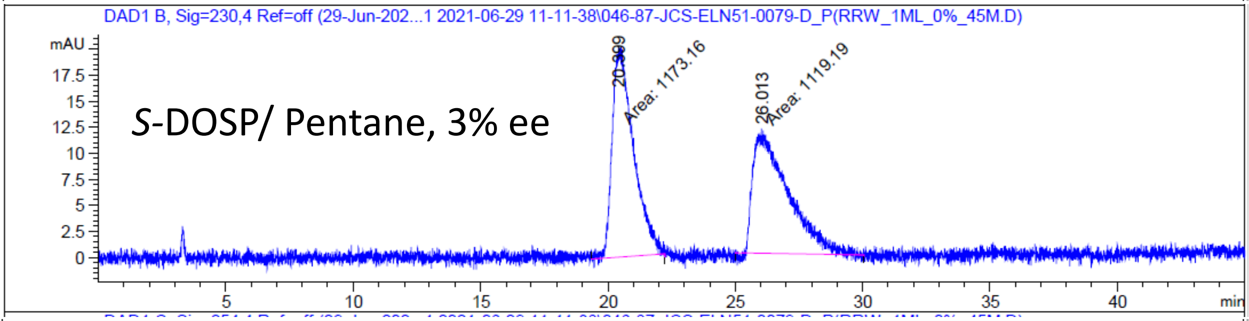

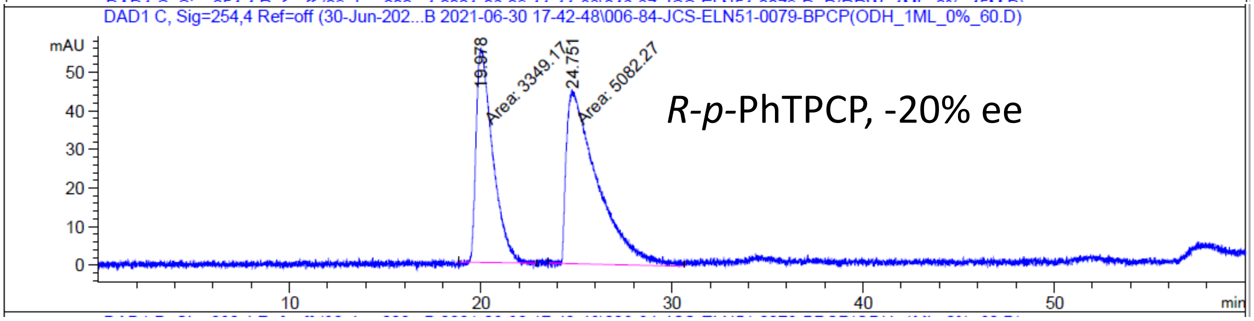

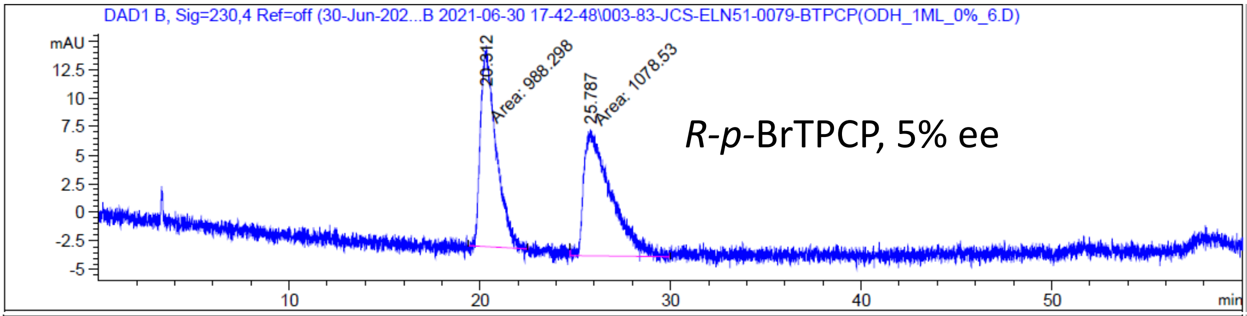
DAD1 B, Sig=230,4 Ref=off (30-Jun-202...B 2021-06-30 17-42-48\003-83-JCS-ELN51-0079-BTPCP(ODH_1ML_0%_6.D)
Area: 988.298
25.787
Area: 1078.53
R-*p*-BrTPCP, 5% ee

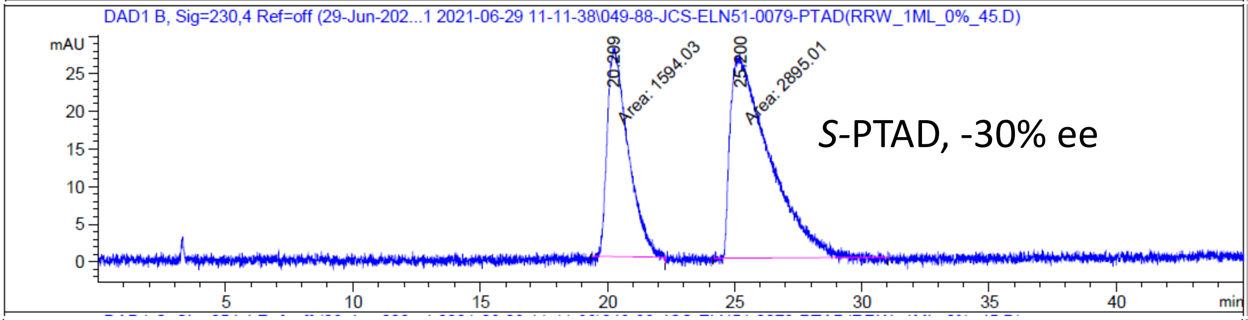
DAD1 B, Sig=230,4 Ref=off (29-Jun-202...1 2021-06-29 11-11-38\049-88-JCS-ELN51-0079-PTAD(RRW_1ML_0%_45.D)
Area: 1594.03
Area: 2895.01
S-PTAD, -30% ee

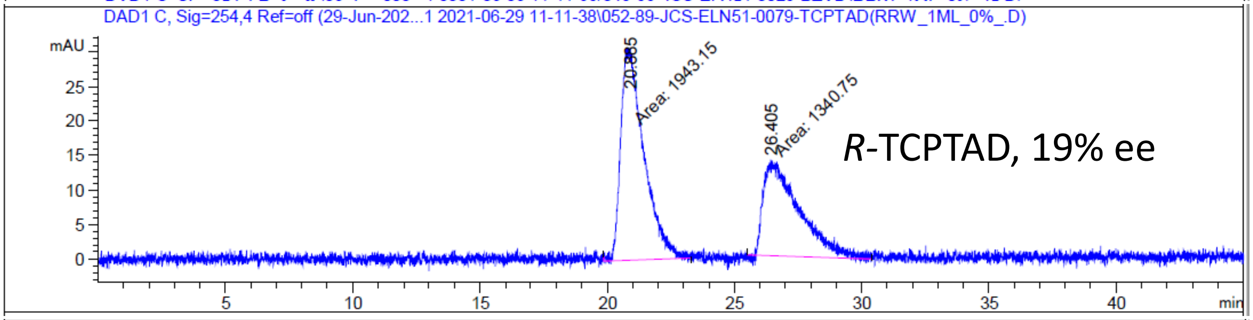
DAD1 C, Sig=254,4 Ref=off (29-Jun-202...1 2021-06-29 11-11-38\052-89-JCS-ELN51-0079-TCPTAD(RRW_1ML_0%_.D)
Area: 1943.15
26.405
Area: 1340.75
R-TCPTAD, 19% ee

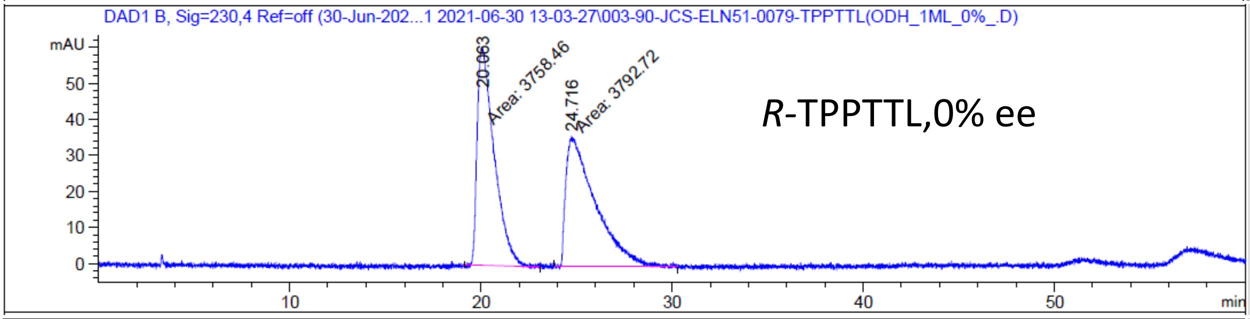
DAD1 B, Sig=230,4 Ref=off (30-Jun-202...1 2021-06-30 13-03-27\003-90-JCS-ELN51-0079-TPPTTL(ODH_1ML_0%_.D)
Area: 3758.46
24.716
Area: 3792.72
R-TPPTTL,0% ee

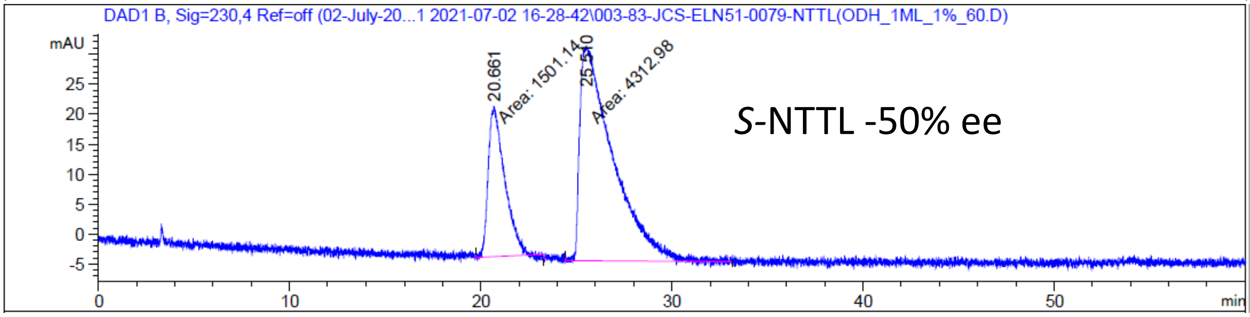
DAD1 B, Sig=230,4 Ref=off (02-July-20...1 2021-07-02 16-28-42\003-83-JCS-ELN51-0079-NTTL(ODH_1ML_1%_60.D)
20.661
Area: 1501.14
Area: 4312.98
S-NTTL -50% ee

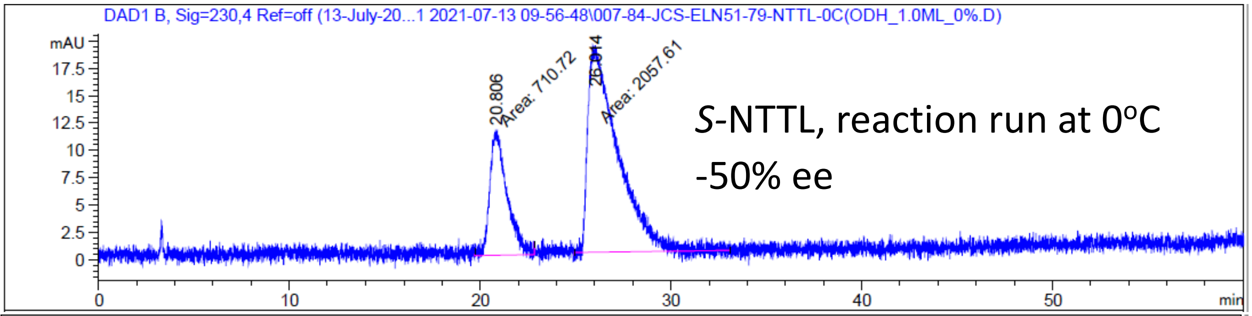

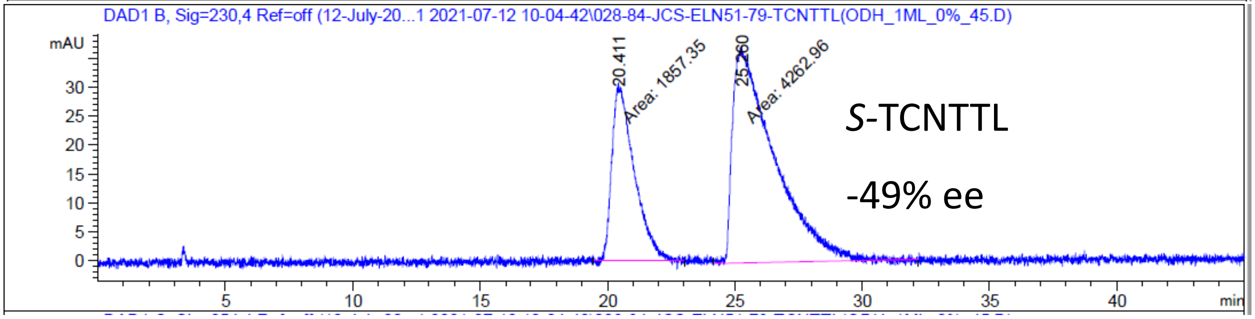

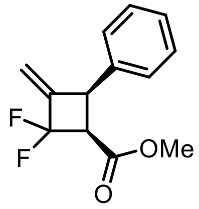

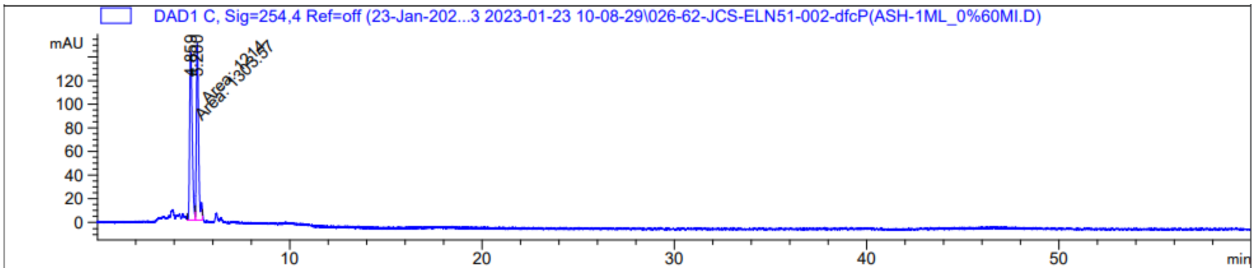

Signal 3: DAD1 C, Sig=254,4 Ref=off

Peak #	RetTime [min]	Type	Width [min]	Area [mAU*s]	Height [mAU]	Area %
1	4.859	MM	0.1528	1303.57104	142.19475	51.7789
2	5.200	MM	0.1353	1213.99963	149.57280	48.2211

Totals : 2517.57068 291.76755

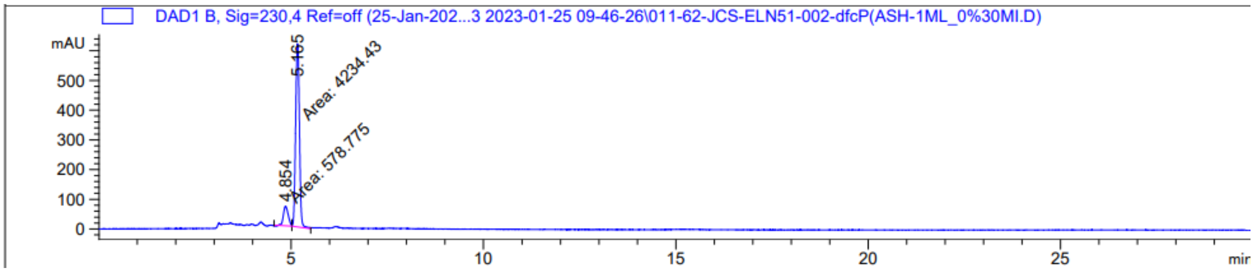

Signal 2: DAD1 B, Sig=230,4 Ref=off

Peak #	RetTime [min]	Type	Width [min]	Area [mAU*s]	Height [mAU]	Area %
1	4.854	MM	0.1462	578.77502	65.97089	12.0247
2	5.165	MM	0.1144	4234.42871	616.86493	87.9753
Totals :				4813.20374	682.83582	

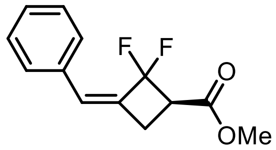

Racemate:

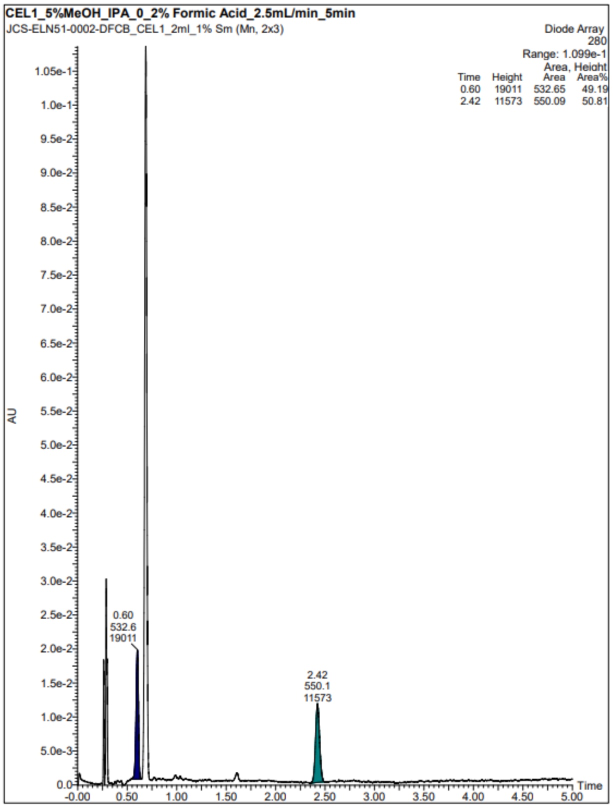

Chiral:

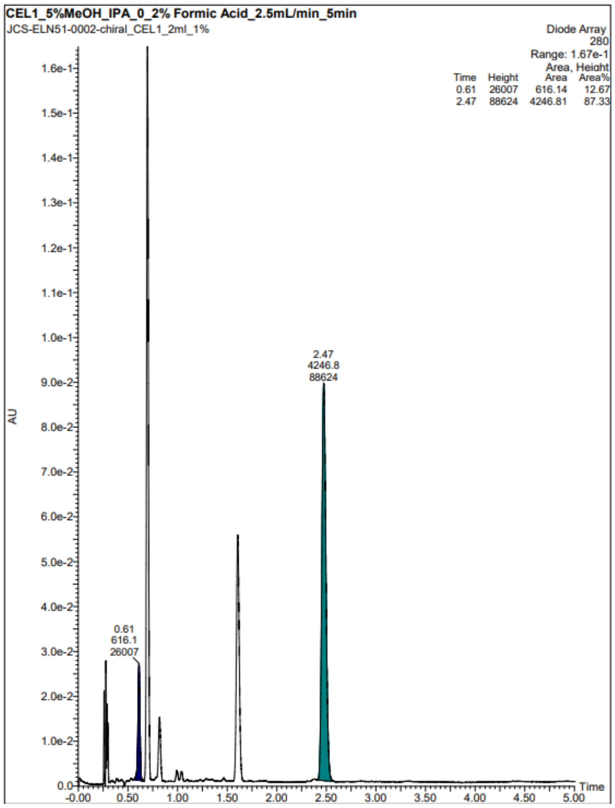

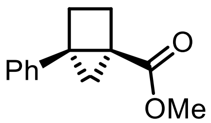

Racemate:

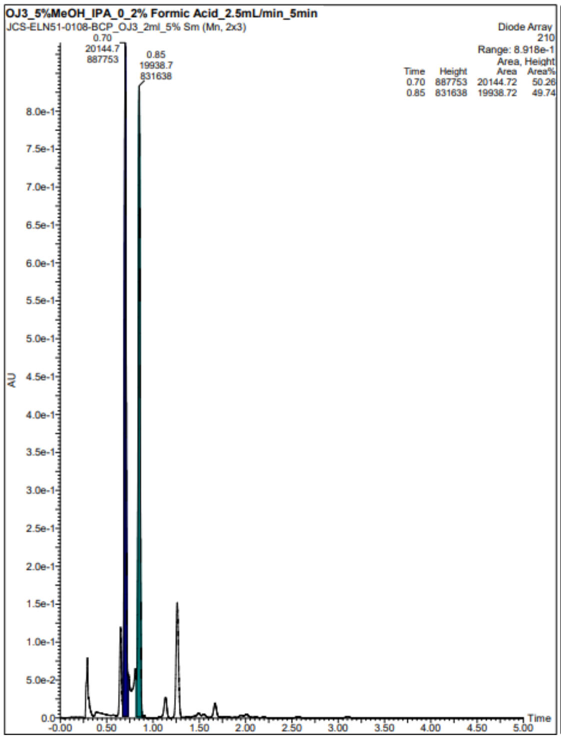

***S-p*-BrTPCP: 7% ee**

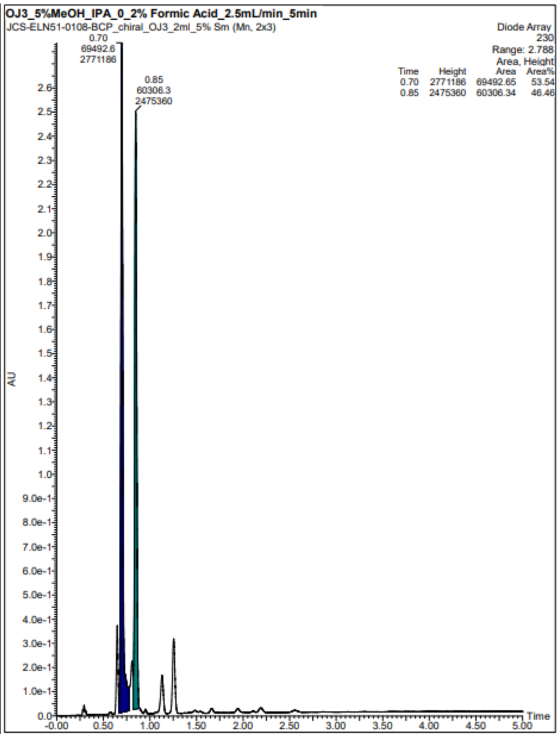

***R*-NTTL: 11% ee**

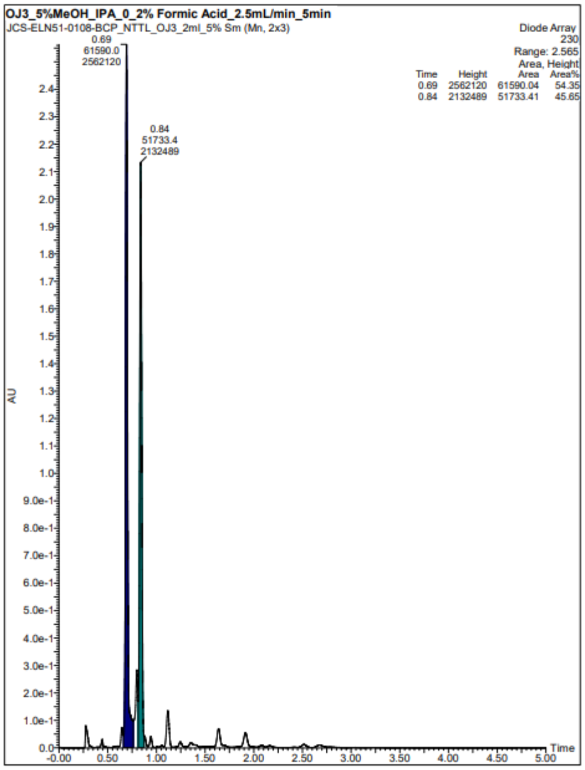

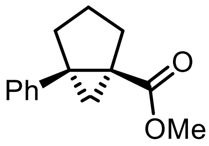

Racemate:

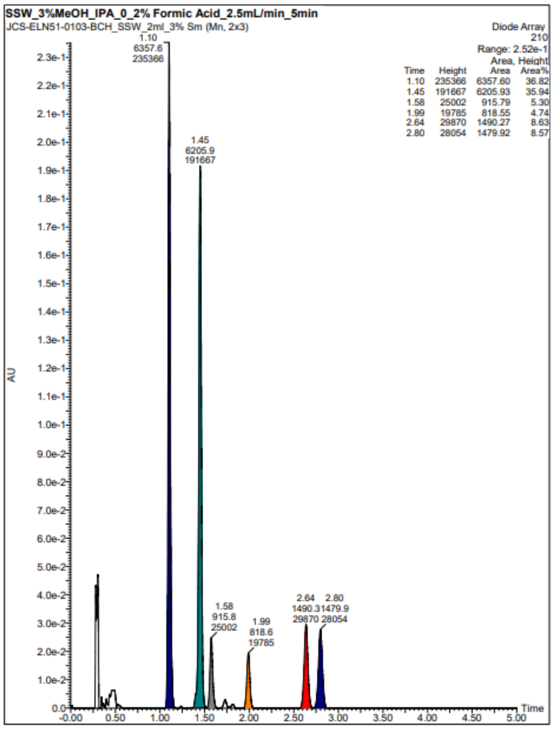

***S*-*p*-BrTPCP: 65% ee**

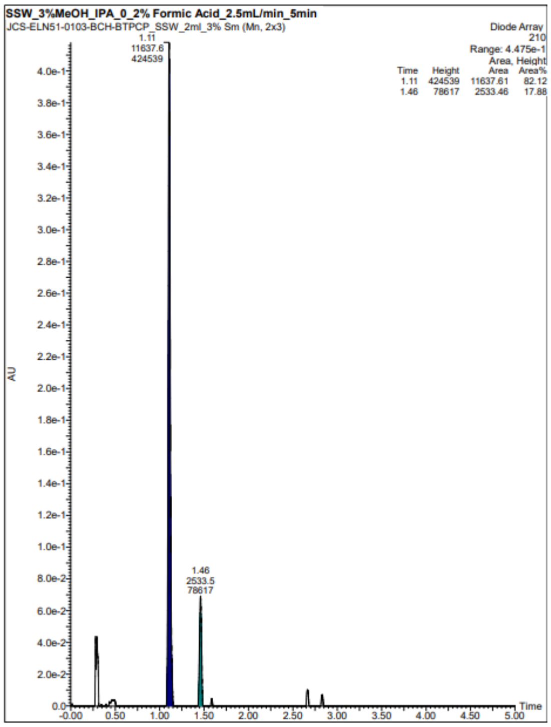

***R*-NTTL:**

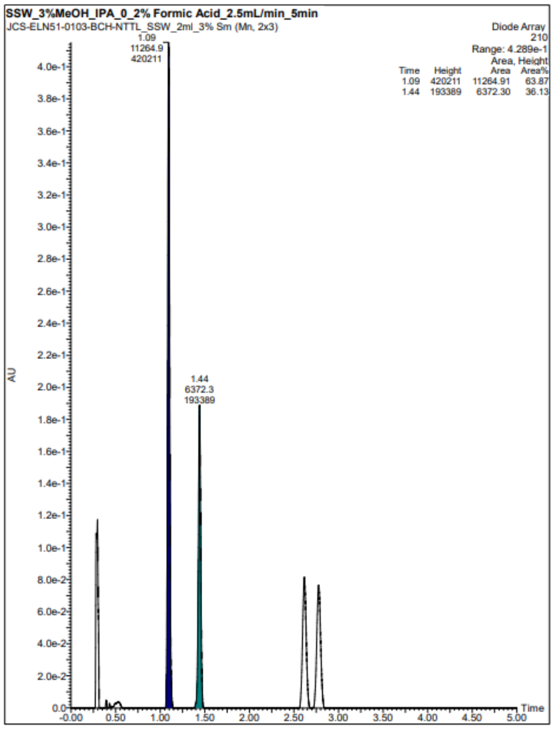

10. References

1. Green, S. P.; Wheelhouse, K. M.; Payne, A. D.; Hallett, J. P.; Miller, P. W.; Bull, J. A., Thermal stability and explosive hazard assessment of diazo compounds and diazo transfer reagents. *Org. Process Res. Dev.* **2019,** *24* (1), 67-84.
2. Chauhan, D. P.; Varma, S. J.; Gudem, M.; Panigrahi, N.; Singh, K.; Hazra, A.; Talukdar, P., Intramolecular cascade rearrangements of enynamine derived ketenimines: access to acyclic and cyclic amidines. *Org. Biomol. Chem.* **2017,** *15* (22), 4822-4830.
3. Malkov, A. V.; Czemerys, L.; Malyshev, D. A., Vanadium-catalyzed asymmetric epoxidation of allylic alcohols in water. *J. Org. Chem.* **2009,** *74* (9), 3350-3355.
4. Qin, C.; Davies, H. M., Enantioselective synthesis of 2-arylbicyclo [1.1. 0] butane carboxylates. *Org. Lett.* **2013,** *15* (2), 310-313.
5. Bychek, R. M.; Hutskalova, V.; Bas, Y. P.; Zaporozhets, O. A.; Zozulya, S.; Levterov, V. V.; Mykhailiuk, P. K., Difluoro-substituted bicyclo [1.1. 1] pentanes for medicinal chemistry: Design, synthesis, and characterization. *J. Org. Chem.* **2019,** *84* (23), 15106-15117.
